# Amyloid precursor protein interaction network in human testis: sentinel proteins for male reproduction

**DOI:** 10.1186/s12859-014-0432-9

**Published:** 2015-01-16

**Authors:** Joana Vieira Silva, Sooyeon Yoon, Sara Domingues, Sofia Guimarães, Alexander V Goltsev, Edgar Figueiredo da Cruz e Silva, José Fernando F Mendes, Odete Abreu Beirão da Cruz e Silva, Margarida Fardilha

**Affiliations:** Laboratory of Signal Transduction, Centre for Cell Biology, Health Sciences Department and Biology Department, University of Aveiro, 3810-193 Aveiro, Portugal; Department of Physics, I3N, University of Aveiro, 3810-193 Aveiro, Portugal; Laboratory of Neurosciences, Centre for Cell Biology, Health Sciences Department and Biology Department, University of Aveiro, 3810-193 Aveiro, Portugal; Centro de Biologia Celular, SACS, Edifício 30, Universidade de Aveiro, 3810-193 Aveiro, Portugal

**Keywords:** Amyloid Precursor Protein, Human Testis, Male Reproduction, Protein-Protein Interaction Network, Yeast Two-Hybrid

## Abstract

**Background:**

Amyloid precursor protein (APP) is widely recognized for playing a central role in Alzheimer's disease pathogenesis. Although APP is expressed in several tissues outside the human central nervous system, the functions of APP and its family members in other tissues are still poorly understood. APP is involved in several biological functions which might be potentially important for male fertility, such as cell adhesion, cell motility, signaling, and apoptosis. Furthermore, APP superfamily members are known to be associated with fertility. Knowledge on the protein networks of APP in human testis and spermatozoa will shed light on the function of APP in the male reproductive system.

**Results:**

We performed a Yeast Two-Hybrid screen and a database search to study the interaction network of APP in human testis and sperm. To gain insights into the role of APP superfamily members in fertility, the study was extended to APP-like protein 2 (APLP2). We analyzed several topological properties of the APP interaction network and the biological and physiological properties of the proteins in the APP interaction network were also specified by gene ontologyand pathways analyses. We classified significant features related to the human male reproduction for the APP interacting proteins and identified modules of proteins with similar functional roles which may show cooperative behavior for male fertility.

**Conclusions:**

The present work provides the first report on the APP interactome in human testis. Our approach allowed the identification of novel interactions and recognition of key APP interacting proteins for male reproduction, particularly in sperm-oocyte interaction.

**Electronic supplementary material:**

The online version of this article (doi:10.1186/s12859-014-0432-9) contains supplementary material, which is available to authorized users.

## Background

Amyloid precursor protein (APP) is known as a pathological hallmark of Alzheimer's disease (AD). Nevertheless, APP, a type I transmembrane glycoprotein consisting of a large extracellular domain, a single transmembrane domain, and a short cytoplasmic tail, is expressed ubiquitously and given its receptor-like and adhesive characteristics may play important roles outside the nervous system. In fact, we have previously showed that APP is present in spermatozoa [1]. The APP superfamily includes APP and APP-like proteins (APLP) 1 and 2. Alternative splicing of the APP mRNA produces eight isoforms, ranging in size from 677–770 amino acids [2]. Alternative splicing produces four APLP1 and two APLP2 protein isoforms. Although some isoforms may be cell type specific, APP and APLP2 are ubiquitously expressed. In contrast, APLP1 is expressed selectively in the nervous system [3]. Only APP, but not APLP1 and 2, contains a sequence encoding the beta-amyloid domain. The transmembranar structure of APP is consistent with a role of APP as a receptor or a mediator of extracellular interactions. It has been suggested that APP may have CAM (Cell Adhesion Molecule) and SAM (Substrate Adhesion Molecule) like activities [4].

Various lines of evidence implicate APP and APLP2 in fertility. APP was shown to be expressed in rat testis and localized in the acrosome region and growing tail of spermatids in the seminiferous tubules [5]. Knock-out mice, homozygotes to either APP(−/−) or APLP2(−/−) were fertile, but mice with the deletion of both APP(−/−) and APLP2(−/−) were infertile (9 of 10 females and all males) [6]. We previously characterized the subcellular distribution of the APP superfamily members in spermatozoa using a variety of antibodies that either recognizes APP-specific epitopes or the epitopes shared with other APP family members [1]. The presence of APP superfamily members along the entire length of the tail may be related to signaling events involved in sperm motility, whereas their presence in the head and particularly in the equatorial region suggests their involvement in sperm-oocyte interaction [1]. These results not only were consistent with the previous localization of APLP2 in mammalian sperm, but also prove the presence of APP itself in human sperm. APP and APLPs distribution only partially overlap suggesting that, besides a common role, they might also have distinct functions in spermatozoa. A human sperm transmembrane protein initially termed YWK-II (later shown to be an APLP2 homologue) was shown to be involved in fertilization [7,8]. The YWK-II gene was shown to be expressed in germ cells at various stages of differentiation and in the plasma membrane enveloping the acrosome of mature spermatozoa [7].

The discovery of tissue-specific interacting proteins can lead to the identification of pathways for the APP family members associated with testis and sperm functions. Hence, we performed a Yeast Two-Hybrid (YTH) screen of a human testis cDNA library using APP as bait. A comprehensive bioinformatic analysis was also performed using the APP interacting proteins identified in the YTH in addition to the proteins selected from public protein-protein interactions (PPI) databases (DB) and published APP interactome data [9] associated with testis/sperm. APLP2 interacting partners were also included. Additionally, protein interaction maps were constructed allowing the visualization of PPI data as a connectivity graph and the data was subjected to a statistical analysis based on the complex network theory. The advantage of this approach is that it allows the study not only of the local properties of proteins in the network, but also their global structural characteristics in the entire network of PPI. We reveal that proteins with similar biological functions are tightly connected to each other and form dense groups (modules or *k-*cores) in the networks. Also, the function, cellular distribution and pathways were analyzed and significant features were classified.

## Results

In this study, we characterized the testis/spermatozoa interactome of APP using a network-based approach.

### Identification of APP interacting proteins in human testis by Yeast Two-Hybrid screening

Nowadays the YTH methodology is a very robust technique to identify PPI [10-13]. The method that we use has been highly improved and overcomes the initial problems of the YTH, e.g. the appearance of false positive or false negative interactions [14], since, for instance, we use four reporter genes with different strength promoters [10-13].

**Figure 1:**
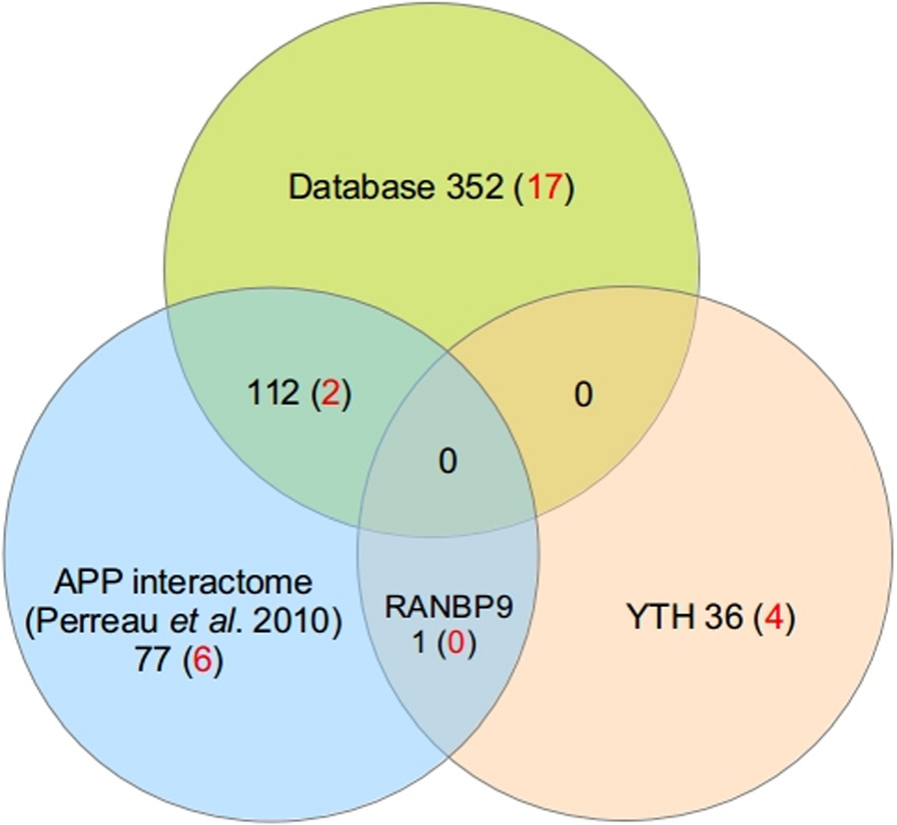
**Experimental methodology.**

**Figure 2:**
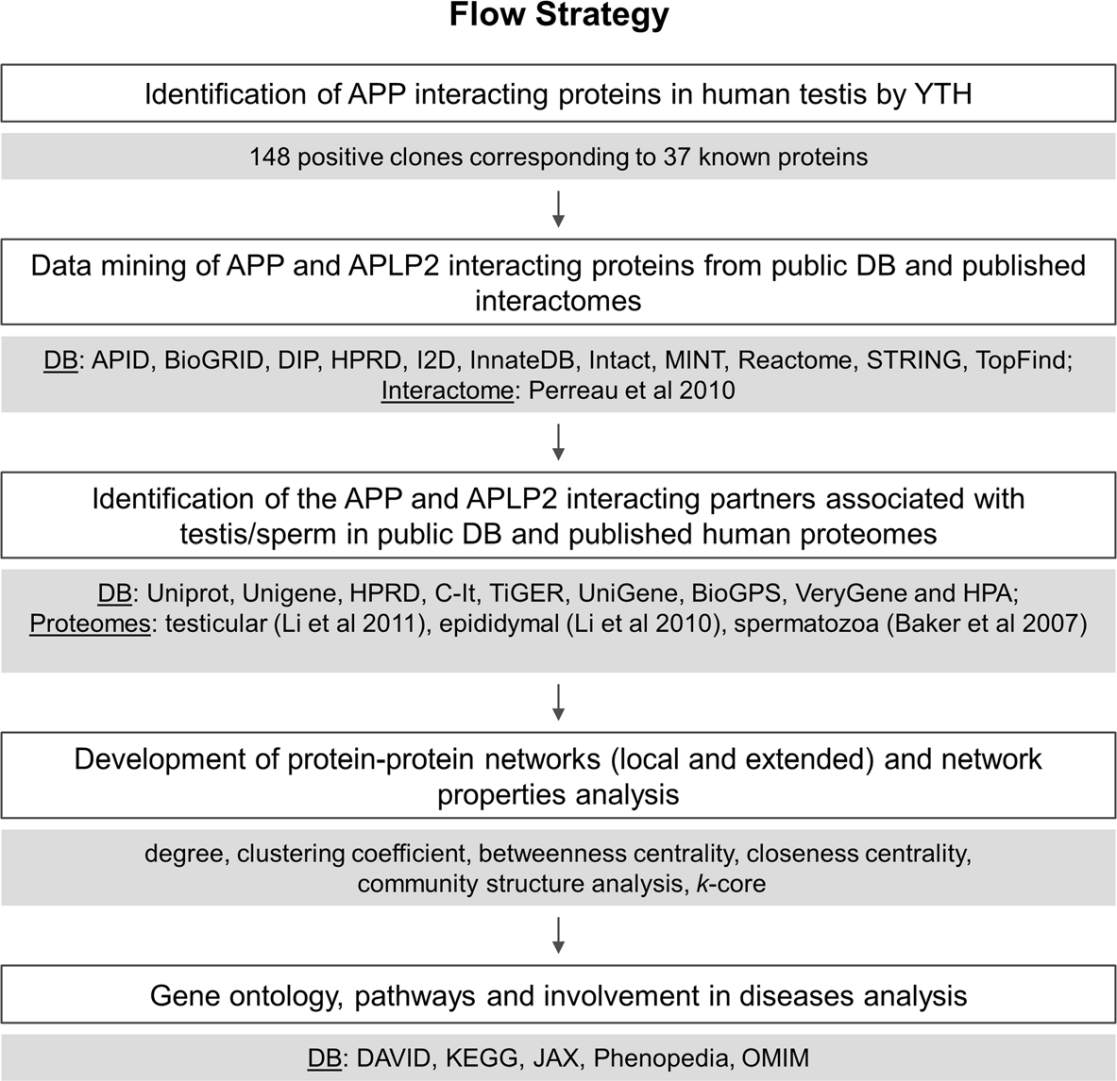
**Diagram for the number of proteins interacting with APP from each dataset.** The numbers indicate testis/sperm-annotated proteins interacting with APP from the different data sources; the numbers within parentheses represent the proteins enriched in testis and sperm. Self-connection for APP was neglected.

In order to identify APP interacting proteins expressed in human testis, an YTH screen of a human testis cDNA library was carried out using full-length human APP. The screen yielded 147 positive clones from a total of 3×10^8^ clones screened. After partial or complete sequence analysis (depending on the length of the positive clone’s cDNAs), in silico searches of the GenBank DB allowed their identification and classification into three separate groups. Table 1 corresponds to library inserts encoding known proteins identified as putative APP interactors. The second and third groups correspond to clones putatively encoding novel APP interacting proteins with homology to genomic sequences and lists positives where the GenBank sequence similarity did not correspond to an annotated gene and false positive hits, respectively. Table 1 lists only 1 positive encoding a previously identified APP interacting protein (RANBP9) (Figure 1). 77 clones encoded 36 known proteins that were not previously associated with APP (Figure 1). Only the clones in Table 1 were included in the network and further functional analyses (Figure 2).

**Table 1:** **Human testis cDNA library inserts encoding known proteins identified as putative APP interactors**

	**GenBank acession**	**Uniprot ID**	**Gene symbol**	**Protein name**	**Chr**	**No. of clones**
1	NM_022735	Q9H3P7	ACBD3	Golgi esidente protein GCP60	1	1
2	NM_007247	Q9UMZ2	SYNRG	Synergin gamma	17	2
3	NM_007348	P18850	ATF6	Cyclic AMP-dependent transcription factor ATF-6 alpha	1	5
4	NM_018844	Q9UHQ4	BCAP29	B-cell receptor-associated protein 29	7	1
5	BC002461	Q12982	BNIP2	BCL2/adenovirus E1B 19 kDa protein-interacting protein 2	15	1
6	NM_020531	Q9HDC9	APMAP	Adipocyte plasma membrane-associated protein	20	2
7	NM_001745	P49069	CAMLG	Calcium signal-modulating cyclophilin ligand	5	4
8	NM_019052	Q8TD31	CCHCR1	Coiled-coil alpha-helical rod protein 1	6	1
9	NM_004356	P60033	CD81	CD81 antigen	11	5
10	NM_002414	P14209	CD99	CD99 antigen	X/Y	7
11	NM_000747	P11230	CHRNB1	Acetylcholine receptor subunit beta	17	2
12	NM_030782	Q96KA5	CLPTM1L	Cleft lip and palate transmembrane protein 1-like protein	5	1
13	NM_006837	Q92905	COPS5	COP9 signalosome complex subunit 5	8	3
14	NM_006368	O43889	CREB3	Cyclic AMP-responsive element-binding protein 3	9	1
15	NM_021227.3	Q9NRP0	OSTC	Oligosaccharyltransferase complex subunit OSTC	4	3
16	NM_004413	P16444	DPEP1	Dipeptidase 1	16	5
17	NM_024293	Q8NC44	FAM134A	Protein FAM134A	2	1
18	NM_020937	Q8IYD8	FANCM	Fanconi anemia group M protein	14	2
19	NM_000146	P02792	FTL	Ferritin light chain	19	1
20	NM_002510	Q14956	GPNMB	Transmembrane glycoprotein NMB	7	1
21	NM_002213	P18084	ITGB5	Integrin beta-5	3	1
22	NM_018559	Q8IXQ4	KIAA1704	Uncharacterized protein KIAA1704	13	2
23	NM_024874	Q8IZA0	KIAA0319L	KIAA0319-like, transcript variant 1		1
24	NM_014400	O95274	LYPD3	Ly6/PLAUR domain-containing protein 3	19	1
25	NM_005493	Q96S59	RANBP9	Ran-binding protein 9	6	4
26	NM_002951	P04844	RPN2	Dolichyl-diphosphooligosaccharide--protein glycosyltransferase subunit 2	20	1
27	NM_005086	Q14714	SSPN	Sarcospan	12	3
28	NM_004206	Q9BRL7	SEC22C	Vesicle-trafficking protein SEC22c	3	9
29	NM_003164	Q13190	STX5	Syntaxin-5	11	1
30	NM_003487	Q92804	TAF15	TATA-binding protein-associated factor 2N	17	1
31	NM_016495	Q9P0N9	TBC1D7	TBC1 domain family member 7	6	1
32	NM_182559	Q86WS5	TMPRSS12	Transmembrane protease serine 12	12	1
33	NM_003270	O43657	TSPAN6	Tetraspanin-6	X	3
34	NP_001001790	Q8N4H5	TOMM5	Mitochondrial import receptor subunit TOM5 homolog	9	1
35	NM_001242313.1	P0C7N4	TMEM191B	Transmembrane protein 191B		1
36	NM_001207052.1	A6NGB0	TMEM191C	Transmembrane protein 191C		
37	NR_003377	A8MUH7	PDZK1P1	Putative PDZ domain-containing protein 1P	1	1

Analysis of the YTH screen revealed that the most abundant interaction was detected with SEC22C (9 out of the 147 positive clones) (Figure 1). This protein is involved in vesicle transport between the ER and the Golgi complex.

The 37 proteins identified as APP interactors were classified into broad functional categories according to Gene Ontology annotation using the DAVID bioinformatics resource (Additional file 1: Table S1). Regarding the biological process, the categories with the largest number of proteins were related to intracellular transport (20.6%) and protein localization (20.6%). From the proteins involved in transport, 5 were linked with vesicle-mediated transport (SYNRG, BCAP29, SEC22C, FTL and STX5). Also, 5 proteins (CD99, LYPD3, GPNMB, ITGB5 and SSPN) were associated with cell adhesion. CD81, CREB3 and FANCM were annotated as being involved in reproduction. The majority of APP interactors identified in the YTH (67.6%) are intrinsic to membrane and 7 are specifically at the plasma membrane (Additional file 1: Table S1).

Analysis of human proteomes (testis, epididymis, and spermatozoa) allowed the classification of DPEP1 and TMPRSS12 as testicular proteins; ITGB5 and COPS5 as sperm-located testicular proteins also detected in epididymal fluid; and FTL as a non-sperm located epididymal fluid protein (Additional file 2: Table S2). CD81, CD99, COPS5 and FAM134 were identified as testis/sperm-enriched in tissue-expression DBs [15-18]. Also, TMPRSS12 was reported in the Unigene as a testicular/spermatozoa restricted protein.

To determine which proteins are known to be important for normal male reproductive function, the dataset was screened against the Jackson Laboratory mutant mouse DB [19] and Phenopedia [20]. From the APP interactors identified in the YTH screen, 3 were connected with reproductive phenotypes in gene knockout models (RANBP9, CREB3 and FANCM). From the comparison with the disease genes listed in Phenopedia no results were obtained.

### Identification of literature curated interactions

In order to identify the potentially relevant interactors of APP and APLP2 to male fertility, human PPI were collected from currently available public DBs, including APID [21], BioGRID [22], DIP [23], HPRD [24], InnateDB [25], Intact [26], MINT [27], Reactome [28], TopFind [29], and STRING [30]. Only the interactions between both proteins associated with the terms “testis” and “sperm” in Unigene, HPRD [24] and Uniprot [31] were selected. Then, the interactors characterized as highly specific to or strongly expressed in testis/sperm were identified from tissue-expression DBs (C-It [15], TiGER [16], UniGene, BioGPS [32], VeryGene [17] and HPA [33]). (See Methods and Additional file 3: Table S3). Besides the DBs, the tissue expression data was also retrieved from the published proteomes of reproductive tissues [34-38]. This analysis allowed the classification of the APP direct interactors into distinct but overlapped localizations (Additional file 2: Table S2).

**Figure 3:**
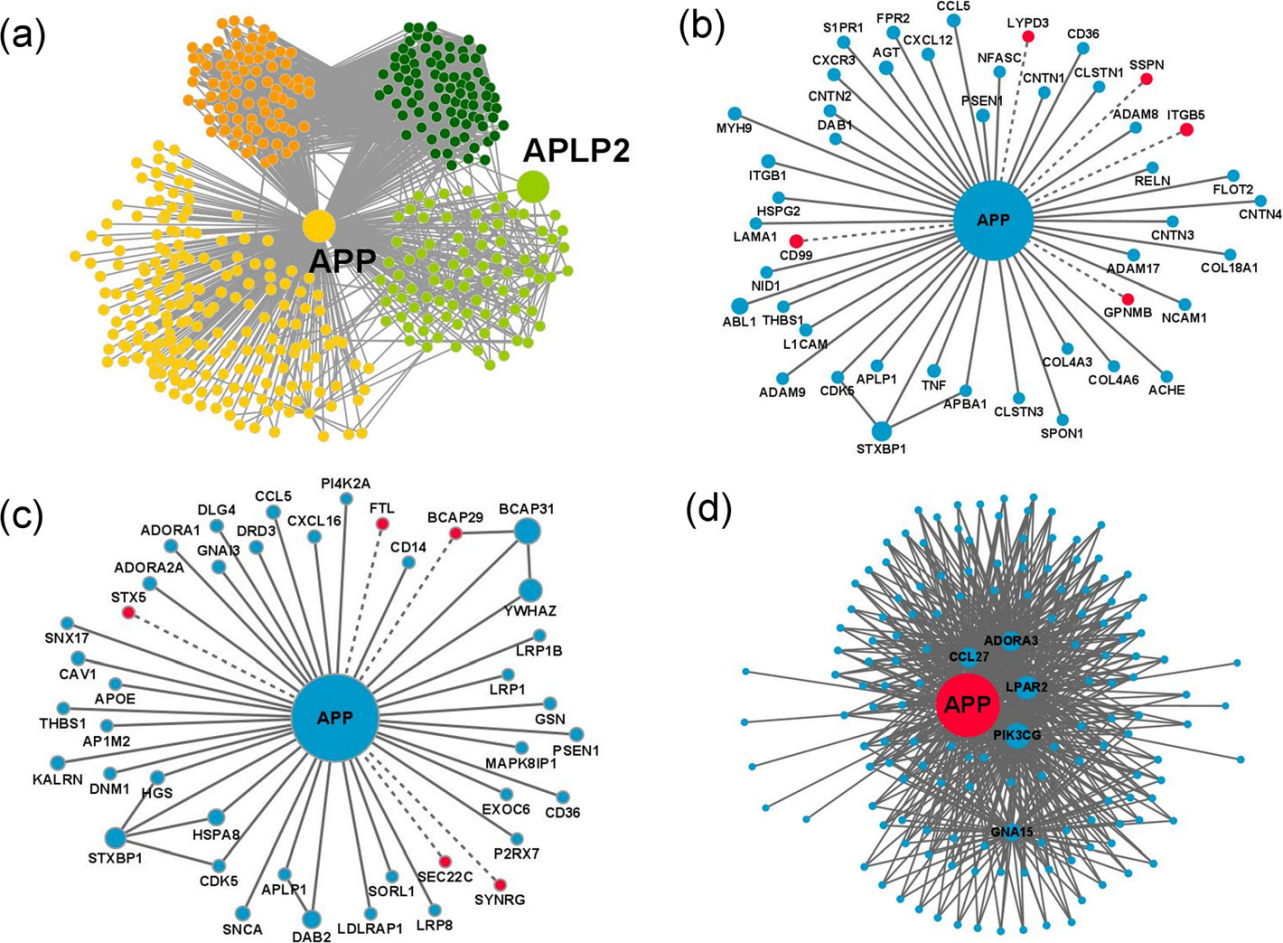
**APP protein-protein interaction networks. (a)** Local APP/APLP2 network. Proteins with light orange (M1) and dark orange (M4) are mostly involved in G-protein coupled receptor protein signaling pathway (Biological Process; BP) and located at the plasma membrane (Cellular Component; CC). Proteins with light green (M2) and dark green (M3) are mostly involved in regulation of apoptosis (BP) and located at the cell surface (CC). **(b)** Subnetwork of APP interactors involved in cell adhesion (BP) extracted from the extended APP/APLP2 protein-protein interaction network. **(c)** Subnetwork of APP interactors involved in vesicle-mediated transport (BP) extracted from the extended network. Red colored nodes represent the proteins from the YTH screen. Blue colored nodes indicate interactors extracted from the DBs. Node size represents relative degree of the nodes and the other interactions of nodes are neglected for the sake of simplicity. Dashed lines represent the interactions revealed by the YTH screening in human testis and solid lines are interactions from DBs. **(d)** Subnetwork of APP interactors involved in G-protein-coupled singnal pathways.

First, we focused on local interactions of APP/APLP2, that is, the first direct interactors of APP/APLP2 and interactions between them. We identified 455 proteins connected to APP (Methods) including the partners identified by YTH (Figure 3a). All the proteins in the YTH data were newly found as interactors of APP except RANBP9, which was previously published as an APP interactor [9]. The absence of protein overlapping may be due to the fact that the YTH was performed using a library from human testis and the previous APP interactors were mainly identified in neuronal tissues. Indeed, published data indicate that 4% of the mammalian genome (more than 2,300 genes) encodes genes specifically expressed in the male germ line during or after the completion of spermatogenesis [39]. Regarding APLP2, we identified 6 proteins (including APP) as its interactors from the DBs which were highly specific to or strongly expressed in testis. In total, 1,803 interactions were identified between 457 proteins including APP and APLP2. Only one protein (BRCA1) among the nearest neighbors of APLP2 was not directly connected to APP which may reflect an isoform-specific role for APLP2.

Second, we extended the local interaction network of APP/APLP2 into the second nearest neighborhoods since the local network of APP could limit an overview of the pathways in which this protein may be involved in testis and spermatozoa. In this network, we had 2,733 proteins and 17,188 interactions between them (Methods).

### Topological analysis of APP/APLP2 PPI network

The overall structural properties for the local and extended APP/APLP2 network showed mostly linear relationship between degree [the number of nearest neighbors (connectivity) of a certain node] and betweenness centrality [fraction of shortest paths between all other nodes that pass through a certain node (Additional file 4: Table S4)]. In our APP/APLP2 local network, proteins with high connectivity also revealed high centrality which can be a significant indication of the relevant proteins in a biological network.

#### Local APP/APLP2 interaction network

In the testis/sperm related APP/APLP2 network (Figure 3a), most proteins were densely connected to each other. Average degree of this network was < q > = 3.95 and global clustering coefficient was C = 0.51. The clustering coefficient reflects how neighbors of a node are connected to each other (Additional file 4: Table S4).

It is known that proteins with high connectivity (hubs) in PPI networks potentially have functional importance in biological systems and are likely to be critical proteins [40]. The key proteins for disease are known to have low clustering coefficients in addition to high connectivities [41]. In order to characterize the APP network topology, the clustering coefficients of each protein were calculated. Betweenness centrality and closeness centrality of each protein in the APP/APLP2 network were also measured to find the relevant proteins involved in pathways (Additional file 4: Table S4). In biological networks, e.g. signaling pathways and genetic interactions, the dysfunction of the proteins with high centrality may be crucial for the other biological functions due to missing of signal transference. In yeast, the proteins with high betweenness centrality, but small number of degrees were found to be important links between well connected modules [42]. Proteins with high centrality rank in our network were represented in Additional file 5: Table S4 (Supporting Text and Additional file 5: Table S4). The top rated interactors included a calcium/phospholipid-binding protein which promotes membrane fusion and is involved in exocytosis (ANXA1, annexin A1). PIK3CG (phosphatidylinositol 4,5-bisphosphate 3-kinase catalytic subunit gamma isoform), PLCB3 (1-phosphatidylinositol 4,5-bisphosphate phosphodiesterase beta-3), LPAR2 (lysophosphatidic acid receptor 2), RLN3 (relaxin-3 receptor 2) and ADORA3 (protein ADORA, isoform 3) also composed the top rated proteins and were all related with the G-protein coupled receptor signaling pathway.

The proteins identified as APP/APLP2 interactors were classified into functional categories according to Gene Ontology annotation using the DAVID program [43]. Regarding the biological process, the results revealed that the categories with the largest number of proteins were related to cell surface receptor linked signal transduction (43.0%; p value = 7.0E-60) and G-protein coupled receptor protein signaling pathway (33.3%; p value = 7.5E-58). 50.8% of the proteins were located at the plasma membrane (p value = 2.4E-26) and 23.2% in the extracellular region (p value = 1.7E-6). 13.0% were associated with vesicles (p value = 1.8E-11). These vesicle related proteins may participate in specialized vesicle activity in the testis, such as acrosome formation. Additionally, 13.9% are annotated as part of a cell projection (e.g. a flagellum) (p value = 1.0E-12).

Metabolic pathways were analyzed using the KEGG PATHWAY [44], which indicated that the top 4 significant categories were: Neuroactive ligand-receptor interaction (16.3%; p value = 2.5E-34); Chemokine signaling pathway (10.4%; p value = 1.3E-18); Calcium signaling pathway (8.6%; p value = 1.8E-13); and Progesterone-mediated oocyte maturation (5.5%; p value = 2.2E-11).

In order to find the core and peripheral part of the local APP/APLP2 network, k-core analysis was performed. k-core is a subgraph of a graph in which all vertices have at least k-degree (Additional file 4: Table S4). The core of this network has a connectivity, k = 11, between them. 75.0% of the proteins within this core shared the same biological process GO category (G-protein coupled receptor protein signaling pathway; p value = 6.6E-15) and 50.0% share the same subcellular localization (plasma membrane; p value = 6.9E-4).

Four modules were identified by community detection analysis (Additional file 4: Table S4). The nodes in a community are more tightly connected to each other than to nodes out of the community and may perform a common function. Our analysis shows that, in the local APP/APLP2 network, 206 proteins are included in module 1 (light orange in Figure 3a). Among them, 19.7% were involved in the regulation of apoptosis (p value = 1.5E-12) and the most significant represented cellular localization was the cell surface (16.3%, p value = 2.3E-17. The most prominent biological process detected in module 2 (which comprised 84 proteins in total, dark green in Figure 3a) was G-protein coupled receptor protein signaling pathway (76.2%; p value = 6.6E-52) and 71.4% of the proteins in module 2 shared the same localization (plasma membrane; p value = 5.6E-15). Module 3 included 85 proteins (light green in the Figure 3a), which were mainly involved in G-protein coupled receptor protein signaling pathway (94.0%; p value = 5.0E-80) and localized at the plasma membrane (76.2%, p value = 2.5E-18). Similarly to module 1, the most significant category in module 4 (which comprised 82 proteins in total, dark orange in Figure 3a) was regulation of apoptosis (39.0%, p value = 4.8E-19). Modules 2 and 3 also share a common biological function and cellular component. In the local APP/APLP2 network, the core part of the network, that is, the proteins with the highest k-core (k = 11) which includes APP, shared the same biological function (G-protein coupled receptor protein signaling pathway). The core part was included mostly in module 2, which was also associated with the same function. Therefore, this result showed that APP might be involved in G-protein coupled receptor protein signaling pathway in human testis/sperm. In addition, APP has high possibility that it is associated with regulation of apoptosis according to the results that the most interaction partners surrounding APP share the same function within modular structure.

#### Extended APP/APLP2 network

The local APP network only allows us to study relationships between APP and its nearest neighboring proteins. In order to study relationships with other proteins, we extended the network to the second nearest neighbors of APP. Topological properties of the extended networks are analyzed in Additional file 6: Table S5. From *k*-core analysis, a densely connected group with high k (=17)-core was found (186 proteins). The proteins in the core were involved in cell cycle (41.1%; p value = 6.4E-46). Also, the majority of proteins were found in the cytosol (49.2%; p value = 1.0E-41) and the nucleoplasm (40.0%, p value = 2.0E-38).

Based on the community structure analysis, APP is located in module 2. The most significant biological processes associated with this module were proteolysis (19.3%; p value = 6.6E-9) and cell adhesion (15.9%; p value = 1.3E-9). Additionally, the majority of proteins were located at the plasma membrane (42.0%; p value = 1.3E-6) and at the extracellular region (32.4%; p value = 5.4E-12).

Table 2 represents the gene ontology analysis for the modules of the extended network in which at least 40% of proteins shared a biological function.

**Table 2:** **Enriched GO categories for each module of the extended network**

**M**	**Most significant biological process**			**Most significant cellular component**		
	**GO term**	**p value**	**%**	**GO term**	**p value**	**%**
4	Cell cycle	2.7E-82	60.2	Cytosol	4.3E-41	48.8
	Regulation of ubiquitin-protein ligase activity during mitotic cell cycle	1.3E-130	41.0	Proteasome complex	1.9E-67	25.3
5	DNA metabolic process	1.2E-88	45.0	Nuclear lumen	2.1E-56	48.6
				Nucleoplasm	8.7E-66	44.1
7	Microtubule-based process	3.8E-40	40.0	Microtubule cytoskeleton	6.1E-68	70.8
				Centrosome	3.8E-70	58.4
10	Cell cycle	6.6E-20	47.2	Chromosomal part	1.2E-19	39.6
				Chromosome, centromeric region	7.9E-26	35.8
11	G-protein coupled receptor protein signaling pathway	9.5E-141	63.1	Plasma membrane	4.3E-45	65.1
12	Modification-dependent protein catabolic process	1.7E-16	40.4	Endoplasmic reticulum	4.2E-9	32.7

Only modules with at least 40.0% of the proteins sharing a biological function are represented. M, modularity.

#### Specific topological features of proteins from YTH

Based on the extended network structure analyses, COPS5 has a large number of connections (q = 152) and also a relatively high betweenness centrality (b = 0.046) among our 37 YTH proteins, contrasting with a low clustering coefficient (C = 0.014). COPS5 is sperm-located testicular protein [36]. CD81 (q = 27, b = 0.005, C = 0.029), CD99 (q = 13, b = 0.001, C = 0.064) and IGTB5 (q = 10, b = 0.0003, C = 0.089) also revealed prominent topological properties (Additional file 6: Table S5).

### Topological role of APP and APLP2 in the network

Previous data has shown that the absence of both APP and APLP2 lead to the abnormal developments of sexual organs, the reduction of synaptic vesicles, and even postnatal lethality in mice [6]. On the other hand, the absence of either APP or APLP2 does not affect viability and fertility. Based on these, one can imagine that these two proteins should co-exist for the mammalian life maintenance. Here, we focus on the role of APP and APLP2 for the human male fertility. Based on experimental results of gene knock-outs in mice [6], one can assume that APP and APLP2 are simultaneously involved in important pathways. Some proteins or protein complexes cannot accomplish biological functions in the absence of APP/APLP2, because this blocks the functional routes between the proteins. In order to find a conformity of the functional property within a structural property, we checked the local triangle structure between APP, APLP2, and the common interactors (CDK1, DAB2, JUN, and PIK3CA). Among the interactors of APLP2, only BRCA1 is not connected to APP. These common interactors of APP and APLP2 form a small modular structure (Additional file 7: Figure S1). Therefore, one can guess the proteins in this module possibly share a biological function in testis.

## Discussion

Biomolecular networks are now frameworks that facilitate many discoveries in molecular biology. The theoretical advances in network science in parallel with high throughput efforts to map biological networks, offer an excellent opportunity to apply the principles of theoretical physics to the molecular biomedicine field.

The APP network in testis/sperm was built first using an YTH screen and then expanded by incorporating literature curated interactions. Since protein profiles of the different tissues are critical to understand the unique characteristics of the various human cell types, in this study, we took into account the tissue expression of the interactors in the network. From the YTH screen, we reported the identification of 36 novel APP interacting proteins in human testis/sperm. Only 1 positive encoded a previously identified APP interacting protein (RANBP9). This may be explained by the fact of testis being a very peculiar organ which possesses specific patterns of transcription and expresses novel protein isoforms [39,40]. APLP2 interacting partners were also included in this study.

To determine which PPI in our APP/APLP2 network were biologically more relevant for male reproduction, we performed network structure analyses and bioinformatic analyses. Based on the community/modularity analysis of the PPI network along with gene ontology analysis, we confirmed that proteins involved in similar functions are group together and form modules. The biological process GO category more significantly represented in the APP/APLP2 local network in human testis/sperm was cell surface receptor linked signal transduction with 43.0% of the proteins annotated in this class. These proteins may indicate how the male germ cells interact with the outside world. Among those proteins, 33.3% carry the GO functional tag for G-protein coupled receptor protein signaling pathway. Some studies indicate that full length APP can function as a cell surface GPCR and show that APP binds heterotrimeric G proteins (Gαo) [45,46]. Recently, Deyts and colleagues discovered an interaction between APP intracellular domain and the heterotrimeric G-protein subunit Gαs [47]. G protein-coupled receptors signalling pathways have been proposed to control several processes essential for sperm function and fertilization, namely in sperm capacitation and acrosome reaction [48-51]. APLP2/YWK-II also exhibits properties of a receptor and its extracellular domain was shown to interact with Müllerian-inhibiting substance [50]. Müllerian-inhibiting substance increases the viability and longevity of human spermatozoa through binding the APLP2/YWK-II component on the sperm membrane [51]. Huang and colleagues showed that APLP2/YWK-II component binds to a GTP-binding protein (Gαo).

The most abundant interaction detected in the YTH was with SEC22C. This protein is involved in vesicle transport between the ER and the Golgi complex [52]. Vesicular membrane trafficking is an essential process during acrosome biogenesis [53]. Also, SEC22C may control the APP traffic through the secretory pathway. Besides SEC22C, other four YTH clones (BCAP29, FTL, STX5, and SYNRG) are involved in vesicle-mediated transport (Figure 3c). This GO term includes the regulation of the acrosomal vesicle exocytosis, an essential process for fertilization, which begins with the fusion of the outer acrosomal membrane with the sperm plasma membrane and ends with the exocytosis of the acrosomal contents into the oocyte.

The cellular component category most enriched in the GO term analysis of the APP/APLP local network was the plasma membrane. Fertilization is achieved through gamete interactions, specifically cell adhesion and then membrane fusion of the gamete plasma membranes. The occurrence of 50.8% of proteins in the plasma membrane may suggest their involvement in sperm-egg interaction. Additionally, 10.2% of APP interactors are involved in cell adhesion (Figure 3b). Of these, CD99, GPNMB, ITGB5, LYPD3, and SSPN were identified in the YTH screen performed using a testis library. The APP yeast mating efficiency in the YTH was much higher than usual (50%, when compared to a normal 5%), which may be related to APP cell adhesion properties. This strengthens previous results suggesting APP to be involved in cell-to-cell contact, a very important process in gamete fusion. Recent approaches to identify candidate proteins involved in sperm-egg interaction have been characterizing the sperm proteome and analyzing specific subpopulations of interest, for instance, glycoslylated proteins and integrins. Additionally, proteins with motifs or belonging to families of interest like transmembrane domains and the tetrasparin family should also be considered. Interestingly, some of the YTH positive clones are included in those categories. APP interacts with ITGB5, identified in the YTH screen, and ITGB1 [54], both belonging to the integrin beta chain family. Integrins on eggs became of interest with the discovery of an integrin ligand-like domain in ADAM2, a sperm antigen essential for sperm-egg interaction.TSPAN6 and CD81 belong to the tetraspanin family. The discovery that the knockout of CD9, a member of the tetraspanin family, in mouse leads to healthy, but subfertile females due to defective sperm-egg interaction revolutionized the fertility field. CD81 is 45% identical to CD9 and Cd81 knockout mouse also presents defects in female fertility. Cd9−/−/Cd81−/− female mice are completely infertile. We found that, in local network, APP, TSPAN6, ITGB1, ITGB5, GPNMB, LYPD3, SSPN, CD81 and CD99 were in the same module (module 1). However, in extended network, APP, TSPAN6, GPNMB, LYPD3, SSPN, and ITGB5 were in module 2, whereas ITGB1, CD81, CD9 and CD99 were well connected in module 9 in which 20.3% of the proteins share the biological function – cell adhesion (Figure 3b).

We also identified tissue-specific APP interacting proteins which can lead to the identification of pathways for the APP family members associated with testis and sperm functions. TMPRSS12, a transmembrane serine protease, was identified in the YTH screen and was reported in the Unigene as testicular/spermatozoa restricted. TMPRSS12 belongs to the same module from network community as APP. Since this protein is connected to APP only, it cannot have any route to the main network without APP. Sperm-surface proteases were already shown to be required for fertilization [55]. There is also evidence for the participation of serine proteolytic activities during spermatogenesis and sperm maturation [56]. However, most of the specific proteases that are involved in these processes are unknown. The exact localization of TMPRSS12 at sperm membrane has to be determined.

## Conclusions

The present work provided the first report on APP interactome in human testis. We identified several novel APP interactions in human testis and incorporated YTH data and PPI databases to construct the PPI network of APP in human testis and spermatozoa. The protein interaction network allowed the recognition of proteins complexes and modules crucial for several biological functions, such as cell adhesion.

## Methods

### Human testis library screening by Yeast Two-Hybrid

The APP cDNA was directionally subcloned into EcoRI/BamHI digested pAS2-1 (GAL4 binding domain expression vector) to produce pAS-APP. This expression vector was first used to confirm the expression of the resulting fusion proteins (GAL4-APP) in yeast strain AH109. For library screening, the yeast strain AH109 transformed with pAS-APP, was mated with yeast strain Y187 expressing the human testis cDNA library in the pACT-2 vector (Gal4 activation domain expression vector). Half the mating mixture was plated onto high stringency medium (quadruple dropout medium (QDO): SD/-Ade/-His/-Leu/-Trp) and the other half onto low stringency medium (triple dropout medium (TDO): SD/-His/- Leu/-Trp), and the plates were incubated at 30°C. Colonies obtained in the low stringency plates were replica plated onto high stringency medium. Finally, all high stringency surviving colonies were plated onto selective medium containing X-α-Gal and incubated at 30°C to check for MEL-1 expression (indicated by the appearance of a blue colour) [10]. All the YTH reagents were purchased from Enzifarma, Clontech, Portugal. All other nonspecified reagents were purchased from Sigma-Aldrich, Portugal. This study did not required ethics approval since the material used was purchased for Enzifarma, Clontech, Portugal (human testis cDNA library which contained cDNAs already inserted in pACT-2 vector.

### Recovery of plasmids from yeast and sequence analysis

Yeast plasmid DNA was recovered and used to transform E. coli XL1-Blue. Plasmid DNA was obtained from each resulting bacterial colony and digested with the restriction enzyme HindIII (NEB, Ipswich, USA) to identify the corresponding library plasmids. DNA sequence analysis was performed using an Automated DNA Sequencer (Applied Biosystems, Carlsbad, USA) using the GAL4-AD primer - TACCACTACAATGGATG (Enzifarma, Clontech, Portugal). The DNA sequences obtained were compared to the GenBank DB, using the BLAST algorithm, to identify the corresponding encoded proteins.

### Data mining of APP and APLP2 interacting proteins from public DB and published interactome

Several data sources were used to human protein-protein interaction data retrieval. First, we collected all interaction data of human proteins from: APID [17], BioGRID [18], DIP [19], HPRD [20], InnateDB [21], Intact [22], MINT [23], Reactome [24], TopFind [25], and STRING [26]. The interaction search was restricted to Homo sapiens (Human, 9606) protein pairs. Then, only the interactions defined as “association (MI:0941)” under the interaction type categories (http://www.ebi.ac.uk/ontology-lookup/) and “experimental interaction detection (MI:0045)” from STRING were extracted (See Additional file 3: Table S3). Next, we unified protein names based on the Uniprot ID and gene symbols in order to prevent abundant interactions caused by the different notations for the same gene between DBs. We removed proteins which have unreviewed, no gene symbols or Uniprot IDs, and removed (obsolete) genes from the Uniprot database up to the date of our data-mining and their interactors from our PPI list. We also included the interacting proteins from the published APP interactome [9] and the YTH experiment (37 proteins). Finally, 248,714 interactions between 15,189 proteins were obtained in total.

### Identification of testis/sperm specific proteins in public DBs and publish human proteomes

From the large PPI data obtained in the previous data-mining, we narrowed down the candidate proteins into the testis/sperm enriched proteins. We first used three distinct data sources to select the proteins associated with the testis: UniProt [53], UniGene and the Human Protein Reference Database (HPRD) [20]. From UniGene expressed sequence tags (ESTs) from Homo sapiens were used as source of gene expression data. Among our previous interaction data set, we chose all proteins associated with the keywords “testis” and/or “sperm” in the description of tissue-specificity. Then, we kept the interactions, if both proteins in a pair of interaction were associated to testis/sperm. We got totally 155,457 interactions among 12,884 proteins in this procedure.

Second, tissue-expression DBs (C-It, TiGER, UniGene, BioGPS, VeryGene and HPA) were used in order to identify the interactors characterized as highly specific or strongly expressed in testis/sperm. The C-it DB was queried with the keywords, 'testis-enriched' for 'Human'. The limitation factors for the literature information were 5 for PubMed and 3 for MeSH terms. Proteins with a SymAtals z-score higher than |1.96| were chosen. The TiGER and the Very Gene database were also searched for 'Testis' in 'Tissue View' category. In HPA (Human Protein Atlas), proteins listed within the fields of high or medium HPA evidence and annotated protein expression based on IHC staining patterns in normal male reproductive tissues were selected. Also, the BioGPS was used to find testis/sperm restricted proteins with the keywords, 'testis, sperm, epididymis, spermatid, spermatogonia, spermatozoa, spermatocyte' in 'Human'. Using the plugin 'Gene expression/activity chart', the proteins with highly/strongly expressed in testis were selected. If the expression level was less than mean value or the data were not shown, those proteins were removed from the list. Also the proteins with high correlation level of expression (≥0.9) with testis-specific proteins, such as ACRV1, AKAP4, BRDT, PGK2, TSGA10, and TSPY8 were selected. From this search, 1,949 testis/sperm enriched proteins were obtained.

### Development of protein-protein network and network properties analysis

From testis/sperm enriched proteins, APP/APLP2 interactors were selected. Functional relationships can be neglected when considering only tissue-enriched/specific proteins. This challenge was addressed by integrating tissue-enriched/specific APP/APLP2 interactors with its interacting proteins regardless whether they were enriched or not. Using the breath-first searching (BFS) algorithm, direct connectors with APP and the interactions between those proteins were kept. The same procedure was applied to APLP2. Since APLP2 is the nearest neighbor of APP, we combined two sub-networks and analyzed several network properties. We extended this local network to the second order neighbors of APP in order to see the wide relations around APP and APLP2. All YTH data were included in this process. Finally, 1,803 interactions between 457 proteins were obtained for the local APP/APLP2 network and 17,188 interactions between 2,733 proteins for the extended APP/APLP2 network.

### Bioinformatic analyses: gene ontology, pathways and involvement in diseases

The interactome was analyzed using the Database for Annotation, Visualization and Integrated Discovery (DAVID) v6.7 [53]. The UniProt [53] identifiers (UniProt_ID) for the proteins were entered into the DAVID functional annotation program. Overall, the proteins were analyzed for gene ontologies and pathways using the Homo sapiens genome-wide genes with at least one annotation in the analyzing categories as background. Proteins associated with defects in male fertility, or a functional or morphological defect in the epididymis, testis, or sperm were obtained from the Jackson Laboratories mouse knockout database (http://www.informatics.jax.org/). To obtain a list of the genes that have a male infertility phenotype, we also queried the OMIM [55], Phenopedia [16] and the Uniprot database [53] with the term “male infertility”, and then downloaded the associated genes.

### Availability of supporting data

The data sets supporting the results of this article are included within the article and its additional files.

## Additional files

Additional file 1: Table S1 Enriched GO categories of the APP interactors identified by YTH. Enriched categories are identified as those with p<0.05. Additional file 2: Table S2 Distribution of APP interacting proteins in human testicular, epididymal and sperm proteomes, and their overlap. The human epididymis proteome includes both epididymal tissue and fluid proteomes. The secretory vesicular (epididymosome) part of the human epididymosome proteome was also considered. In bold are the APP interactors identified in the YTH screen. Additional file 3: Table S3 Statistics of collected protein-protein interaction data. Additional file 4
**Topological properties of network.**
Additional file 5: Table S4 (a). Rank of topological properties of 457 proteins in local APP/APLP2 network. Additional file 6: Table S5 (a). Top 1000 rank of topological properties of proteins in extended APP/APLP2 network. Note that proteins with clustering coefficient 0 are neglected. Additional file 7: Figure S1 Modular structures of poteins sharing APP-APLP2 interaction. Four common proteins interacting with APP and APLP2 form triangle or square modules (indicated by green links) which shows that they can be highly possible functional modules. Blue nodes are testis-specific and orange node (APLP2) is not.
