# Supplementary material for: Amyloid precursor protein interaction network in human testis: sentinel proteins for male reproduction
Source: BMC Bioinformatics. 2015 Jan 16;16(1):12. doi: 10.1186/s12859-014-0432-9 (PMC4384327; doi:10.1186/s12859-014-0432-9)
Supplement: Additional file 1: Table S1 — Enriched GO categories of the APP interactors identified by YTH. Enriched categories are identified as those with p<0.05. [file 12859_2014_432_MOESM1_ESM.docx]

| **GO designator** | **GO Term** | **No. of proteins** | **%** | **P-Value** | **Proteins (Uniprot Acession)** |
| --- | --- | --- | --- | --- | --- |
| **Biological Process** | | | | | |
| GO:0046907 | intracellular transport | 7 | 20.6 | 1,90E-03 | STX5, SYNRG, TOMM5, SEC22C, CCHCR1, FTL, BCAP29 |
| GO:0008104 | protein localization | 7 | 20.6 | 8,20E-03 | STX5, CD81, SYNRG, TOMM5, SEC22C, CCHCR1, BCAP29 |
| GO:0015031 | protein transport | 6 | 18 | 1,90E-02 | STX5, SYNRG, TOMM5, SEC22C, CCHCR1, BCAP29 |
| GO:0016192 | vesicle-mediated transport | 5 | 15 | 3,00E-02 | STX5, SYNRG, SEC22C, P02792, BCAP29 |
| **Cellular Component** | | | | | |
| GO:0031224 | intrinsic to membrane | 23 | 67.6 | 1,30E-04 | CD99, STX5, OSTC, CD81, CLPTM1L, TSPAN6, CHRNB1, SSPN, TMEM191B, APMAP, BCAP29, DPEP1, ITGB5, TMPRSS12, FAM134A, LYPD3, TOMM5, CREB3, SEC22C, GPNMB, ATF6, RPN2, CAMLG |
| GO:0005783 | endoplasmic reticulum | 8 | 24 | 3,90E-03 | OSTC, STX5, CREB3, SEC22C, ATF6, CAMLG, RPN2, BCAP29 |
| GO:0008250 | oligosaccharyltransferase complex | 2 | 5.9 | 2,20E-02 | OSTC, RPN2 |
| GO:0012505 | endomembrane system | 6 | 18 | 2,60E-02 | OSTC, STX5, SYNRG, ATF6, BNIP2, RPN2 |
| GO:0031226 | intrinsic to plasma membrane | 7 | 21 | 4,50E-02 | ITGB5, CD99, LYPD3, CD81, GPNMB, CHRNB1, SSPN |
| **Molecular Funtion** | | | | | |
| GO:0004579 | dolichyl-diphosphooligosaccharide-protein glycotransferase activity | 2 | 5.9 | 1,40E-02 | OSTC, RPN2 |
| GO:0004576 | oligosaccharyl transferase activity | 2 | 5.9 | 1,50E-02 | OSTC, RPN2 |
| GO:0003713 | transcription coactivator activity | 3 | 8.8 | 4,20E-02 | CREB3, COPS5, ATF6 |

Table S1. Enriched GO categories of the APP interactors identified by YTH. Enriched categories are identified as those with p<0.05.

GO, Gene Ontology; No, number; %, percentage;
