# Supplementary material for: Amyloid precursor protein interaction network in human testis: sentinel proteins for male reproduction
Source: BMC Bioinformatics. 2015 Jan 16;16(1):12. doi: 10.1186/s12859-014-0432-9 (PMC4384327; doi:10.1186/s12859-014-0432-9)
Supplement: Additional file 2: Table S2 — Distribution of APP interacting proteins in human testicular, epididymal and sperm proteomes, and their overlap. The human epididymis proteome includes both epididymal tissue and fluid proteomes. The secretory vesicular (epididymosome) part of the human epididymosome proteome was also considered. In bold are the APP interactors identified in the YTH screen. [file 12859_2014_432_MOESM2_ESM.docx]

Table S2. Distribution of APP interacting proteins in human testicular, epididymal and sperm proteomes, and their overlap. The human epididymis proteome includes both epididymal tissue and fluid proteomes. The secretory vesicular (epididymosome) part of the human epididymosome proteome was also considered. In bold are the APP interactors identified in the YTH screen.

| **testicular tissue protein** | **non-sperm located sperm milieu epididymal protein** | **sperm-located epididymal protein also detected in sperm milieu** | **sperm-located testicular protein also detected in sperm milieu** | **sperm milieu testicular protein** | **non common between**  **epididymis and testis** | | **common between epididymis and testis** | **common between sperm and testis** | **common between epididymosome and testis** |
| --- | --- | --- | --- | --- | --- | --- | --- | --- | --- |
| **DPEP1** | **FTL** | ADCY1 | **ITGB5** | CRYAB | ADCY5 | PDIA4 | ANXA1 | ADCY8 | APCS |
| **TMPRSS12** | ADCY5 | ADCY8 | **COPS5** | HSD17B10 | ADCY8 | PGAM1 | APCS | APCS | CLU |
| APLP2 | APOA1 | ANXA1 | CLU | HSPA1A | APLP2 | PPID | CALR | C3 | PDIA3 |
| CDK5 | CANX | APCS | CTSD | HSPB1 | APOA1 | TGM2 | CAT | CLU | PPIA |
| DNAH1 | CAT | C3 | HADH | HYOU1 | C3 | TIMP1 | CDK5 | GANAB |  |
| GNB1 | COL1A2 | CALR | HSP90AA1 | PDIA6 | COL1A2 | TTR | CLU | PDIA3 |  |
| GRP | CPE | CAPZA2 | HSP90B1 | PPIB | CST3 | UCHL1 | CRYAB |  |  |
| OAT | CRYAB | CLU | PPIA |  | CTSB |  | CTSD |  |  |
| PDIA4 | CST3 | CTSB |  |  | DNAH1 |  | GAPDH |  |  |
| PPID | GNAQ | CTSD |  |  | GANAB |  | HADH |  |  |
| TUBB | HSD17B10 | GANAB |  |  | GNA15 |  | HSD17B10 |  |  |
|  | HSPA1A | GAPDH |  |  | GNAQ |  | HSP90AA1 |  |  |
|  | HSPB1 | GNA15 |  |  | GNB1 |  | HSP90B1 |  |  |
|  | HSPB6 | GPC1 |  |  | GPC1 |  | HSPA1A |  |  |
|  | HSPD1 | HSP90AA1 |  |  | GRP |  | HSPA4 |  |  |
|  | HYOU1 | HSP90B1 |  |  | HSPA5 |  | HSPB1 |  |  |
|  | NID1 | HSPA4 |  |  | HSPA8 |  | HYOU1 |  |  |
|  | PGAM1 | HSPA5 |  |  | HSPB6 |  | PDIA6 |  |  |
|  | TGM2 | HSPA8 |  |  | HSPD1 |  | PPIA |  |  |
|  | TIMP1 | NMBR |  |  | ITM2B |  | PPIB |  |  |
|  | UCHL1 | NSF |  |  | NMBR |  | TUBB |  |  |
|  | YWHAZ | NTN1 |  |  | NTN1 |  |  |  |  |
|  |  | PDIA3 |  |  | OAT |  |  |  |  |
|  |  | PPIA |  |  | PDIA3 |  |  |  |  |
| **11 proteins** | **22 proteins** | **24 proteins** | **8 proteins** | **7 proteins** | **31 proteins** | | **21 proteins** | **6 proteins** | **4 proteins** |
