# Supplementary material for: Amyloid precursor protein interaction network in human testis: sentinel proteins for male reproduction
Source: BMC Bioinformatics. 2015 Jan 16;16(1):12. doi: 10.1186/s12859-014-0432-9 (PMC4384327; doi:10.1186/s12859-014-0432-9)
Supplement: Additional file 3: Table S3 — Statistics of collected protein-protein interaction data. [file 12859_2014_432_MOESM3_ESM.pdf]

Table S3 Statistics of collected protein-protein interaction data

Database	downloaded date (or released date)	total Interaction	MI:0190 (interaction type)	MI Ontology [3]	Ratio of non-physical Interactions [1]	Final interactions [2]
APID	03.02.2012 (90.02.2009)	123902	77504	MI:0914(77504)		77504
BioGRID	18.04.2012 (29.03.2012)	491567	101925	MI:0403(1068), MI:0407(35443),<br>MI:0794(1), MI:0796(136),<br>MI:0799(136), MI:0914(1641),<br>MI:0915(63491)	1.33 % (1350/101925)	100570
DIP	03.02.2012 (30.12.2009)	18681	16583	MI:0192(2), MI:0194(12),<br>MI:0195(21), MI:0203(193),<br>MI:0204(2), MI:0213(5),<br>MI:0217(60), MI:0218(1470),<br>MI:0220(18), MI:0254(17),<br>MI:0403(83), MI:0407(1314),<br>MI:0408(1), MI:0570(2),<br>MI:0844(1), MI:0914(27),<br>MI:0915(13353), MI:0931(1),<br>MI:0945(1)	0.11 % (18/16583)	12190
HPRD	03.05.2012 (29.06.2010)	39240		no MI information		39018
InnateDB	60.02.2012 (15.07.2011)	14261	12924	MI:0192(2), MI:0194(101),<br>MI:0195(2), MI:0203(25),<br>MI:0204(2), MI:0213(4),<br>MI:0217(566), MI:0220(20),<br>MI:0401(6), MI:0407(4),<br>MI:0408(6), MI:0570(193),<br>MI:0914(119), MI:0915(11855)	0.13 % (17/12924)	8474
IntAct	06.02.2012 (03.01.2012)	290729	57131	MI:0192(13), MI:0194(12),<br>MI:0195(3), MI:0197(16)<br>MI:0203(212), MI:0213(15), MI:0216(4)<br>MI:0217(352), MI:0220(88),<br>MI:0403(966), MI:0407(2082),<br>MI:0414(25), MI:0557(3),<br>MI:0559(1), MI:0566(1),<br>MI:0567(11), MI:0570(35),<br>MI:0844(1), MI:0871(2), MI:0883(1),<br>MI:0902(6), MI:0914(16067),<br>MI:0915(37214), MI:0931(1)	1.69 % (967/57131)	55344
MINT	12.09.2012 (01.08.2012)	33718	23970	MI:0192(18), MI:0194(64),<br>MI:0195(13), MI:0197(1)<br>MI:0203(91), MI:0204(9),<br>MI:0208(95), MI:0210(10),<br>MI:0213(20), MI:0217(269),<br>MI:0220(14), MI:0403(3),<br>MI:0407(2634), MI:0408(25),<br>MI:0414(6), MI:0557(55),<br>MI:0566(3), MI:0567(1), MI:0569(1),<br>MI:0914(155),<br>MI:0915(20467)	0.41 % (98/23970)	23585
Reactome	06.02.2012 (06.02.2012)	116776	116775	MI:0914(47451), MI:0915(69,324)		116775
TopFind	03.02.2012 (?)	4986	4956	MI:0570(4956)		4938
STRING	07.02.2012 (?)	656493	479330	MI:0036(476), MI:0045(52158),<br>MI:0064(52522), MI:0085(2917),<br>MI:0087(396519), MI:0364(125005),<br>MI:0686(26896)		52158

1. Non-physical interaction : Child interaction types of MI:0403(colocallizaiton) and MI:0208(genetic interaction) (blue in MI ontology categories)
2. Final Interactions : The number of interactions after removing non-Homo sapiens interactions, unknown gene names, unreviewed proteins, and other interaction types.
3. The number in parenthesis is proteins with the MI ontology among proteins which both in an interaction are from Homo sapiens.
* Interactions may overlap between databases.

MI:0190 (interaction type)
MI:0192 (acetylation reaction)
MI:0194 (cleavage reaction)
MI:0195 (covalent binding)
MI:0197 (deacetylation reaction)

MI:0197 (deacetylation reaction)
MI:0203 (dyphosphorylation reaction)
MI:0204 (deubiquitination reaction)
MI:0208 (genetic interaction)
MI:0210 (hydroxylation reaction)
MI:0213 (methylation reaction)
MI:0216 (palmitoylation reaction)
MI:0217 (phosphorylation reaction)
MI:0218 (physical interaction)
MI:0220 (ubiquitination reaction)
MI:0254 (genetic interference)
MI:0401 (biochemical)
MI:0403 (colocalization)
MI:0407 (direct interaction)
MI:0408 (disulfide bond)
MI:0414 (enzymatic reaction)
MI:0557 (adp ribosylation reaction)
MI:0559 (glycosylation reaction)
MI:0566 (sumoylation reaction)
MI:0567 (neddylation reaction)
MI:0569 (deneddylation reaction)
MI:0570 (protein cleavage)
MI:0794 (synthetic)
MI:0796 (suppression)
MI:0844 (phosphotransfer reaction)
MI:0871 (demethylation reaction)
MI:0883 (gtpase reaction)
MI:0902 (rna cleavage)
MI:0914 (association)
MI:0915 (physical association)psi-mi:"MI:0036"(domain fusion)
MI:0931 (genetic interaction defined by inequality)
MI:0945 (oxidoreductase activity electron transfer reaction)

STRING
MI:0036 (domain fusion)
MI:0045 (experimental interaction detection)
MI:0064 (interologs mapping)
MI:0085 (phylogenetic profile)
MI:0087 (predictive text mining)
MI:0364 (inferred by curator)
MI:0686 (unspecified method – coexpression)
