# Supplementary material for: Amyloid precursor protein interaction network in human testis: sentinel proteins for male reproduction
Source: BMC Bioinformatics. 2015 Jan 16;16(1):12. doi: 10.1186/s12859-014-0432-9 (PMC4384327; doi:10.1186/s12859-014-0432-9)
Supplement: Additional file 4 — Topological properties of network. [file 12859_2014_432_MOESM4_ESM.docx]

**Supporting Text. Topological properties of network**

1. Clustering coefficient

In a network, the local clustering coefficient characterizes how nearest neighboring nodes of a node are connected to each other. If all of them are tightly connected to each other, then they form a clique and the clustering coefficient is 1. If there is no connection between the nearest neighbors, then the coefficient is zero. The mean clustering coefficient is about 0.66 for the local APP/APLP2 network and about 0.35 for the extended APP/APLP2 network in our paper. In general, biological networks have a large mean clustering coefficient {Wagner, 2001 #161}, therefore, nodes in these networks are strongly interconnected. In our network, the nodes with large degree have a large clustering coefficient. It also confirms that the large degree proteins are tightly interconnected and form local clusters, furthermore, densely interconnected proteins form modules in which proteins share the same biological functions. We also focused on the proteins that have low clustering coefficient and high connectivity, because they can be regarded as important proteins that are involved in signal pathways {Guan, 2008 #160}.

2. Betweenness centrality

Centrality {Freeman, 1977} characterizes a position of a node relative to others in network. Among several ways to define centrality, betweenness centrality of a node is determined by a fraction of shortest paths between all other nodes that pass through the node. The removal of nodes with high betweenness centrality can significantly affect the functionality of networks {Holme, 2002}, even if the nodes have small degree. In biological network, the proteins with high betweenness centrality can play a crucial role in communication between proteins. Therefore, in protein networks, this property can give us distinguishable information to identify important proteins. In our paper, we measure the betweenness centrality of nodes using an algorithm from Ref. {Brandes, 2008} in order to figure out the critical proteins in the functional vulnerability of the APP network.

3. Closeness centrality

Another important parameter is the closeness centrality of proteins. The closeness centrality represents how close/far the node is to/from other nodes in a network. It is calculated as the reciprocal of the total number of shortest paths between a given node and all other nodes. The proteins with high closeness centrality in a biological network can be functionally relevant to other proteins with lower centrality.

4. Community

Community structure is another important feature of network structure. Nodes in a community are more tightly connected to each other than to nodes out of the community. Nodes in a community are usually involved in the same function. As an example, communities in protein-protein interaction networks are identified as functional modules {Jonsson, 2006}. We identified community structure without taking into account overlapping by use of a modularity matrix of Newman {Newman, 2006}. We also found overlapped proteins between communities by use of the community detecting method from Lancichinetti, *et. al.* {Lancichinetti, 2011}.

5. *k*-core

Most interacting proteins in protein complexes share the similar functions. In turn, the members of functional modules interact more tightly with each other {Gavin, 2002}. In order to understand the connectivity of proteins in the APP/APLP2 network, we used the *k*-core decomposition method {Wuchty, 2005}. *k*-core decomposition is a method to obtain strongly interconnected parts of a network recursively removing nodes with degree lower than *k*. Therefore, *k*-core is the maximal subgraphs of a network such that all nodes in the subgraph have at least *k* connections.

**References for Supplementary**

{Freeman, 1977} L. A. Freeman, A set of measures of centrality based on betweenness, Sociometry 40, pp.35-41, 1977.

{Brandes, 2008} U. Brandes, On variants of shortest-path betweenness centrality and their generic computation, Social Networks 30(2), pp.136-145, 2008.

{Holme, 2002} P. Holme, B. J. Kim, C. N. Yoon, and S. K. Kim, Attack vulnerability of complex networks, Phys. Rev. E 65, 056109, 2002.

{Newman, 2006} M. E. J. Newman, Modularity and community structure in networks, Proc. Natl. Acad. Sci. USA 103, pp.8577-8582, 2006.

{Jonsson, 2006} P. F. Jonsson, T. Cavanna, D. Zicha, and P. A. Bates, Cluster analysis of networks generated through homology: automatic identification of important protein communities involved in cancer metastasis, BMC Bioinformatics 7(2), pp. 1471-2105, 2006.

{Lancichinetti, 2011} A. Lancichinetti, F. Radicchi, J. J. Ramasco, and S. Fortunato, Finding statistical significant communities in networks, PloS ONE 6, e18961, 2011.

{Gavin, 2002} A. Gavin. et. al. Functional organization of the yeast proteome by systematic analysis of protein complexes, Nature415(10), pp.141-147, 2002.

{Wuchty, 2005} S. Wuchty and E. Almaas, Peeling the yeast protein network, Proteomics 5, pp.444-449, 2005.
