# Supplementary material for: Amyloid precursor protein interaction network in human testis: sentinel proteins for male reproduction
Source: BMC Bioinformatics. 2015 Jan 16;16(1):12. doi: 10.1186/s12859-014-0432-9 (PMC4384327; doi:10.1186/s12859-014-0432-9)
Supplement: Additional file 5: Table S4 — (a). Rank of topological properties of 457 proteins in local APP/APLP2 network. [file 12859_2014_432_MOESM5_ESM.pdf]

Table S4 Topological properties of 457 proteins in local APP/APLP2 network. (a) Rank of degree, clustering coefficient, betweenness centrality, and closeness centrality. (b) *k*-core and community that proteins are involved. Note that the number in community column is only index of the community.

(a)

Rank	Degree		Clustering Coefficient		Betweenness Centrality		Closeness Centrality	
1	**APP**	**455**	ABCB1	0	**APP**	**0.868575**	**APP**	**0.997812**
2	ANXA1	170	ACBD3	0	ANXA1	0.026885	ANXA1	0.613728
3	PIK3CG	149	ACE	0	PIK3CG	0.01889	PIK3CG	0.596859
4	PLCB3	141	ADAM33	0	PLCB3	0.015228	PLCB3	0.590674
5	LPAR2	132	ADAM8	0	LPAR2	0.013245	LPAR2	0.583867
6	RLN3	106	ADAM9	0	RLN3	0.00676	RLN3	0.565056
7	ADORA3	104	ADNP	0	ADORA3	0.006566	ADORA3	0.563659
8	CCL27	103	AGER	0	PIK3CA	0.006445	CCL27	0.562963
9	PIK3CA	84	ALB	0	CCL27	0.006258	PIK3CA	0.550725
10	GNA15	82	AP1M2	0	GNA15	0.004118	GNA15	0.548736
11	PROK1	80	APBA3	0	PROK1	0.003863	PROK1	0.547419
12	CASP3	32	APBB2	0	JUN	0.00294	CASP3	0.517594
13	JUN	28	APBB3	0	CASP3	0.002037	JUN	0.515837
14	UBC	26	APEH	0	CDK1	0.001798	UBC	0.514673
15	NGFR	20	APMAP	0	**APLP2**	**0.001447**	NGFR	0.510638
16	COPS5	19	APOA2	0	UBC	0.001173	COPS5	0.510638
17	HSPA1A	17	APOE	0	NGFR	0.000727	CDK1	0.509497
18	CDK1	17	APPBP2	0	COPS5	0.000607	HSPA1A	0.508929
19	STUB1	16	BACE2	0	PDIA3	0.000475	GAPDH	0.508929
20	PDIA3	16	BGN	0	GAPDH	0.000413	STUB1	0.508361
21	GAPDH	16	BLMH	0	HSPA1A	0.000368	PDIA3	0.508361
22	HSPA5	15	BNIP2	0	HSPA5	0.000359	HSPA5	0.507795
23	HSPB1	12	CALU	0	STUB1	0.000355	HSPB1	0.506104
24	PMCH	11	CAMLG	0	HSPB1	0.000197	PMCH	0.505543
25	NMUR2	11	CAPZA1	0	STXBP1	0.000127	NMUR2	0.505543
26	NMUR1	11	CAPZA2	0	DAB2	0.000112	NMUR1	0.505543
27	NMU	11	CASP8	0	BCAP31	0.000094	NMU	0.505543
28	MCHR2	11	CAT	0	YWHAZ	0.000083	MCHR2	0.505543
29	MCHR1	11	CCHCR1	0	NFKB1	0.000048	MCHR1	0.505543
30	GNG2	11	CD14	0	FBLN1	0.000048	GNG2	0.505543
31	GNB1	11	CD36	0	UCHL1	0.000041	GNB1	0.505543
32	FPR2	11	CHRNA7	0	CXCR4	0.000041	FPR2	0.505543
33	BDKRB2	11	CHRNB1	0	GRB2	0.000038	BDKRB2	0.505543
34	BDKRB1	11	CLPTM1L	0	HSPA8	0.000037	BDKRB1	0.505543
35	AGTR1	11	CLSTN1	0	MDM2	0.000036	AGTR1	0.505543
36	AGT	11	CLSTN3	0	HSP90AA1	0.000036	AGT	0.505543
37	YWHAZ	10	CNTN1	0	RELA	0.000031	YWHAZ	0.504983
38	LPAR3	10	CNTN2	0	C3	0.000031	LPAR3	0.504983
39	LPAR1	10	CNTN3	0	ARHGEF1	0.000025	LPAR1	0.504983
40	AGTR2	10	CNTN4	0	PIK3R1	0.000023	DAB2	0.504983
41	STXBP1	9	COL18A1	0	CD81	0.000022	CXCR4	0.504983
42	OPRK1	9	COL4A1	0	NSF	0.00002	AGTR2	0.504983
43	NPSR1	9	COL4A2	0	MTNR1A	0.00002	STXBP1	0.504425
44	HRH4	9	COL4A3	0	SNCA	0.000019	OPRK1	0.504425
45	GNA13	9	COL4A5	0	GABBR2	0.000018	NPSR1	0.504425
46	DAB2	9	COL4A6	0	GABBR1	0.000018	NFKB1	0.504425
47	CXCR4	9	CPE	0	SYVN1	0.000016	HRH4	0.504425
48	CNR1	9	CST3	0	MAPT	0.000016	GNA13	0.504425
49	CHRM2	9	CTSB	0	HSP90AB1	0.000014	CNR1	0.504425
50	C3	9	CTSD	0	CD99	0.000014	CHRM2	0.504425
51	BCAP31	9	DAB1	0	ITGB1	0.000013	C3	0.504425
52	UTS2D	8	DLG4	0	F2RL1	0.000012	BCAP31	0.504425
53	UTS2	8	DNAH1	0	EDNRB	0.000012	UTS2D	0.503867
54	TRH	8	DNM1	0	EDNRA	0.000012	UTS2	0.503867

55	SSTR3	8	DPEP1	0	ABL1	0.000012	TRH	0.503867
56	SSTR2	8	ECE1	0	SET	0.000009	SSTR3	0.503867
57	RXFP4	8	EPB41	0	HMGB1	0.000009	SSTR2	0.503867
58	RXFP3	8	EPB41L3	0	UNG	0.000008	RXFP4	0.503867
59	PYY	8	ERP44	0	SHC1	0.000008	RXFP3	0.503867
60	PROKR2	8	EXOC6	0	MAPK1	0.000007	RELA	0.503867
61	PROKR1	8	F10	0	NOTCH1	0.000006	PYY	0.503867
62	POMC	8	F12	0	HSPD1	0.000006	PROKR2	0.503867
63	PLCB2	8	F7	0	GSN	0.000006	PROKR1	0.503867
64	PLCB1	8	FAM134A	0	TGM2	0.000005	POMC	0.503867
65	PIK3R1	8	FANCM	0	NAE1	0.000005	PLCB2	0.503867
66	PENK	8	FKBP1A	0	MAPK8	0.000005	PLCB1	0.503867
67	PDYN	8	FLOT2	0	CBFB	0.000005	PIK3R1	0.503867
68	P2RY14	8	GPC1	0	TNF	0.000004	PENK	0.503867
69	P2RY1	8	GPNMB	0	PSEN1	0.000004	PDYN	0.503867
70	P2RY13	8	GSK3A	0	PMCH	0.000004	P2RY14	0.503867
71	P2RY12	8	HADH	0	NMUR2	0.000004	P2RY1	0.503867
72	P2RY11	8	HADHB	0	NMUR1	0.000004	P2RY13	0.503867
73	OPN4	8	HMOX1	0	NMU	0.000004	P2RY12	0.503867
74	NPW	8	HMOX2	0	MCHR2	0.000004	P2RY11	0.503867
75	NPBWR1	8	HOMER2	0	MCHR1	0.000004	OPN4	0.503867
76	NPB	8	HSD17B10	0	LPAR3	0.000004	NPW	0.503867
77	NMBR	8	HSP90B1	0	LPAR1	0.000004	NPBWR1	0.503867
78	NMB	8	HSPB6	0	GSK3B	0.000004	NPB	0.503867
79	NFKB1	8	HTRA1	0	GNG2	0.000004	NMBR	0.503867
80	MTNR1A	8	HTRA2	0	GNB1	0.000004	NMB	0.503867
81	LPAR6	8	IDE	0	FPR2	0.000004	MTNR1A	0.503867
82	LPAR4	8	IFIT3	0	CASP1	0.000004	MDM2	0.503867
83	HTR5A	8	ITM2A	0	BDKRB2	0.000004	LPAR6	0.503867
84	HTR2A	8	ITM2B	0	BDKRB1	0.000004	LPAR4	0.503867
85	HRH3	8	KIAA0319L	0	AGTR1	0.000004	HTR5A	0.503867
86	HRH1	8	KIAA1704	0	AGT	0.000004	HTR2A	0.503867
87	HEBP1	8	KLC1	0	PIN1	0.000003	HSPA8	0.503867
88	HCAR2	8	KLK2	0	PARK2	0.000003	HSP90AA1	0.503867
89	GRPR	8	KLK6	0	ITGB5	0.000003	HRH3	0.503867
90	GRP	8	L1CAM	0	DDB1	0.000003	HRH1	0.503867
91	GPR68	8	LAMA1	0	CLU	0.000003	HEBP1	0.503867
92	GPR65	8	LDLRAP1	0	APLP1	0.000003	HCAR2	0.503867
93	GPR55	8	LRP1	0	ADAM17	0.000003	GRPR	0.503867
94	GPR4	8	LRP1B	0	ACTB	0.000003	GRP	0.503867
95	GPR18	8	LRP8	0	PLCB2	0.000002	GPR68	0.503867
96	GPER	8	LYL1	0	PLCB1	0.000002	GPR65	0.503867
97	GNRHR2	8	LYPD3	0	MYH9	0.000002	GPR55	0.503867
98	GNRHR	8	MAPK8IP2	0	MAPK3	0.000002	GPR4	0.503867
99	GNRH1	8	MAST1	0	HSPA4	0.000002	GPR18	0.503867
100	GNAI3	8	MAT1A	0	GNA13	0.000002	GPER	0.503867
101	GNAI2	8	MBP	0	GFAP	0.000002	GNRHR2	0.503867
102	GNAI1	8	MED12	0	FOS	0.000002	GNRHR	0.503867
103	GNA12	8	MEFV	0	CDC37	0.000002	GNRH1	0.503867
104	GHSR	8	MMEL1	0	CAV1	0.000002	GNAI3	0.503867
105	GHRL	8	MMP14	0	AGTR2	0.000002	GNAI2	0.503867
106	GABBR2	8	MMP17	0	XIAP	0.000001	GNAI1	0.503867
107	GABBR1	8	MMP2	0	TUBB	0.000001	GNA12	0.503867
108	FPR3	8	MMP9	0	RCN2	0.000001	GHSR	0.503867
109	F2RL1	8	NCAM1	0	P2RY11	0.000001	GHRL	0.503867
110	EDNRB	8	NECAB3	0	OPRK1	0.000001	GABBR2	0.503867
111	EDNRA	8	NEFL	0	NPSR1	0.000001	GABBR1	0.503867
112	DRD3	8	NF1	0	HRH4	0.000001	FPR3	0.503867
113	CXCR7	8	NFASC	0	CSNK2A1	0.000001	F2RL1	0.503867
114	CXCR3	8	NLRP3	0	CNR1	0.000001	EDNRB	0.503867

115	CXCL9	8	NOTCH2	0	CHRM2	0.000001	EDNRA	0.503867
116	CXCL16	8	NUMB	0	CASP6	0.000001	DRD3	0.503867
117	CXCL12	8	NUMBL	0	BRCA1	0.000001	CXCR7	0.503867
118	CXCL10	8	OAT	0	UTS2D	0	CXCR3	0.503867
119	CHRM5	8	OSTC	0	UTS2	0	CXCL9	0.503867
120	CHRM3	8	P2RX7	0	TXNIP	0	CXCL16	0.503867
121	CCR7	8	PAK3	0	TTR	0	CXCL12	0.503867
122	CCR2	8	PCBD1	0	TSPAN6	0	CXCL10	0.503867
123	CCR10	8	PDIA6	0	TRIO	0	CHRM5	0.503867
124	CCL5	8	PDZK1P1	0	TRH	0	CHRM3	0.503867
125	CCL21	8	PGAM1	0	TPD52L2	0	CCR7	0.503867
126	CCL20	8	PI4K2A	0	TP53BP2	0	CCR2	0.503867
127	C5	8	PITRM1	0	TOMM5	0	CCR10	0.503867
128	C3AR1	8	PLG	0	TNFRSF21	0	CCL5	0.503867
129	AVPR1A	8	PPID	0	TMPRSS12	0	CCL21	0.503867
130	ARHGEF1	8	PPP1R2	0	TMEM30B	0	CCL20	0.503867
131	APLNR	8	PREP	0	TMEM30A	0	C5	0.503867
132	APLN	8	PRSS1	0	TMEM191C	0	C3AR1	0.503867
133	ADRA1A	8	PRSS2	0	TMEM191B	0	AVPR1A	0.503867
134	ADORA2A	8	PRSS3	0	TMEFF2	0	ARHGEF1	0.503867
135	UCHL1	7	PRSSL1	0	TMCC2	0	APLNR	0.503867
136	S1PR5	7	RELN	0	TM2D1	0	APLN	0.503867
137	S1PR3	7	RNF32	0	THBS1	0	ADRA1A	0.503867
138	S1PR2	7	SEC22C	0	TGFB2	0	ADORA2A	0.503867
139	S1PR1	7	SERPINA3	0	TGFB1	0	UCHL1	0.503311
140	RELA	7	SLC40A1	0	TBC1D7	0	S1PR5	0.503311
141	PTGFR	7	SMUG1	0	TAF9	0	S1PR3	0.503311
142	PTGER3	7	SNX17	0	TAF15	0	S1PR2	0.503311
143	PTAFR	7	SORL1	0	SYNRG	0	S1PR1	0.503311
144	PROK2	7	SPARCL1	0	SUGT1	0	PTGFR	0.503311
145	PIK3R3	7	SPON1	0	STX5	0	PTGER3	0.503311
146	PIK3R2	7	SRGAP3	0	SSTR3	0	PTAFR	0.503311
147	OXER1	7	SSPN	0	SSTR2	0	PROK2	0.503311
148	NTSR2	7	SUGT1	0	SSPN	0	PIK3R3	0.503311
149	NPY5R	7	SYNRG	0	SRGAP3	0	PIK3R2	0.503311
150	NPY1R	7	TBC1D7	0	SPTAN1	0	OXER1	0.503311
151	NPFFR2	7	TGFB1	0	SPON1	0	NTSR2	0.503311
152	NPFFR1	7	TGFB2	0	SPARCL1	0	NPY5R	0.503311
153	MDM2	7	THBS1	0	SORL1	0	NPY1R	0.503311
154	LTB4R2	7	TM2D1	0	SNX17	0	NPFFR2	0.503311
155	HSPA8	7	TMCC2	0	SMUG1	0	NPFFR1	0.503311
156	HSP90AA1	7	TMEFF2	0	SLC40A1	0	LTB4R2	0.503311
157	GRB2	7	TMEM191B	0	SERPINA3	0	GRB2	0.503311
158	GPRC6A	7	TMEM191C	0	SEC22C	0	GPRC6A	0.503311
159	GPR44	7	TMEM30A	0	S1PR5	0	GPR44	0.503311
160	GNAZ	7	TMEM30B	0	S1PR3	0	GNAZ	0.503311
161	GNAQ	7	TMPRSS12	0	S1PR2	0	GNAQ	0.503311
162	GNA14	7	TNFRSF21	0	S1PR1	0	GNA14	0.503311
163	GNA11	7	TOMM5	0	RXFP4	0	GNA11	0.503311
164	GALR1	7	TPD52L2	0	RXFP3	0	GALR1	0.503311
165	F2RL3	7	TSPAN6	0	RTN4R	0	F2RL3	0.503311
166	F2RL2	7	TXNIP	0	RPN2	0	F2RL2	0.503311

167	F2R	7	**APP**	**0.013022**	RNF32	0	F2R	0.503311
168	CCL28	7	ANXA1	0.076157	RELN	0	CCL28	0.503311
169	CCKBR	7	PLCB3	0.077508	RANBP9	0	CCKBR	0.503311
170	ADRA2A	7	PIK3CG	0.077725	PYY	0	**APLP2**	**0.503311**
171	ADORA1	7	CASP3	0.080645	PTGFR	0	ADRA2A	0.503311
172	HSP90AB1	6	JUN	0.082011	PTGER3	0	ADORA1	0.503311
173	FBLN1	6	LPAR2	0.102591	PTAFR	0	MAPT	0.502756
174	ARHGEF12	6	PIK3CA	0.11704	PSEN2	0	HSP90AB1	0.502756
175	**APLP2**	**6**	RLN3	0.119137	PRSSL1	0	FBLN1	0.502756
176	ADRBK1	6	ADORA3	0.121359	PRSS3	0	ARHGEF12	0.502756
177	ADCY9	6	CCL27	0.123358	PRSS2	0	ADRBK1	0.502756
178	ADCY8	6	NGFR	0.126316	PRSS1	0	ADCY9	0.502756
179	ADCY7	6	UBC	0.129231	PROKR2	0	ADCY8	0.502756
180	ADCY6	6	GNA15	0.147847	PROKR1	0	ADCY7	0.502756
181	ADCY5	6	PDIA3	0.15	PROK2	0	ADCY6	0.502756
182	ADCY4	6	PROK1	0.152848	PRNP	0	ADCY5	0.502756
183	ADCY3	6	CDK1	0.161765	PREP	0	ADCY4	0.502756
184	ADCY2	6	GAPDH	0.175	PPP1R2	0	ADCY3	0.502756
185	ADCY1	6	HSPA5	0.180952	PPID	0	ADCY2	0.502756
186	TRIO	5	COPS5	0.181287	PPIB	0	ADCY1	0.502756
187	SYVN1	5	STUB1	0.191667	PPIA	0	ABL1	0.502756
188	SNCA	5	STXBP1	0.222222	POMC	0	TRIO	0.502203
189	PLCB4	5	HSPA1A	0.235294	PLG	0	SYVN1	0.502203
190	NSF	5	HSPB1	0.272727	PLD1	0	SNCA	0.502203
191	MAPT	5	DAB2	0.305556	PLCB4	0	SET	0.502203
192	KALRN	5	FBLN1	0.333333	PITRM1	0	PLCB4	0.502203
193	GRK5	5	GRB2	0.333333	PIK3R3	0	PIN1	0.502203
194	CD81	5	HSP90AA1	0.333333	PIK3R2	0	NSF	0.502203
195	ARHGEF25	5	RELA	0.333333	PI4K2A	0	MAPK8	0.502203
196	ABL1	5	YWHAZ	0.333333	PGAM1	0	MAPK1	0.502203
197	UNG	4	BCAP31	0.361111	PENK	0	KALRN	0.502203
198	TGM2	4	HSPA8	0.380952	PDZK1P1	0	HMGB1	0.502203
199	SHC1	4	MDM2	0.380952	PDYN	0	GSK3B	0.502203
200	SET	4	UCHL1	0.380952	PDIA6	0	GRK5	0.502203
201	PSEN1	4	NFKB1	0.392857	PDIA4	0	CD81	0.502203
202	PIN1	4	ABL1	0.4	PCBD1	0	ARHGEF25	0.502203
203	PARK2	4	CD81	0.4	PAK3	0	UNG	0.50165
204	NOTCH1	4	MAPT	0.4	P2RY14	0	TUBB	0.50165
205	MAPK8	4	NSF	0.4	P2RY1	0	TNF	0.50165
206	MAPK1	4	**APLP2**	**0.466667**	P2RY13	0	TGM2	0.50165
207	ITGB1	4	HSP90AB1	0.466667	P2RY12	0	SHC1	0.50165
208	HSPD1	4	CD99	0.5	P2RX7	0	PSEN1	0.50165
209	HMGB1	4	HMGB1	0.5	OXER1	0	PARK2	0.50165
210	GSN	4	HSPD1	0.5	OSTC	0	NOTCH1	0.50165
211	GSK3B	4	ITGB1	0.5	OPN4	0	MAPK3	0.50165
212	CD99	4	MAPK1	0.5	OAT	0	KAT5	0.50165
213	CASP1	4	SHC1	0.5	NUMBL	0	ITGB1	0.50165
214	ACTB	4	SNCA	0.5	NUMB	0	HSPD1	0.50165
215	XIAP	3	TGM2	0.5	NTSR2	0	HSPA4	0.50165
216	TUBB	3	SYVN1	0.6	NPY5R	0	GSN	0.50165
217	TNF	3	ACTB	0.666667	NPY1R	0	GFAP	0.50165
218	RCN2	3	ADAM17	0.666667	NPW	0	FOS	0.50165

219	PSEN2	3	APLP1	0.666667	NPFFR2	0	CSNK2A1	0.50165
220	NAE1	3	BRCA1	0.666667	NPFFR1	0	CD99	0.50165
221	MYH9	3	CASP1	0.666667	NPBWR1	0	CBFB	0.50165
222	MME	3	CASP6	0.666667	NPB	0	CASP1	0.50165
223	MAPK3	3	CAV1	0.666667	NOTCH2	0	ACTB	0.50165
224	MAP3K5	3	CBFB	0.666667	NMBR	0	XIAP	0.501099
225	KAT5	3	CDC37	0.666667	NMB	0	RCN2	0.501099
226	ITGB5	3	CLU	0.666667	NLRP3	0	PSEN2	0.501099
227	HSPA4	3	CSNK2A1	0.666667	NID1	0	NAE1	0.501099
228	GFAP	3	DDB1	0.666667	NFKB2	0	MYH9	0.501099
229	FOS	3	FOS	0.666667	NFASC	0	MME	0.501099
230	DDB1	3	GFAP	0.666667	NF1	0	MAPK10	0.501099
231	CSNK2A1	3	GSK3B	0.666667	NEFL	0	MAP3K5	0.501099
232	CRYAB	3	GSN	0.666667	NECAB3	0	ITGB5	0.501099
233	COL1A2	3	HSPA4	0.666667	NCSTN	0	GTF2F2	0.501099
234	CLU	3	ITGB5	0.666667	NCAM1	0	DDB1	0.501099
235	CDC37	3	MAPK3	0.666667	MMP9	0	CRYAB	0.501099
236	CBFB	3	MAPK8	0.666667	MMP2	0	CREB3	0.501099
237	CAV1	3	MYH9	0.666667	MMP17	0	CREB1	0.501099
238	CASP6	3	NAE1	0.666667	MMP14	0	CPEB1	0.501099
239	CANX	3	NOTCH1	0.666667	MMEL1	0	COL1A2	0.501099
240	CALR	3	PARK2	0.666667	MME	0	CLU	0.501099
241	BRCA1	3	PIN1	0.666667	MEFV	0	CDC37	0.501099
242	ATF6	3	PSEN1	0.666667	MED12	0	CAV1	0.501099
243	APLP1	3	RCN2	0.666667	MBP	0	CASP6	0.501099
244	ADAM17	3	SET	0.666667	MAT1A	0	CANX	0.501099
245	TTR	2	TNF	0.666667	MAST1	0	CALR	0.501099
246	TP53BP2	2	TUBB	0.666667	MAPK8IP2	0	ATF6	0.501099
247	TAF9	2	UNG	0.666667	MAPK8IP1	0	APLP1	0.501099
248	TAF15	2	XIAP	0.666667	MAPK10	0	ADAM17	0.501099
249	STX5	2	ARHGEF1	0.678571	MAP3K5	0	ACHE	0.501099
250	SPTAN1	2	EDNRA	0.714286	MAP3K11	0	TTR	0.500549
251	RTN4R	2	EDNRB	0.714286	LYPD3	0	TP53BP2	0.500549
252	RPN2	2	F2RL1	0.714286	LYL1	0	TAF9	0.500549
253	RANBP9	2	GABBR1	0.75	LTB4R2	0	TAF15	0.500549
254	PRNP	2	GABBR2	0.75	LRP8	0	STX5	0.500549
255	PPIB	2	MTNR1A	0.75	LRP1B	0	SPTAN1	0.500549
256	PPIA	2	LPAR1	0.755556	LRP1	0	RTN4R	0.500549
257	PLD1	2	LPAR3	0.755556	LPAR6	0	RPN2	0.500549
258	PDIA4	2	C3	0.777778	LPAR4	0	RANBP9	0.500549
259	NID1	2	CXCR4	0.777778	LINGO1	0	PRNP	0.500549
260	NFKB2	2	PIK3R1	0.785714	LDLRAP1	0	PPIB	0.500549
261	NCSTN	2	PLCB1	0.785714	LAMA1	0	PPIA	0.500549
262	MAPK8IP1	2	PLCB2	0.785714	L1CAM	0	PLD1	0.500549
263	MAPK10	2	AGT	0.8	KLK6	0	PDIA4	0.500549
264	MAP3K11	2	AGTR1	0.8	KLK2	0	NID1	0.500549
265	LINGO1	2	BDKRB1	0.8	KLC1	0	NFKB2	0.500549
266	HYOU1	2	BDKRB2	0.8	KIAA1704	0	NCSTN	0.500549
267	HSPG2	2	FPR2	0.8	KIAA0319L	0	MAPK8IP1	0.500549
268	HSPB8	2	GNB1	0.8	KAT5	0	MAP3K11	0.500549
269	HGS	2	GNG2	0.8	KALRN	0	LINGO1	0.500549
270	GTF2F2	2	MCHR1	0.8	ITM2B	0	HYOU1	0.500549

271	GNAO1	2	MCHR2	0.8	ITM2A	0	HSPG2	0.500549
272	GANAB	2	NMU	0.8	IFIT3	0	HSPB8	0.500549
273	FTL	2	NMUR1	0.8	IDE	0	HGS	0.500549
274	FLOT1	2	NMUR2	0.8	HYOU1	0	GNAO1	0.500549
275	CREB3	2	PMCH	0.8	HTRA2	0	GANAB	0.500549
276	CREB1	2	P2RY11	0.821429	HTRA1	0	FTL	0.500549
277	CPEB1	2	AGTR2	0.844444	HTR5A	0	FLOT1	0.500549
278	COL25A1	2	GNA13	0.861111	HTR2A	0	COL25A1	0.500549
279	CDK5	2	NPSR1	0.861111	HSPG2	0	CDK5	0.500549
280	CASP4	2	CHRM2	0.888889	HSPB8	0	CASP4	0.500549
281	BCAP29	2	CNR1	0.888889	HSPB6	0	BCAP29	0.500549
282	BACE1	2	HRH4	0.888889	HSP90B1	0	BACE1	0.500549
283	APOA1	2	OPRK1	0.888889	HSD17B10	0	APOA1	0.500549
284	APCS	2	CCKBR	0.904762	HRH3	0	APCS	0.500549
285	APBB1	2	F2R	0.904762	HRH1	0	APBB1	0.500549
286	APBA2	2	F2RL2	0.904762	HOMER2	0	APBA2	0.500549
287	APBA1	2	F2RL3	0.904762	HMOX2	0	APBA1	0.500549
288	AGRN	2	GNA11	0.904762	HMOX1	0	AGRN	0.500549
289	ADAM10	2	GNA14	0.904762	HGS	0	ADAM10	0.500549
290	ACHE	2	GNAQ	0.904762	HEBP1	0	A2M	0.500549
291	A2M	2	GPRC6A	0.904762	HCAR2	0	TXNIP	0.5
292	TXNIP	1	LTB4R2	0.904762	HADHB	0	TSPAN6	0.5
293	TSPAN6	1	NPFFR1	0.904762	HADH	0	TPD52L2	0.5
294	TPD52L2	1	NPFFR2	0.904762	GTF2F2	0	TOMM5	0.5
295	TOMM5	1	NTSR2	0.904762	GSK3A	0	TNFRSF21	0.5
296	TNFRSF21	1	PROK2	0.904762	GRPR	0	TMPRSS12	0.5
297	TMPRSS12	1	PTAFR	0.904762	GRP	0	TMEM30B	0.5
298	TMEM30B	1	PTGFR	0.904762	GRK5	0	TMEM30A	0.5
299	TMEM30A	1	ADRA1A	0.928571	GPRC6A	0	TMEM191C	0.5
300	TMEM191C	1	AVPR1A	0.928571	GPR68	0	TMEM191B	0.5
301	TMEM191B	1	CHRM3	0.928571	GPR65	0	TMEFF2	0.5
302	TMEFF2	1	CHRM5	0.928571	GPR55	0	TMCC2	0.5
303	TMCC2	1	GHRL	0.928571	GPR44	0	TM2D1	0.5
304	TM2D1	1	GHSR	0.928571	GPR4	0	THBS1	0.5
305	THBS1	1	GNRH1	0.928571	GPR18	0	TGFB2	0.5
306	TGFB2	1	GNRHR	0.928571	GPNMB	0	TGFB1	0.5
307	TGFB1	1	GNRHR2	0.928571	GPER	0	TBC1D7	0.5
308	TBC1D7	1	GPR4	0.928571	GPC1	0	SYNRG	0.5
309	SYNRG	1	GPR65	0.928571	GNRHR2	0	SUGT1	0.5
310	SUGT1	1	GPR68	0.928571	GNRHR	0	SSPN	0.5
311	SSPN	1	GRP	0.928571	GNRH1	0	SRGAP3	0.5
312	SRGAP3	1	GRPR	0.928571	GNAZ	0	SPON1	0.5
313	SPON1	1	HRH1	0.928571	GNAQ	0	SPARCL1	0.5
314	SPARCL1	1	HTR2A	0.928571	GNAO1	0	SORL1	0.5
315	SORL1	1	LPAR4	0.928571	GNAI3	0	SNX17	0.5
316	SNX17	1	LPAR6	0.928571	GNAI2	0	SMUG1	0.5
317	SMUG1	1	NMB	0.928571	GNAI1	0	SLC40A1	0.5
318	SLC40A1	1	NMBR	0.928571	GNA14	0	SERPINA3	0.5
319	SERPINA3	1	OPN4	0.928571	GNA12	0	SEC22C	0.5
320	SEC22C	1	P2RY1	0.928571	GNA11	0	RNF32	0.5
321	RNF32	1	PROKR1	0.928571	GHSR	0	RELN	0.5
322	RELN	1	PROKR2	0.928571	GHRL	0	PRSSL1	0.5

323	PRSSL1	1	TRH	0.928571	GANAB	0	PRSS3	0.5
324	PRSS3	1	UTS2	0.928571	GALR1	0	PRSS2	0.5
325	PRSS2	1	UTS2D	0.928571	FTL	0	PRSS1	0.5
326	PRSS1	1	ADORA1	0.952381	FPR3	0	PREP	0.5
327	PREP	1	ADRA2A	0.952381	FLOT2	0	PPP1R2	0.5
328	PPP1R2	1	CCL28	0.952381	FLOT1	0	PPID	0.5
329	PPID	1	GALR1	0.952381	FKBP1A	0	PLG	0.5
330	PLG	1	GPR44	0.952381	FANCM	0	PITRM1	0.5
331	PITRM1	1	NPY1R	0.952381	FAM134A	0	PI4K2A	0.5
332	PI4K2A	1	NPY5R	0.952381	F7	0	PGAM1	0.5
333	PGAM1	1	OXER1	0.952381	F2RL3	0	PDZK1P1	0.5
334	PDZK1P1	1	PTGER3	0.952381	F2RL2	0	PDIA6	0.5
335	PDIA6	1	S1PR1	0.952381	F2R	0	PCBD1	0.5
336	PCBD1	1	S1PR2	0.952381	F12	0	PAK3	0.5
337	PAK3	1	S1PR3	0.952381	F10	0	P2RX7	0.5
338	P2RX7	1	S1PR5	0.952381	EXOC6	0	OSTC	0.5
339	OSTC	1	ADORA2A	0.964286	ERP44	0	OAT	0.5
340	OAT	1	APLN	0.964286	EPB41L3	0	NUMBL	0.5
341	NUMBL	1	APLNR	0.964286	EPB41	0	NUMB	0.5
342	NUMB	1	C3AR1	0.964286	ECE1	0	NOTCH2	0.5
343	NOTCH2	1	C5	0.964286	DRD3	0	NLRP3	0.5
344	NLRP3	1	CCL20	0.964286	DPEP1	0	NFASC	0.5
345	NFASC	1	CCL21	0.964286	DNM1	0	NF1	0.5
346	NF1	1	CCL5	0.964286	DNAH1	0	NEFL	0.5
347	NEFL	1	CCR10	0.964286	DLG4	0	NECAB3	0.5
348	NECAB3	1	CCR2	0.964286	DAB1	0	NCAM1	0.5
349	NCAM1	1	CCR7	0.964286	CXCR7	0	MMP9	0.5
350	MMP9	1	CXCL10	0.964286	CXCR3	0	MMP2	0.5
351	MMP2	1	CXCL12	0.964286	CXCL9	0	MMP17	0.5
352	MMP17	1	CXCL16	0.964286	CXCL16	0	MMP14	0.5
353	MMP14	1	CXCL9	0.964286	CXCL12	0	MMEL1	0.5
354	MMEL1	1	CXCR3	0.964286	CXCL10	0	MEFV	0.5
355	MEFV	1	CXCR7	0.964286	CTSD	0	MED12	0.5
356	MED12	1	DRD3	0.964286	CTSB	0	MBP	0.5
357	MBP	1	FPR3	0.964286	CST3	0	MAT1A	0.5
358	MAT1A	1	GNA12	0.964286	CRYAB	0	MAST1	0.5
359	MAST1	1	GNAI1	0.964286	CREB3	0	MAPK8IP2	0.5
360	MAPK8IP2	1	GNAI2	0.964286	CREB1	0	LYPD3	0.5
361	LYPD3	1	GNAI3	0.964286	CPEB1	0	LYL1	0.5
362	LYL1	1	GPER	0.964286	CPE	0	LRP8	0.5
363	LRP8	1	GPR18	0.964286	COL4A6	0	LRP1B	0.5
364	LRP1B	1	GPR55	0.964286	COL4A5	0	LRP1	0.5
365	LRP1	1	HCAR2	0.964286	COL4A3	0	LDLRAP1	0.5
366	LDLRAP1	1	HEBP1	0.964286	COL4A2	0	LAMA1	0.5
367	LAMA1	1	HRH3	0.964286	COL4A1	0	L1CAM	0.5
368	L1CAM	1	HTR5A	0.964286	COL25A1	0	KLK6	0.5
369	KLK6	1	NPB	0.964286	COL1A2	0	KLK2	0.5
370	KLK2	1	NPBWR1	0.964286	COL18A1	0	KLC1	0.5
371	KLC1	1	NPW	0.964286	CNTN4	0	KIAA1704	0.5
372	KIAA1704	1	P2RY12	0.964286	CNTN3	0	KIAA0319L	0.5
373	KIAA0319L	1	P2RY13	0.964286	CNTN2	0	ITM2B	0.5
374	ITM2B	1	P2RY14	0.964286	CNTN1	0	ITM2A	0.5

375	ITM2A	1	PDYN	0.964286	CLSTN3	0	IFIT3	0.5
376	IFIT3	1	PENK	0.964286	CLSTN1	0	IDE	0.5
377	IDE	1	POMC	0.964286	CLPTM1L	0	HTRA2	0.5
378	HTRA2	1	PYY	0.964286	CHRNB1	0	HTRA1	0.5
379	HTRA1	1	RXFP3	0.964286	CHRNA7	0	HSPB6	0.5
380	HSPB6	1	RXFP4	0.964286	CHRM5	0	HSP90B1	0.5
381	HSP90B1	1	SSTR2	0.964286	CHRM3	0	HSD17B10	0.5
382	HSD17B10	1	SSTR3	0.964286	CDK5	0	HOMER2	0.5
383	HOMER2	1	A2M	1	CD36	0	HMOX2	0.5
384	HMOX2	1	ACHE	1	CD14	0	HMOX1	0.5
385	HMOX1	1	ADAM10	1	CCR7	0	HADHB	0.5
386	HADHB	1	ADCY1	1	CCR2	0	HADH	0.5
387	HADH	1	ADCY2	1	CCR10	0	GSK3A	0.5
388	GSK3A	1	ADCY3	1	CCL5	0	GPNMB	0.5
389	GPNMB	1	ADCY4	1	CCL28	0	GPC1	0.5
390	GPC1	1	ADCY5	1	CCL21	0	FLOT2	0.5
391	FLOT2	1	ADCY6	1	CCL20	0	FKBP1A	0.5
392	FKBP1A	1	ADCY7	1	CCKBR	0	FANCM	0.5
393	FANCM	1	ADCY8	1	CCHCR1	0	FAM134A	0.5
394	FAM134A	1	ADCY9	1	CAT	0	F7	0.5
395	F7	1	ADRBK1	1	CASP8	0	F12	0.5
396	F12	1	AGRN	1	CASP4	0	F10	0.5
397	F10	1	APBA1	1	CAPZA2	0	EXOC6	0.5
398	EXOC6	1	APBA2	1	CAPZA1	0	ERP44	0.5
399	ERP44	1	APBB1	1	CANX	0	EPB41L3	0.5
400	EPB41L3	1	APCS	1	CAMLG	0	EPB41	0.5
401	EPB41	1	APOA1	1	CALU	0	ECE1	0.5
402	ECE1	1	ARHGEF12	1	CALR	0	DPEP1	0.5
403	DPEP1	1	ARHGEF25	1	C5	0	DNM1	0.5
404	DNM1	1	ATF6	1	C3AR1	0	DNAH1	0.5
405	DNAH1	1	BACE1	1	BNIP2	0	DLG4	0.5
406	DLG4	1	BCAP29	1	BLMH	0	DAB1	0.5
407	DAB1	1	CALR	1	BGN	0	CTSD	0.5
408	CTSD	1	CANX	1	BCAP29	0	CTSB	0.5
409	CTSB	1	CASP4	1	BACE2	0	CST3	0.5
410	CST3	1	CDK5	1	BACE1	0	CPE	0.5
411	CPE	1	COL1A2	1	AVPR1A	0	COL4A6	0.5
412	COL4A6	1	COL25A1	1	ATF6	0	COL4A5	0.5
413	COL4A5	1	CPEB1	1	ARHGEF25	0	COL4A3	0.5
414	COL4A3	1	CREB1	1	ARHGEF12	0	COL4A2	0.5
415	COL4A2	1	CREB3	1	APPBP2	0	COL4A1	0.5
416	COL4A1	1	CRYAB	1	APOE	0	COL18A1	0.5
417	COL18A1	1	FLOT1	1	APOA2	0	CNTN4	0.5
418	CNTN4	1	FTL	1	APOA1	0	CNTN3	0.5
419	CNTN3	1	GANAB	1	APMAP	0	CNTN2	0.5
420	CNTN2	1	GNAO1	1	APLNR	0	CNTN1	0.5
421	CNTN1	1	GNAZ	1	APLN	0	CLSTN3	0.5
422	CLSTN3	1	GRK5	1	APEH	0	CLSTN1	0.5
423	CLSTN1	1	GTF2F2	1	APCS	0	CLPTM1L	0.5
424	CLPTM1L	1	HGS	1	APBB3	0	CHRNB1	0.5
425	CHRNB1	1	HSPB8	1	APBB2	0	CHRNA7	0.5
426	CHRNA7	1	HSPG2	1	APBB1	0	CD36	0.5

427	CD36	1	HYOU1	1	APBA3	0	CD14	0.5
428	CD14	1	KALRN	1	APBA2	0	CCHCR1	0.5
429	CCHCR1	1	KAT5	1	APBA1	0	CAT	0.5
430	CAT	1	LINGO1	1	AP1M2	0	CASP8	0.5
431	CASP8	1	MAP3K11	1	ALB	0	CAPZA2	0.5
432	CAPZA2	1	MAP3K5	1	AGRN	0	CAPZA1	0.5
433	CAPZA1	1	MAPK10	1	AGER	0	CAMLG	0.5
434	CAMLG	1	MAPK8IP1	1	ADRBK1	0	CALU	0.5
435	CALU	1	MME	1	ADRA2A	0	BNIP2	0.5
436	BNIP2	1	NCSTN	1	ADRA1A	0	BLMH	0.5
437	BLMH	1	NFKB2	1	ADORA2A	0	BGN	0.5
438	BGN	1	NID1	1	ADORA1	0	BACE2	0.5
439	BACE2	1	PDIA4	1	ADNP	0	APPBP2	0.5
440	APPBP2	1	PIK3R2	1	ADCY9	0	APOE	0.5
441	APOE	1	PIK3R3	1	ADCY8	0	APOA2	0.5
442	APOA2	1	PLCB4	1	ADCY7	0	APMAP	0.5
443	APMAP	1	PLD1	1	ADCY6	0	APEH	0.5
444	APEH	1	PPIA	1	ADCY5	0	APBB3	0.5
445	APBB3	1	PPIB	1	ADCY4	0	APBB2	0.5
446	APBB2	1	PRNP	1	ADCY3	0	APBA3	0.5
447	APBA3	1	PSEN2	1	ADCY2	0	AP1M2	0.5
448	AP1M2	1	RANBP9	1	ADCY1	0	ALB	0.5
449	ALB	1	RPN2	1	ADAM9	0	AGER	0.5
450	AGER	1	RTN4R	1	ADAM8	0	ADNP	0.5
451	ADNP	1	SPTAN1	1	ADAM33	0	ADAM9	0.5
452	ADAM9	1	STX5	1	ADAM10	0	ADAM8	0.5
453	ADAM8	1	TAF15	1	ACHE	0	ADAM33	0.5
454	ADAM33	1	TAF9	1	ACE	0	ACE	0.5
455	ACE	1	TP53BP2	1	ACBD3	0	ACBD3	0.5
456	ACBD3	1	TRIO	1	ABCB1	0	ABCB1	0.5
457	ABCB1	1	TTR	1	A2M	0	BRCA1	0.343632

(b)

k-core			Community		
gene symbols	uniprot ID	*k-core*	gene symbols	uniprot ID	index of comm.
RLN3	Q8WXF3	11	A2M	P01023	1
PROK1	P58294	11	ABCB1	P08183	1
PMCH	P20382	11	ACBD3	Q9H3P7	1
PLCB3	Q01970	11	ACE	P12821	1
PIK3CG	P48736	11	ADAM10	O14672	1
PIK3CA	P42336	11	ADAM17	P78536	1
NMUR2	Q9GZQ4	11	ADAM33	Q9BZ11	1
NMUR1	Q9HB89	11	ADAM8	P78325	1
NMU	P48645	11	ADAM9	Q13443	1
MCHR2	Q969V1	11	ADNP	Q9H2P0	1
MCHR1	Q99705	11	AGER	Q15109	1
LPAR2	Q9HBW0	11	AGRN	O00468	1
GNG2	P59768	11	ALB	P02768	1
GNB1	P62873	11	AP1M2	Q9Y6Q5	1
GNA15	P30679	11	APBA1	Q02410	1
FPR2	P25090	11	APBA2	Q99767	1
CCL27	Q9Y4X3	11	APBA3	O96018	1
BDKRB2	P30411	11	APBB2	Q92870	1
BDKRB1	P46663	11	APBB3	O95704	1
**APP**	P05067	**11**	APEH	P13798	1
ANXA1	P04083	11	APMAP	Q9HDC9	1
AGTR1	P30556	11	APOA1	P02647	1
AGT	P01019	11	APOA2	P02652	1
ADORA3	P33765	11	APOE	P02649	1
LPAR3	Q9UBY5	10	**APP**	P05067	**1**
LPAR1	Q92633	10	APPBP2	Q92624	1
AGTR2	P50052	10	ATF6	P18850	1
OPRK1	P41145	9	BACE1	P56817	1
NPSR1	Q6W5P4	9	BACE2	Q9Y5Z0	1
HRH4	Q9H3N8	9	BGN	P21810	1
GNA13	Q14344	9	BLMH	Q13867	1
CNR1	P21554	9	BNIP2	Q12982	1
CHRM2	P08172	9	CALR	P27797	1
UTS2D	Q765I0	8	CALU	O43852	1
UTS2	O95399	8	CAMLG	P49069	1
TRH	P20396	8	CANX	P27824	1
SSTR3	P32745	8	CAPZA1	P52907	1
SSTR2	P30874	8	CAPZA2	P47755	1
RXFP4	Q8TDU9	8	CASP8	Q14790	1
RXFP3	Q9NSD7	8	CAT	P04040	1
PYY	P10082	8	CAV1	Q03135	1
PROKR2	Q8NFJ6	8	CBFB	Q13951	1
PROKR1	Q8TCW9	8	CCHCR1	Q8TD31	1
POMC	P01189	8	CD14	P08571	1
PLCB2	Q00722	8	CD36	P16671	1
PLCB1	Q9NQ66	8	CD81	P60033	1
PENK	P01210	8	CD99	P14209	1
PDYN	P01213	8	CDK5	Q00535	1
P2RY14	Q15391	8	CHRNA7	P36544	1
P2RY1	P47900	8	CHRNB1	P11230	1
P2RY13	Q9BPV8	8	CLPTM1L	Q96KA5	1
P2RY12	Q9H244	8	CLSTN1	O94985	1
P2RY11	Q96G91	8	CLSTN3	Q9BQT9	1

OPN4	Q9UHM6	8	CLU	P10909	1
NPW	Q8N729	8	CNTN1	Q12860	1
NPBWR1	P48145	8	CNTN2	Q02246	1
NPB	Q8NG41	8	CNTN3	Q9P232	1
NMBR	P28336	8	CNTN4	Q8IWV2	1
NMB	P08949	8	COL18A1	P39060	1
LPAR6	P43657	8	COL4A1	P02462	1
LPAR4	Q99677	8	COL4A2	P08572	1
HTR5A	P47898	8	COL4A3	Q01955	1
HTR2A	P28223	8	COL4A5	P29400	1
HRH3	Q9Y5N1	8	COL4A6	Q14031	1
HRH1	P35367	8	CPE	P16870	1
HEBP1	Q9NRV9	8	CST3	P01034	1
HCAR2	Q8TDS4	8	CTSB	P07858	1
GRPR	P30550	8	CTSD	P07339	1
GRP	P07492	8	DAB1	O75553	1
GPR68	Q15743	8	DLG4	P78352	1
GPR65	Q8IYL9	8	DNAH1	Q9P2D7	1
GPR55	Q9Y2T6	8	DNM1	Q05193	1
GPR4	P46093	8	DPEP1	P16444	1
GPR18	Q14330	8	ECE1	P42892	1
GPER	Q99527	8	EPB41	P11171	1
GNRHR2	Q96P88	8	EPB41L3	Q9Y2J2	1
GNRHR	P30968	8	ERP44	Q9BS26	1
GNRH1	P01148	8	EXOC6	Q8TAG9	1
GNAI3	P08754	8	F10	P00742	1
GNAI2	P04899	8	F12	P00748	1
GNAI1	P63096	8	F7	P08709	1
GNA12	Q03113	8	FAM134A	Q8NC44	1
GHSR	Q92847	8	FANCM	Q8IYD8	1
GHRL	Q9UBU3	8	FBLN1	P23142	1
FPR3	P25089	8	FKBP1A	P62942	1
DRD3	P35462	8	FLOT2	Q14254	1
CXCR7	P25106	8	GANAB	Q14697	1
CXCR4	P61073	8	GPC1	P35052	1
CXCR3	P49682	8	GPNMB	Q14956	1
CXCL9	Q07325	8	GSK3A	P49840	1
CXCL16	Q9H2A7	8	HADH	Q16836	1
CXCL12	P48061	8	HADHB	P55084	1
CXCL10	P02778	8	HGS	O14964	1
CHRM5	P08912	8	HMOX1	P09601	1
CHRM3	P20309	8	HMOX2	P30519	1
CCR7	P32248	8	HOMER2	Q9NSB8	1
CCR2	P41597	8	HSD17B10	Q99714	1
CCR10	P46092	8	HSP90B1	P14625	1
CCL5	P13501	8	HSPA5	P11021	1
CCL21	O00585	8	HSPB6	O14558	1
CCL20	P78556	8	HSPG2	P98160	1
C5	P01031	8	HTRA1	Q92743	1
C3AR1	Q16581	8	HTRA2	O43464	1
C3	P01024	8	IDE	P14735	1
AVPR1A	P37288	8	IFIT3	O14879	1
APLNR	P35414	8	ITGB1	P05556	1
APLN	Q9ULZ1	8	ITGB5	P18084	1
ADRA1A	P35348	8	ITM2A	O43736	1
ADORA2A	P29274	8	ITM2B	Q9Y287	1
S1PR5	Q9H228	7	KIAA0319L	Q8IZA0	1
S1PR3	Q99500	7	KIAA1704	Q8IXQ4	1
S1PR2	O95136	7	KLC1	Q07866	1

S1PR1	P21453	7	KLK2	P20151	1
PTGFR	P43088	7	KLK6	Q92876	1
PTGER3	P43115	7	L1CAM	P32004	1
PTAFR	P25105	7	LAMA1	P25391	1
PROK2	Q9HC23	7	LDLRAP1	Q5SW96	1
PIK3R3	Q92569	7	LRP1	Q07954	1
PIK3R2	O00459	7	LRP1B	Q9NZR2	1
PIK3R1	P27986	7	LRP8	Q14114	1
OXER1	Q8TDS5	7	LYL1	P12980	1
NTSR2	O95665	7	LYPD3	O95274	1
NPY5R	Q15761	7	MAPK8IP2	Q13387	1
NPY1R	P25929	7	MAST1	Q9Y2H9	1
NPFFR2	Q9Y5X5	7	MAT1A	Q00266	1
NPFFR1	Q9GZQ6	7	MBP	P02686	1
MTNR1A	P48039	7	MED12	Q93074	1
LTB4R2	Q9NPC1	7	MEFV	O15553	1
GPRC6A	Q5T6X5	7	MMEL1	Q495T6	1
GPR44	GPR44	7	MMP14	P50281	1
GNAZ	P19086	7	MMP17	Q9ULZ9	1
GNAQ	P50148	7	MMP2	P08253	1
GNA14	O95837	7	MMP9	P14780	1
GNA11	P29992	7	NAE1	Q13564	1
GALR1	P47211	7	NCAM1	P13591	1
GABBR2	O75899	7	NECAB3	Q96P71	1
GABBR1	Q9UBS5	7	NEFL	P07196	1
F2RL3	Q96RI0	7	NF1	P21359	1
F2RL2	O00254	7	NFASC	O94856	1
F2RL1	P55085	7	NFKB2	Q00653	1
F2R	P25116	7	NID1	P14543	1
EDNRB	P24530	7	NLRP3	Q96P20	1
EDNRA	P25101	7	NOTCH2	Q04721	1
CCL28	Q9NRJ3	7	NSF	P46459	1
CCKBR	P32239	7	NUMB	P49757	1
ARHGEF1	Q92888	7	NUMBL	Q9Y6R0	1
ADRA2A	P08913	7	OAT	P04181	1
ADORA1	P30542	7	OSTC	Q9NRP0	1
YWHAZ	P63104	6	P2RX7	Q99572	1
UCHL1	P09936	6	PAK3	O75914	1
UBC	P0CG48	6	PCBD1	P61457	1
STUB1	Q9UNE7	6	PDIA3	P30101	1
RELA	Q04206	6	PDIA4	P13667	1
NGFR	P08138	6	PDIA6	Q15084	1
NFKB1	P19838	6	PDZK1P1	A8MUH7	1
MDM2	Q00987	6	PGAM1	P18669	1
JUN	P05412	6	PI4K2A	Q9BTU6	1
HSPB1	P04792	6	PITRM1	Q5JRX3	1
HSPA8	P11142	6	PLD1	Q13393	1
HSPA5	P11021	6	PLG	P00747	1
HSPA1A	P08107	6	PPIA	P62937	1
HSP90AB1	P08238	6	PPIB	P23284	1
HSP90AA1	P07900	6	PPID	Q08752	1
GRB2	P62993	6	PPP1R2	P41236	1
GAPDH	P04406	6	PREP	P48147	1
COPS5	Q92905	6	PRNP	P04156	1
CASP3	P42574	6	PRSS1	P07477	1
ARHGEF12	Q9NZN5	6	PRSS2	P07478	1
ADRBK1	P25098	6	PRSS3	P35030	1
ADCY9	O60503	6	PRSSL1	PRSSL1	1
ADCY8	P40145	6	RELN	P78509	1

ADCY7	P51828	6	RNF32	Q9H0A6	1
ADCY6	O43306	6	SEC22C	Q9BRL7	1
ADCY5	O95622	6	SERPINA3	P01011	1
ADCY4	Q8NFM4	6	SLC40A1	Q9NP59	1
ADCY3	O60266	6	SMUG1	Q53HV7	1
ADCY2	Q08462	6	SNX17	Q15036	1
ADCY1	Q08828	6	SORL1	Q92673	1
TRIO	O75962	5	SPARCL1	Q14515	1
PLCB4	Q15147	5	SPON1	Q9HCB6	1
KALRN	O60229	5	SRGAP3	O43295	1
GRK5	P34947	5	SSPN	Q14714	1
DAB2	P98082	5	STX5	Q13190	1
CDK1	P06493	5	STXBP1	P61764	1
ARHGEF25	Q86VW2	5	SUGT1	Q9Y2Z0	1
**APLP2**	Q06481	**5**	SYNRG	Q9UMZ2	1
ABL1	P00519	5	SYVN1	Q86TM6	1
TGM2	P21980	4	TAF9	Q16594	1
STXBP1	P61764	4	TBC1D7	Q9P0N9	1
SNCA	P37840	4	TGFB1	P01137	1
SHC1	P29353	4	TGFB2	P61812	1
SET	Q01105	4	THBS1	P07996	1
PSEN1	P49768	4	TM2D1	Q9BX74	1
PIN1	Q13526	4	TMCC2	O75069	1
PDIA3	P30101	4	TMEFF2	Q9UIK5	1
PARK2	O60260	4	TMEM191B	P0C7N4	1
NSF	P46459	4	TMEM191C	A6NGB0	1
NOTCH1	P46531	4	TMEM30A	Q9NV96	1
MAPT	P10636	4	TMEM30B	Q3MIR4	1
MAPK8	P45983	4	TMPRSS12	Q86WS5	1
MAPK1	P28482	4	TNFRSF21	O75509	1
HSPD1	P10809	4	TOMM5	Q8N4H5	1
HMGB1	P09429	4	TP53BP2	Q13625	1
GSN	P06396	4	TPD52L2	O43399	1
GSK3B	P49841	4	TSPAN6	O43657	1
CD81	P60033	4	TXNIP	Q9H3M7	1
CASP1	P29466	4	ADRA1A	P35348	2
BCAP31	P51572	4	ADRBK1	P25098	2
ACTB	P60709	4	AGT	P01019	2
XIAP	P98170	3	AGTR1	P30556	2
UNG	P13051	3	AGTR2	P50052	2
TUBB	P07437	3	ANXA1	P04083	2
TNF	P01375	3	APBB1	O00213	2
SYVN1	Q86TM6	3	ARHGEF25	Q86VW2	2
RCN2	Q14257	3	AVPR1A	P37288	2
PSEN2	P49810	3	BDKRB1	P46663	2
MYH9	P35579	3	BDKRB2	P30411	2
MME	P08473	3	CCKBR	P32239	2
MAPK3	P27361	3	CHRM3	P20309	2
MAP3K5	Q99683	3	CHRM5	P08912	2
KAT5	Q92993	3	COL1A2	P08123	2
ITGB5	P18084	3	EDNRA	P25101	2
ITGB1	P05556	3	EDNRB	P24530	2
HSPA4	P34932	3	F2R	P25116	2
GFAP	P14136	3	F2RL1	P55085	2
FOS	P01100	3	F2RL2	O00254	2
DDB1	Q16531	3	F2RL3	Q96RI0	2
CSNK2A1	P68400	3	FPR2	P25090	2
CRYAB	P02511	3	FTL	P02792	2
COL1A2	P08123	3	GHRL	Q9UBU3	2

CLU	P10909	3	GHSR	Q92847	2
CDC37	Q16543	3	GNA11	P29992	2
CBFB	Q13951	3	GNA14	O95837	2
CAV1	Q03135	3	GNA15	P30679	2
CASP6	P55212	3	GNAO1	P09471	2
CANX	P27824	3	GNAQ	P50148	2
CALR	P27797	3	GNB1	P62873	2
BRCA1	P38398	3	GNG2	P59768	2
ATF6	P18850	3	GNRH1	P01148	2
APLP1	P51693	3	GNRHR	P30968	2
ADAM17	P78536	3	GNRHR2	Q96P88	2
TTR	P02766	2	GPR4	P46093	2
TP53BP2	Q13625	2	GPR65	Q8IYL9	2
TAF9	Q16594	2	GPR68	Q15743	2
TAF15	Q92804	2	GPRC6A	Q5T6X5	2
STX5	Q13190	2	GRK5	P34947	2
SPTAN1	Q13813	2	GRP	P07492	2
RTN4R	Q9BZR6	2	GRPR	P30550	2
RPN2	P04844	2	GSN	P06396	2
RANBP9	Q96S59	2	HRH1	P35367	2
PRNP	P04156	2	HTR2A	P28223	2
PPIB	P23284	2	KALRN	O60229	2
PPIA	P62937	2	LPAR1	Q92633	2
PLD1	Q13393	2	LPAR3	Q9UBY5	2
PDIA4	P13667	2	LPAR4	Q99677	2
NID1	P14543	2	LPAR6	P43657	2
NFKB2	Q00653	2	LTB4R2	Q9NPC1	2
NCSTN	Q92542	2	MCHR1	Q99705	2
NAE1	Q13564	2	MCHR2	Q969V1	2
MAPK8IP1	Q9UQF2	2	NMB	P08949	2
MAPK10	P53779	2	NMBR	P28336	2
MAP3K11	Q16584	2	NMU	P48645	2
LINGO1	Q96FE5	2	NMUR1	Q9HB89	2
HYOU1	Q9Y4L1	2	NMUR2	Q9GZQ4	2
HSPG2	P98160	2	NOTCH1	P46531	2
HSPB8	Q9UJY1	2	NPFFR1	Q9GZQ6	2
HGS	O14964	2	NPFFR2	Q9Y5X5	2
GTF2F2	P13984	2	NPSR1	Q6W5P4	2
GNAO1	P09471	2	NTSR2	O95665	2
GANAB	Q14697	2	OPN4	Q9UHM6	2
FTL	P02792	2	P2RY11	Q96G91	2
FLOT1	O75955	2	P2RY1	P47900	2
FBLN1	P23142	2	PIK3CA	P42336	2
CREB3	O43889	2	PIK3CG	P48736	2
CREB1	P16220	2	PIK3R1	P27986	2
CPEB1	Q9BZB8	2	PIK3R2	O00459	2
COL25A1	Q9BXS0	2	PIK3R3	Q92569	2
CDK5	Q00535	2	PLCB3	Q01970	2
CD99	P14209	2	PLCB4	Q15147	2
CASP4	P49662	2	PMCH	P20382	2
BCAP29	Q9UHQ4	2	PROK1	P58294	2
BACE1	P56817	2	PROK2	Q9HC23	2
APOA1	P02647	2	PROKR1	Q8TCW9	2
APCS	P02743	2	PROKR2	Q8NFJ6	2
APBB1	O00213	2	PTAFR	P25105	2
APBA2	Q99767	2	PTGFR	P43088	2
APBA1	Q02410	2	TRH	P20396	2
AGRN	O00468	2	TRIO	O75962	2
ADAM10	O14672	2	UTS2	O95399	2

ACHE	P22303	2	UTS2D	Q765I0	2
A2M	P01023	2	ADCY1	Q08828	3
TXNIP	Q9H3M7	1	ADCY2	Q08462	3
TSPAN6	O43657	1	ADCY3	O60266	3
TPD52L2	O43399	1	ADCY4	Q8NFM4	3
TOMM5	Q8N4H5	1	ADCY5	O95622	3
TNFRSF21	O75509	1	ADCY6	O43306	3
TMPRSS12	TMPRSS12	1	ADCY7	P51828	3
TMEM30B	Q3MIR4	1	ADCY8	P40145	3
TMEM30A	Q9NV96	1	ADCY9	O60503	3
TMEM191C	TMEM191C	1	ADORA1	P30542	3
TMEM191B	TMEM191B	1	ADORA2A	P29274	3
TMEFF2	Q9UIK5	1	ADORA3	P33765	3
TMCC2	O75069	1	ADRA2A	P08913	3
TM2D1	Q9BX74	1	APLN	Q9ULZ1	3
THBS1	P07996	1	APLNR	P35414	3
TGFB2	P61812	1	ARHGEF12	Q9NZN5	3
TGFB1	P01137	1	ARHGEF1	Q92888	3
TBC1D7	Q9P0N9	1	C3	P01024	3
SYNRG	Q9UMZ2	1	C3AR1	Q16581	3
SUGT1	Q9Y2Z0	1	C5	P01031	3
SSPN	Q14714	1	CCL20	P78556	3
SRGAP3	O43295	1	CCL21	O00585	3
SPON1	Q9HCB6	1	CCL27	Q9Y4X3	3
SPARCL1	Q14515	1	CCL28	Q9NRJ3	3
SORL1	Q92673	1	CCL5	P13501	3
SNX17	Q15036	1	CCR10	P46092	3
SMUG1	Q53HV7	1	CCR2	P41597	3
SLC40A1	Q9NP59	1	CCR7	P32248	3
SERPINA3	P01011	1	CHRM2	P08172	3
SEC22C	Q9BRL7	1	CNR1	P21554	3
RNF32	Q9H0A6	1	CXCL10	P02778	3
RELN	P78509	1	CXCL12	P48061	3
PRSSL1	PRSSL1	1	CXCL16	Q9H2A7	3
PRSS3	P35030	1	CXCL9	Q07325	3
PRSS2	P07478	1	CXCR3	P49682	3
PRSS1	P07477	1	CXCR4	P61073	3
PREP	P48147	1	CXCR7	P25106	3
PPP1R2	P41236	1	DRD3	P35462	3
PPID	Q08752	1	FPR3	P25089	3
PLG	P00747	1	GABBR1	Q9UBS5	3
PITRM1	Q5JRX3	1	GABBR2	O75899	3
PI4K2A	Q9BTU6	1	GALR1	P47211	3
PGAM1	P18669	1	GNA12	Q03113	3
PDZK1P1	PDZK1P1	1	GNA13	Q14344	3
PDIA6	Q15084	1	GNAI1	P63096	3
PCBD1	P61457	1	GNAI2	P04899	3
PAK3	O75914	1	GNAI3	P08754	3
P2RX7	Q99572	1	GNAZ	P19086	3
OSTC	Q9NRP0	1	GPER	Q99527	3
OAT	P04181	1	GPR18	Q14330	3
NUMBL	Q9Y6R0	1	GPR44	GPR44	3
NUMB	P49757	1	GPR55	Q9Y2T6	3
NOTCH2	Q04721	1	HCAR2	Q8TDS4	3
NLRP3	Q96P20	1	HEBP1	Q9NRV9	3
NFASC	O94856	1	HRH3	Q9Y5N1	3
NF1	P21359	1	HRH4	Q9H3N8	3
NEFL	P07196	1	HTR5A	P47898	3
NECAB3	Q96P71	1	LPAR2	Q9HBW0	3

NCAM1	P13591	1	MTNR1A	P48039	3
MMP9	P14780	1	NPB	Q8NG41	3
MMP2	P08253	1	NPBWR1	P48145	3
MMP17	Q9ULZ9	1	NPW	Q8N729	3
MMP14	P50281	1	NPY1R	P25929	3
MMEL1	Q495T6	1	NPY5R	Q15761	3
MEFV	O15553	1	OPRK1	P41145	3
MED12	Q93074	1	OXER1	Q8TDS5	3
MBP	P02686	1	P2RY12	Q9H244	3
MAT1A	Q00266	1	P2RY13	Q9BPV8	3
MAST1	Q9Y2H9	1	P2RY14	Q15391	3
MAPK8IP2	Q13387	1	PDYN	P01213	3
LYPD3	O95274	1	PENK	P01210	3
LYL1	P12980	1	PLCB1	Q9NQ66	3
LRP8	Q14114	1	PLCB2	Q00722	3
LRP1B	Q9NZR2	1	POMC	P01189	3
LRP1	Q07954	1	PTGER3	P43115	3
LDLRAP1	Q5SW96	1	PYY	P10082	3
LAMA1	P25391	1	RLN3	Q8WXF3	3
L1CAM	P32004	1	RXFP3	Q9NSD7	3
KLK6	Q92876	1	RXFP4	Q8TDU9	3
KLK2	P20151	1	S1PR1	P21453	3
KLC1	Q07866	1	S1PR2	O95136	3
KIAA1704	Q8IXQ4	1	S1PR3	Q99500	3
KIAA0319L	Q8IZA0	1	S1PR5	Q9H228	3
ITM2B	Q9Y287	1	SSTR2	P30874	3
ITM2A	O43736	1	SSTR3	P32745	3
IFIT3	O14879	1	ABL1	P00519	4
IDE	P14735	1	ACHE	P22303	4
HTRA2	O43464	1	ACTB	P60709	4
HTRA1	Q92743	1	APCS	P02743	4
HSPB6	O14558	1	APLP1	P51693	4
HSP90B1	P14625	1	**APLP2**	Q06481	**4**
HSD17B10	Q99714	1	BCAP29	Q9UHQ4	4
HOMER2	Q9NSB8	1	BCAP31	P51572	4
HMOX2	P30519	1	BRCA1	P38398	4
HMOX1	P09601	1	CASP1	P29466	4
HADHB	P55084	1	CASP3	P42574	4
HADH	Q16836	1	CASP4	P49662	4
GSK3A	P49840	1	CASP6	P55212	4
GPNMB	Q14956	1	CDC37	Q16543	4
GPC1	P35052	1	CDK1	P06493	4
FLOT2	Q14254	1	COL25A1	Q9BXS0	4
FKBP1A	P62942	1	COPS5	Q92905	4
FANCM	Q8IYD8	1	CPEB1	Q9BZB8	4
FAM134A	Q8NC44	1	CREB1	P16220	4
F7	P08709	1	CREB3	O43889	4
F12	P00748	1	CRYAB	P02511	4
F10	P00742	1	CSNK2A1	P68400	4
EXOC6	Q8TAG9	1	DAB2	P98082	4
ERP44	Q9BS26	1	DDB1	Q16531	4
EPB41L3	Q9Y2J2	1	FLOT1	O75955	4
EPB41	P11171	1	FOS	P01100	4
ECE1	P42892	1	GAPDH	P04406	4
DPEP1	P16444	1	GFAP	P14136	4
DNM1	Q05193	1	GRB2	P62993	4
DNAH1	Q9P2D7	1	GSK3B	P49841	4
DLG4	P78352	1	GTF2F2	P13984	4
DAB1	O75553	1	HMGB1	P09429	4

CTSD	P07339	1	HSP90AA1	P07900	4
CTSB	P07858	1	HSP90AB1	P08238	4
CST3	P01034	1	HSPA1A	P08107	4
CPE	P16870	1	HSPA4	P34932	4
COL4A6	Q14031	1	HSPA8	P11142	4
COL4A5	P29400	1	HSPB1	P04792	4
COL4A3	Q01955	1	HSPB8	Q9UJY1	4
COL4A2	P08572	1	HSPD1	P10809	4
COL4A1	P02462	1	HYOU1	Q9Y4L1	4
COL18A1	P39060	1	JUN	P05412	4
CNTN4	Q8IWV2	1	KAT5	Q92993	4
CNTN3	Q9P232	1	LINGO1	Q96FE5	4
CNTN2	Q02246	1	MAP3K11	Q16584	4
CNTN1	Q12860	1	MAP3K5	Q99683	4
CLSTN3	Q9BQT9	1	MAPK10	P53779	4
CLSTN1	O94985	1	MAPK1	P28482	4
CLPTM1L	Q96KA5	1	MAPK3	P27361	4
CHRNB1	P11230	1	MAPK8	P45983	4
CHRNA7	P36544	1	MAPK8IP1	Q9UQF2	4
CD36	P16671	1	MAPT	P10636	4
CD14	P08571	1	MDM2	Q00987	4
CCHCR1	Q8TD31	1	MME	P08473	4
CAT	P04040	1	MYH9	P35579	4
CASP8	Q14790	1	NCSTN	Q92542	4
CAPZA2	P47755	1	NFKB1	P19838	4
CAPZA1	P52907	1	NGFR	P08138	4
CAMLG	P49069	1	PARK2	O60260	4
CALU	O43852	1	PIN1	Q13526	4
BNIP2	Q12982	1	PSEN1	P49768	4
BLMH	Q13867	1	PSEN2	P49810	4
BGN	P21810	1	RANBP9	Q96S59	4
BACE2	Q9Y5Z0	1	RCN2	Q14257	4
APPBP2	Q92624	1	RELA	Q04206	4
APOE	P02649	1	RPN2	P04844	4
APOA2	P02652	1	RTN4R	Q9BZR6	4
APMAP	Q9HDC9	1	SET	Q01105	4
APEH	P13798	1	SHC1	P29353	4
APBB3	O95704	1	SNCA	P37840	4
APBB2	Q92870	1	SPTAN1	Q13813	4
APBA3	O96018	1	STUB1	Q9UNE7	4
AP1M2	Q9Y6Q5	1	TAF15	Q92804	4
ALB	P02768	1	TGM2	P21980	4
AGER	Q15109	1	TNF	P01375	4
ADNP	Q9H2P0	1	TTR	P02766	4
ADAM9	Q13443	1	TUBB	P07437	4
ADAM8	P78325	1	UBC	P0CG48	4
ADAM33	Q9BZ11	1	UCHL1	P09936	4
ACE	P12821	1	UNG	P13051	4
ACBD3	Q9H3P7	1	XIAP	P98170	4
ABCB1	P08183	1	YWHAZ	P63104	4
