# Supplementary material for: Amyloid precursor protein interaction network in human testis: sentinel proteins for male reproduction
Source: BMC Bioinformatics. 2015 Jan 16;16(1):12. doi: 10.1186/s12859-014-0432-9 (PMC4384327; doi:10.1186/s12859-014-0432-9)
Supplement: Additional file 6: Table S5 — (a). Top 1000 rank of topological properties of proteins in extended APP/APLP2 network. Note that proteins with clustering coefficient 0 are neglected. [file 12859_2014_432_MOESM6_ESM.pdf]

Table S5 Topological properties of proteins in extended APP/APLP2 network. (a) Top 1000 rank of degree, clustering coefficient, betweenness centrality, and closeness centrality. Note that proteins with clustering coefficient 0 are neglected. (b) *k*-core and community that proteins are involved. The number in the community column is only index of the community. (c) Topological properties of APP partners identified by YTH in the extended APP/APLP2 network. Proteins with prominent topological properties are indicated with purple color.

(a)

Rank	Degree		Clustering coefficient		Betweenness centrality		Closeness centrality	
1	UBC	754	CTSS	0.000816	UBC	0.34864	UBC	0.572027
2	**APP**	**455**	UBC	0.005453	**APP**	**0.198072**	**APP**	**0.545418**
3	CDK1	335	STXBP1	0.00559	CASP3	0.088527	CASP3	0.470467
4	CASP3	320	NSF	0.005634	CDK1	0.06678	CDK1	0.469093
5	KIAA0101	264	SLX4	0.005848	COPS5	0.045504	EGFR	0.46518
6	PIK3CG	231	PRKAB1	0.006667	JUN	0.04443	KIAA0101	0.453821
7	PLCB3	220	HPRT1	0.008333	KIAA0101	0.035582	GAPDH	0.445241
8	EP300	215	C14orf1	0.008658	EGFR	0.031648	JUN	0.444806
9	ANXA1	213	RCN2	0.008658	PIK3CA	0.029848	FYN	0.444011
10	JUN	206	CASP3	0.009032	GAPDH	0.028337	HSPA5	0.442358
11	PIK3CA	202	UNC119	0.009447	PLCB3	0.028294	HSPB1	0.442286
12	EGFR	199	HLA-B	0.009967	ANXA1	0.02588	ANXA1	0.441928
13	FYN	161	IQCB1	0.010526	EP300	0.025344	COPS5	0.438946
14	COPS5	152	FBLN1	0.010695	HSPA1A	0.02418	HSPA1A	0.43719
15	STAT1	151	ATXN1	0.011696	HSPA5	0.024011	EP300	0.43691
16	CALM1	149	**APP**	**0.013022**	FYN	0.023752	BRCA1	0.43524
17	LPAR2	145	CALM1	0.013332	STAT1	0.023122	STAT1	0.432826
18	SUMO2	143	FAF1	0.014113	PIK3CG	0.020136	UBE2I	0.431937
19	BRCA1	141	COPS5	0.014291	NSF	0.019149	PIK3CA	0.430033
20	RLN3	140	ANK2	0.014706	STXBP1	0.018028	YWHAZ	0.42601
21	HSPA1A	134	GABARAPL2	0.014706	STUB1	0.017359	RELA	0.422976
22	CCNB1	132	GSTP1	0.014706	BCAP31	0.016407	VIM	0.422583
23	PSME3	129	USP2	0.014706	CALM1	0.015773	NGFR	0.421475
24	HSPA5	129	ULK2	0.015054	PDIA3	0.015423	STUB1	0.420114
25	GAPDH	128	SDHA	0.015152	HSPB1	0.014827	HSP90AA1	0.419985
26	CBL	128	UBE2S	0.015152	CBL	0.014601	GRB2	0.419598
27	CDC25A	125	GABARAP	0.016327	NGFR	0.013709	SUMO2	0.419404
28	PSMD6	124	UBE2U	0.016667	BRCA1	0.011485	IKBKB	0.417993
29	MNAT1	124	GAPDH	0.016855	VIM	0.011332	CDH1	0.416845
30	GNA15	121	LRPAP1	0.017544	UBE2I	0.009664	BCAP31	0.416845
31	IKBKB	117	RNF10	0.017544	IKBKB	0.009049	DAB2	0.415893
32	VIM	116	ACAT2	0.018182	SUMO2	0.008148	ABL1	0.41583
33	PSMA5	116	GET4	0.018182	CLTC	0.007582	PSME3	0.414756
34	PSMD10	111	TEX10	0.018182	SYVN1	0.006949	SIRT1	0.413877
35	CHUK	111	MYC	0.018692	GNA15	0.006738	CCNB1	0.413877
36	STUB1	109	VIM	0.01904	GRB2	0.006699	HSP90AB1	0.413001
37	PSMD8	109	HSPA5	0.019259	PSME3	0.006655	PARP1	0.412191
38	MAPRE1	109	JUN	0.019512	DAB2	0.00653	CBL	0.412004
39	MYC	108	MAP1LC3A	0.019512	CHUK	0.006439	PLCB3	0.411694
40	UBE2I	106	MAP1LC3B	0.019934	YWHAZ	0.00626	CLTC	0.411632
41	PAFAH1B1	106	ESR2	0.02	FBLN1	0.006178	NFKB1	0.41157
42	PSMB8	105	PDIA3	0.020103	CCNB1	0.005827	KPNB1	0.410889
43	ADORA3	105	STUB1	0.020217	MAPRE1	0.005401	UCHL1	0.410149
44	CCL27	104	GABARAPL1	0.020362	CD81	0.005357	MDM2	0.409595
45	BUB1B	103	IKBKE	0.020456	STAT2	0.005326	TRIM28	0.409227
46	CLTC	98	SUMO2	0.020684	SIRT1	0.005027	CDC25A	0.409166
47	PSMA8	95	BIN1	0.021645	CDC25A	0.005026	ACTB	0.408737
48	DYNC1H1	95	SH3GLB1	0.021645	MYC	0.004948	CCNA2	0.408066
49	NEK2	94	CSTB	0.021978	FLNA	0.004862	TP53	0.407154
50	PDIA3	93	SEPT2	0.021978	MNAT1	0.004236	YWHAQ	0.407093
51	HDAC2	91	TNIK	0.021978	CREB1	0.004077	DDX5	0.407032
52	PROK1	90	VCAN	0.021978	RUVBL1	0.004066	CALM1	0.407032
53	EDN1	90	COL6A1	0.022222	KPNB1	0.00406	CHUK	0.406185
54	CKS1B	87	DYNLL2	0.022222	RARA	0.003998	MCM5	0.406125
55	TUBG1	86	NEK6	0.022222	HDAC2	0.003951	MAPRE1	0.406064

56	RUVBL1	86	PCBD1	0.022222	DYNC1H1	0.003877	MAPK1	0.405642
57	NGFR	86	PPM1G	0.022222	CDH1	0.003838	DYNC1H1	0.405522
58	BCAP31	85	PRKAA1	0.022222	PARP1	0.003824	LMNA	0.404561
59	HSPB1	84	SNCG	0.022222	TRIM28	0.003696	ESR1	0.403843
60	PARP1	80	HSPA1A	0.023005	TP53	0.003685	RARA	0.403426
61	MCM7	78	BCAP31	0.023249	RIF1	0.003427	FLNA	0.403366
62	TP53	74	KIAA0101	0.023707	PSMD6	0.003341	HSPA8	0.403247
63	PCNT	74	VHL	0.024823	UCHL1	0.003108	KPNA2	0.40295
64	KPNB1	74	KLK5	0.025641	TERF2	0.003025	HSPA4	0.40289
65	FLNA	74	RPS6KB2	0.025641	RCN2	0.002954	BCAR1	0.40289
66	PLK4	72	UBE2D2	0.025641	EWSR1	0.002947	MAPT	0.402001
67	CEP76	72	MSN	0.025806	PSMA5	0.002936	PDIA3	0.401765
68	STAT2	71	ST13	0.026316	KPNA2	0.002841	TUBB	0.40147
69	NSF	71	NGFR	0.026539	PDGFRB	0.002772	MNAT1	0.401352
70	GMNN	71	SIAH1	0.026578	RLN3	0.002729	SET	0.40088
71	XRCC5	70	FLNA	0.026657	PSMB8	0.002719	CTNNB1	0.40088
72	TERF2	70	STAT1	0.027461	DDX5	0.002667	BCL6	0.40088
73	SIRT1	70	PRKAR1A	0.027778	BCAR1	0.002607	PSMA5	0.400234
74	ODF2	70	RIBC2	0.027778	TBK1	0.002581	ELF3	0.399941
75	CEP78	70	SKIL	0.027778	TUBG1	0.002559	SYVN1	0.399707
76	RARA	68	SSFA2	0.027778	ESR1	0.002555	EWSR1	0.399649
77	ITGB3BP	68	TPD52L1	0.027778	ABL1	0.002553	TOP2A	0.39889
78	YWHAZ	67	TRADD	0.027778	GSK3B	0.002547	NSF	0.398716
79	GRB2	67	EP300	0.028168	RELA	0.002502	PSMD6	0.398658
80	CDH1	65	MCC	0.028226	PAFAH1B1	0.002495	PSMB8	0.398077
81	CCNA1	65	CD81	0.02849	BCL6	0.002466	BRCA2	0.398077
82	U2AF2	64	USP11	0.02849	ITGB3BP	0.002444	MYC	0.397961
83	TRIM28	64	LLGL1	0.028571	UIMC1	0.002325	HSPD1	0.397961
84	RBM8A	64	MME	0.028571	LPAR2	0.002296	RBL1	0.397613
85	MCM5	63	TRAF6	0.028571	MDM2	0.00229	PDGFRB	0.397555
86	RPA3	62	WDR61	0.028571	HSP90AA1	0.002278	BUB1B	0.397266
87	KPNA2	62	EGFR	0.02873	GABARAPL1	0.002273	GSK3B	0.397093
88	ESR1	62	UBXN7	0.028986	MCM5	0.002247	TFAP2A	0.397035
89	POLR2D	61	FYN	0.029736	YWHAQ	0.00214	HDAC2	0.396517
90	PDGFRB	61	CDK16	0.030303	RBL1	0.002114	TBK1	0.396344
91	TBK1	60	LRIF1	0.030303	BUB1B	0.002101	STAT2	0.396344
92	SNRPD1	60	MAGEB2	0.030303	XRCC5	0.002075	UBB	0.396172
93	RBBP4	60	EWSR1	0.03156	IRF3	0.002034	STXBP1	0.396057
94	DDX5	60	DAB2	0.032717	NAE1	0.002019	RBBP4	0.395827
95	SNRPD3	59	EGR1	0.032717	PSMD10	0.00198	PIK3R1	0.395827
96	IKBKE	59	TSC22D1	0.032967	GMNN	0.001931	CFLAR	0.39577
97	EWSR1	59	EIF1B	0.033333	MAPK14	0.001922	RAD51	0.395655
98	SNRNP200	58	TNFRSF1B	0.033333	ALB	0.001887	RUVBL1	0.395598
99	NUP107	57	HSPB1	0.033563	MCM7	0.001879	XRCC5	0.395541
100	SNRPF	56	RIF1	0.034574	PSMD8	0.001843	TUBG1	0.395255
101	MCM4	56	LGALS3BP	0.034632	CTNNB1	0.00184	SMARCC1	0.395197
102	SUMO1	55	CBL	0.035064	CCNA1	0.00184	PAFAH1B1	0.395026
103	STXBP1	54	STK11	0.035573	NEK2	0.001806	PIAS2	0.394741
104	SF3A2	54	DDX5	0.035593	UBB	0.001803	HSF1	0.394741
105	RAE1	53	BAG6	0.035714	PCNT	0.001798	CSNK2A1	0.394627
106	INSL3	53	DRG1	0.035714	SRC	0.001784	AR	0.394456
107	PLK1	52	EXOC7	0.035714	CCNA2	0.001764	SUMO1	0.394114
108	GABARAPL1	52	MAX	0.035714	SIAH1	0.001696	MAPK8	0.394114
109	RBL1	51	MMP2	0.035714	CD82	0.001694	UNG	0.394057
110	RAN	51	NDRG1	0.035714	HSP90AB1	0.001676	SLC9A3R1	0.394057
111	CENPA	51	PRKCG	0.035714	UBA52	0.001672	RACGAP1	0.393944
112	TRAF6	50	SSX2IP	0.035714	SUMO1	0.001671	CD99	0.39383
113	TCP1	50	TRAF3IP1	0.035714	HSPA8	0.001647	RIF1	0.393717
114	NUDC	50	WDR62	0.035714	YWHAG	0.001616	MCM7	0.39366
115	GABARAP	50	HDGF	0.035985	RAN	0.001599	TMPO	0.393603
116	CTSS	50	PIK3CA	0.036156	TP63	0.001592	PCNT	0.393377
117	CCNA2	50	CDK6	0.036364	ARHGEF11	0.001587	MTA1	0.39315
118	UBB	49	SVIL	0.036364	CD44	0.00156	MAPK14	0.39315
119	UBA52	49	TK1	0.036364	RBBP4	0.001551	CD81	0.393037
120	PRPF8	49	YWHAZ	0.036635	NFKB1	0.00155	TERF2	0.392924
121	VHL	48	NASP	0.036765	PIK3R1	0.001548	PSMD10	0.392924

122	RIF1	48	STAT2	0.037022	DNMT1	0.001517	GMNN	0.392867
123	RELA	48	CSNK2B	0.038095	APOA1	0.001517	SPTAN1	0.392811
124	HSP90AA1	48	XPO5	0.038095	ATF2	0.001515	DNMT1	0.392754
125	IRF3	47	IKBKG	0.038407	UNG	0.001514	RCN2	0.392529
126	ATF2	47	AKAP8L	0.038462	VHL	0.001513	PSMD8	0.392416
127	RPS27A	46	FBXO7	0.038462	RPL26L1	0.001492	PIK3CG	0.392416
128	HDAC5	46	RFWD3	0.038462	HSF1	0.001482	ARHGEF11	0.392416
129	HDAC1	46	SMAD3	0.03871	RPS27A	0.001468	MYH9	0.39236
130	CTNNB1	45	GRB2	0.038896	ECT2	0.001453	UBA52	0.392247
131	CDK6	45	SUMO1	0.039057	FAF1	0.001446	ITGB3BP	0.392078
132	CDC45	45	CLTC	0.039344	RBM8A	0.001425	NAE1	0.391797
133	CCT2	45	HSPH1	0.039886	TRAF6	0.001418	DCC	0.391516
134	BCAR1	45	PLCB3	0.039892	DIABLO	0.001404	AKT1	0.391404
135	TP63	44	MAP2K6	0.040244	RACGAP1	0.001401	CASP1	0.391068
136	SNRPA1	44	PIK3CG	0.040391	IKBKE	0.001373	UIMC1	0.390956
137	CPSF3	44	ARRB2	0.04086	YWHAE	0.001336	SSB	0.390788
138	SIAH1	43	CDK1	0.040897	UNC119	0.001334	RAN	0.390788
139	MAP1LC3B	43	CDC34	0.040936	PSMA8	0.001315	CREB1	0.390788
140	LMNA	43	IL7R	0.040936	RPA3	0.001294	RPS27A	0.390621
141	HLA-B	43	CHUK	0.041278	ODF2	0.001293	CKS1B	0.390509
142	CCT8	43	RUVBL1	0.041587	POLR2D	0.001278	ATF2	0.390453
143	YWHAQ	42	SYVN1	0.041667	LMNA	0.001262	PRKD1	0.390341
144	PRIM1	42	COPS6	0.042105	DCC	0.00125	FBL	0.390341
145	CREBBP	42	SAFB	0.042105	RAE1	0.001246	RAD23B	0.390174
146	CCT5	42	RARA	0.042142	VCAN	0.001241	PSMA8	0.390119
147	YWHAG	41	HDAC5	0.042512	GABARAP	0.001233	GFAP	0.390119
148	MAP2K6	41	ARRB1	0.043333	CEP76	0.001233	DDX1	0.390063
149	MAP1LC3A	41	SMAD2	0.043544	TCP1	0.001232	NBN	0.390007
150	PCNA	40	CREB1	0.043678	MAP2K6	0.001232	PIN1	0.389951
151	KIF2C	40	SMC4	0.043956	USP11	0.00123	GABARAPL1	0.389896
152	HSF1	40	SQSTM1	0.043956	HLA-B	0.001229	RAE1	0.389729
153	CDKN1A	40	VCP	0.044118	MYB	0.00122	IRF3	0.389507
154	CDK2	40	FSCN1	0.044335	RNF139	0.001209	MSH6	0.389451
155	BUB1	40	WDR5	0.044444	COIL	0.00118	SRC	0.389396
156	UNC119	39	COIL	0.044974	SLC9A3R1	0.001157	RPA3	0.389396
157	SMARCC1	39	A2M	0.045455	EGR1	0.00114	NEK2	0.389396
158	SGOL2	39	ARHGDIA	0.045455	CFLAR	0.001138	YWHAG	0.38934
159	RANBP2	39	CKAP4	0.045455	PLK4	0.001131	CD44	0.389285
160	PRMT5	39	ITCH	0.045455	U2AF2	0.001124	HMGB1	0.389229
161	NUP155	39	MAGED1	0.045455	RAD23B	0.001124	HSPA2	0.389063
162	DNMT1	39	MAPK3	0.045455	ELF3	0.001103	DDB1	0.389008
163	BCL6	39	MVP	0.045455	IKBKG	0.001101	MAPKAPK5	0.388731
164	BARD1	39	TGFBR1	0.045455	HDAC1	0.001099	CSE1L	0.388675
165	SRC	38	TRIP13	0.045455	BRD7	0.001099	PTGES3	0.38862
166	PIAS2	38	DTNBP1	0.045752	CDK6	0.001096	PARK7	0.38862
167	NUP62	38	CPSF6	0.047368	SMAD2	0.001094	CHAF1B	0.388454
168	IKBKG	38	TP63	0.047569	PCBD1	0.001085	KAT5	0.388234
169	EMD	38	ATXN3	0.047619	CEP78	0.001085	NUDC	0.388123
170	EGR1	38	BCR	0.047619	SSR1	0.001081	PARK2	0.388068
171	DBF4	38	DDX19B	0.047619	CKS1B	0.001076	CEP76	0.388068
172	DAB2	38	FAM48A	0.047619	CD99	0.001072	NOTCH1	0.387903
173	YWHAE	37	GADD45G	0.047619	MSN	0.00107	MYB	0.387903
174	XPO1	37	HUWE1	0.047619	PPP2R1B	0.001061	PLK4	0.387793
175	TOP2A	37	MEN1	0.047619	APH1B	0.00106	TARDBP	0.387683
176	SMAD2	37	MOBKL3	0.047619	PRKD1	0.001039	PRIM1	0.387518
177	RAD23B	37	MRPS22	0.047619	STX7	0.001027	YWHAE	0.387463
178	RACGAP1	37	PAICS	0.047619	TRAF2	0.001024	ODF2	0.387463
179	KIF18A	37	PPP2R2B	0.047619	CUL4B	0.001024	CEP78	0.387408
180	CDC20	37	RRBP1	0.047619	PRMT5	0.001014	BUB1	0.387353
181	CASC5	37	SMURF2	0.047619	CCL27	0.001014	NUP107	0.387078
182	SMC3	36	TBC1D15	0.047619	CD46	0.001001	TP63	0.386969
183	MYB	36	TCERG1	0.047619	MAGI1	0.000996	PRKCD	0.386914
184	H2AFX	36	UBE2H	0.047619	CDKN1A	0.000995	U2AF2	0.38675
185	DIABLO	36	UBE2N	0.047619	C3	0.000984	MCM4	0.386695
186	ARHGEF11	36	UFD1L	0.047619	PIAS2	0.000983	HIP1	0.386695
187	TAF10	35	XBP1	0.047619	UBE2K	0.000981	EMD	0.386531

188	NUP88	35	KPNA2	0.049709	MAPKAPK5	0.00098	FOS	0.386476
189	MSH2	35	SSR1	0.05	CSNK2A1	0.00098	APLP2	0.386094
190	MDM2	35	FRS3	0.050265	PROK1	0.000977	SIAH1	0.385985
191	GTF2H3	35	IKBKB	0.050398	ARRB2	0.000952	CDC37	0.385985
192	DAXX	35	ANXA1	0.050802	FXR2	0.00095	RBM8A	0.38593
193	CSTF3	35	TERF2	0.050932	EDN1	0.00094	MEF2D	0.38593
194	CENPN	35	AMFR	0.051282	MAPK1	0.000937	PPP2R1B	0.385712
195	CDCA8	35	CIAO1	0.051282	MAPK8	0.00093	FKBP4	0.385658
196	UCHL1	34	DNAJB6	0.051282	PARK7	0.000918	SNRPD3	0.385603
197	RAD51	34	NAE1	0.051282	FBL	0.000911	POT1	0.385331
198	KIF2B	34	USP7	0.052632	CCT2	0.000908	USP1	0.385168
199	FBLN1	34	UBE2I	0.05319	SF3A2	0.0009	DIABLO	0.385168
200	CD44	34	CDH1	0.053846	C14orf1	0.000892	TFDP2	0.385006
201	SYVN1	33	HDAC6	0.054545	VWF	0.000888	S100A11	0.385006
202	SUGP1	33	RAG1	0.054545	STK11	0.000887	CASP6	0.385006
203	RFC4	33	TSNAX	0.054545	IQCB1	0.000887	MAGI1	0.384897
204	PPP2R1B	33	PPP2R1B	0.054924	CASP6	0.000887	PRMT5	0.384843
205	NBN	33	WIPI2	0.054945	AKT1	0.000882	ECT2	0.384843
206	HDGF	33	UCHL1	0.055258	CDH2	0.00088	CCNB2	0.384735
207	BRCA2	33	CDK9	0.055336	KLK5	0.000879	IKBKG	0.38468
208	ABL1	33	MEPCE	0.055385	SMARCC1	0.000875	SNCA	0.384626
209	RPL26L1	32	AHSA1	0.055556	DAXX	0.000871	NUP88	0.384302
210	RB1	32	CASP4	0.055556	PRKCD	0.000868	HDAC1	0.384302
211	NFKB1	32	DIABLO	0.055556	AR	0.000866	RDX	0.384139
212	MCC	32	EIF2C4	0.055556	SMAD3	0.000858	CKS2	0.384031
213	GSK3B	32	ROCK1	0.055556	USP1	0.000849	PPP5C	0.38387
214	FBL	32	RRP12	0.055556	RNF10	0.000848	NUP62	0.383816
215	FAF1	32	TRIM28	0.055556	HSPH1	0.000848	NUP155	0.383762
216	ULK2	31	DDX1	0.056277	CCL5	0.000846	SNRPF	0.383654
217	SMAD3	31	CDH2	0.05665	STAM2	0.000845	POLR2D	0.383546
218	PSMA3	31	EMD	0.056899	TOP2A	0.000844	MAP1B	0.383546
219	PIK3R1	31	FXR2	0.057143	SNRNP200	0.000839	XIAP	0.383385
220	PARK7	31	HDAC2	0.057875	BRCA2	0.000838	UBE2K	0.383385
221	MSN	31	CD82	0.057895	COPS6	0.000837	NFKB2	0.383385
222	ECT2	31	ESR1	0.05817	CD14	0.000831	CBX3	0.383331
223	DDX20	31	EPB41	0.058333	ARRB1	0.000827	CCT8	0.383277
224	BUB3	31	KIAA1377	0.058333	ACTB	0.000818	SMC3	0.38317
225	ARRB2	31	MAP3K3	0.058462	PIK3R3	0.000811	GTF2E2	0.383116
226	ACTB	31	GSK3B	0.058468	TMPO	0.000803	DAXX	0.383009
227	ZWILCH	30	MAPK13	0.05848	BARD1	0.000801	STAM2	0.382955
228	UIMC1	30	CD44	0.058824	PRKCB	0.000799	SOX9	0.382955
229	SMN1	30	CDKN2C	0.058824	MCM4	0.000799	CDKN1A	0.382901
230	CREB1	30	PPP1CA	0.058824	PRPF8	0.000795	CASC5	0.382901
231	CFLAR	30	IRF3	0.059204	HLA-E	0.000786	TCP1	0.382848
232	BAG2	30	MAPK8	0.06	CCT5	0.000786	KIF11	0.382848
233	AR	30	DAZAP1	0.060606	GNB1	0.000767	BAG2	0.382848
234	STAM2	29	SSB	0.060847	STAT3	0.000766	HSPH1	0.38274
235	NUP35	29	MAP1B	0.061594	NUDC	0.000765	C14orf1	0.38274
236	HSP90AB1	29	RTN4	0.061905	PLK1	0.000763	NASP	0.382687
237	FSCN1	29	TP53	0.062199	CCT8	0.000757	SNRNP200	0.382633
238	ELF3	29	ILF2	0.062678	EMD	0.000755	TP53BP1	0.382579
239	CDH2	29	YWHAQ	0.062718	MAP1LC3B	0.000748	CENPN	0.382472
240	TUBA4A	28	XRCC5	0.06294	LRPAP1	0.00074	CDCA8	0.382419
241	TMPO	28	BRCA1	0.063121	TUBA4A	0.000733	LYN	0.382365
242	SSB	28	PIN1	0.063158	SMC3	0.000733	CCT2	0.382258
243	POT1	28	MDM2	0.063866	CALU	0.000732	PSEN1	0.381724
244	NFKBIE	28	ATN1	0.064103	RAD51	0.000725	AHR	0.381724
245	MSH6	28	CD99	0.064103	ITPR1	0.000722	FAF1	0.381618
246	MAD2L1	28	ETFA	0.064103	SNRPD1	0.000718	NFKBIA	0.381564
247	HSPA8	28	NSFL1C	0.064103	CSE1L	0.000718	TRAF6	0.381511
248	FRS3	28	YY1AP1	0.064103	TUBB	0.000699	CCT5	0.381458
249	CUL4B	28	PARP1	0.064241	POT1	0.000698	COIL	0.381351
250	COIL	28	H2AFX	0.065079	TFAP2A	0.000695	PRPF8	0.381138
251	CDK7	28	MAP3K1	0.065359	SH3GLB1	0.000695	EIF4G2	0.381085
252	BRD7	28	DCC	0.065527	CENPA	0.000695	CNOT7	0.381085
253	AURKB	28	HLA-C	0.065934	NUP107	0.000692	BARD1	0.380979

254	USP11	27	MPRIP	0.065934	ITGA3	0.000691	DHX30	0.380926
255	TRAF2	27	APOA1	0.066667	HERPUD1	0.000686	PBK	0.38082
256	PPP2R1A	27	BAG2	0.066667	BAG2	0.000678	VAV1	0.380554
257	MED26	27	BCL2	0.066667	HDAC5	0.000675	THAP11	0.380554
258	LYAR	27	BIRC2	0.066667	CDC45	0.000674	HMGA1	0.380554
259	ILF2	27	C7orf64	0.066667	SSB	0.00067	NSFL1C	0.380501
260	HSPH1	27	CUL3	0.066667	RTN4	0.000668	ATP2B4	0.380501
261	DHX9	27	DLC1	0.066667	SMAD4	0.000664	RABEP1	0.380448
262	DCC	27	DNAJB4	0.066667	TRRAP	0.000657	KIF2C	0.380448
263	CKAP5	27	EPAS1	0.066667	NBN	0.000657	TGM2	0.380342
264	CDKN1B	27	FN1	0.066667	PPP2R1A	0.000651	CDKN3	0.380184
265	CDC42	27	GOPC	0.066667	SNRPD3	0.00065	SGOL2	0.380025
266	CD81	27	ID2	0.066667	ATN1	0.000646	RTN4	0.379919
267	CCNE1	27	KIAA1683	0.066667	FSCN1	0.000644	DKC1	0.379867
268	UBE2K	26	LGALS1	0.066667	BNIP3L	0.000644	DGKE	0.379867
269	TRRAP	26	MLF2	0.066667	PCNA	0.000639	PML	0.379761
270	SP1	26	MPHOSPH9	0.066667	DDB1	0.000638	ILF2	0.379761
271	SLC9A3R1	26	PPM1B	0.066667	PPP2R4	0.000635	FXR2	0.379761
272	RPA2	26	PRNP	0.066667	IL7R	0.000634	KIF18A	0.379708
273	RAD21	26	PTEN	0.066667	UBXN7	0.000621	GSN	0.379708
274	PRKACA	26	RDX	0.066667	NFATC2	0.000617	SPAG5	0.379655
275	PPP5C	26	SNCA	0.066667	VAV1	0.000611	RFC4	0.379655
276	MTA1	26	TRIM37	0.066667	PTGES3	0.000603	CUL4B	0.379497
277	MEPCE	26	TSGA10	0.066667	CREBBP	0.000603	MAPK3	0.379392
278	MAP3K3	26	TTR	0.066667	CREM	0.000586	CAV1	0.379286
279	HDAC3	26	USP44	0.066667	CXCR4	0.000585	WNK3	0.379234
280	CUL1	26	VAMP2	0.066667	MSH2	0.000583	KRT7	0.379234
281	CLASP1	26	SIRT1	0.068737	TAF9	0.000582	LGALS3BP	0.379129
282	CCND1	26	BCL6	0.068826	MAP1LC3A	0.000582	PSEN2	0.378918
283	AHR	26	RELA	0.070035	MCF2	0.00058	RRP1B	0.378866
284	VWF	25	PRMT5	0.070175	FN1	0.000578	FSCN1	0.378866
285	UBE2D1	25	UBE2K	0.070769	DGKE	0.000573	MBD1	0.378761
286	TUBB	25	ECT2	0.070968	RANBP2	0.000566	SNRPD1	0.378708
287	SUPT5H	25	CDC37	0.071429	XPO1	0.000565	SH3GL3	0.378708
288	SSR1	25	L1CAM	0.071429	APC	0.000565	ARRB2	0.378656
289	SRRM2	25	LAMA4	0.071429	HUWE1	0.000563	VWF	0.378551
290	SKP1	25	LUC7L2	0.071429	MAPT	0.000562	ACLY	0.378551
291	RPA1	25	MKRN1	0.071429	CBX3	0.000562	DNAJB6	0.378498
292	PRKAB1	25	MYB	0.071429	COL6A1	0.000561	BRD7	0.378393
293	NDE1	25	PLSCR1	0.071429	GTF2F2	0.000559	CCND1	0.378341
294	MYBL2	25	RANBP9	0.071429	HSPA4	0.000558	STAT3	0.378184
295	MAPK8	25	RNF185	0.071429	H2AFX	0.000558	IKBKE	0.378184
296	MAPK1	25	MAD2L2	0.072727	GNA13	0.000551	LPAR2	0.378131
297	GTF2F1	25	TAB2	0.073529	DERL2	0.00055	ZWILCH	0.37787
298	ESR2	25	CTNNB1	0.073737	VCP	0.000548	TNF	0.37787
299	CSE1L	25	ELF3	0.073892	ULK2	0.000548	SF3A2	0.377818
300	CBX3	25	HSF1	0.074359	UBE2D1	0.000548	EPB41	0.377818
301	ATM	25	PRKD1	0.075099	LGALS3BP	0.000544	LYAR	0.377713
302	ARRB1	25	MYH9	0.075758	WDR61	0.000542	GTF2H3	0.377661
303	APH1B	25	PBK	0.075758	SNRPF	0.000541	VRK1	0.377609
304	UBXN7	24	TPX2	0.075758	MLH1	0.000541	FANCI	0.377609
305	STAT3	24	CFLAR	0.075862	RDX	0.000536	RPL26L1	0.377504
306	RANBP1	24	KPNB1	0.075898	ADORA3	0.000534	COPS6	0.377452
307	NUP153	24	HERPUD1	0.076023	SET	0.00053	KRT18	0.3774
308	MCF2	24	RNF139	0.076023	PRIM1	0.00053	CDK6	0.3774
309	MAP1B	24	CDKN2A	0.07619	AGT	0.00053	PTAFR	0.377348
310	GTF2F2	24	HIF1A	0.07619	SP1	0.000525	TAF15	0.377088
311	FANCI	24	ACLY	0.076923	PLCG1	0.000525	LGALS1	0.377088
312	CSNK2A1	24	LONP1	0.076923	PTAFR	0.000521	SAFB	0.376932
313	CHD3	24	PAXIP1	0.076923	LAMB2	0.000521	UTS2	0.37688
314	CCNH	24	PPP5C	0.076923	HGS	0.00052	GSTP1	0.37688
315	APC	24	ATF2	0.078631	ST13	0.000517	DDX20	0.37688
316	TP53BP1	23	DAXX	0.078992	PML	0.000516	TRRAP	0.376776
317	TFDP2	23	STAT3	0.07971	ATXN1	0.000514	CD82	0.376724
318	TFAP2A	23	RLN3	0.080267	LYAR	0.000512	TUBA4A	0.37662
319	STK11	23	CD46	0.080882	PRKACA	0.000507	SUPT5H	0.376568

320	SRSF1	23	SMARCC1	0.082321	MAP3K3	0.000503	SMAD2	0.376568
321	SMAD4	23	TRAF2	0.082621	RB1	0.000502	CDK5	0.376568
322	PTGES3	23	PTGES3	0.083004	SQSTM1	0.000498	SMU1	0.376464
323	PRKD1	23	AHR	0.083077	BUB1	0.000496	PYGL	0.376464
324	PPP2CA	23	HMGB1	0.083333	EPB41	0.000492	PIK3R2	0.376464
325	ORC1	23	HMGCS1	0.083333	LLGL1	0.000491	PCNA	0.376412
326	MAPK14	23	PDXK	0.083333	MAP1B	0.000488	TPX2	0.37636
327	HNRNPA1	23	RABEP1	0.083333	CDC42	0.000487	CASP8AP2	0.376309
328	GTF2E2	23	TNFRSF1A	0.083333	HIP1	0.000486	CASP4	0.376309
329	ERCC1	23	TRAF1	0.083333	PPP5C	0.000483	MSN	0.376205
330	DYNLL1	23	ARHGEF11	0.084127	KLC1	0.000483	CKAP4	0.376205
331	CREM	23	ABL1	0.085227	RRP1B	0.000478	NUP35	0.376153
332	CDK9	23	MAPK9	0.085714	RHOA	0.000469	MAP3K5	0.376153
333	CDC6	23	MSH2	0.085714	CDC37	0.000469	WTAP	0.375998
334	CDC27	23	HDAC3	0.086154	USP2	0.000464	PCBD1	0.375946
335	AKT1	23	GNA15	0.086777	TPD52L2	0.000464	IL7R	0.375894
336	USP1	22	BRD7	0.087302	SUPT5H	0.000459	RB1	0.375739
337	SPAG9	22	TMPO	0.087302	RBX1	0.000459	CPSF3	0.375739
338	SIKE1	22	TBK1	0.087571	LYN	0.000459	CXCR4	0.375688
339	SH3GLB1	22	RAF1	0.087912	CCHCR1	0.000459	S100A6	0.375636
340	RCN2	22	SRC	0.088193	MAGEH1	0.000451	STK11	0.375584
341	PPP2R4	22	NEDD8	0.088235	WDTC1	0.00045	HLA-E	0.375584
342	PPP1CC	22	CREBBP	0.088269	SH3GL3	0.000448	GNA15	0.375584
343	PML	22	BNIP3L	0.088889	SGOL2	0.000448	TSNAX	0.375533
344	NR3C1	22	CUL2	0.088889	HSPA2	0.000447	TOPBP1	0.375533
345	NFATC2	22	IRS1	0.088889	RFC4	0.000444	PEX5	0.375533
346	MEF2D	22	ITGB5	0.088889	FKBP4	0.000442	RECQL4	0.375481
347	LGALS3BP	22	LAMC1	0.088889	KIF11	0.000441	HPRT1	0.375326
348	HSPA4	22	PRKCZ	0.088889	GTF2H3	0.00044	TAF9	0.375275
349	FZR1	22	RANBP3	0.088889	MTA1	0.000439	PPP2R1A	0.375223
350	E2F1	22	RUNX1	0.088889	F2RL1	0.000439	GTF2F2	0.375172
351	DDX1	22	SLX1A	0.088889	BRF2	0.000438	CSTB	0.375172
352	CDT1	22	UNG	0.088889	SNRPA1	0.000436	MSH2	0.37512
353	C14orf1	22	MAPK1	0.09	RAC1	0.000435	HSP90B1	0.37512
354	BIN1	22	VWF	0.09	CTSH	0.000434	SDHA	0.375069
355	ATR	22	CHD3	0.09058	MYH9	0.000432	MTDH	0.375069
356	TERF1	21	CSNK2A1	0.09058	PPP2R2B	0.000431	HMGN1	0.375069
357	TBP	21	BCAR1	0.090909	PBK	0.00043	FOSL2	0.375017
358	SRSF9	21	CREM	0.090909	CASP4	0.000429	RECQL5	0.374966
359	RTN4	21	EEF1D	0.090909	FHL1	0.000428	DNMT3A	0.374966
360	PSMD4	21	GNB2L1	0.090909	NOTCH1	0.000427	SLC4A8	0.374914
361	PSMD2	21	MEF2D	0.090909	APBB1	0.000427	PRPS1L1	0.374914
362	PPP2R2B	21	MGMT	0.090909	MEF2D	0.000426	FBXO21	0.374914
363	NR5A1	21	NEDD4	0.090909	PIN1	0.000424	DAZAP1	0.374914
364	NCK1	21	SFN	0.090909	LSM4	0.000424	CNTN2	0.374914
365	HUWE1	21	SMAD4	0.090909	TAF10	0.000421	TEX10	0.374863
366	HSPA2	21	PCNA	0.091026	TSNAX	0.000417	HUWE1	0.374863
367	FXR2	21	CDC42	0.091168	NUP155	0.000416	ANKS1A	0.374863
368	EIF1B	21	VAV2	0.091503	SIKE1	0.000413	NQO1	0.37476
369	CSNK2B	21	RBBP4	0.091525	AHR	0.000407	ERCC1	0.37476
370	CPSF1	21	UIMC1	0.091954	LAMC1	0.000406	EID1	0.37476
371	CDC37	21	MAGI1	0.093567	KIF2C	0.000405	PDXK	0.374709
372	ST13	20	MED26	0.094017	CCND1	0.000405	F2RL1	0.374657
373	SAFB	20	RAN	0.094118	ATP2B4	0.000402	PTPRE	0.374606
374	RUVBL2	20	INSL3	0.09434	ITGB1	0.000401	DRG1	0.374554
375	RRP1B	20	PDGFRB	0.094536	PRNP	0.000399	SP1	0.374452
376	PSMC5	20	MAPK14	0.094862	IL4R	0.000399	PIK3R3	0.374452
377	PIN1	20	BID	0.095238	FANCI	0.000398	FBXO25	0.374452
378	PAK2	20	COMT	0.095238	PSMA3	0.00039	HDAC5	0.3744
379	NPM1	20	CSTA	0.095238	CDK2	0.00039	EIF2B3	0.3744
380	IQCB1	20	DNM2	0.095238	SNCA	0.000385	MCF2	0.374349
381	HNRNPU	20	EGR2	0.095238	DYNLL1	0.000385	RRP12	0.374298
382	HNRNPD	20	HSP90B1	0.095238	NASP	0.000382	NQO2	0.374247
383	FKBP4	20	HSPA2	0.095238	NUP62	0.00038	RNF139	0.374195
384	EIF4E	20	ITGA6	0.095238	SPA17	0.000378	COMT	0.374144
385	DHX30	20	JAK3	0.095238	RABEP1	0.000377	TTI2	0.374093

386	CPSF6	20	JUND	0.095238	EIF5	0.000375	AHSA1	0.374093
387	COPS6	20	KLK13	0.095238	NFKBIE	0.000372	MSTO1	0.374042
388	CDC16	20	MAPKAPK2	0.095238	POMC	0.00037	ATP2B1	0.374042
389	CD82	20	MDM4	0.095238	BIN1	0.000369	TNC	0.37399
390	USP7	19	MRPS31	0.095238	NSFL1C	0.000368	MRPS22	0.37399
391	UBE2E1	19	MYD88	0.095238	TGM2	0.000367	BRF2	0.37399
392	TAF9	19	NCK1	0.095238	CAV1	0.000367	NUDT1	0.373939
393	SLX4	19	NFATC3	0.095238	PRKCG	0.000365	AGT	0.373939
394	SKP2	19	NR3C1	0.095238	MSH6	0.000365	SNRPA1	0.373888
395	RNF139	19	NR5A1	0.095238	BCAT1	0.000363	SARNP	0.373888
396	RNF10	19	PAFAH1B3	0.095238	UBE2S	0.000362	RRBP1	0.373888
397	RBX1	19	RYK	0.095238	HMGB1	0.000362	DUSP15	0.373888
398	NUP210	19	SMARCD3	0.095238	HMGCR	0.000357	CSTA	0.373888
399	NFKB2	19	SOD1	0.095238	HDGF	0.000356	RPL10L	0.373837
400	NDEL1	19	TP53BP2	0.095238	TKT	0.000355	MDH1	0.373786
401	MAPKAPK5	19	UCHL5	0.095238	HTT	0.000355	DDX4	0.373735
402	MAPK13	19	LPAR2	0.095307	TXNRD1	0.000354	C14orf166	0.373735
403	MAGI1	19	BRF2	0.095588	GTF2E2	0.000353	KIF15	0.373632
404	LRPAP1	19	DNMT1	0.095816	CUL1	0.000353	BAZ1B	0.373632
405	KPNA1	19	TCP1	0.096327	SPAG5	0.000352	HGS	0.373581
406	IL7R	19	YWHAG	0.097561	PPP2CA	0.000346	TFAP2C	0.37353
407	HNRNPC	19	YWHAB	0.098039	LYL1	0.000346	HIST1H2AG	0.37353
408	HERPUD1	19	PPP2CA	0.098814	FOS	0.000346	ST13	0.373479
409	EIF5	19	SPTAN1	0.098901	TPD52L1	0.000345	MAP2K6	0.373428
410	DDB1	19	BAZ1B	0.099415	SRRM2	0.000345	ALCAM	0.373326
411	CDC34	19	BARD1	0.099865	SREBF2	0.000345	VPS13A	0.373275
412	BTRC	19	ADAM9	0.1	PRKAR1A	0.000345	SUGP1	0.373224
413	BIRC5	19	ADD1	0.1	USO1	0.000344	FAM98A	0.373224
414	BAZ1B	19	AIFM1	0.1	CSTB	0.000342	PLBD2	0.373122
415	ATXN1	19	ANXA2	0.1	MLF2	0.000341	ELAVL2	0.373122
416	YWHAB	18	ATF4	0.1	ILF2	0.00034	CSTF3	0.373071
417	VAV2	18	BABAM1	0.1	CD4	0.00034	RANBP1	0.37302
418	TUBB4	18	C9orf78	0.1	DDX20	0.000339	PLCH1	0.37302
419	TUBA1A	18	CAPZA1	0.1	EIF2B3	0.000337	MAPK8IP1	0.37302
420	SLBP	18	CDC73	0.1	CPSF3	0.000337	RNF10	0.372867
421	SF3B3	18	CETN3	0.1	PPM1G	0.000335	OIP5	0.372715
422	RAC1	18	DGCR8	0.1	CLGN	0.000335	GRK4	0.372715
423	PSMD11	18	DLG1	0.1	SMN1	0.000333	SHC1	0.372613
424	PRKDC	18	DNM1	0.1	PRKCQ	0.000333	HJURP	0.372613
425	MLH1	18	FBXO25	0.1	PRKAB1	0.000332	VHL	0.372512
426	MAP3K1	18	FHL5	0.1	PLA2G4A	0.00033	APH1B	0.372309
427	MAP3K14	18	GANAB	0.1	CDKN1B	0.00033	ADRBK1	0.372309
428	INCENP	18	GRIN2B	0.1	RAD21	0.000328	KIAA1683	0.372207
429	HNRNPM	18	GSR	0.1	DCTN1	0.000327	ITGA3	0.372207
430	DTNBP1	18	HDAC7	0.1	CDKN2C	0.000326	MAGEH1	0.372106
431	DEPDC7	18	HIST1H2AG	0.1	TRIP13	0.000325	ITGB5	0.372106
432	CHEK1	18	HSD17B3	0.1	RANBP1	0.000325	PSMA3	0.372004
433	CD4	18	HSPE1	0.1	MAPK13	0.000324	NPW	0.372004
434	ANAPC2	18	HTRA2	0.1	COMT	0.000324	C12orf10	0.372004
435	VCP	17	IFT57	0.1	AP2B1	0.000323	YY1AP1	0.371954
436	USP2	17	ITPR2	0.1	SPAG9	0.000322	CDK16	0.371954
437	TXNRD1	17	ITPR3	0.1	LGALS1	0.000321	APLP1	0.371954
438	TOPBP1	17	MAST1	0.1	VAV2	0.000319	SIKE1	0.371903
439	TKT	17	NUDT1	0.1	MME	0.000319	PROK1	0.371903
440	TAB2	17	PCID2	0.1	CDK16	0.000318	EDNRB	0.371903
441	SNRNP70	17	PKN1	0.1	QKI	0.000316	EDNRA	0.371903
442	RHOA	17	PLD2	0.1	CRK	0.000316	CHD1L	0.371852
443	RECQL5	17	PPIB	0.1	CKAP4	0.000316	CDC45	0.371852
444	RBL2	17	PRRC2A	0.1	MAP3K14	0.000315	CAPZA1	0.371802
445	PSME1	17	RAP1A	0.1	GSTP1	0.000315	UBE2T	0.371751
446	PSMD7	17	RNF32	0.1	CCNE1	0.000315	CCNA1	0.371751
447	PSMD12	17	RPL10L	0.1	KAT5	0.000313	COL1A2	0.371701
448	PSMC6	17	SARNP	0.1	YWHAB	0.000312	POGZ	0.37165
449	PSMA1	17	SERPINA5	0.1	GET4	0.000311	LRR1	0.37165
450	PPP1CA	17	SERPINB9	0.1	CPSF6	0.00031	SOD1	0.3716
451	PIK3R2	17	SMC3	0.1	RAF1	0.000308	ARRB1	0.371549

452	NEDD8	17	SMG1	0.1	HSPD1	0.000307	SCAPER	0.371499
453	NCOR2	17	SPECC1L	0.1	EDNRB	0.000306	CREBBP	0.371499
454	NASP	17	SPG20	0.1	EDNRA	0.000306	USP11	0.371448
455	IL4R	17	TBL2	0.1	NR3C1	0.000305	SAE1	0.371448
456	HTT	17	TLE1	0.1	GSR	0.000305	LAMB2	0.371347
457	HNRNPR	17	UBE2E2	0.1	RAB11FIP5	0.000304	GAPVD1	0.371246
458	HMGA1	17	HDAC1	0.100483	NR5A1	0.000304	NFRKB	0.371095
459	GSTP1	17	HSP90AB1	0.100985	KIF18A	0.000302	CDH2	0.371095
460	GNA13	17	EDN1	0.101124	GNAI3	0.0003	MAGEB2	0.371044
461	GABARAPL2	17	LYAR	0.102564	BAG6	0.000298	HERPUD1	0.371044
462	FHL1	17	SH3GL3	0.102564	PNMA1	0.000296	ERBB2	0.371044
463	DNMT3A	17	RECQL5	0.102941	NUP88	0.000296	CPSF6	0.371044
464	DCTN1	17	CUL4B	0.103175	ITGB5	0.000296	ALB	0.370994
465	CRK	17	SPAG9	0.103896	CSNK2B	0.000296	AGTR1	0.370994
466	CDKN2C	17	RAD21	0.104615	BECN1	0.000296	ADORA3	0.370994
467	CD46	17	RPL26L1	0.104839	WIPI2	0.000294	DDX42	0.370893
468	BRF2	17	RUVBL2	0.105263	TGFBR2	0.000292	RAD18	0.370843
469	ARHGAP19	17	QKI	0.106061	HLA-C	0.000292	PGM1	0.370793
470	ANK2	17	TAB1	0.106061	DEPDC7	0.000292	EGR1	0.370793
471	ANAPC11	17	APH1B	0.106667	A2M	0.000292	TRIOBP	0.370591
472	AGT	17	APBB1	0.107143	EGR2	0.000288	PPP2R4	0.370591
473	VAV1	16	BTK	0.107143	PAK2	0.000285	BRD1	0.370591
474	UBE2U	16	CASP10	0.107143	PRKCZ	0.000283	INTS6	0.370541
475	TNFRSF1B	16	DDX3X	0.107143	HPRT1	0.000283	CDKN2C	0.370541
476	TNFRSF1A	16	FES	0.107143	CTSS	0.000283	SPAG9	0.370441
477	SNCA	16	HSPA1L	0.107143	NFKBIA	0.000281	MVP	0.37029
478	RIPK3	16	ILK	0.107143	DHX9	0.00028	NPHP1	0.37024
479	RDX	16	ITGAX	0.107143	RPA2	0.000279	CD46	0.37024
480	PTBP1	16	ITPR1	0.107143	CDC20	0.000279	XPO5	0.37019
481	PSMF1	16	MAPK10	0.107143	ANK2	0.000279	NR3C1	0.37014
482	PSMB6	16	PRKCI	0.107143	XPO5	0.000277	HELLS	0.37014
483	PSMA7	16	SMAD1	0.107143	SFN	0.000277	GNA13	0.370039
484	POLE	16	TOX4	0.107143	ESR2	0.000277	TRIO	0.369989
485	POLA2	16	UBE3A	0.107143	EZR	0.000275	ANK2	0.369989
486	ORC2	16	TRRAP	0.107692	RPA1	0.000274	RPN2	0.369939
487	NFKBIA	16	DGKE	0.108333	PSEN1	0.000274	RLN3	0.369939
488	LGALS1	16	HMGCR	0.108333	NUCB2	0.000273	SYCP1	0.369889
489	KIAA1377	16	ITGA3	0.108333	HRAS	0.000272	CRYAB	0.369889
490	KAT5	16	RIPK3	0.108333	ERCC1	0.000271	WIPI2	0.369739
491	ITGA3	16	VAV1	0.108333	HIF1A	0.000269	RCOR3	0.369689
492	HPRT1	16	HSPA8	0.108466	ATM	0.000269	EIF2AK2	0.369689
493	HNRNPA2B1	16	FBL	0.108871	SAFB	0.000268	ATM	0.369689
494	HMGCS1	16	PIAS2	0.109531	DLG4	0.000268	F2RL2	0.369639
495	HMGCR	16	LMNA	0.109635	ARHGAP19	0.000268	PSAT1	0.369589
496	FANCA	16	PARK7	0.109677	SPTAN1	0.000265	G6PD	0.369589
497	EPB41	16	EIF2AK2	0.10989	FBXO7	0.000265	SORT1	0.369539
498	DGKE	16	SAE1	0.10989	CSTF3	0.000265	VCAN	0.369489
499	CCNB2	16	HSP90AA1	0.109929	NCK1	0.000264	LAMC1	0.369489
500	ANAPC7	16	CDC25A	0.110194	MAP3K1	0.000264	NPY5R	0.369439
501	XPO5	15	RHOA	0.110294	PIK3R2	0.000263	NUTF2	0.369389
502	WDR61	15	ATP2B4	0.111111	CKS2	0.000263	MAP3K14	0.369389
503	UCHL5	15	DDB1	0.111111	TGFB1	0.000262	SPECC1L	0.369289
504	UBE2C	15	EID1	0.111111	EXOC7	0.000262	MME	0.369289
505	TUBB2C	15	EIF6	0.111111	MAPK9	0.000261	CCNE1	0.369289
506	SPARC	15	IFIT3	0.111111	HMGA1	0.000261	ARHGEF1	0.369189
507	SOD1	15	MAP3K14	0.111111	CANX	0.000259	BNIP3L	0.369139
508	SAP130	15	MAPKAPK5	0.111111	EIF4G2	0.000258	CCKBR	0.369089
509	RBM5	15	MTDH	0.111111	PARK2	0.000257	NCAM1	0.36904
510	PTK2	15	PRKCB	0.111111	BAZ1B	0.000257	LLGL1	0.36904
511	PSMD3	15	RCOR3	0.111111	RECQL5	0.000256	SMAD3	0.36894
512	PSMD1	15	SLX1B	0.111111	MCC	0.000256	CDK2	0.36889
513	PSMD13	15	TGFBR2	0.111111	DDX1	0.000255	CDC34	0.36889
514	PSMC4	15	TLR4	0.111111	TGFBR1	0.000254	HYOU1	0.36879
515	PSMC2	15	CCNA2	0.111837	DCP1B	0.000253	DEPDC7	0.36879
516	PSMB1	15	AR	0.112644	NUP153	0.00025	SH3GLB1	0.368741
517	PSMA6	15	CBX3	0.113333	NFKB2	0.00025	ZNF473	0.368691

518	PSMA4	15	CCNB1	0.115198	MEF2C	0.00025	SSR1	0.368691
519	PSMA2	15	NCOA2	0.115385	BCL2	0.00025	GINS1	0.368691
520	PRKCD	15	POU2F1	0.115385	CDC27	0.000249	GABBR1	0.368591
521	PLCG1	15	UBA52	0.115646	SYK	0.000246	KHDRBS1	0.368542
522	PIAS1	15	DHX30	0.115789	EIF2AK2	0.000246	EGR2	0.368542
523	PCF11	15	RBL1	0.116078	CDCA8	0.000246	ARHGAP19	0.368542
524	ORC3	15	ACTB	0.116129	CASP1	0.000244	UBE2D1	0.368492
525	NINL	15	PIK3R1	0.116129	MYH10	0.000243	TTK	0.368492
526	MME	15	CCT2	0.116162	VAMP2	0.000241	MPRIP	0.368442
527	MCM3	15	SRRM2	0.116667	MOB4	0.000241	FBXO7	0.368442
528	MBD1	15	PPP2R4	0.116883	CNOT7	0.000241	AKAP8L	0.368442
529	MAPK9	15	NFKB1	0.116935	ERBB2	0.00024	ZNF318	0.368393
530	LSM4	15	CCNA1	0.118269	NFYA	0.000239	DHX9	0.368393
531	LLGL1	15	RAD23B	0.118619	NEDD1	0.000239	YEATS4	0.368343
532	KIF11	15	MNAT1	0.118804	CRYAB	0.000239	LRPAP1	0.368343
533	KALRN	15	ADORA3	0.119231	KALRN	0.000238	CXCL10	0.368343
534	ITGB1	15	MCF2	0.119565	CCNB2	0.000238	FLOT1	0.368244
535	HSPD1	15	CSE1L	0.12	KPNA1	0.000236	SSFA2	0.368194
536	HLA-E	15	LAMB2	0.120879	HMGCS1	0.000236	SENP1	0.368194
537	HIF1A	15	SUMO3	0.120879	DHX30	0.000236	PAK2	0.368144
538	GNB1	15	CCL27	0.120986	CHD3	0.000236	NCAPD2	0.368144
539	GNA12	15	JAG2	0.121212	KRT18	0.000234	QKI	0.368095
540	FOS	15	MOB4	0.121212	GNB2L1	0.000234	ACAT2	0.367946
541	FN1	15	NFATC2	0.121212	DNAJB6	0.000234	MAPK10	0.367897
542	FBXO5	15	RIPK2	0.121212	SOD1	0.000233	CCL27	0.367897
543	EFTUD2	15	SIKE1	0.121212	GABARAPL2	0.000233	UBXN7	0.367847
544	DCTN2	15	UBB	0.121599	DYNLL2	0.000232	STX7	0.367847
545	CDKN2A	15	RACGAP1	0.121622	FRS3	0.000231	POLR3B	0.367847
546	CDC26	15	TAF9	0.122807	PTPN11	0.00023	CD36	0.367847
547	CDC23	15	SLC9A3R1	0.123077	MAPK3	0.00023	BLMH	0.367847
548	ARHGAP28	15	SUPT5H	0.123333	TP53BP1	0.000229	ARFGEF1	0.367847
549	ANAPC5	15	MCM7	0.123543	INSL3	0.000228	PLA2G4A	0.367798
550	ANAPC4	15	FOS	0.12381	UBE2U	0.000227	MAD2L2	0.367798
551	ANAPC1	15	HSPD1	0.12381	GFAP	0.000227	RFWD3	0.367748
552	AKAP9	15	ITGB1	0.12381	HDAC3	0.000226	GABARAP	0.367699
553	WIPI2	14	TBP	0.12381	MMEL1	0.000221	DIAPH3	0.367699
554	VCAN	14	MLH1	0.124183	SEPT2	0.000219	PLK1	0.367649
555	UBE2T	14	HTT	0.125	KIAA1377	0.000219	MAP3K3	0.367649
556	TSC22D1	14	CCT5	0.126597	SKP2	0.000218	RAC1	0.3676
557	TRIO	14	FANCI	0.126812	PTK2	0.000218	DTL	0.3676
558	TP73	14	IRF1	0.127273	GRK4	0.000218	CETN3	0.3676
559	TOP1	14	SMN2	0.127273	ATR	0.000218	ATG9A	0.3676
560	TNIK	14	TRAF3	0.127273	DTNBP1	0.000217	RANBP3	0.36755
561	TFDP1	14	SREBF2	0.128205	ANAPC11	0.000217	DYNLL2	0.36755
562	SUMO3	14	NFKB2	0.128655	G6PD	0.000216	PTMA	0.367501
563	SQSTM1	14	HSPA4	0.12987	ARHGEF1	0.000216	HLA-B	0.367451
564	SPTAN1	14	ATM	0.13	TSC22D1	0.000215	WDR61	0.367402
565	SMC4	14	RPS27A	0.131401	TRIO	0.000215	GNAI2	0.367402
566	SET	14	FKBP4	0.131579	GNA12	0.000215	MTNR1A	0.367352
567	SEPT2	14	RRP1B	0.131579	TNFRSF1B	0.000213	PDGFD	0.367303
568	SAE1	14	PSME3	0.131783	UBE2E1	0.000212	MAP1LC3B	0.367303
569	RFWD2	14	YWHAE	0.132132	TNIK	0.000212	TBC1D15	0.367254
570	RAF1	14	SP1	0.132308	KIF15	0.000211	SYK	0.367254
571	PTPN11	14	ADA	0.133333	APOA1BP	0.000211	LPAR4	0.367254
572	PSME2	14	BAX	0.133333	SOX9	0.000209	ADAM17	0.367204
573	PSMD9	14	CDK5	0.133333	MBD1	0.000209	WDR62	0.367155
574	PSMD5	14	CNTN2	0.133333	PYGL	0.000208	USP2	0.367056
575	PSMD14	14	DIAPH3	0.133333	GABBR1	0.000208	EIF5	0.367056
576	PSMC3	14	ELAVL1	0.133333	KCTD13	0.000204	ADA	0.367056
577	PSMC1	14	ERBB3	0.133333	MED26	0.000203	SSX2IP	0.366958
578	PSMB7	14	FANCA	0.133333	F10	0.000202	RECQL	0.366908
579	PSMB5	14	FGFR1	0.133333	TEX10	0.0002	MRPS31	0.366908
580	PSMB4	14	GRK4	0.133333	NEDD8	0.0002	ETFA	0.366908
581	PSMB3	14	HIST1H1A	0.133333	CDC34	0.0002	CDC42	0.366908
582	PSMB2	14	HLA-A	0.133333	ACTG1	0.0002	TKT	0.366859
583	PSMB10	14	HLA-C	0.133333	GABBR2	0.000199	PPIB	0.36681

584	POLE2	14	HMGN1	0.133333	PTPRC	0.000198	HMGCR	0.36681
585	PAXIP1	14	MAP3K5	0.133333	TANK	0.000197	ANKIB1	0.36681
586	ORC6	14	MAP3K8	0.133333	CASC5	0.000197	TP53BP2	0.366761
587	ORC4	14	MDH1	0.133333	EIF4G1	0.000196	LONP1	0.366711
588	OFD1	14	NPHP1	0.133333	SHC1	0.000195	VEZT	0.366662
589	MPRIP	14	PARK2	0.133333	PEX5	0.000195	FHL1	0.366662
590	MCM6	14	PLAA	0.133333	GCDH	0.000194	CXCL9	0.366662
591	MCM2	14	PPARG	0.133333	SUGP1	0.000193	AASS	0.366662
592	MAP3K7	14	PRKCE	0.133333	SLC1A5	0.000193	UBR2	0.366613
593	LONP1	14	RAD52	0.133333	SKP1	0.000192	TRAF2	0.366613
594	LAMB2	14	RXRA	0.133333	UBE2D2	0.00019	VRK3	0.366515
595	KTN1	14	S100A11	0.133333	MAGEB2	0.00019	SMC4	0.366515
596	KIF15	14	SLC2A4	0.133333	DNM1	0.00019	PNMA1	0.366465
597	JAK2	14	SPAG5	0.133333	PRKDC	0.000189	JPH1	0.366465
598	HLA-C	14	STAT6	0.133333	SNX17	0.000188	IL4R	0.366465
599	HIP1	14	TERF1	0.133333	UTS2	0.000186	SATB2	0.366416
600	GNG2	14	THAP11	0.133333	RANBP3	0.000186	MMP2	0.366416
601	EIF2AK2	14	UBR2	0.133333	PRKG1	0.000186	SEPT2	0.366367
602	E2F4	14	UHRF2	0.133333	KIT	0.000186	LPAR1	0.366367
603	CSTB	14	USP19	0.133333	CKAP5	0.000186	KTN1	0.366367
604	CKS2	14	CCT8	0.133998	TNFRSF1A	0.000185	KLK5	0.366367
605	ARHGEF1	14	TAF10	0.136134	RFWD3	0.000185	HTR2A	0.366367
606	ARHGEF12	14	ACTG1	0.136364	MAD2L2	0.000185	AASDHPPT	0.366367
607	ANAPC10	14	HGS	0.136364	TOPBP1	0.000184	RSBN1L	0.366318
608	ZBTB32	13	PSEN1	0.136364	MEPCE	0.000184	PLAA	0.366318
609	YY1AP1	13	TARDBP	0.136364	MAPK8IP2	0.000184	GNB1	0.366318
610	UBE2D2	13	XIAP	0.136364	ITGB4	0.000184	CHIC2	0.366318
611	TUBGCP4	13	DEPDC7	0.137255	BIRC5	0.000184	ZBTB32	0.366269
612	SYK	13	POT1	0.137566	AP2A2	0.000184	TXNRD1	0.366269
613	SREBF2	13	AKT1	0.13834	SDHA	0.000181	PLCG1	0.366269
614	SH3GL3	13	CSNK2A2	0.138889	LONP1	0.000181	DDX19B	0.366269
615	RPS6KB2	13	G6PD	0.138889	PSEN2	0.00018	BTRC	0.366269
616	RFWD3	13	GADD45A	0.138889	MPRIP	0.00018	APC	0.366269
617	PSME4	13	NRIP1	0.138889	DNMT3A	0.00018	PTK2	0.36622
618	POU2F1	13	PRPF40A	0.138889	CUL5	0.00018	PPM1G	0.36622
619	POLA1	13	RCC1	0.138889	XIAP	0.000178	HMGCS1	0.36622
620	PLCB2	13	RIPK1	0.138889	TBP	0.000178	GNG2	0.36622
621	PIK3R3	13	TGM2	0.138889	PTEN	0.000178	MAP3K1	0.366171
622	PCM1	13	TTK	0.138889	PPP1CA	0.000178	LSM4	0.366171
623	ORC5	13	APC	0.141304	EIF1B	0.000177	GSTM3	0.366171
624	NSFL1C	13	RFC4	0.142045	USP7	0.000176	GCDH	0.366171
625	NEDD1	13	TP53BP1	0.142292	S100A6	0.000176	TOR1AIP1	0.366122
626	NCOA2	13	APEX1	0.142857	CDK9	0.000176	PIWIL1	0.366122
627	NAE1	13	BAG1	0.142857	YY1AP1	0.000175	MPHOSPH9	0.366122
628	MCM8	13	BCL2L1	0.142857	TUBA1A	0.000175	MLF2	0.366122
629	KLK5	13	CALM3	0.142857	MBP	0.000175	HSD17B4	0.366122
630	FGFR1OP	13	CYCS	0.142857	CPSF1	0.000175	BDKRB2	0.366122
631	FBXO7	13	DNAJB11	0.142857	SMU1	0.000174	P2RY1	0.366073
632	ETFA	13	DSG1	0.142857	MAP3K5	0.000174	ADORA2A	0.366024
633	ERCC2	13	ERBB4	0.142857	CSNK1A1	0.000174	TRIP13	0.365975
634	ERBB2	13	GTF3C4	0.142857	POLR3B	0.000173	LMNB1	0.365975
635	EIF4G1	13	HLA-B	0.142857	TNF	0.000172	HNRNPU	0.365975
636	DNAJB6	13	HYRC	0.142857	IRF1	0.000172	GNAQ	0.365975
637	CXCR4	13	IRAK1	0.142857	DRG1	0.000171	COL6A1	0.365975
638	CSNK1D	13	KARS	0.142857	PLAA	0.00017	NMUR2	0.365926
639	CSNK1A1	13	MED31	0.142857	SGK1	0.000169	NMUR1	0.365926
640	CNOT7	13	MYBBP1A	0.142857	DLG1	0.000169	NMU	0.365926
641	CIAO1	13	PEX5	0.142857	AASDHPPT	0.000169	NFATC2	0.365926
642	CHD1L	13	RPS6KA5	0.142857	CARS	0.000168	MCHR2	0.365926
643	CETN2	13	SAP130	0.142857	BTRC	0.000168	MCHR1	0.365926
644	CEP70	13	SAP18	0.142857	UCHL5	0.000167	GNA12	0.365926
645	CDK5RAP2	13	SLC9A3R2	0.142857	RUNX1	0.000167	GHRL	0.365926
646	CD99	13	STAM2	0.142857	MAPK10	0.000167	FPR2	0.365926
647	CCP110	13	TNC	0.142857	TP73	0.000166	BDKRB1	0.365926
648	CASP6	13	TP73	0.142857	MAPRE2	0.000166	APBB1	0.365926
649	BDKRB2	13	TRIM24	0.142857	MAP3K7	0.000166	WAPAL	0.365877

650	ATN1	13	TSC1	0.142857	ATP5A1	0.000165	UBE2S	0.365877
651	AMFR	13	TXN	0.142857	PSMD13	0.000164	SCAF4	0.365877
652	AKAP8L	13	UBA1	0.142857	MTNR1A	0.000164	MYO18A	0.365877
653	AGTR1	13	WTAP	0.142857	KTN1	0.000164	F2R	0.365877
654	ACLY	13	PSMD6	0.143457	CENPN	0.000164	F2RL3	0.365877
655	XIAP	12	CUL4A	0.145455	BUB3	0.000164	SIK3	0.365828
656	UBQLN4	12	KRT18	0.145455	SLX4	0.000163	NPSR1	0.365828
657	UBE2S	12	LAMC3	0.145455	VRK1	0.000161	ANLN	0.365828
658	TRIP13	12	NCOA1	0.145455	PTMA	0.000161	IFIT3	0.365779
659	TPX2	12	POGZ	0.145455	PSMD2	0.000161	DERL2	0.365779
660	TNF	12	CRK	0.147059	POGZ	0.000161	DCTN2	0.365779
661	TGFBR1	12	DNMT3A	0.147059	NPW	0.000161	CCR10	0.365779
662	TARDBP	12	SMN1	0.147126	IL16	0.000161	VPS37A	0.36573
663	TAP1	12	PROK1	0.147566	CXCL10	0.000161	UTS2D	0.36573
664	TANK	12	CD4	0.150327	STOM	0.00016	TRH	0.36573
665	TAF4	12	DYNC1H1	0.150504	KIAA1683	0.00016	PROKR2	0.36573
666	TAF1	12	PML	0.151515	ARHGEF12	0.00016	PROKR1	0.36573
667	TAB1	12	PLCG1	0.152381	TARDBP	0.000159	OPN4	0.36573
668	SOX9	12	PAK2	0.152632	ATG9A	0.000158	OGG1	0.36573
669	SFN	12	CASP6	0.153846	UBQLN4	0.000157	NMBR	0.36573
670	SDHA	12	MAP3K7	0.153846	OXSR1	0.000157	NMB	0.36573
671	RIPK2	12	ARHGAP19	0.154412	E2F1	0.000157	LPAR6	0.36573
672	RFC1	12	TOP2A	0.154655	HNRNPU	0.000155	LAMP2	0.36573
673	QKI	12	CDKN1A	0.155128	EID1	0.000155	HRH1	0.36573
674	PTMA	12	RB1	0.155242	USP44	0.000154	GRPR	0.36573
675	PTAFR	12	BRCA2	0.155303	TAB2	0.000152	GRP	0.36573
676	PSEN1	12	EIF4G2	0.155556	MAGED1	0.000152	GPR68	0.36573
677	PLCB1	12	KHDRBS1	0.155556	HNRNPD	0.000152	GPR65	0.36573
678	PBK	12	NFKBIB	0.155556	GNAI2	0.000152	GPR4	0.36573
679	OIP5	12	PPP2R2D	0.155556	USP45	0.000151	GNRHR	0.36573
680	NMUR2	12	STK24	0.155556	RCOR3	0.000151	GNRHR2	0.36573
681	NMUR1	12	STRN4	0.155556	GTF2F1	0.000151	GNRH1	0.36573
682	NMU	12	NEK2	0.1558	ACAT2	0.000151	GHSR	0.36573
683	NCL	12	RAC1	0.156863	TXN	0.00015	CHRM5	0.36573
684	MYH9	12	RAD51	0.156863	ITPR3	0.000149	CHRM3	0.36573
685	MVP	12	GMNN	0.157344	ITPR2	0.000149	AVPR1A	0.36573
686	MOB4	12	EIF5	0.157895	TRPC1	0.000147	ADRA1A	0.36573
687	MCHR2	12	TFAP2A	0.158103	CACNA1A	0.000146	PMCH	0.365681
688	MCHR1	12	NFKBIA	0.158333	EXOSC8	0.000145	PLG	0.365681
689	MAPK3	12	NFKBIE	0.15873	UBE2T	0.000144	LPAR3	0.365681
690	MAGED1	12	RPA3	0.160762	IFIT3	0.000144	EXOC7	0.365681
691	MAGEB2	12	PSMA5	0.16087	PAXIP1	0.000143	GNA14	0.365632
692	LRIF1	12	LSM4	0.161905	FOSL2	0.000143	GNA11	0.365632
693	LPAR1	12	AP2B1	0.163636	TFDP2	0.000142	GET4	0.365632
694	LMNB1	12	CAV1	0.163636	PLD2	0.000142	WDR35	0.365583
695	JAK1	12	NFYA	0.163636	NUP35	0.000142	PROK2	0.365583
696	JAG2	12	S100A6	0.163636	RUVBL2	0.000141	P2RY11	0.365583
697	ITCH	12	MCM5	0.164363	PSMD4	0.000141	CALB2	0.365583
698	HGS	12	USP1	0.164502	JAK2	0.000141	HTT	0.365534
699	GTF2B	12	HIP1	0.164835	AMOT	0.000141	YWHAB	0.365485
700	GNB2L1	12	SET	0.164835	ZBTB32	0.000139	RAB11FIP5	0.365485
701	FPR2	12	TRIO	0.164835	GSN	0.000139	PTGFR	0.365485
702	ETS1	12	ABCA1	0.166667	AHSA1	0.000139	NTSR2	0.365485
703	EIF2B3	12	AKT2	0.166667	ITCH	0.000138	NPFFR2	0.365485
704	EEF1D	12	ALS2CR11	0.166667	HNRNPA1	0.000138	NPFFR1	0.365485
705	DYNC1I2	12	AMPH	0.166667	GRIN2B	0.000138	LTB4R2	0.365485
706	DKC1	12	AP2A2	0.166667	NPY5R	0.000137	GPRC6A	0.365485
707	DAZAP1	12	AP2M1	0.166667	RNF216	0.000136	C5orf25	0.365485
708	CSNK1E	12	BHLHE40	0.166667	NPM1	0.000136	WDTC1	0.365436
709	CKAP4	12	CALD1	0.166667	CDK7	0.000136	STK11IP	0.365436
710	CEP63	12	CD19	0.166667	TNC	0.000135	KLHL8	0.365436
711	CEP57	12	CD36	0.166667	TERF1	0.000135	DNAJB4	0.365436
712	CEP250	12	CHIC2	0.166667	TAP1	0.000135	DLG4	0.365436
713	CDK16	12	CTTN	0.166667	SAP130	0.000135	TPP2	0.365387
714	CCNE2	12	CYP17A1	0.166667	LCK	0.000135	SRRM2	0.365387
715	CCL5	12	CYP51A1	0.166667	KARS	0.000135	NGLY1	0.365338

716	CBX1	12	DDX4	0.166667	TTI2	0.000134	MKRN1	0.365338
717	CASP7	12	DEDD	0.166667	CDK5	0.000134	HMGN3	0.365338
718	C3	12	DHX16	0.166667	MVP	0.000133	NSMAF	0.365289
719	BLM	12	DNAJA2	0.166667	PLG	0.00013	GTF3C4	0.365289
720	BDKRB1	12	EEA1	0.166667	MED12	0.00013	ATAD2	0.365289
721	AZI1	12	EIF4G1	0.166667	CDC6	0.00013	ANXA4	0.365289
722	ATG5	12	ERBB2IP	0.166667	RPS20	0.000129	PIM2	0.365241
723	ARHGDIA	12	ERG	0.166667	AP2M1	0.000129	NEFL	0.365241
724	ALB	12	FANCC	0.166667	SH3KBP1	0.000128	MAP1LC3A	0.365241
725	ACTR1A	12	FTL	0.166667	PSMC5	0.000128	C9orf78	0.365241
726	ACTG1	12	HLA-C	0.166667	APBA1	0.000128	RANBP2	0.365192
727	A2M	12	HNF4A	0.166667	ACLY	0.000128	DCP1B	0.365192
728	TUBGCP6	11	IL2RG	0.166667	MGMT	0.000127	XPNPEP1	0.365143
729	TUBGCP5	11	INO80B	0.166667	CAMK2B	0.000126	TBL2	0.365143
730	TUBGCP3	11	INSIG2	0.166667	SMC4	0.000125	IFIT5	0.365143
731	TUBGCP2	11	INSR	0.166667	PPP1CC	0.000125	DHX16	0.365143
732	TUBG2	11	ITGA2	0.166667	MYBL2	0.000125	AARS	0.365143
733	TTI2	11	KAT5	0.166667	MMP2	0.000125	RALGPS2	0.365094
734	TSNAX	11	KLK2	0.166667	KLK2	0.000125	PPIA	0.365094
735	TSGA14	11	MAPKAPK3	0.166667	IFIT5	0.000125	NCAPH	0.365094
736	TRPC1	11	NID1	0.166667	SAP18	0.000124	CBFB	0.365094
737	TRAF3	11	NOTCH3	0.166667	RANBP9	0.000124	VCP	0.365045
738	TK1	11	NR4A2	0.166667	COL1A2	0.000124	SLC1A5	0.365045
739	TEX10	11	PLD1	0.166667	TOP1	0.000123	KLHDC3	0.365045
740	TAP2	11	PPIA	0.166667	SMURF2	0.000123	GLE1	0.365045
741	SVIL	11	PPP3CA	0.166667	KHDRBS1	0.000123	EXOSC8	0.365045
742	SSNA1	11	PRKCH	0.166667	HNRNPR	0.000123	AP4S1	0.365045
743	SMN2	11	PSAT1	0.166667	FLOT2	0.000123	MKRN2	0.364997
744	SHC1	11	PTPRG	0.166667	CALM3	0.000123	DAZ1	0.364997
745	SH3KBP1	11	PTPRK	0.166667	SAE1	0.000122	CNOT10	0.364997
746	SFI1	11	PYGL	0.166667	PSMD12	0.000122	ADNP2	0.364997
747	SEC61B	11	RICTOR	0.166667	EIF4E	0.000122	SEPT7	0.364948
748	SDCCAG8	11	RPL18A	0.166667	ARHGDIA	0.000122	RAG2	0.364948
749	S100A6	11	SATB2	0.166667	JAK3	0.000121	HEATR1	0.364948
750	RAG1	11	SELE	0.166667	ETFA	0.000121	CTSH	0.364948
751	PTTG1	11	SNCAIP	0.166667	CCT4	0.000121	COX7A2	0.364948
752	PRKAR2B	11	SOCS5	0.166667	ANKS1A	0.000121	WBSCR22	0.364899
753	PRIM2	11	SRF	0.166667	IDE	0.00012	SCAP	0.364899
754	POMC	11	STK11IP	0.166667	GSTM3	0.00012	RNF216	0.364899
755	POGZ	11	STK3	0.166667	GAD1	0.00012	PLEKHG4	0.364899
756	PNMA1	11	STX5	0.166667	BCL3	0.00012	MAPRE2	0.364899
757	PMCH	11	SYK	0.166667	RPLP1	0.000119	HSD17B10	0.364899
758	NPSR1	11	TPM1	0.166667	OIP5	0.000119	HIST1H2BK	0.364899
759	NFYA	11	TRPC6	0.166667	HDAC6	0.000119	EIF4G3	0.364899
760	NEDD4	11	UBE2A	0.166667	FES	0.000119	CDC27	0.364899
761	NCOA6	11	UBE2V1	0.166667	TAP2	0.000118	AMZ2	0.364899
762	NCOA1	11	MAPRE1	0.167856	SSSCA1	0.000118	SQSTM1	0.36485
763	MGMT	11	NCOR2	0.169118	SSFA2	0.000118	QRICH1	0.36485
764	MCM10	11	ARHGAP28	0.171429	CALB2	0.000118	PCID2	0.36485
765	MAD2L2	11	PSMD10	0.171663	ANXA4	0.000118	KCTD13	0.36485
766	LYN	11	MSH6	0.171958	WDR5	0.000117	AKR1B1	0.36485
767	LPAR3	11	ATR	0.17316	TSSC1	0.000117	MLF1	0.364802
768	LAMC3	11	NBN	0.176136	ITGAX	0.000117	IFT57	0.364802
769	KRT18	11	PRKDC	0.176471	GNAS	0.000117	RNFT1	0.364753
770	IRF1	11	TUBG1	0.176744	DDX19B	0.000116	NPM1	0.364753
771	HDAC6	11	E2F1	0.177489	RPS6KB2	0.000115	IST1	0.364753
772	HAUS2	11	PAFAH1B1	0.177538	NFRKB	0.000115	APOA1BP	0.364753
773	GET4	11	DUSP15	0.177778	ZWILCH	0.000114	ZZEF1	0.364704
774	FOXM1	11	EZR	0.177778	MCM3	0.000114	ZC3H18	0.364704
775	F2RL1	11	MAPT	0.177778	TPX2	0.000113	STOM	0.364704
776	EZH2	11	NOTCH1	0.177778	ELAVL1	0.000113	OXSR1	0.364704
777	DCTN3	11	PSEN2	0.177778	FANCA	0.000112	ASRGL1	0.364704
778	CXCL10	11	PSMD8	0.17788	RIPK2	0.000111	RNH1	0.364656
779	CUL4A	11	ANKS1A	0.178571	TK1	0.00011	PRKCZ	0.364656
780	CNTRL	11	FOXO1	0.178571	POLE	0.00011	P4HA1	0.364656
781	CEP72	11	NONO	0.178571	LRIF1	0.00011	MPHOSPH8	0.364656

782	CEP290	11	PRKCQ	0.178571	CSNK2A2	0.00011	MORC4	0.364656
783	CEP192	11	PRPF19	0.178571	TCF4	0.000109	L1CAM	0.364656
784	CEP164	11	SPI1	0.178571	TRAF1	0.000108	MAP1S	0.364607
785	CEP152	11	ITGB3BP	0.180421	SRSF1	0.000108	GSR	0.364607
786	CEP135	11	MBD1	0.180952	IST1	0.000108	ETFB	0.364607
787	CENPJ	11	PIAS1	0.180952	S100A11	0.000107	USP44	0.364558
788	CAV1	11	PTK2	0.180952	GTF3C4	0.000107	TSSC1	0.364558
789	CASP1	11	RBX1	0.181287	APLP2	0.000107	RASA2	0.364558
790	AXIN1	11	MTA1	0.181538	RAD18	0.000106	MEX3B	0.364558
791	AP2B1	11	CASP7	0.181818	HLA-C	0.000106	ISYNA1	0.364558
792	ALMS1	11	EIF2B3	0.181818	DAZAP1	0.000106	DAZL	0.364558
793	AGTR2	11	EZH2	0.181818	RIPK3	0.000105	CCT6B	0.364558
794	ADORA2A	11	NCOA6	0.181818	GTF2B	0.000105	B4GALT1	0.364558
795	ACAT2	11	PRKACA	0.184615	TCF7L2	0.000104	ALDH18A1	0.364558
796	YES1	10	BUB1B	0.184656	NEDD4	0.000104	TOMM34	0.36451
797	WDR5	10	CDC45	0.184848	HNRNPC	0.000104	GLO1	0.36451
798	VRK1	10	JAK2	0.186813	AKAP8L	0.000104	CGRRF1	0.36451
799	UTS2	10	PLK1	0.189291	ADAM17	0.000104	CCL5	0.36451
800	UNG	10	EIF4E	0.189474	TPM2	0.000103	CARS	0.36451
801	UHRF2	10	RPA1	0.19	CDKN2A	0.000103	ZBTB5	0.364461
802	UBE2D3	10	APAF1	0.190476	B4GALT1	0.000103	TTLL5	0.364461
803	TLR4	10	EIF3J	0.190476	ADORA2A	0.000103	HNRPLL	0.364461
804	TGFBR2	10	MED12	0.190476	TGFB2	0.000102	DR1	0.364461
805	STX7	10	NUMA1	0.190476	RXRA	0.000102	CENPA	0.364461
806	STRN4	10	PDGFA	0.190476	BLVRB	0.000102	RSPH3	0.364412
807	STK24	10	PDGFB	0.190476	EIF4G3	0.000101	C15orf39	0.364412
808	SSRP1	10	PDPK1	0.190476	DDX3X	0.000101	BCAT1	0.364412
809	SNW1	10	SENP1	0.190476	S100A13	0.0001	ABHD2	0.364412
810	SNCG	10	TICAM1	0.190476	ACE2	0.0001	MEF2C	0.364364
811	SLX1A	10	TSSK6	0.190476	SNRNP70	0.000099	ITGB8	0.364364
812	S100A11	10	RPA2	0.190769	PRKCE	0.000099	FRYL	0.364364
813	RXRA	10	PSMB8	0.190842	JAK1	0.000099	DAZ4	0.364364
814	RUNX1	10	CDK2	0.192308	LUC7L2	0.000098	CSNK1G1	0.364364
815	RHOG	10	MYBL2	0.193333	LMNB1	0.000098	CHMP5	0.364364
816	RCOR3	10	ACTL6A	0.194444	HBEGF	0.000098	ZNF541	0.364315
817	RANBP3	10	EIF4B	0.194444	CSTA	0.000098	ZNF200	0.364315
818	RAD18	10	MEF2C	0.194444	ADAM10	0.000098	ZMYM1	0.364315
819	PTEN	10	PLA2G4A	0.194444	HNRNPM	0.000097	USP30	0.364315
820	PSEN2	10	RPLP1	0.194444	SUMO3	0.000096	UBN2	0.364315
821	PRKCZ	10	CKS1B	0.196739	ISYNA1	0.000096	SMS	0.364315
822	PRKAA1	10	CBX1	0.19697	FAM134A	0.000096	SMC6	0.364315
823	PPP2R2D	10	ETS1	0.19697	SLC9A3R2	0.000095	PARP10	0.364315
824	PPM1G	10	PTMA	0.19697	RPL18A	0.000095	NDUFAF3	0.364315
825	POLR3B	10	TANK	0.19697	PLSCR1	0.000095	METTL13	0.364315
826	PCBD1	10	TOP1	0.197802	NCAPD2	0.000095	HOMEZ	0.364315
827	PARK2	10	PPP2R1A	0.19943	EIF3J	0.000095	GABBR2	0.364315
828	PABPC1	10	ADAM10	0.2	STX1A	0.000094	FIGNL1	0.364315
829	P2RY1	10	ADAM17	0.2	B2M	0.000094	CYP51A1	0.364315
830	P2RY11	10	ANLN	0.2	DKC1	0.000093	CDV3	0.364315
831	OPRK1	10	AP1B1	0.2	TRADD	0.000092	C21orf59	0.364315
832	NOTCH1	10	ATG9A	0.2	SARS2	0.000091	ASB9	0.364315
833	NFKBIB	10	ATP2B1	0.2	GRK5	0.000091	AIFM2	0.364315
834	NEK6	10	BAD	0.2	FANCC	0.000091	KIF14	0.364267
835	MTDH	10	CBLL1	0.2	STMN2	0.00009	GART	0.364267
836	MAPT	10	CD9	0.2	NCOR2	0.00009	CEP350	0.364267
837	MAP3K5	10	CUX1	0.2	ITGB8	0.00009	ANKLE2	0.364267
838	LPAR4	10	DCP1B	0.2	C9orf78	0.00009	TUBA3D	0.364218
839	LAMC1	10	DDX24	0.2	C5orf25	0.00009	TSPY1	0.364218
840	KLC1	10	DERL1	0.2	L1CAM	0.000089	RAB3GAP2	0.364218
841	KHDRBS1	10	EIF3E	0.2	CLCN7	0.000089	HSDL2	0.364218
842	ITGB5	10	FGFR4	0.2	HSD17B4	0.000088	DALRD3	0.364218
843	IRS1	10	HBEGF	0.2	CUL4A	0.000088	CRTAP	0.364218
844	IFIT3	10	HIST1H2BK	0.2	TES	0.000087	ACOT7	0.364218
845	HTR2A	10	HLA-A	0.2	PRKCI	0.000087	TES	0.36417
846	HSD17B4	10	HLA-A	0.2	ORC1	0.000087	S100A13	0.36417
847	HRAS	10	HLA-B	0.2	EEF1D	0.000087	RIOK1	0.36417

848	HJURP	10	HLA-B	0.2	CXCL9	0.000087	ODF2L	0.36417
849	GNAQ	10	HLA-B	0.2	CLTA	0.000087	KPNA5	0.36417
850	GNAI3	10	HLA-B	0.2	CASP8AP2	0.000087	DNHD1	0.36417
851	GNAI2	10	HLA-B	0.2	AURKB	0.000087	CRYZ	0.36417
852	GHRL	10	HLA-B	0.2	WNK1	0.000086	CLGN	0.36417
853	F2RL2	10	HLA-B	0.2	NQO1	0.000086	CIRH1A	0.36417
854	EZR	10	HLA-B	0.2	ITGA6	0.000086	CAPZA3	0.36417
855	ELAVL1	10	HLA-B	0.2	ATXN3	0.000086	BLVRB	0.36417
856	EIF6	10	HLA-B	0.2	WTAP	0.000085	ZNHIT2	0.364121
857	EIF4G2	10	HLA-B	0.2	RRP12	0.000085	SPACA3	0.364121
858	DYNLL2	10	HLA-B	0.2	PDGFD	0.000085	PHF7	0.364121
859	DUSP15	10	HLA-B	0.2	MAST1	0.000085	MTFR1	0.364121
860	DTL	10	HLA-B	0.2	DIAPH3	0.000085	MGMT	0.364121
861	DRD3	10	HLA-B	0.2	SRSF9	0.000084	LZIC	0.364121
862	CUL5	10	HLA-B	0.2	RAG2	0.000084	LSS	0.364121
863	CUL2	10	HLA-B	0.2	PLCB2	0.000084	ITGB1	0.364121
864	COL6A1	10	HLA-B	0.2	NUP210	0.000084	HSPA14	0.364121
865	CDC7	10	HLA-B	0.2	GRIA1	0.000084	GALK1	0.364121
866	CCT4	10	HLA-B	0.2	FZR1	0.000084	FDXR	0.364121
867	BNIP3L	10	HLA-B	0.2	DBF4	0.000084	DHPS	0.364121
868	BCL2	10	HLA-B	0.2	CCNH	0.000084	DCXR	0.364121
869	ATP2B4	10	HLA-B	0.2	BCL2L1	0.000084	CKAP2	0.364121
870	ADRBK1	10	HLA-B	0.2	TAB1	0.000083	BTN3A3	0.364121
871	ZNF746	9	HLA-B	0.2	SMS	0.000083	ADAM10	0.364121
872	ZEB1	9	HLA-B	0.2	CAPZA3	0.000083	TSPY3	0.364072
873	UTS2D	9	HLA-B	0.2	UBR2	0.000082	SPAG1	0.364072
874	UBE2E3	9	HLA-B	0.2	IL3RA	0.000082	SPA17	0.364072
875	TTK	9	HLA-B	0.2	HYRC	0.000082	NANOG	0.364072
876	TRH	9	HLA-B	0.2	HSPA1L	0.000082	NAA11	0.364072
877	TRAF1	9	HLA-B	0.2	HMOX2	0.000082	MBOAT7	0.364072
878	TRADD	9	HLA-B	0.2	HLA-A	0.000082	LPIN2	0.364072
879	TPD52L1	9	HLA-C	0.2	BTBD2	0.000082	KPNA1	0.364072
880	TGM2	9	HLA-C	0.2	SLX1A	0.000081	KBTBD3	0.364072
881	SSTR3	9	HLA-C	0.2	RFWD2	0.000081	INTS7	0.364072
882	SSFA2	9	HLA-F	0.2	POU2F1	0.000081	G2E3	0.364072
883	SLX1B	9	HLA-G	0.2	PDPK1	0.000081	FAM83F	0.364072
884	SLC1A5	9	KDM1A	0.2	MDM4	0.000081	DCTN5	0.364072
885	SKIL	9	KLHL8	0.2	ETS1	0.000081	CTSL2	0.364072
886	SGK1	9	KRT8	0.2	DKKL1	0.000081	ARMC10	0.364072
887	RRP12	9	MEF2A	0.2	CSK	0.000081	ARL6	0.364072
888	RPLP1	9	MITF	0.2	CRKL	0.000081	ALG2	0.364072
889	RPL18A	9	NAPA	0.2	BCR	0.000081	USP6	0.364024
890	ROCK1	9	NCAM1	0.2	AMFR	0.000081	UBAP2	0.364024
891	RIPK1	9	NGFRAP1	0.2	ADRBK1	0.000081	TSPYL5	0.364024
892	RIBC2	9	PRDX1	0.2	ADA	0.000081	TDRD7	0.364024
893	RCC1	9	PRKCD	0.2	WDR62	0.00008	SV2A	0.364024
894	RABEP1	9	PTPN12	0.2	NEFM	0.00008	SLC25A31	0.364024
895	PYGL	9	PTPRO	0.2	NCOA1	0.00008	PRPS2	0.364024
896	PTPRE	9	RAG2	0.2	CDT1	0.00008	PDXDC1	0.364024
897	PTPRC	9	S100A7	0.2	TRIP6	0.000079	NUDT11	0.364024
898	PSIP1	9	SENP3	0.2	PIAS1	0.000079	FAM92A1	0.364024
899	PRPF40A	9	SKI	0.2	PAICS	0.000079	CLCN7	0.364024
900	PROKR2	9	SNW1	0.2	HNRNPA2B1	0.000079	CD276	0.364024
901	PROKR1	9	STX7	0.2	FANCM	0.000079	CCDC58	0.364024
902	PRKCB	9	TAF15	0.2	CNOT10	0.000079	ATP6V1E2	0.364024
903	PRKAR1A	9	TTI2	0.2	CDC16	0.000079	ARL6IP5	0.364024
904	PPP4C	9	VRK3	0.2	AP2A1	0.000079	ALDH1A2	0.364024
905	PPP2R2A	9	WDTC1	0.2	PSMD11	0.000078	AGPAT5	0.364024
906	PLA2G4A	9	XRCC4	0.2	MKRN1	0.000078	ZP3	0.363927
907	PDXK	9	RAE1	0.201016	CHD1L	0.000078	ZNF711	0.363927
908	OPN4	9	MCM4	0.201299	TUBB2A	0.000077	ZFHX4	0.363927
909	NUTF2	9	PSMA8	0.202016	RIPK1	0.000077	ZDHHC20	0.363927
910	NSMAF	9	CDKN1B	0.202279	RIBC2	0.000077	ZBTB39	0.363927
911	NRIP1	9	ERBB2	0.205128	POLA2	0.000077	YBX2	0.363927
912	NPW	9	TUBA4A	0.21164	NINL	0.000077	WDR54	0.363927
913	NMBR	9	DKC1	0.212121	MAD2L1	0.000077	TUBD1	0.363927

914	NMB	9	JAK1	0.212121	DUSP15	0.000077	TTLL4	0.363927
915	NFRKB	9	NCL	0.212121	UBQLN1	0.000076	TTC7B	0.363927
916	MTNR1A	9	HMGA1	0.213235	TAF15	0.000076	TTC33	0.363927
917	MEF2C	9	TOPBP1	0.213235	SEC61B	0.000076	TTC26	0.363927
918	MBD3	9	BIRC3	0.214286	PPFIA1	0.000076	TSEN34	0.363927
919	MAPK8IP2	9	DDX21	0.214286	NCL	0.000076	TPTE	0.363927
920	LPAR6	9	DERL2	0.214286	LSM3	0.000076	TMEM67	0.363927
921	LCK	9	DNAJA3	0.214286	IRS1	0.000076	TMEM57	0.363927
922	IFIT2	9	FOSL2	0.214286	GHRL	0.000076	TMEM192	0.363927
923	HRH4	9	IFIT5	0.214286	BID	0.000076	THYN1	0.363927
924	HRH1	9	LSM3	0.214286	AIFM1	0.000076	THUMPD3	0.363927
925	HMGB1	9	MAGEH1	0.214286	UBE2N	0.000075	TDRKH	0.363927
926	GSTM3	9	PTPN1	0.214286	NEK6	0.000075	TBC1D8B	0.363927
927	GRPR	9	RPS20	0.214286	ACTL6A	0.000075	SOAT1	0.363927
928	GRP	9	TCF4	0.214286	SERPINA5	0.000074	SLC45A4	0.363927
929	GPR68	9	CHEK1	0.215686	PTBP1	0.000074	SLC41A3	0.363927
930	GPR65	9	KPNA1	0.216374	PIM2	0.000074	SLC2A3	0.363927
931	GPR4	9	PSMA3	0.217204	GNAI1	0.000074	SLC2A14	0.363927
932	GNRHR	9	CASP1	0.218182	EIF4B	0.000074	SETD4	0.363927
933	GNRHR2	9	CCND1	0.218462	BAT3	0.000074	SESN3	0.363927
934	GNRH1	9	PTPN11	0.21978	UBE2E3	0.000073	SCO1	0.363927
935	GNAI1	9	CDC6	0.221344	TCERG1	0.000073	SCCPDH	0.363927
936	GNA14	9	CASP8AP2	0.222222	KLK6	0.000073	SAMD9	0.363927
937	GNA11	9	CASP9	0.222222	CST3	0.000073	S100PBP	0.363927
938	GINS1	9	COL1A2	0.222222	CASP7	0.000073	RNF19B	0.363927
939	GHSR	9	DNAJB1	0.222222	UFD1L	0.000072	RAB28	0.363927
940	GCDH	9	HRAS	0.222222	TRAF3	0.000072	PXDNL	0.363927
941	GADD45A	9	PABPC1	0.222222	TRAF3IP1	0.000072	PTDSS2	0.363927
942	GABBR2	9	PTPRC	0.222222	MPHOSPH8	0.000072	PSMG1	0.363927
943	GABBR1	9	VRK1	0.222222	DTL	0.000072	PRSS21	0.363927
944	G6PD	9	ZEB1	0.222222	ATP2B1	0.000072	PIGV	0.363927
945	FANCC	9	SNRNP200	0.22444	RPN2	0.000071	PGM3	0.363927
946	F2R	9	CCNE1	0.225071	RCC1	0.000071	PGK2	0.363927
947	F2RL3	9	POLR2D	0.226776	KIF2B	0.000071	PCYT2	0.363927
948	EXOSC8	9	GTF2B	0.227273	CSF2	0.000071	PAPOLB	0.363927
949	EIF4B	9	SOX9	0.227273	CCT6B	0.000071	ORMDL2	0.363927
950	EIF2C4	9	ARHGEF1	0.230769	UBE2D3	0.00007	NXT2	0.363927
951	EID1	9	RBM8A	0.234623	SVIL	0.00007	NSUN3	0.363927
952	EDNRB	9	U2AF2	0.234623	SPARC	0.00007	NSDHL	0.363927
953	EDNRA	9	PCNT	0.235098	SKIL	0.00007	NEK10	0.363927
954	DNAJB1	9	GTF2H3	0.235294	PTPRE	0.00007	NDRG3	0.363927
955	CXCR3	9	FOXM1	0.236364	MCM6	0.00007	MUM1L1	0.363927
956	CXCL9	9	TUBB	0.236667	ABCA1	0.00007	MTX2	0.363927
957	CXCL12	9	DYNLL1	0.237154	TUBB4	0.000069	MORC1	0.363927
958	CSNK2A2	9	BCL3	0.238095	SMAD1	0.000069	MFF	0.363927
959	COL1A2	9	CANX	0.238095	PXN	0.000069	MFAP3L	0.363927
960	COBRA1	9	CSK	0.238095	KRT7	0.000069	MEX3C	0.363927
961	CNR1	9	GNAS	0.238095	FLOT1	0.000069	METAP1	0.363927
962	CNOT10	9	HLA-E	0.238095	EPHA8	0.000069	MAPK8IP2	0.363927
963	CLTA	9	MYH10	0.238095	TSC1	0.000068	MAP3K11	0.363927
964	CLSPN	9	NCAPD2	0.238095	SRF	0.000068	MAGEB1	0.363927
965	CITED1	9	PTPRB	0.238095	PTH2R	0.000068	LZTFL1	0.363927
966	CHRM5	9	SF3A2	0.238295	MPHOSPH9	0.000068	LRWD1	0.363927
967	CHRM3	9	GTF2E2	0.241107	LAMA4	0.000068	LRRC37B	0.363927
968	CHRM2	9	TFDP1	0.241758	DSG1	0.000068	LMBRD2	0.363927
969	CHAF1B	9	TAF1	0.242424	BLM	0.000068	LCORL	0.363927
970	CCR10	9	GNA13	0.242647	ANAPC2	0.000068	KLHL7	0.363927
971	CCL21	9	DDX20	0.243011	AGTR1	0.000068	KLHL11	0.363927
972	CCL20	9	DTL	0.244444	TPP2	0.000067	KCTD7	0.363927
973	CCKBR	9	RAD18	0.244444	STRN4	0.000067	KBTBD4	0.363927
974	CASP9	9	PLK4	0.245305	PTHLH	0.000067	KATNAL1	0.363927
975	CASP8AP2	9	CEP76	0.245696	PRKAA2	0.000067	IMPACT	0.363927
976	CASP4	9	KALRN	0.247619	HELLS	0.000067	IL32	0.363927
977	AVPR1A	9	COBRA1	0.25	G2E3	0.000067	IL13RA2	0.363927
978	APLN	9	DDX42	0.25	APLP1	0.000067	IFT81	0.363927
979	AP2M1	9	EIF2S1	0.25	XPNPEP1	0.000066	IFT74	0.363927

980	AP2A2	9	IFIT2	0.25	VTN	0.000066	IFT52	0.363927
981	ANXA7	9	ITGB4	0.25	ROCK1	0.000066	IFT122	0.363927
982	AHSA1	9	KIT	0.25	LAMC3	0.000066	HN1	0.363927
983	ADRA1A	9	MAPK11	0.25	BDKRB2	0.000066	HINT3	0.363927
984	ACTL6A	9	NFRKB	0.25	WWTR1	0.000065	HENMT1	0.363927
985	ZNRF4	8	NRAS	0.25	TXNIP	0.000065	HAS3	0.363927
986	ZNF473	8	SMARCD1	0.25	THAP11	0.000065	HACL1	0.363927
987	WWTR1	8	STIP1	0.25	SYT1	0.000065	GPAT2	0.363927
988	WNK1	8	VDR	0.25	STX4	0.000065	FBRS	0.363927
989	WDR62	8	CUL1	0.252308	PTPRB	0.000065	FAM54A	0.363927
990	VDR	8	SHC1	0.254545	LDLR	0.000065	FAM184A	0.363927
991	UBE3A	8	CHD1L	0.25641	JUND	0.000065	EGFLAM	0.363927
992	TXN	8	XPO1	0.256757	PABPC1	0.000064	EFCAB11	0.363927
993	TSC1	8	SKP2	0.25731	NFKBIB	0.000064	DOPEY1	0.363927
994	TRAF3IP1	8	TNF	0.257576	GRIP1	0.000064	DOLK	0.363927
995	TOX4	8	CEP78	0.258799	WBSCR22	0.000063	DNAJC28	0.363927
996	TERF2IP	8	ODF2	0.258799	SLX1B	0.000063	DNAJC17	0.363927
997	TCF4	8	CDK7	0.259259	SLC2A1	0.000063	DNAAF1	0.363927
998	TADA2A	8	APPL1	0.266667	SH3BP4	0.000063	DHX35	0.363927
999	STIP1	8	DYX1C1	0.266667	RRBP1	0.000063	DHDDS	0.363927
1000	SSX2IP	8	EPHA8	0.266667	PRDX2	0.000063	DENND4C	0.363927

(b)

k-core			Community		
**gene symbols**	**uniprot ID**	***k-core***	**gene symbols**	**uniprot ID**	**index of comm.**
ZWILCH	Q9H900	17	ACIN1	Q9UKV3	1
YWHAZ	P63104	17	AIFM1	O95831	1
YWHAQ	P27348	17	AIMP2	Q13155	1
YWHAG	P61981	17	ALDH18A1	P54886	1
YWHAE	P62258	17	ALDOA	P04075	1
XRCC5	P13010	17	ANXA2	P07355	1
XPO1	O14980	17	ARFGEF1	Q9Y6D6	1
VIM	P08670	17	BAG4	O95429	1
VHL	P40337	17	BLMH	Q13867	1
UBE2I	P63279	17	BRF2	Q9HAW0	1
UBE2D1	P51668	17	C14orf166	Q9Y224	1
UBC	P0CG48	17	C15orf39	Q6ZRI6	1
UBB	P0CG47	17	CASP4	P49662	1
UBA52	P62987	17	CDC73	Q6P1J9	1
U2AF2	P26368	17	CDK5	Q00535	1
TUBG1	P23258	17	CHIC2	Q9UKJ5	1
TUBB	P07437	17	CIAO1	O76071	1
TUBB4	TUBB4	17	CNOT10	Q9H9A5	1
TUBA4A	P68366	17	CNOT7	Q9UIV1	1
TUBA1A	Q71U36	17	CPSF1	Q10570	1
TRIM28	Q13263	17	CPSF3	Q9UKF6	1
TRAF6	Q9Y4K3	17	CRTAP	O75718	1
TP53	P04637	17	CSTF3	Q12996	1
TOP2A	P11388	17	DAZ1	Q9NQZ3	1
TCP1	P17987	17	DAZ4	Q86SG3	1
TBK1	Q9UHD2	17	DAZL	Q92904	1
SUMO2	P61956	17	DCP1B	Q8IZD4	1
SUMO1	P63165	17	DDX20	Q9UHI6	1
SUGP1	Q8IWZ8	17	DDX21	Q9NR30	1
STUB1	Q9UNE7	17	DDX24	Q9GZR7	1
STAT1	P42224	17	DDX42	Q86XP3	1
SRSF9	Q13242	17	DFFA	O00273	1
SRSF1	Q07955	17	DFFB	O76075	1
SRRM2	Q9UQ35	17	DHX16	O60231	1
SNRPF	P62306	17	DHX9	Q08211	1
SNRPD3	P62318	17	DKC1	O60832	1
SNRPD1	P62314	17	DNAJA2	O60884	1
SNRPA1	P09661	17	DNAJC10	Q8IXB1	1
SNRNP200	O75643	17	DNAJC3	Q13217	1
SMN1	Q16637	17	DSG1	Q02413	1
SMARCC1	Q92922	17	EDC4	Q6P2E9	1
SMAD2	Q15796	17	EEA1	Q15075	1
SKP1	P63208	17	EFTUD2	Q15029	1
SIRT1	Q96EB6	17	EIF2B3	Q9NR50	1
SGOL2	Q562F6	17	EIF2S1	P05198	1
SF3A2	Q15428	17	EIF3E	P60228	1
RUVBL1	Q9Y265	17	EIF3J	O75822	1
RPS27A	P62979	17	EIF4B	P23588	1
RELA	Q04206	17	EIF4E	P06730	1
RBM8A	Q9Y5S9	17	EIF4G1	Q04637	1
RBL1	P28749	17	EIF4G2	P78344	1
RBBP4	Q09028	17	EIF4H	Q15056	1
RB1	P06400	17	EIF5	P55010	1

RAN	P62826	17	ELAVL1	Q15717	1
RANBP2	P49792	17	ELAVL2	Q12926	1
RAE1	P78406	17	EPB41	P11171	1
PSME3	P61289	17	ERO1L	Q96HE7	1
PSMD8	P48556	17	ERP29	P30040	1
PSMD6	Q15008	17	EXOSC8	Q96B26	1
PSMD4	P55036	17	FBL	P22087	1
PSMD10	O75832	17	GLE1	Q53GS7	1
PSMB8	P28062	17	GOLGA3	Q08378	1
PSMA8	Q8TAA3	17	GOPC	Q9HD26	1
PSMA5	P28066	17	GTF2B	Q00403	1
PSMA3	P25788	17	GTF2E2	P29084	1
PRPF8	Q6P2Q9	17	GTF2F1	P35269	1
PRMT5	O14744	17	GTF2F2	P13984	1
PPP2R1A	P30153	17	GTF3C4	Q9UKN8	1
PPP1CC	P36873	17	HDAC5	Q9UQL6	1
POLR2D	O15514	17	HDGF	P51858	1
PLK4	O00444	17	HELLS	Q9NRZ9	1
PLK1	P53350	17	HIST1H2BK	O60814	1
PCNT	O95613	17	HMGA1	P17096	1
PARP1	P09874	17	HNRNPA1	P09651	1
PAFAH1B1	P43034	17	HNRNPA2B1	P22626	1
ORC1	Q13415	17	HNRNPC	P07910	1
ODF2	Q5BJF6	17	HNRNPD	Q14103	1
NUP88	Q99567	17	HNRNPM	P52272	1
NUP62	P37198	17	HNRNPR	O43390	1
NUP35	Q8NFH5	17	HNRNPU	Q00839	1
NUP210	Q8TEM1	17	HNRPLL	Q8WVV9	1
NUP155	O75694	17	HSD17B10	Q99714	1
NUP153	P49790	17	HSP90B1	P14625	1
NUP107	P57740	17	HSPA1L	P34931	1
NUDC	Q9Y266	17	IFIT2	P09913	1
NFKB1	P19838	17	IFIT3	O14879	1
NEK2	P51955	17	ILF2	Q12905	1
NDEL1	Q9GZM8	17	ILF3	Q12906	1
NDE1	Q9NXR1	17	INTS6	Q9UL03	1
MYC	P01106	17	INTS7	Q9NVH2	1
MNAT1	P51948	17	KIAA0101	KIAA0101	1
MCM7	P33993	17	KLHL13	Q9P2N7	1
MCM5	P33992	17	KPNA1	P52294	1
MCM4	P33991	17	KPNA5	O15131	1
MAPRE1	Q15691	17	KPNB1	Q14974	1
MAP3K3	Q99759	17	KRI1	Q8N9T8	1
MAP1LC3B	Q9GZQ8	17	LSM3	P62310	1
MAP1LC3A	Q9H492	17	LSM4	Q9Y4Z0	1
MAD2L1	Q13257	17	LYAR	Q9NX58	1
LMNA	P02545	17	LZTS1	Q9Y250	1
KPNB1	Q14974	17	MAGEA6	P43360	1
KPNA2	P52292	17	MAGEB2	O15479	1
KIF2C	Q99661	17	MED12	Q93074	1
KIF2B	Q8N4N8	17	MED26	O95402	1
KIF18A	Q8NI77	17	MEPCE	Q7L2J0	1
KIAA0101	Q15004	17	MFAP1	P55081	1
JUN	P05412	17	MGMT	P16455	1
ITGB3BP	Q13352	17	MLF2	Q15773	1
INCENP	Q9NQS7	17	MYCBPAP	Q8TBZ2	1
IKBKG	Q9Y6K9	17	MYSM1	Q5VVJ2	1
IKBKE	Q14164	17	NANOG	Q9H9S0	1
IKBKB	O14920	17	NCL	P19338	1

HSPB1	P04792	17	NHP2	Q9NX24	1
HSPA5	P11021	17	NUP107	P57740	1
HSPA1A	P08107	17	NUP153	P49790	1
HSP90AA1	P07900	17	NUP155	O75694	1
HNRNPU	Q00839	17	NUP210	Q8TEM1	1
HNRNPD	Q14103	17	NUP35	Q8NFH5	1
HNRNPC	P07910	17	NUP62	P37198	1
HDAC5	Q9UQL6	17	NUP88	Q99567	1
HDAC2	Q92769	17	NUTF2	P61970	1
HDAC1	Q13547	17	OTUD5	Q96G74	1
GTF2F1	P35269	17	PABPC1	P11940	1
GRB2	P62993	17	PAICS	P22234	1
GMNN	O75496	17	PASK	Q96RG2	1
GAPDH	P04406	17	PCF11	O94913	1
GABARAP	O95166	17	PGAM5	Q96HS1	1
GABARAPL1	Q9H0R8	17	PLBD2	Q8NHP8	1
FZR1	Q9UM11	17	PLOD1	Q02809	1
FYN	P06241	17	POLR2D	O15514	1
FLNA	P21333	17	POLR3B	Q9NW08	1
ESR1	P03372	17	POLR3H	Q9Y535	1
EP300	Q09472	17	PPM1A	P35813	1
EGFR	P00533	17	PPP2CA	P67775	1
DYNC1H1	Q14204	17	PRMT5	O14744	1
DHX9	Q08211	17	PRPF19	Q9UMS4	1
DDX5	P17844	17	PRPF8	Q6P2Q9	1
DDX20	Q9UHI6	17	PRRC2A	P48634	1
DDX1	Q92499	17	PSIP1	O75475	1
CUL1	Q13616	17	PTBP1	P26599	1
CTNNB1	P35222	17	PTMA	P06454	1
CSTF3	Q12996	17	QKI	Q96PU8	1
CPSF3	Q9UKF6	17	QRICH2	Q9H0J4	1
CPSF1	Q10570	17	RAE1	P78406	1
COPS5	Q92905	17	RANBP1	P43487	1
CLTC	Q00610	17	RANBP2	P49792	1
CLASP1	Q7Z460	17	RANBP3	Q9H6Z4	1
CKS1B	P61024	17	RAN	P62826	1
CKAP5	Q14008	17	RBM39	Q14498	1
CHUK	O15111	17	RBM5	P52756	1
CEP78	Q5JTW2	17	RBM8A	Q9Y5S9	1
CEP76	Q8TAP6	17	RCC1	P18754	1
CENPN	Q96H22	17	REEP6	Q96HR9	1
CENPA	P49450	17	RGS20	O76081	1
CDT1	Q9H211	17	RPL18A	Q02543	1
CDKN1B	P46527	17	RPL26L1	Q9UNX3	1
CDKN1A	P38936	17	RPLP1	P05386	1
CDK2	P24941	17	RPN2	P04844	1
CDK1	P06493	17	RPS20	P60866	1
CDCA8	Q53HL2	17	RRP1B	Q14684	1
CDC27	P30260	17	S100A11	P31949	1
CDC25A	P30304	17	SF3A2	Q15428	1
CDC20	Q12834	17	SF3B3	Q15393	1
CCT8	P50990	17	SLBP	Q14493	1
CCT5	P48643	17	SMG1	Q96Q15	1
CCT2	P78371	17	SMN1	Q16637	1
CCNE1	P24864	17	SMN2	SMN2	1
CCND1	P24385	17	SMU1	Q2TAY7	1
CCNB1	P14635	17	SNAPC5	O75971	1
CCNA2	P20248	17	SNRNP200	O75643	1
CCNA1	P78396	17	SNRNP70	P08621	1

CBL	P22681	17	SNRPA1	P09661	1
CASP3	P42574	17	SNRPD1	P62314	1
CASC5	Q8NG31	17	SNRPD3	P62318	1
CALM1	P62158	17	SNRPF	P62306	1
BUB3	O43684	17	SRP54	P61011	1
BUB1	O43683	17	SRP72	O76094	1
BUB1B	O60566	17	SRRM2	Q9UQ35	1
BRCA1	P38398	17	SRSF1	Q07955	1
BIRC5	O15392	17	SRSF9	Q13242	1
AURKB	Q96GD4	17	SSB	P05455	1
ARRB2	P32121	17	SSR1	P43307	1
AR	P10275	17	SUGP1	Q8IWZ8	1
**APP**	**P05067**	**17**	SYNCRIP	O60506	1
APC	P25054	17	TADA2A	O75478	1
ACTB	P60709	17	TAF10	Q12962	1
UCHL1	P09936	16	TAF15	Q92804	1
UBE2E1	P51965	16	TAF1	P21675	1
TP63	Q9H3D4	16	TAF4	O00268	1
TP53BP1	Q12888	16	TAF9	Q16594	1
TFDP2	Q14188	16	TBP	P20226	1
TERF2	Q15554	16	TCERG1	O14776	1
SSB	P05455	16	TOP1	P11387	1
SRC	P12931	16	TPI1	P60174	1
SP1	P08047	16	TPX2	Q9ULW0	1
SNRNP70	P08621	16	TRA2B	P62995	1
SMC3	Q9UQE7	16	TRAF4	Q9BUZ4	1
SMAD3	P84022	16	TRAM1	Q15629	1
SKP2	Q13309	16	TSSC1	Q53HC9	1
RPA3	P35244	16	TUBA3D	Q13748	1
RPA2	P15927	16	U2AF2	P26368	1
RPA1	P27694	16	USP11	P51784	1
RFC4	P35249	16	USP30	Q70CQ3	1
RARA	P10276	16	VHL	P40337	1
RANBP1	P43487	16	VRK1	Q99986	1
RAD51	Q06609	16	VRK3	Q8IV63	1
RAD23B	P54727	16	WIBG	Q9BRP8	1
PSME1	Q06323	16	ZNF473	Q8WTR7	1
PSMD7	P51665	16	ABCB1	P08183	2
PSMD2	Q13200	16	ACBD3	Q9H3P7	2
PSMD12	O00232	16	ACE	P12821	2
PSMC6	P62333	16	ACHE	P22303	2
PSMC5	P62195	16	ADAM10	O14672	2
PSMB6	P28072	16	ADAM17	P78536	2
PSMA7	O14818	16	ADAM33	Q9BZ11	2
PSMA1	P25786	16	ADAM8	P78325	2
PRIM1	P49642	16	ADNP	Q9H2P0	2
PIK3CA	P42336	16	AGER	Q15109	2
PCNA	P12004	16	AGRN	O00468	2
ORC2	Q13416	16	ALDH1A2	O94788	2
NR3C1	P04150	16	ALG8	Q9BVK2	2
NGFR	P08138	16	APBA1	Q02410	2
NFKBIE	O00221	16	APBA2	Q99767	2
NBN	O60934	16	APCS	P02743	2
MYBL2	P10244	16	APMAP	Q9HDC9	2
MTA1	Q13330	16	APOA1BP	Q8NCW5	2
MSH6	P52701	16	APOA1	P02647	2
MSH2	P43246	16	APOA2	P02652	2
MEPCE	Q7L2J0	16	**APP**	**P05067**	**2**
MDM2	Q00987	16	ATF3	P18847	2

MCC	P23508	16	B2M	P61769	2
IRF3	Q14653	16	BACE1	P56817	2
ILF2	Q12905	16	BACE2	Q9Y5Z0	2
HSPH1	Q92598	16	BBS7	Q8IWZ6	2
HSPA8	P11142	16	BCAP29	Q9UHQ4	2
HSP90AB1	P08238	16	BCAP31	P51572	2
HSF1	Q00613	16	BCL3	P20749	2
HNRNPR	O43390	16	BGN	P21810	2
HNRNPM	P52272	16	BLVRB	P30043	2
HNRNPA1	P09651	16	CALR	P27797	2
HMGA1	P17096	16	CALU	O43852	2
HDGF	P51858	16	CAMP	P49913	2
H2AFX	P16104	16	CANX	P27824	2
GTF2F2	P13984	16	CAPZA2	P47755	2
GSK3B	P49841	16	CASP1	P29466	2
FBL	P22087	16	CASP8	Q14790	2
EWSR1	Q01844	16	CAT	P04040	2
EMD	P50402	16	CBFB	Q13951	2
E2F1	Q01094	16	CCHCR1	Q8TD31	2
DYNLL1	P63167	16	CD14	P08571	2
DNMT1	P26358	16	CD36	P16671	2
DBF4	Q9UBU7	16	CHRNA7	P36544	2
CREBBP	Q92793	16	CHRNB1	P11230	2
CHEK1	O14757	16	CLPTM1L	Q96KA5	2
CHD3	Q12873	16	CLSTN1	O94985	2
CDK9	P50750	16	CLSTN3	Q9BQT9	2
CDK7	P50613	16	CLU	P10909	2
CDH1	P12830	16	CNTN1	Q12860	2
CDC6	Q99741	16	CNTN3	Q9P232	2
CDC45	O75419	16	CNTN4	Q8IWV2	2
CDC16	Q13042	16	COL18A1	P39060	2
CCNH	P51946	16	COL1A2	P08123	2
BTRC	Q9Y297	16	COL25A1	Q9BXS0	2
BRCA2	P51587	16	COL4A1	P02462	2
BARD1	Q99728	16	COL4A2	P08572	2
ATR	Q13535	16	COL4A3	Q01955	2
ATM	Q13315	16	COL4A5	P29400	2
ANXA1	P04083	16	COL4A6	Q14031	2
ANAPC7	Q9UJX3	16	COL6A1	P12109	2
AKT1	P31749	16	COL6A3	P12111	2
TUBB2C	TUBB2C	15	COMT	P21964	2
TRAF2	Q12933	15	CPEB1	Q9BZB8	2
TOPBP1	Q92547	15	CPE	P16870	2
TMPO	P42166	15	CREB3	O43889	2
TAF10	Q12962	15	CRYAB	P02511	2
SUPT5H	O00267	15	CST3	P01034	2
SLBP	Q14493	15	CTSD	P07339	2
SIAH1	Q8IUQ4	15	CTSL2	O60911	2
SF3B3	Q15393	15	CTSS	P25774	2
RIF1	Q5UIP0	15	DCBLD1	Q8N8Z6	2
RBM5	P52756	15	DNAH1	Q9P2D7	2
RBL2	Q08999	15	DNM1L	O00429	2
PTGES3	Q15185	15	DPEP1	P16444	2
PTBP1	P26599	15	ECE1	P42892	2
PSMD1	Q99460	15	EFEMP1	Q12805	2
PSMD11	O00231	15	EPB41L3	Q9Y2J2	2
PSMC4	P43686	15	ERAP1	Q9NZ08	2
PSMC2	P35998	15	ERAP2	Q6P179	2
PSMB1	P20618	15	ERO1LB	Q86YB8	2

PSMA6	P60900	15	ERP44	Q9BS26	2
PSMA4	P25789	15	EXOC6	Q8TAG9	2
PSMA2	P25787	15	EXOC7	Q9UPT5	2
PPP2R2B	Q00005	15	F12	P00748	2
POLE	Q07864	15	F7	P08709	2
PML	P29590	15	FIS1	Q9Y3D6	2
PIN1	Q13526	15	FKBP1A	P62942	2
PIAS2	O75928	15	FLOT2	Q14254	2
PARK7	Q99497	15	FTL	P02792	2
ORC3	Q9UBD5	15	GFAP	P14136	2
NPM1	P06748	15	GNAO1	P09471	2
NFKBIA	P25963	15	GPC1	P35052	2
NFKB2	Q00653	15	GPNMB	Q14956	2
MYB	P10242	15	GSN	P06396	2
MAPK1	P28482	15	HADHB	P55084	2
MAP3K1	Q13233	15	HADH	Q16836	2
HSPA4	P34932	15	HLA-A	P01892	2
HNRNPA2B1	P22626	15	HLA-A	P04439	2
HLA-B	P30480	15	HLA-A	P18462	2
HDAC3	O15379	15	HLA-A	P30443	2
GTF2H3	Q13889	15	HLA-B	P01889	2
GTF2E2	P29084	15	HLA-B	P03989	2
FSCN1	Q16658	15	HLA-B	P10319	2
FBXO5	Q9UKT4	15	HLA-B	P18463	2
EIF1B	O60739	15	HLA-B	P18464	2
EFTUD2	Q15029	15	HLA-B	P18465	2
DCTN2	Q13561	15	HLA-B	P30460	2
DAXX	Q9UER7	15	HLA-B	P30461	2
CSNK2A1	P68400	15	HLA-B	P30462	2
CSE1L	P55060	15	HLA-B	P30464	2
CDK6	Q00534	15	HLA-B	P30466	2
CDC26	Q8NHZ8	15	HLA-B	P30475	2
CDC23	Q9UJX2	15	HLA-B	P30479	2
BAG2	O95816	15	HLA-B	P30481	2
ATF2	P15336	15	HLA-B	P30483	2
ARRB1	P49407	15	HLA-B	P30484	2
ANAPC5	Q9UJX4	15	HLA-B	P30485	2
ANAPC4	Q9UJX5	15	HLA-B	P30487	2
ANAPC2	Q9UJX6	15	HLA-B	P30488	2
ANAPC1	Q9H1A4	15	HLA-B	P30490	2
ANAPC11	Q9NYG5	15	HLA-B	P30491	2
AHR	P35869	15	HLA-B	P30492	2
ABL1	P00519	15	HLA-B	P30493	2
UBE2C	O00762	14	HLA-B	P30495	2
TRRAP	Q9Y4A5	14	HLA-B	P30498	2
TFDP1	Q14186	14	HLA-B	P30685	2
TFAP2A	P05549	14	HLA-B	Q04826	2
TBP	P20226	14	HLA-B	Q29718	2
TAF9	Q16594	14	HLA-B	Q29836	2
STAT3	P40763	14	HLA-B	Q29940	2
SMAD4	Q13485	14	HLA-B	Q31610	2
SAFB	Q15424	14	HLA-B	Q31612	2
RAD21	O60216	14	HLA-B	Q95365	2
PSMF1	Q92530	14	HLA-C	P04222	2
PSME2	Q9UL46	14	HLA-C	P10321	2
PSMD9	O00233	14	HLA-C	P30504	2
PSMD5	Q16401	14	HLA-C	P30508	2
PSMD3	O43242	14	HLA-C	Q29963	2
PSMD14	O00487	14	HLA-C	Q9TNN7	2

PSMD13	Q9UNM6	14	HLA-E	P13747	2
PSMC3	P17980	14	HLA-F	P30511	2
PSMC1	P62191	14	HLA-G	P17693	2
PSMB7	Q99436	14	HMOX1	P09601	2
PSMB5	P28074	14	HMOX2	P30519	2
PSMB4	P28070	14	HOMER2	Q9NSB8	2
PSMB3	P49720	14	HSPB6	O14558	2
PSMB2	P49721	14	HSPB8	Q9UJY1	2
PSMB10	P40306	14	HTRA1	Q92743	2
PRKDC	P78527	14	HTRA2	O43464	2
PRKACA	P17612	14	HYOU1	Q9Y4L1	2
PPP5C	P53041	14	IFITM1	P13164	2
PCF11	O94913	14	IL1B	P01584	2
PAK2	Q13177	14	IL6ST	P40189	2
ORC6	Q9Y5N6	14	ITGAM	P11215	2
OFD1	O75665	14	ITGB5	P18084	2
NFATC2	Q13469	14	ITM2A	O43736	2
MEF2D	Q14814	14	ITM2B	Q9Y287	2
MAPK8	P45983	14	KIAA0319L	Q8IZA0	2
MAP3K14	Q99558	14	KIAA1704	Q8IXQ4	2
MAP1B	P46821	14	KLK10	O43240	2
ELF3	P78545	14	KLK5	Q9Y337	2
EGR1	P18146	14	KLK6	Q92876	2
DHX30	Q7L2E3	14	LAMB1	P07942	2
DCTN1	Q14203	14	LAMC2	Q13753	2
CDC37	Q16543	14	LOXL2	Q9Y4K0	2
ANAPC10	Q9UM13	14	LRP1B	Q9NZR2	2
ULK2	Q8IYT8	13	LRP1	Q07954	2
UBE2K	P61086	13	LRP8	Q14114	2
TOP1	P11387	13	LYPD3	O95274	2
TERF1	P54274	13	MAG	P20916	2
TAB2	Q9NYJ8	13	MAP3K11	Q16584	2
STAT2	P52630	13	MAPK8IP1	Q9UQF2	2
SIKE1	Q9BRV8	13	MAT1A	Q00266	2
RRP1B	Q14684	13	MBOAT7	Q96N66	2
RFWD2	Q8NHY2	13	MBP	P02686	2
RBX1	P62877	13	MEFV	O15553	2
RACGAP1	Q9H0H5	13	MFI2	P08582	2
PSME4	Q14997	13	MMP17	Q9ULZ9	2
PRKAB1	Q9Y478	13	NAE1	Q13564	2
PPP2R1B	P30154	13	NCAM1	P13591	2
PPP2CA	P67775	13	NECAB3	Q96P71	2
PPP1CA	P62136	13	NF1	P21359	2
POT1	Q9NUX5	13	NFASC	O94856	2
POLE2	P56282	13	NLRP3	Q96P20	2
POLA2	Q14181	13	NUCB2	P80303	2
POLA1	P09884	13	OAT	P04181	2
PCM1	Q15154	13	OSTC	Q9NRP0	2
ORC4	O43929	13	P2RX7	Q99572	2
NEDD8	Q15843	13	PAK3	O75914	2
NCOR2	Q9Y618	13	PDGFA	P04085	2
MLH1	P40692	13	PDGFB	P01127	2
MED26	O95402	13	PDGFD	Q9GZP0	2
MCM3	P25205	13	PDIA3	P30101	2
MAPK14	Q16539	13	PDIA4	P13667	2
MAPK13	O15264	13	PDIA6	Q15084	2
MAP2K6	P52564	13	PDXDC1	Q6P996	2
LYAR	Q9NX58	13	PDZK1P1	A8MUH7	2
LGALS3BP	Q08380	13	PGAM1	P18669	2

KPNA1	P52294	13	PI4K2A	Q9BTU6	2
KAT5	Q92993	13	PITRM1	Q5JRX3	2
IQCB1	Q15051	13	PKD2	Q13563	2
IL7R	P16871	13	PLG	P00747	2
HSPD1	P10809	13	PLP2	Q04941	2
HSPA2	P54652	13	PLXNA1	Q9UIW2	2
GSTP1	P09211	13	PPID	Q08752	2
ERCC2	P18074	13	PPP3R2	Q96LZ3	2
ERCC1	P07992	13	PREP	P48147	2
EIF2AK2	P19525	13	PRPS2	P11908	2
E2F4	Q16254	13	PRPSAP1	Q14558	2
CSNK1D	P48730	13	PRSS1	P07477	2
CFLAR	O15519	13	PRSS2	P07478	2
CDC34	P49427	13	PRSS3	P35030	2
CD4	P01730	13	PRSSL1	PRSSL1	2
CBX3	Q13185	13	PSEN1	P49768	2
BCL6	P41182	13	PSEN2	P49810	2
AKAP9	Q99996	13	PTPLB	Q6Y1H2	2
YWHAB	P31946	12	Q14790	Q14790	2
VAV2	P52735	12	RELN	P78509	2
VAV1	P15498	12	SEC22C	Q9BRL7	2
UIMC1	Q96RL1	12	SLC40A1	Q9NP59	2
TUBGCP4	Q9UGJ1	12	SMUG1	Q53HV7	2
TRIO	O75962	12	SORL1	Q92673	2
TNFRSF1B	TNFRSF1B	12	SPATA24	Q86W54	2
TNFRSF1A	P19438	12	SPEF2	Q9C093	2
TAF4	O00268	12	SPON1	Q9HCB6	2
TAF1	P21675	12	SSPN	Q14714	2
SPTAN1	Q13813	12	STAR	P49675	2
SPAG9	O60271	12	STAT1	P42224	2
SNCA	P37840	12	STAT2	P52630	2
SET	Q01105	12	STIM1	Q13586	2
SAP130	Q9H0E3	12	SYNRG	Q9UMZ2	2
RUVBL2	Q9Y230	12	TAPBP	O15533	2
RLN3	Q8WXF3	12	TBC1D7	Q9P0N9	2
RIPK3	Q9Y572	12	TGM2	P21980	2
RHOA	P61586	12	TM2D1	Q9BX74	2
RFC1	P35251	12	TMCC2	O75069	2
RCN2	Q14257	12	TMEFF2	Q9UIK5	2
RAC1	P63000	12	TMEM191B	P0C7N4	2
PTK2	Q05397	12	TMEM191C	A6NGB0	2
PROK1	P58294	12	TMEM30A	Q9NV96	2
PRKD1	Q15139	12	TMEM30B	Q3MIR4	2
PRKCD	Q05655	12	TMPRSS12	Q86WS5	2
PPP2R4	Q15257	12	TNFRSF21	O75509	2
PLCB3	Q01970	12	TOMM5	Q8N4H5	2
PIK3R3	Q92569	12	TRIP12	Q14669	2
PIK3R2	O00459	12	TSPAN6	O43657	2
PIK3R1	P27986	12	TXNDC2	Q86VQ3	2
PIK3CG	P48736	12	UBE2M	P61081	2
PIAS1	O75925	12	VAMP1	P23763	2
PDGFRB	P09619	12	VAMP3	Q15836	2
ORC5	O43913	12	ZPBP	Q9BS86	2
NR5A1	Q13285	12	AARS	P49588	3
NMUR2	Q9GZQ4	12	AASDHPPT	Q9NRN7	3
NMUR1	Q9HB89	12	ABHD2	P08910	3
NMU	P48645	12	ACOT7	O00154	3
NINL	Q9Y2I6	12	ACSL5	Q9ULC5	3
NEDD1	Q8NHV4	12	ACTB	P60709	3

NCK1	P16333	12	ACTC1	P68032	3
NASP	P49321	12	ACTG1	P63261	3
MSN	P26038	12	ADAMTSL2	Q86TH1	3
MPRIP	Q6WCQ1	12	ADO	Q96SZ5	3
MCM8	Q9UJA3	12	AGPAT5	Q9NUQ2	3
MCM6	Q14566	12	AGPS	O00116	3
MCM2	P49736	12	AHR	P35869	3
MCHR2	Q969V1	12	AHSA1	O95433	3
MCHR1	Q99705	12	AKR1B1	P15121	3
MCF2	P10911	12	AKT1	P31749	3
MBD1	Q9UIS9	12	ALDOC	P09972	3
MAP3K7	O43318	12	ALG2	Q9H553	3
LPAR2	Q9HBW0	12	ALS2CR11	Q53TS8	3
LPAR1	Q92633	12	ANGEL1	Q9UNK9	3
KTN1	Q86UP2	12	ANK2	Q01484	3
KALRN	O60229	12	ANKIB1	Q9P2G1	3
JAK2	O60674	12	ANKLE2	Q86XL3	3
HPRT1	P00492	12	ANLN	Q9NQW6	3
GTF2B	Q00403	12	ANXA7	P20073	3
GNG2	P59768	12	AP2A2	O94973	3
GNB1	P62873	12	AP2B1	P63010	3
GNA15	P30679	12	AP2M1	Q96CW1	3
GNA13	Q14344	12	APAF1	O14727	3
GNA12	Q03113	12	APEX1	P27695	3
FPR2	P25090	12	APLP1	P51693	3
FOS	P01100	12	APOB	P04114	3
FKBP4	Q02790	12	ARL15	Q9NXU5	3
FGFR1OP	O95684	12	ARMC10	Q8N2F6	3
FANCI	Q9NVI1	12	AR	P10275	3
FANCA	O15360	12	ARRB1	P49407	3
ESR2	Q92731	12	ARRB2	P32121	3
EPB41	P11171	12	ATCAY	Q86WG3	3
EDN1	P05305	12	ATF2	P15336	3
ECT2	Q9H8V3	12	ATG9A	Q7Z3C6	3
DYNC1I2	Q13409	12	ATP8B3	O60423	3
DNMT3A	Q9Y6K1	12	ATXN1	P54253	3
DIABLO	Q9NR28	12	ATXN3	P54252	3
DEPDC7	Q96QD5	12	BABAM1	Q9NWV8	3
DDB1	Q16531	12	BAD	Q92934	3
DCC	P43146	12	BAG1	Q99933	3
DAB2	P98082	12	BAG2	O95816	3
CXCR4	P61073	12	BAG3	O95817	3
CUL4B	Q13620	12	BAG6	P46379	3
CSNK1E	P49674	12	BAP1	Q92560	3
CREM	Q03060	12	BAT3	P46379	3
CREB1	P16220	12	BAX	Q07812	3
CPSF6	Q16630	12	BAZ1B	Q9UIG0	3
COPS6	Q7L5N1	12	BCL2L1	Q07817	3
COIL	P38432	12	BCL2	P10415	3
CETN2	P41208	12	BECN1	Q14457	3
CEP70	Q8NHQ1	12	BEX1	Q9HBH7	3
CEP63	Q96MT8	12	BHLHE40	O14503	3
CEP57	Q86XR8	12	BID	P55957	3
CEP250	Q9BV73	12	BING2	Q9UER7	3
CDKN2A	P42771	12	BIRC3	Q13489	3
CDH2	P19022	12	BMPR1B	O00238	3
CDC42	P60953	12	BNIP3L	O60238	3
CD44	P16070	12	BPGM	P07738	3
CCP110	O43303	12	BRAF	P15056	3

CCNE2	O96020	12	BRD7	Q9NPI1	3
CCL27	Q9Y4X3	12	BRPF1	P55201	3
BDKRB2	P30411	12	BRSK1	Q8TDC3	3
BDKRB1	P46663	12	BTBD2	Q9BX70	3
BCAR1	P56945	12	BTN3A3	O00478	3
AZI1	Q9UPN4	12	C12orf10	Q9HB07	3
ARHGEF1	Q92888	12	C13orf27	Q5JUR7	3
ARHGEF12	Q9NZN5	12	C14orf1	Q9UKR5	3
ARHGEF11	O15085	12	C15orf23	Q9Y448	3
ARHGAP19	Q14CB8	12	C16orf78	Q8WTQ4	3
AGTR1	P30556	12	C17orf53	Q8N3J3	3
AGT	P01019	12	C18orf21	Q32NC0	3
ADORA3	P33765	12	C19orf42	Q9BQ49	3
ACTR1A	P61163	12	C19orf51	Q8N9W5	3
ACLY	P53396	12	C19orf62	Q9NWV8	3
XIAP	P98170	11	C1D	Q13901	3
VWF	P04275	11	C1orf112	Q9NSG2	3
USP7	Q93009	11	C1orf173	Q5RHP9	3
USP1	O94782	11	C20orf111	Q9NX31	3
USP11	P51784	11	C2CD2	Q9Y426	3
UNC119	Q13432	11	C5orf25	Q8NDZ2	3
UCHL5	Q9Y5K5	11	C5orf35	Q8NE22	3
TUBGCP6	Q96RT7	11	C7orf64	Q5RL73	3
TUBGCP5	Q96RT8	11	C9orf78	Q9NZ63	3
TUBGCP3	Q96CW5	11	CACNA1B	Q00975	3
TUBGCP2	Q9BSJ2	11	CACNA2D2	Q9NY47	3
TUBG2	Q9NRH3	11	CACNB3	P54284	3
TSGA14	TSGA14	11	CACNB4	O00305	3
TPX2	Q9ULW0	11	CACNG2	Q9Y698	3
TNF	P01375	11	CACNG4	Q9UBN1	3
TKT	P29401	11	CAD	P27708	3
SYK	P43405	11	CAMK1	Q14012	3
SUMO3	P55854	11	CAPZA3	Q96KX2	3
STAM2	O75886	11	CARD11	Q9BXL7	3
ST13	P50502	11	CARS	P49589	3
SSR1	P43307	11	CASP10	Q92851	3
SSNA1	O43805	11	CASP2	P42575	3
SQSTM1	Q13501	11	CASP6	P55212	3
SMN2	SMN2	11	CASP7	P55210	3
SLC9A3R1	O14745	11	CASP8AP2	Q9UKL3	3
SFI1	A8K8P3	11	CASP9	P55211	3
SDCCAG8	Q86SQ7	11	CATSPERG	Q6ZRH7	3
SAE1	Q9UBE0	11	CCDC42	Q96M95	3
RPL26L1	Q9UNX3	11	CCDC58	Q4VC31	3
RIPK2	O43353	11	CCDC86	Q9H6F5	3
RECQL5	O94762	11	CCT2	P78371	3
PTTG1	O95997	11	CCT4	P50991	3
PTPN11	Q06124	11	CCT5	P48643	3
PTMA	P06454	11	CCT6B	Q92526	3
PTAFR	P25105	11	CCT7	Q99832	3
PRKAR2B	P31323	11	CCT8	P50990	3
PRIM2	P49643	11	CDC34	P49427	3
PMCH	P20382	11	CDC37	Q16543	3
PLCB2	Q00722	11	CDK16	Q00536	3
PLCB1	Q9NQ66	11	CDK5R1	Q15078	3
MCM10	Q7L590	11	CDKN3	Q16667	3
MAPKAPK5	Q8IW41	11	CETN3	O15182	3
MAPK9	P45984	11	CFLAR	O15519	3
LPAR3	Q9UBY5	11	CGRRF1	Q99675	3

LMNB1	P20700	11	CHD3	Q12873	3
JAK1	P23458	11	CHUK	O15111	3
HUWE1	Q7Z6Z7	11	CIAPIN1	Q6FI81	3
HTT	P42858	11	CIITA	P33076	3
HAUS2	Q9NVX0	11	CIRH1A	Q969X6	3
GABARAPL2	P60520	11	CITED1	Q99966	3
FHL1	Q13642	11	CKAP2	Q8WWK9	3
FAF1	Q9UNN5	11	CLTC	Q00610	3
ERBB2	P04626	11	CLUAP1	Q96AJ1	3
EIF4E	P06730	11	COPS3	Q9UNS2	3
DKC1	O60832	11	COX7A2	P14406	3
DGKE	P52429	11	CPLX1	O14810	3
DCTN3	O75935	11	CPSF6	Q16630	3
CSNK2B	P67870	11	CPT1A	P50416	3
CSNK1A1	P48729	11	CREB1	P16220	3
CRK	P46108	11	CREBBP	Q92793	3
CNTRL	Q7Z7A1	11	CREM	Q03060	3
CKS2	P33552	11	CRYBB2	P43320	3
CEP72	Q9P209	11	CRYGC	P07315	3
CEP290	O15078	11	CRYZL1	O95825	3
CEP192	Q8TEP8	11	CRYZ	Q08257	3
CEP164	Q9UPV0	11	CSE1L	P55060	3
CEP152	O94986	11	CSNK1G1	Q9HCP0	3
CEP135	Q66GS9	11	CSNK1G3	Q9Y6M4	3
CENPJ	Q9HC77	11	CSNK2A1	P68400	3
CDKN2C	P42773	11	CSNK2A2	P19784	3
CDK5RAP2	Q96SN8	11	CSTA	P01040	3
CCNB2	O95067	11	CSTB	P04080	3
CASP7	P55210	11	CTAG2	O75638	3
CASP6	P55212	11	CTGF	P29279	3
BLM	P54132	11	CTSB	P07858	3
BAZ1B	Q9UIG0	11	CTSH	P09668	3
ARHGAP28	Q9P2N2	11	CTTN	Q14247	3
ANK2	Q01484	11	CYCS	P99999	3
ALMS1	Q8TCU4	11	CYP51A1	Q16850	3
AGTR2	P50052	11	DAB2IP	Q5VWQ8	3
YY1AP1	Q9H869	10	DAB2	P98082	3
XPO5	Q9HAV4	10	DALRD3	Q5D0E6	3
VCP	P55072	10	DAPP1	Q9UN19	3
UTS2	O95399	10	DAXX	Q9UER7	3
UHRF2	Q96PU4	10	DAZ2	Q13117	3
UBXN7	O94888	10	DAZ3	Q9NR90	3
UBE2T	Q9NPD8	10	DAZAP1	Q96EP5	3
TXNRD1	Q16881	10	DBN1	Q16643	3
TSC22D1	Q15714	10	DCXR	Q7Z4W1	3
TRAF3	Q13114	10	DDIT3	P35638	3
TP73	O15350	10	DDX19B	Q9UMR2	3
TNIK	Q9UKE5	10	DDX1	Q92499	3
TK1	P04183	10	DDX4	Q9NQI0	3
TAP1	Q03518	10	DDX5	P17844	3
TANK	Q92844	10	DENND4C	Q5VZ89	3
SYVN1	Q86TM6	10	DGCR8	Q8WYQ5	3
STK11	Q15831	10	DHDDS	Q86SQ9	3
SSRP1	Q08945	10	DHPS	P49366	3
SOX9	P48436	10	DHX30	Q7L2E3	3
SOD1	P00441	10	DHX35	DHX35	3
SNW1	Q13573	10	DIAPH3	Q9NSV4	3
SMC4	Q9NTJ3	10	DIP2A	Q14689	3
SLX4	Q8IY92	10	DMWD	Q09019	3

SEPT2	Q15019	10	DNAAF1	Q8NEP3	3
SEC61B	P60468	10	DNAJB1	P25685	3
SDHA	P31040	10	DNAJB4	Q9UDY4	3
S100A11	P31949	10	DNAJB6	O75190	3
RXRA	P19793	10	DNAJC17	Q9NVM6	3
RTN4	Q9NQC3	10	DNAJC1	Q96KC8	3
RHOG	P84095	10	DNAJC28	Q9NX36	3
RDX	P35241	10	DNAJC5	Q9H3Z4	3
RAF1	P04049	10	DNM2	P50570	3
PSEN1	P49768	10	DNMT1	P26358	3
POU2F1	P14859	10	DNMT3A	Q9Y6K1	3
POGZ	Q7Z3K3	10	DOC2A	Q14183	3
PLCG1	P19174	10	DOC2B	Q14184	3
PDIA3	P30101	10	DOLK	Q9UPQ8	3
P2RY1	P47900	10	DOPEY1	Q5JWR5	3
P2RY11	Q96G91	10	DPH1	Q9BZG8	3
OPRK1	P41145	10	DR1	Q01658	3
OIP5	O43482	10	DVL1	O14640	3
NPSR1	Q6W5P4	10	DVL2	O14641	3
NFYA	P23511	10	DVL3	Q92997	3
NFKBIB	Q15653	10	DYRK2	Q92630	3
NEDD4	P46934	10	DYRK3	O43781	3
NCOA6	Q14686	10	DYX1C1	Q8WXU2	3
NCOA2	Q15596	10	ECM1	Q16610	3
NCOA1	Q15788	10	EEF1D	P29692	3
NCL	P19338	10	EFCAB11	Q9BUY7	3
MYH9	P35579	10	EGFLAM	Q63HQ2	3
MTDH	Q86UE4	10	EGR1	P18146	3
MOB4	Q9Y3A3	10	EGR2	P11161	3
MME	P08473	10	EID1	Q9Y6B2	3
MGMT	P16455	10	EIF2AK2	P19525	3
MAPK3	P27361	10	EIF2AK3	Q9NZJ5	3
MAGI1	Q96QZ7	10	EIF2C4	Q9HCK5	3
MAGEB2	O15479	10	EIF4EBP1	Q13541	3
LYN	P07948	10	EIF4G3	O43432	3
LRPAP1	P30533	10	ELN	P15502	3
LPAR4	Q99677	10	EP300	Q09472	3
LGALS1	P09382	10	EPAS1	Q99814	3
KRT18	P05783	10	EPHA10	EPHA10	3
KIF15	Q9NS87	10	EPM2A	O95278	3
KIF11	P52732	10	ERLIN2	O94905	3
KIAA1377	Q9P2H0	10	ESR1	P03372	3
INSL3	P51460	10	ESR2	Q92731	3
HTR2A	P28223	10	ETFB	P38117	3
HMGCS1	Q01581	10	ETS1	P14921	3
HMGCR	P04035	10	EWSR1	Q01844	3
HLA-C	P30504	10	EZH2	Q15910	3
HJURP	Q8NCD3	10	F13A1	P00488	3
HIF1A	Q16665	10	F8	P00451	3
HERPUD1	Q15011	10	FABP7	O15540	3
GNAQ	P50148	10	FAM134A	Q8NC44	3
FXR2	P51116	10	FAM135A	Q9P2D6	3
FOXM1	Q08050	10	FAM184A	Q8NB25	3
FN1	P02751	10	FAM48A	Q8NEM7	3
FBLN1	P23142	10	FAM54A	FAM54A	3
F2RL2	O00254	10	FAM83F	Q8NEG4	3
F2RL1	P55085	10	FAM98A	Q8NCA5	3
ETFA	P13804	10	FBLN1	P23142	3
EEF1D	P29692	10	FBRS	Q9HAH7	3

DUSP15	Q9H1R2	10	FBXO25	Q8TCJ0	3
DTNBP1	Q96EV8	10	FBXO27	Q8NI29	3
DRD3	P35462	10	FBXO2	Q9UK22	3
DNAJB6	O75190	10	FBXO7	Q9Y3I1	3
CXCL10	P02778	10	FCHSD1	Q86WN1	3
CSTB	P04080	10	FCHSD2	O94868	3
CIAO1	O76071	10	FDXR	P22570	3
CHD1L	Q86WJ1	10	FHL1	Q13642	3
CCL5	P13501	10	FHL5	Q5TD97	3
CBX1	P83916	10	FKBP4	Q02790	3
CAV1	Q03135	10	FKBP6	O75344	3
C14orf1	Q9UKR5	10	FLNA	P21333	3
BRF2	Q9HAW0	10	FOS	P01100	3
BRD7	Q9NPI1	10	FOXO1	Q12778	3
BCAP31	P51572	10	FRYL	O94915	3
AXIN1	O15169	10	FTH1	P02794	3
ATN1	P54259	10	FXR2	P51116	3
ARHGDIA	P52565	10	G2E3	Q7L622	3
APH1B	Q8WW43	10	GABARAPL1	Q9H0R8	3
AMFR	Q9UKV5	10	GABARAPL2	P60520	3
AKAP8L	Q9ULX6	10	GABARAP	O95166	3
ADRBK1	P25098	10	GADD45A	P24522	3
ADORA2A	P29274	10	GADD45G	O95257	3
ACTG1	P63261	10	GALK1	P51570	3
ZBTB32	Q9Y2Y4	9	GAPDH	P04406	3
YES1	P07947	9	GAPVD1	Q14C86	3
WDR61	Q9GZS3	9	GART	P22102	3
WDR5	P61964	9	GAS7	O60861	3
VCAN	P13611	9	GFI1	Q99684	3
UTS2D	Q765I0	9	GLO1	Q04760	3
USP2	O75604	9	GOSR1	O95249	3
UBE2D2	P62837	9	GPAT2	Q6NUI2	3
TTK	P33981	9	GPR37	O15354	3
TTI2	Q6NXR4	9	GRIA1	P42261	3
TRH	P20396	9	GRIA2	P42262	3
TRAF1	Q13077	9	GRIA3	P42263	3
TRADD	Q15628	9	GRIA4	P48058	3
TGFBR1	P36897	9	GRIP1	Q9Y3R0	3
TARDBP	Q13148	9	GRIP2	Q9C0E4	3
TAP2	Q03519	9	GSK3A	P49840	3
TAB1	Q15750	9	GSS	P48637	3
STXBP1	P61764	9	GSTP1	P09211	3
STK24	Q9Y6E0	9	GUCY1A3	Q02108	3
SSTR3	P32745	9	GUCY1B3	Q02153	3
SREBF2	Q12772	9	HACL1	Q9UJ83	3
SLX1B	SLX1B	9	HAS3	O00219	3
SLX1A	Q9BQ83	9	HBXIP	O43504	3
SHC1	P29353	9	HDAC1	Q13547	3
SH3KBP1	Q96B97	9	HDAC2	Q92769	3
SH3GLB1	Q9Y371	9	HDAC3	O15379	3
SH3GL3	Q99963	9	HDAC6	Q9UBN7	3
SGK1	O00141	9	HENMT1	Q5T8I9	3
S100A6	P06703	9	HES1	Q14469	3
RUNX1	Q01196	9	HIF1A	Q16665	3
ROCK1	Q13464	9	HINT3	Q9NQE9	3
RNF10	Q8N5U6	9	HIP1	O00291	3
RFWD3	Q6PCD5	9	HIST1H2AG	P0C0S8	3
RAG1	P15918	9	HMGB1	P09429	3
RAD18	Q9NS91	9	HMGCR	P04035	3

PTPRE	P23469	9	HMGCS1	Q01581	3
PTEN	P60484	9	HMGN1	P05114	3
PSIP1	O75475	9	HMGN3	Q15651	3
PRPF40A	O75400	9	HN1	Q9UK76	3
PROKR2	Q8NFJ6	9	HOOK2	Q96ED9	3
PROKR1	Q8TCW9	9	HSBP1	O75506	3
PRKCZ	Q05513	9	HSD17B4	P51659	3
PRKCB	P05771	9	HSDL2	Q6YN16	3
PPP2R2D	Q66LE6	9	HSF1	Q00613	3
PPP2R2A	P63151	9	HSP90AB1	P08238	3
POMC	P01189	9	HSPA14	Q0VDF9	3
PLA2G4A	P47712	9	HSPA1A	P08107	3
PDXK	O00764	9	HSPA1B	P08107	3
PAXIP1	Q6ZW49	9	HSPA2	P54652	3
OPN4	Q9UHM6	9	HSPA4	P34932	3
NSMAF	Q92636	9	HSPA5	P11021	3
NSF	P46459	9	HSPA8	P11142	3
NSFL1C	Q9UNZ2	9	HSPB1	P04792	3
NRIP1	P48552	9	HSPBP1	Q9NZL4	3
NPW	Q8N729	9	HSPD1	P10809	3
NOTCH1	P46531	9	HSPE1	P61604	3
NMBR	P28336	9	HSPH1	Q92598	3
NMB	P08949	9	HTR3A	P46098	3
MVP	Q14764	9	HTT	P42858	3
MTNR1A	P48039	9	ID2	Q02363	3
MEF2C	Q06413	9	IFT122	Q9HBG6	3
MBD3	O95983	9	IFT52	Q9Y366	3
MAPT	P10636	9	IFT57	Q9NWB7	3
MAP3K5	Q99683	9	IFT74	Q96LB3	3
MAGED1	Q9Y5V3	9	IFT81	Q8WYA0	3
LRIF1	Q5T3J3	9	IGSF21	Q96ID5	3
LPAR6	P43657	9	IKBKB	O14920	3
LONP1	P36776	9	IKBKE	Q14164	3
KHDRBS1	Q07666	9	IKBKG	Q9Y6K9	3
ITCH	Q96J02	9	IL13RA2	Q14627	3
IRS1	P35568	9	IL32	P24001	3
IL4R	P24394	9	IMPACT	Q9P2X3	3
IFIT3	O14879	9	INSIG2	Q9Y5U4	3
IFIT2	P09913	9	IQCB1	Q15051	3
HRH4	Q9H3N8	9	IQCG	Q9H095	3
HRH1	P35367	9	IRAK1	P51617	3
HMGB1	P09429	9	IRF1	P10914	3
HGS	O14964	9	IRF3	Q14653	3
HDAC6	Q9UBN7	9	ISYNA1	Q9NPH2	3
GRPR	P30550	9	ITCH	Q96J02	3
GRP	P07492	9	JPH1	Q9HDC5	3
GPR68	Q15743	9	KARS	Q15046	3
GPR65	Q8IYL9	9	KATNAL1	Q9BW62	3
GPR4	P46093	9	KBTBD3	Q8NAB2	3
GNRHR	P30968	9	KBTBD4	Q9NVX7	3
GNRHR2	Q96P88	9	KCNE3	Q9Y6H6	3
GNRH1	P01148	9	KCNIP3	Q9Y2W7	3
GNB2L1	P63244	9	KCTD7	Q96MP8	3
GNAI2	P04899	9	KDM1A	O60341	3
GNA14	O95837	9	KHDRBS1	Q07666	3
GNA11	P29992	9	KIF14	Q15058	3
GINS1	Q14691	9	KLHL11	Q9NVR0	3
GHSR	Q92847	9	KLHL7	Q8IXQ5	3
GHRL	Q9UBU3	9	KPNA2	P52292	3

GADD45A	P24522	9	KRT15	P19012	3
GABBR2	O75899	9	KRT8	P05787	3
GABBR1	Q9UBS5	9	KTN1	Q86UP2	3
FBXO7	Q9Y3I1	9	LAMP2	P13473	3
FANCC	Q00597	9	LCORL	Q8N3X6	3
EZR	P15311	9	LDLRAP1	Q5SW96	3
EZH2	Q15910	9	LDLR	P01130	3
ETS1	P14921	9	LGALS3BP	Q08380	3
ELAVL1	Q15717	9	LLGL1	Q15334	3
EDNRB	P24530	9	LMBRD2	LMBRD2	3
EDNRA	P25101	9	LONP1	P36776	3
DTL	Q9NZJ0	9	LRIF1	Q5T3J3	3
DAZAP1	Q96EP5	9	LRMP	Q12912	3
CXCR3	P49682	9	LRP2	P98164	3
CXCL9	Q07325	9	LRP6	O75581	3
CXCL12	P48061	9	LRPAP1	P30533	3
CUL4A	Q13619	9	LRRC37B	Q96QE4	3
COBRA1	Q8WX92	9	LRRK2	Q5S007	3
CNR1	P21554	9	LRWD1	Q9UFC0	3
CLSPN	Q9HAW4	9	LSS	P48449	3
CHRM5	P08912	9	LTBP4	Q8N2S1	3
CHRM3	P20309	9	LUC7L2	Q9Y383	3
CHRM2	P08172	9	LYL1	P12980	3
CHAF1B	Q13112	9	LZIC	Q8WZA0	3
CDC7	O00311	9	LZTFL1	Q9NQ48	3
CCT4	P50991	9	MAF	O75444	3
CCR10	P46092	9	MAGEB1	MAGEB1	3
CCL21	O00585	9	MAGEH1	Q9H213	3
CCL20	P78556	9	MALT1	Q9UDY8	3
CCKBR	P32239	9	MAP1B	P46821	3
CASP8AP2	Q9UKL3	9	MAP1LC3A	Q9H492	3
CASP1	P29466	9	MAP1LC3B	Q9GZQ8	3
BCL2	P10415	9	MAP1S	Q66K74	3
AVPR1A	P37288	9	MAP2K1	Q02750	3
ATXN1	P54253	9	MAP3K14	Q99558	3
ATG5	Q9H1Y0	9	MAP3K1	Q13233	3
ADRA1A	P35348	9	MAP3K2	Q9Y2U5	3
ACAT2	Q9BWD1	9	MAP3K3	Q99759	3
ZNF473	Q8WTR7	8	MAP3K8	P41279	3
ZEB1	P37275	8	MAP7	Q14244	3
WIPI2	Q9Y4P8	8	MAPK13	O15264	3
WDR62	O43379	8	MAPK3	P27361	3
VRK1	Q99986	8	MAPKAPK2	P49137	3
UBE2U	Q5VVX9	8	MAVS	Q7Z434	3
UBE2E3	Q969T4	8	MAX	P61244	3
TOX4	O94842	8	MBD1	Q9UIS9	3
TGFBR2	P37173	8	MBD3	O95983	3
TEX10	Q9NXF1	8	MDH1	P40925	3
TERF2IP	Q9NYB0	8	MDM2	Q00987	3
SVIL	O95425	8	MDM4	O15151	3
STRN4	Q9NRL3	8	MED31	Q9Y3C7	3
SSX2IP	Q9Y2D8	8	MEF2D	Q14814	3
SSTR2	P30874	8	MEN1	O00255	3
SSFA2	P28290	8	METAP1	P53582	3
SNCG	O76070	8	MEX3B	Q6ZN04	3
SMARCD1	Q96GM5	8	MEX3C	Q5U5Q3	3
SFN	P31947	8	MFAP3L	O75121	3
S1PR5	Q9H228	8	MFAP5	Q13361	3
S1PR1	P21453	8	MFF	Q9GZY8	3

RXFP4	Q8TDU9	8	MIB1	Q86YT6	3
RXFP3	Q9NSD7	8	MITF	O75030	3
RRP12	Q5JTH9	8	MKRN1	Q9UHC7	3
RPS6KB2	Q9UBS0	8	MKRN2	Q9H000	3
RPLP1	P05386	8	MLF1	P58340	3
RPL18A	Q02543	8	MLL5	Q8IZD2	3
RNF139	Q8WU17	8	MME	P08473	3
RIPK1	Q13546	8	MOB4	Q9Y3A3	3
RCOR3	Q9P2K3	8	MOBKL3	Q9Y3A3	3
RCC1	P18754	8	MORC1	Q86VD1	3
RANBP3	Q9H6Z4	8	MORC4	Q8TE76	3
PYY	P10082	8	MORG1	Q9BRX9	3
PXN	P49023	8	MPHOSPH8	Q99549	3
PTPRC	P08575	8	MPHOSPH9	Q99550	3
PTPN1	P18031	8	MPRIP	Q6WCQ1	3
PTGFR	P43088	8	MRPS22	P82650	3
PSEN2	P49810	8	MRPS31	Q92665	3
PROK2	Q9HC23	8	MSR1	P21757	3
PRKCQ	Q04759	8	MSTO1	Q9BUK6	3
PRKCI	P41743	8	MTA1	Q13330	3
PRKCG	P05129	8	MTA3	Q9BTC8	3
PPP4C	P60510	8	MTDH	Q86UE4	3
PPM1G	O15355	8	MTFR1	Q15390	3
PNMA1	Q8ND90	8	MTX2	O75431	3
PENK	PENK	8	MUM1L1	Q5H9M0	3
PDYN	PDYN	8	MVP	Q14764	3
PCBD1	P61457	8	MYBBP1A	Q9BQG0	3
PBK	Q96KB5	8	MYB	P10242	3
PARK2	O60260	8	MYD88	Q99836	3
P2RY14	Q15391	8	MYH10	P35580	3
P2RY13	Q9BPV8	8	MYO18A	Q92614	3
P2RY12	Q9H244	8	MYOCD	Q8IZQ8	3
NUTF2	P61970	8	NAA11	Q9BSU3	3
NTSR2	O95665	8	NAPA	P54920	3
NRAS	P01111	8	NAPG	Q99747	3
NPY5R	Q15761	8	NASP	P49321	3
NPFFR2	Q9Y5X5	8	NCAPD2	Q15021	3
NPFFR1	Q9GZQ6	8	NCAPH	Q15003	3
NPBWR1	P48145	8	NCOA1	Q15788	3
NPB	Q8NG41	8	NCOA2	Q15596	3
NONO	Q15233	8	NCOA6	Q14686	3
NFRKB	Q6P4R8	8	NCOR2	Q9Y618	3
NDRG1	Q92597	8	NDNL2	Q96MG7	3
MYBBP1A	Q9BQG0	8	NDN	Q99608	3
LUC7L2	Q9Y383	8	NDRG1	Q92597	3
LTB4R2	Q9NPC1	8	NDRG3	Q9UGV2	3
LLGL1	Q15334	8	NECAP1	Q8NC96	3
LCK	P06239	8	NEDD8	Q15843	3
LAMB2	P55268	8	NEFH	P12036	3
KLC1	Q07866	8	NEFL	P07196	3
ITGB5	P18084	8	NEK10	Q6ZWH5	3
ITGB1	P05556	8	NFATC2	Q13469	3
ITGA3	P26006	8	NFKB1	P19838	3
IRF1	P10914	8	NFKB2	Q00653	3
HYRC	HYRC	8	NFKBIA	P25963	3
HTR5A	P47898	8	NFKBIB	Q15653	3
HSD17B4	P51659	8	NFKBIE	O00221	3
HRH3	Q9Y5N1	8	NFYB	P25208	3
HRAS	P01112	8	NFYC	Q13952	3

HIP1	O00291	8	NGFR	P08138	3
HEBP1	Q9NRV9	8	NHLRC1	Q6VVB1	3
HCAR2	Q8TDS4	8	NME5	P56597	3
GSTM3	P21266	8	NOD1	Q9Y239	3
GRK5	P34947	8	NONO	Q15233	3
GPRC6A	Q5T6X5	8	NOV	P48745	3
GPR55	Q9Y2T6	8	NPTX1	Q15818	3
GPR18	Q14330	8	NPTX2	P47972	3
GPER	Q99527	8	NPTXR	O95502	3
GNAI3	P08754	8	NQO1	P15559	3
GNAI1	P63096	8	NQO2	P16083	3
FRS3	O43559	8	NR3C1	P04150	3
FPR3	P25089	8	NR4A2	P43354	3
FANCG	O15287	8	NRIP1	P48552	3
F2R	P25116	8	NSDHL	Q15738	3
F2RL3	Q96RI0	8	NSF	P46459	3
ERCC8	Q13216	8	NSMAF	Q92636	3
EIF6	P56537	8	NSUN3	Q9H649	3
EIF5	P55010	8	NTF4	P34130	3
EIF4G1	Q04637	8	NUDT11	Q96G61	3
EIF2C4	Q9HCK5	8	NUDT1	P36639	3
DNAJB1	P25685	8	NUDT2	P50583	3
DNAJA3	Q96EY1	8	NXT2	Q9NPJ8	3
DDX3X	O00571	8	OGG1	O15527	3
DDX21	Q9NR30	8	ORMDL2	Q53FV1	3
DDB2	Q92466	8	OSMR	Q99650	3
CXCR7	P25106	8	OXSR1	O95747	3
CXCL16	Q9H2A7	8	PACSIN2	Q9UNF0	3
CUL5	Q93034	8	PACSIN3	Q9UKS6	3
CSNK2A2	P19784	8	PAFAH1B3	Q15102	3
COL1A2	P08123	8	PAPOLB	Q9NRJ5	3
CLTA	P09496	8	PARD3	Q8TEW0	3
CKAP4	Q07065	8	PARK7	Q99497	3
CITED1	Q99966	8	PARP1	P09874	3
CD82	P27701	8	PAX8	Q06710	3
CD81	P60033	8	PBK	Q96KB5	3
CD46	P15529	8	PCBD1	P61457	3
CCT7	Q99832	8	PCYT2	Q99447	3
CCR7	P32248	8	PDHX	O00330	3
CCR2	P41597	8	PDXK	O00764	3
CASP9	P55211	8	PEX5	P50542	3
CASP4	P49662	8	PGK1	P00558	3
C5	P01031	8	PGK2	P07205	3
C3	P01024	8	PGM1	P36871	3
C3AR1	Q16581	8	PGM2	Q96G03	3
BTK	Q06187	8	PGM3	O95394	3
BIRC3	Q13489	8	PHF7	Q9BWX1	3
BIN1	O00499	8	PIAS1	O75925	3
APLNR	P35414	8	PIAS2	O75928	3
APLN	Q9ULZ1	8	PIGV	Q9NUD9	3
AP2M1	Q96CW1	8	PIH1D2	Q8WWB5	3
AP2B1	P63010	8	PIM2	Q9P1W9	3
AP2A2	O94973	8	PIN1	Q13526	3
ANXA7	P20073	8	PIWIL1	Q96J94	3
ALB	P02768	8	PLSCR1	O15162	3
AHSA1	O95433	8	PML	P29590	3
ADCY9	O60503	8	PNMA1	Q8ND90	3
ADCY8	P40145	8	POP7	O75817	3
ADCY7	P51828	8	POU2F1	P14859	3

ADCY6	O43306	8	POU2F2	P09086	3
ADCY5	O95622	8	POU5F1	Q01860	3
ADCY4	Q8NFM4	8	PPA1	Q15181	3
ADCY3	O60266	8	PPARG	P37231	3
ADCY2	Q08462	8	PPIB	P23284	3
ADCY1	Q08828	8	PPM1B	O75688	3
ACTL6A	O96019	8	PPM1G	O15355	3
A2M	P01023	8	PPP1R13B	Q96KQ4	3
ZNRF4	Q8WWF5	7	PPP1R15A	O75807	3
WTAP	Q15007	7	PPP2R1B	P30154	3
VDR	P11473	7	PPP2R2A	P63151	3
UBQLN4	Q9NRR5	7	PPP2R2B	Q00005	3
UBE3A	Q05086	7	PPP2R2D	Q66LE6	3
UBE2S	Q16763	7	PPP2R4	Q15257	3
UBE2N	P61088	7	PPP4C	P60510	3
UBE2D3	P61077	7	PRDM4	Q9UKN5	3
UBA1	P22314	7	PRDX1	Q06830	3
TXN	P10599	7	PRKAA1	Q13131	3
TSSK6	Q9BXA6	7	PRKAA2	P54646	3
TSNAX	Q99598	7	PRKACB	P22694	3
TSC1	Q92574	7	PRKCB	P05771	3
TRIM24	O15164	7	PRKCG	P05129	3
TP53BP2	Q13625	7	PRKCI	P41743	3
TLR4	O00206	7	PRNP	P04156	3
TICAM1	Q8IUC6	7	PROS1	P07225	3
TGM2	P21980	7	PRPF40A	O75400	3
TCF7L2	Q9NQB0	7	PRR5	P85299	3
TCF4	P15884	7	PRSS21	Q9Y6M0	3
TBC1D15	Q8TC07	7	PSAT1	Q9Y617	3
STIP1	P31948	7	PSMG1	O95456	3
SPI1	P17947	7	PTDSS2	Q9BVG9	3
SPARC	P09486	7	PTGES3	Q15185	3
SMARCD3	Q6STE5	7	PTPN13	Q12923	3
SMAD1	Q15797	7	PTPN9	P43378	3
SLC1A5	Q15758	7	PXDNL	PXDNL	3
SIK3	Q9Y2K2	7	PYGL	P06737	3
SENP1	Q9P0U3	7	QRICH1	Q2TAL8	3
S1PR3	Q99500	7	RAB11FIP5	Q9BXF6	3
S1PR2	O95136	7	RAB24	Q969Q5	3
RRM2	P31350	7	RAB28	RAB28	3
RRBP1	Q9P2E9	7	RAB2A	P61019	3
RPS6KA5	O75582	7	RAB3GAP2	Q9H2M9	3
RPS20	P60866	7	RAD21	O60216	3
RNF216	Q9NWF9	7	RAD52	P43351	3
RNF185	Q96GF1	7	RALGPS2	Q86X27	3
RECQL4	O94761	7	RANBP9	Q96S59	3
RBM39	Q14498	7	RAPGEF2	Q9Y4G8	3
RAB11FIP5	Q9BXF6	7	RARA	P10276	3
PTGER3	P43115	7	RBBP6	Q7Z6E9	3
PRPF19	Q9UMS4	7	RCN2	Q14257	3
PRKAA1	Q13131	7	RCOR3	Q9P2K3	3
PLSCR1	O15162	7	RECQL	P46063	3
PLCB4	Q15147	7	RELA	Q04206	3
PAFAH1B3	Q15102	7	RFWD3	Q6PCD5	3
PABPC1	P11940	7	RIBC2	Q9H4K1	3
OXER1	Q8TDS5	7	RICTOR	Q6R327	3
NPY1R	P25929	7	RIF1	Q5UIP0	3
NEK6	Q9HC98	7	RIOK1	Q9BRS2	3
NAE1	Q13564	7	RIPK1	Q13546	3

MYH10	P35580	7	RIPK2	O43353	3
MYD88	Q99836	7	RIPK3	Q9Y572	3
MRPS31	Q92665	7	RNF10	Q8N5U6	3
MRPS22	P82650	7	RNF138	Q8WVD3	3
MMP2	P08253	7	RNF139	Q8WU17	3
MEN1	O00255	7	RNF146	Q9NTX7	3
MDM4	O15151	7	RNF19B	Q6ZMZ0	3
MBP	P02686	7	RNF216	Q9NWF9	3
MAX	P61244	7	RNF2	Q99496	3
MAVS	Q7Z434	7	RNF5	Q99942	3
MAPKAPK2	P49137	7	RNFT1	Q5M7Z0	3
MAPK8IP2	Q13387	7	RNH1	P13489	3
MAPK11	Q15759	7	RPL10L	Q96L21	3
MAPK10	P53779	7	RPS6KA5	O75582	3
MAGEH1	Q9H213	7	RRBP1	Q9P2E9	3
LSM4	Q9Y4Z0	7	RRP12	Q5JTH9	3
LSM3	P62310	7	RSPH3	Q86UC2	3
LAMC3	Q9Y6N6	7	RUNX1	Q01196	3
LAMC1	P11047	7	RXRA	P19793	3
LAMA4	Q16363	7	S100A6	P06703	3
L1CAM	P32004	7	S100PBP	Q96BU1	3
KRAS	P01116	7	SAE1	Q9UBE0	3
KIT	P10721	7	SAFB	Q15424	3
KIF9	Q9HAQ2	7	SALL2	Q9Y467	3
KARS	Q15046	7	SAMD9	Q5K651	3
JAK3	P52333	7	SAP18	O00422	3
ITPR1	Q14643	7	SATB2	Q9UPW6	3
ITGB4	P16144	7	SCAF4	O95104	3
ITGA6	P23229	7	SCAPER	Q9BY12	3
IRAK1	P51617	7	SCAP	Q12770	3
IL3RA	P26951	7	SCCPDH	Q8NBX0	3
HSPA1L	P34931	7	SCO1	O75880	3
HSP90B1	P14625	7	SDHA	P31040	3
HLA-E	P13747	7	SEC22B	O75396	3
HLA-B	P01889	7	SENP1	Q9P0U3	3
HELLS	Q9NRZ9	7	SENP3	Q9H4L4	3
GPR44	GPR44	7	SEPT2	Q15019	3
GNAZ	P19086	7	SEPT7	Q16181	3
GNAS	P63092	7	SERPINB9	P50453	3
GALR1	P47211	7	SERTAD1	Q9UHV2	3
GADD45G	O95257	7	SESN3	P58005	3
FOXO1	Q12778	7	SETD4	Q9NVD3	3
FES	P07332	7	SGK1	O00141	3
EXOSC8	Q96B26	7	SH3BP4	Q9P0V3	3
EIF4G2	P78344	7	SH3GLB1	Q9Y371	3
EIF4B	P23588	7	SIAH1	Q8IUQ4	3
EIF2S1	P05198	7	SIK3	Q9Y2K2	3
EIF2B3	Q9NR50	7	SIKE1	Q9BRV8	3
EID1	Q9Y6B2	7	SIL1	Q9H173	3
DNM2	P50570	7	SIRT1	Q96EB6	3
DNAJB11	Q9UBS4	7	SIRT3	Q9NTG7	3
DDX42	Q86XP3	7	SKIL	P12757	3
CYCS	P99999	7	SKI	P12755	3
CUL2	Q13617	7	SLC18A2	Q05940	3
CTSS	P25774	7	SLC18A3	Q16572	3
CSK	P41240	7	SLC1A5	Q15758	3
COL1A1	P02452	7	SLC25A31	Q9H0C2	3
CNOT7	Q9UIV1	7	SLC2A14	Q8TDB8	3
CNOT10	Q9H9A5	7	SLC2A1	P11166	3

CDK16	Q00536	7	SLC2A3	P11169	3
CDC25B	P30305	7	SLC2A4	P14672	3
CCL28	Q9NRJ3	7	SLC32A1	Q9H598	3
CASP10	Q92851	7	SLC41A3	Q96GZ6	3
CANX	P27824	7	SLC45A4	SLC45A4	3
CALR	P27797	7	SLX4	Q8IY92	3
C5orf25	Q8NDZ2	7	SMAD1	Q15797	3
BID	P55957	7	SMAD2	Q15796	3
BECN1	Q14457	7	SMAD3	P84022	3
BCR	P11274	7	SMAD4	Q13485	3
BCL3	P20749	7	SMAD5	Q99717	3
BAG6	P46379	7	SMARCC1	Q92922	3
BAG1	Q99933	7	SMARCD1	Q96GM5	3
ATXN3	P54252	7	SMARCD3	Q6STE5	3
ATAD2	Q6PL18	7	SMC4	Q9NTJ3	3
ARID4A	P29374	7	SMURF1	Q9HCE7	3
APAF1	O14727	7	SMURF2	Q9HAU4	3
AP2A1	O95782	7	SNAP23	O00161	3
AKR1B1	P15121	7	SNAP29	O95721	3
ADRA2A	P08913	7	SNAP47	Q5SQN1	3
ADORA1	P30542	7	SNAP91	O60641	3
ZNF746	Q6NUN9	6	SNCA	P37840	3
YWHAH	Q04917	6	SNRK	Q9NRH2	3
YEATS4	O95619	6	SNW1	Q13573	3
XRCC4	Q13426	6	SOAT1	P35610	3
XBP1	P17861	6	SOD1	P00441	3
WWTR1	Q9GZV5	6	SORCS3	Q9UPU3	3
WNK1	Q9H4A3	6	SORT1	Q99523	3
VRK3	Q8IV63	6	SOX10	P56693	3
VPS37A	Q8NEZ2	6	SOX9	P48436	3
VPS13A	Q96RL7	6	SP1	P08047	3
USP19	O94966	6	SPECC1L	Q69YQ0	3
UNG	P13051	6	SPG20	Q8N0X7	3
UFD1L	Q92890	6	SPI1	P17947	3
UBE2W	Q96B02	6	SQSTM1	Q13501	3
UBE2H	P62256	6	SREBF2	Q12772	3
TUBB2A	Q13885	6	SSFA2	P28290	3
TTR	P02766	6	SSSCA1	O60232	3
TSGA10	Q9BZW7	6	SSX2IP	Q9Y2D8	3
TRIP13	Q15645	6	ST13	P50502	3
TRIM37	O94972	6	STAT3	P40763	3
TRAF3IP1	Q8TDR0	6	STIP1	P31948	3
TPP2	P29144	6	STK11IP	Q8N1F8	3
TPD52L1	Q16890	6	STK11	Q15831	3
TNC	P24821	6	STK24	Q9Y6E0	3
TIAM1	Q13009	6	STK3	Q13188	3
THAP11	Q96EK4	6	STK4	Q13043	3
TFAP2C	Q92754	6	STMN1	P16949	3
TCERG1	O14776	6	STOM	P27105	3
TADA2A	O75478	6	STRN4	Q9NRL3	3
TAC1	TAC1	6	STUB1	Q9UNE7	3
SYCP1	Q15431	6	STX10	O60499	3
STAT6	P42226	6	STX12	Q86Y82	3
SMURF2	Q9HAU4	6	STX1A	Q16623	3
SMU1	Q2TAY7	6	STX1B1	STX1B1	3
SLC9A3R2	Q15599	6	STX1B	P61266	3
SKI	P12755	6	STX2	P32856	3
SKIL	P12757	6	STX3	Q13277	3
SENP3	Q9H4L4	6	STX4	Q12846	3

SAP18	O00422	6	STX6	O43752	3
RYK	P34925	6	STX7	O15400	3
RPN2	P04844	6	STX8	Q9UNK0	3
RNF2	Q99496	6	STXBP1	P61764	3
RNF138	Q8WVD3	6	SUGT1	Q9Y2Z0	3
RIBC2	Q9H4K1	6	SUMO1	P63165	3
RANBP9	Q96S59	6	SUMO3	P55854	3
RAD52	P43351	6	SV2A	Q7L0J3	3
RABEP1	Q15276	6	SVIL	O95425	3
QRFP	P83859	6	SYCP1	Q15431	3
QKI	Q96PU8	6	SYTL4	Q96C24	3
PYGL	P06737	6	TAB2	Q9NYJ8	3
PTPRB	P23467	6	TANK	Q92844	3
PTPN2	P17706	6	TBC1D15	Q8TC07	3
PTPN12	Q05209	6	TBC1D8B	TBC1D8B	3
PTH2R	P49190	6	TBK1	Q9UHD2	3
PRKCE	Q02156	6	TBL2	Q9Y4P3	3
PRKAR1A	P10644	6	TCF19	Q9Y242	3
PRDX1	Q06830	6	TCF4	P15884	3
PPM1B	O75688	6	TCF7L2	Q9NQB0	3
PPM1A	P35813	6	TCP1	P17987	3
POLR3H	Q9Y535	6	TDGF1	P13385	3
POLR3B	Q9NW08	6	TDG	Q13569	3
PDPK1	O15530	6	TDRKH	Q9Y2W6	3
PDGFRA	P16234	6	TEX10	Q9NXF1	3
PDGFB	P01127	6	TFAP2A	P05549	3
PDGFA	P04085	6	TFAP2C	Q92754	3
PARD3	Q8TEW0	6	TGFB1I1	O43294	3
PAICS	P22234	6	TGFBR1	P36897	3
OXT	P01178	6	THAP11	Q96EK4	3
NUMA1	Q14980	6	THUMPD3	Q9BV44	3
NTRK1	P04629	6	THYN1	Q9P016	3
NQO1	P15559	6	TICAM1	Q8IUC6	3
NPHP1	O15259	6	TK1	P04183	3
NPFF	O15130	6	TLR4	O00206	3
NFATC3	Q12968	6	TMEM132A	Q24JP5	3
MPHOSPH9	Q99550	6	TMEM192	Q8IY95	3
MPG	P29372	6	TMEM57	Q8N5G2	3
MOBKL3	MOBKL3	6	TMEM62	Q0P6H9	3
MKRN1	Q9UHC7	6	TMEM67	Q5HYA8	3
MEF2A	Q02078	6	TNF	P01375	3
MED31	Q9Y3C7	6	TNFRSF1A	P19438	3
MED12	Q93074	6	TNFRSF1B	P20333	3
MDH1	P40925	6	TNP1	P09430	3
MAP3K8	P41279	6	TOMM34	Q15785	3
MAGEA6	P43360	6	TOMM70A	O94826	3
MAF	O75444	6	TP53BP2	Q13625	3
MAD2L2	Q9UI95	6	TP53	P04637	3
KISS1	Q15726	6	TP63	Q9H3D4	3
KDM1A	O60341	6	TP73	O15350	3
JUND	P17535	6	TPPP	O94811	3
JUNB	P17275	6	TPTE	P56180	3
JAG2	Q9Y219	6	TRADD	Q15628	3
ITGAX	P20702	6	TRAF1	Q13077	3
INTS6	Q9UL03	6	TRAF2	Q12933	3
ILK	Q13418	6	TRAF3	Q13114	3
ILF3	Q12906	6	TRAF5	O00463	3
ID2	Q02363	6	TRAF6	Q9Y4K3	3
HMGN1	P05114	6	TRIAP1	O43715	3

HLA-C	P10321	6	TRIM24	O15164	3
HLA-A	P30443	6	TRIM28	Q13263	3
HIST1H1A	Q02539	6	TRIM37	O94972	3
HEATR1	Q9H583	6	TRIM38	O00635	3
HCRT	O43612	6	TRIOBP	Q9H2D6	3
HBEGF	Q99075	6	TRIP4	Q15650	3
GTF3C4	Q9UKN8	6	TSC22D1	Q15714	3
GSN	P06396	6	TSC22D3	Q99576	3
GOPC	Q9HD26	6	TSEN34	Q9BSV6	3
GFAP	P14136	6	TSGA10	Q9BZW7	3
GET4	Q7L5D6	6	TSNAX	Q99598	3
GCDH	Q92947	6	TSPY1	Q01534	3
FTH1	P02794	6	TSPY3	P0CV98	3
FOSL2	P15408	6	TSSK4	Q6SA08	3
FKBP6	O75344	6	TSSK6	Q9BXA6	3
FGFR1	P11362	6	TTC26	A0AVF1	3
FEN1	P39748	6	TTC33	Q6PID6	3
FAM48A	Q8NEM7	6	TTC7B	Q86TV6	3
ERBB4	Q15303	6	TTK	P33981	3
EPHA8	P29322	6	TTLL4	Q14679	3
EPAS1	Q99814	6	TTLL5	Q6EMB2	3
EIF3J	O75822	6	TTR	P02766	3
EGF	P01133	6	TUBB2A	Q13885	3
DYX1C1	Q8WXU2	6	TUBD1	Q9UJT1	3
DYNLL2	Q96FJ2	6	TXNIP	Q9H3M7	3
DSG1	Q02413	6	TXN	P10599	3
DRG1	Q9Y295	6	TXNRD1	Q16881	3
DNAJB4	Q9UDY4	6	UBA1	P22314	3
DLG4	P78352	6	UBAP2	Q5T6F2	3
DLC1	Q96QB1	6	UBC	P0CG48	3
DDX19B	Q9UMR2	6	UBE2D2	P62837	3
CUX1	P39880	6	UBE2D3	P61077	3
CUL3	Q13618	6	UBE2E2	Q96LR5	3
CRKL	P46109	6	UBE2E3	Q969T4	3
COL6A1	P12109	6	UBE2H	P62256	3
CNTN2	Q02246	6	UBE2I	P63279	3
CDK5	Q00535	6	UBE2K	P61086	3
CD99	P14209	6	UBE2N	P61088	3
CALB2	P22676	6	UBE2Q1	Q7Z7E8	3
CACNA1A	O00555	6	UBE2U	Q5VVX9	3
C7orf64	C7orf64	6	UBE2V1	Q13404	3
BTBD2	Q9BX70	6	UBE2W	Q96B02	3
BRE	Q9NXR7	6	ULK2	Q8IYT8	3
BRCC3	P46736	6	UNC119	Q13432	3
BNIP3L	O60238	6	UNC13B	O14795	3
BIRC2	Q13490	6	USO1	O60763	3
BCL2L1	Q07817	6	USP19	O94966	3
BAX	Q07812	6	USP2	O75604	3
BAP1	Q92560	6	USP38	Q8NB14	3
B2M	P61769	6	USP45	Q70EL2	3
ATP5A1	P25705	6	VAMP2	P63027	3
ATP2B4	P23634	6	VAMP4	O75379	3
ARHGEF25	Q86VW2	6	VAMP7	P51809	3
ARFGEF1	Q9Y6D6	6	VDR	P11473	3
APPL1	Q9UKG1	6	VIM	P08670	3
**APLP2**	**Q06481**	**6**	VTI1A	Q96AJ9	3
APEX1	P27695	6	VTI1B	Q9UEU0	3
APBB1	O00213	6	WDR16	Q8N1V2	3
ANXA4	P09525	6	WDR54	Q9H977	3

ANKS1A	Q92625	6	WDR62	O43379	3
AMOT	Q4VCS5	6	WDR6	Q9NNW5	3
ADRB1	P08588	6	WIPI2	Q9Y4P8	3
ADA	P00813	6	WNK1	Q9H4A3	3
ADAM17	P78536	6	WTAP	Q15007	3
AASDHPPT	Q9NRN7	6	XIAP	P98170	3
ZC3H18	Q86VM9	5	XPNPEP1	Q9NQW7	3
XPNPEP1	Q9NQW7	5	XPO5	Q9HAV4	3
WEE1	P30291	5	YBX2	Q9Y2T7	3
WDTC1	Q8N5D0	5	YWHAB	P31946	3
WBSCR22	O43709	5	YWHAG	P61981	3
VTN	P04004	5	YWHAQ	P27348	3
VIPR1	P32241	5	YWHAZ	P63104	3
USP45	Q70EL2	5	YY1AP1	Q9H869	3
UBTF	P17480	5	ZBTB39	ZBTB39	3
UBR2	Q8IWV8	5	ZBTB5	O15062	3
UBQLN1	Q9UMX0	5	ZC3H18	Q86VM9	3
UBE2E2	Q96LR5	5	ZDHHC20	Q5W0Z9	3
TXNIP	Q9H3M7	5	ZEB1	P37275	3
TSC2	P49815	5	ZFHX4	Q86UP3	3
TRPC1	P48995	5	ZNF200	P98182	3
TRIP6	Q15654	5	ZNF274	Q96GC6	3
TOMM34	Q15785	5	ZNF318	Q5VUA4	3
TLE1	Q04724	5	ZNF711	Q9Y462	3
TELO2	Q9Y4R8	5	ZNRF4	Q8WWF5	3
TDG	Q13569	5	ZP3	P21754	3
TAF15	Q92804	5	ZZEF1	O43149	3
SYNCRIP	O60506	5	ADAM9	Q13443	4
STOM	P27105	5	AMPH	P49418	4
SRGAP3	O43295	5	ANAPC10	Q9UM13	4
SPTBN1	Q01082	5	ANAPC11	Q9NYG5	4
SPG20	Q8N0X7	5	ANAPC1	Q9H1A4	4
SPECC1L	Q69YQ0	5	ANAPC2	Q9UJX6	4
SNAP91	O60641	5	ANAPC4	Q9UJX5	4
SMG1	Q96Q15	5	ANAPC5	Q9UJX4	4
SMAD5	Q99717	5	ANAPC7	Q9UJX3	4
SLC2A4	P14672	5	AP1B1	Q10567	4
SH3BP4	Q9P0V3	5	APC	P25054	4
SERPINB9	P50453	5	APEH	P13798	4
SARNP	P82979	5	APPBP2	Q92624	4
S100A7	P31151	5	ARL6IP5	O75915	4
RPL10L	Q96L21	5	ARL6	Q9H0F7	4
RNF32	Q9H0A6	5	ASB9	Q96DX5	4
RBBP6	Q7Z6E9	5	AXIN1	O15169	4
RASA1	P20936	5	BTRC	Q9Y297	4
RAP1A	P62834	5	BUB1B	O60566	4
RALGPS2	Q86X27	5	C13orf15	C13orf15	4
RAG2	P55895	5	CABYR	O75952	4
PTPRO	Q16827	5	CALD1	Q05682	4
PTPRJ	Q12913	5	CBLL1	Q75N03	4
PTHLH	P12272	5	CCNB1	P14635	4
PTH1R	Q03431	5	CCNB2	O95067	4
PRRC2A	P48634	5	CCND1	P24385	4
PRDX2	P32119	5	CCNE2	O96020	4
PPIB	P23284	5	CCNF	P41002	4
PPARG	P37231	5	CCNH	P51946	4
PLG	P00747	5	CD4	P01730	4
PLD2	O14939	5	CD8A	P01732	4
PLAA	Q9Y263	5	CDC16	Q13042	4

PKN1	Q16512	5	CDC23	Q9UJX2	4
PIM2	Q9P1W9	5	CDC25A	P30304	4
PIK3CB	P42338	5	CDC25B	P30305	4
PEX5	P50542	5	CDC25C	P30307	4
PDGFD	Q9GZP0	5	CDC26	Q8NHZ8	4
PCID2	Q5JVF3	5	CDC27	P30260	4
NUDT1	P36639	5	CDCA2	Q69YH5	4
NOD1	Q9Y239	5	CDH1	P12830	4
NGFRAP1	Q00994	5	CDK1	P06493	4
NFYB	P25208	5	CHMP5	Q9NZZ3	4
NEFL	P07196	5	CKS1B	P61024	4
NCSTN	Q92542	5	CKS2	P33552	4
NCAPD2	Q15021	5	CSNK1A1	P48729	4
NCAM1	P13591	5	CTNNB1	P35222	4
MYST2	MYST2	5	CUL1	Q13616	4
MYO18A	Q92614	5	CUL3	Q13618	4
MTA3	Q9BTC8	5	CUL5	Q93034	4
MPHOSPH8	Q99549	5	CUX1	P39880	4
MMP9	P14780	5	DEDD	O75618	4
MMP14	P50281	5	DLGAP5	Q15398	4
MKI67	P46013	5	DNHD1	Q96M86	4
MITF	O75030	5	DUT	P33316	4
MET	P08581	5	ERG	P11308	4
MAST1	Q9Y2H9	5	FAM92A1	A1XBS5	4
MAPRE2	Q15555	5	FBXO5	Q9UKT4	4
MAP2K1	Q02750	5	FER	P16591	4
MAP1S	Q66K74	5	FRAT2	O75474	4
LZTS1	Q9Y250	5	FZR1	Q9UM11	4
LYL1	P12980	5	GADD45B	O75293	4
LTBP1	Q14766	5	GMNN	O75496	4
LRR1	Q96L50	5	GORASP1	Q9BQQ3	4
LHCGR	P22888	5	GSK3B	P49841	4
KRT8	P05787	5	HMGA2	P52926	4
KRT7	P08729	5	IL16	Q14005	4
KRT15	P19012	5	IST1	P53990	4
KLK5	Q9Y337	5	KIF20B	Q96Q89	4
KLHL8	Q9P2G9	5	LATS1	O95835	4
KIAA1683	Q9H0B3	5	MAD2L1	Q13257	4
KCTD13	Q8WZ19	5	MAD2L2	Q9UI95	4
KAT7	O95251	5	MAP4	P27816	4
ITPR3	Q14573	5	MEF2C	Q06413	4
ITPR2	Q14571	5	MNAT1	P51948	4
IST1	P53990	5	MNDA	P41218	4
INPP5D	Q92835	5	MYO6	Q9UM54	4
IFT57	Q9NWB7	5	MYT1	Q01538	4
IFIT5	Q13325	5	NEDD9	Q14511	4
HSPE1	P61604	5	NES	P48681	4
HSPBP1	Q9NZL4	5	NFYA	P23511	4
HLA-G	P17693	5	NSFL1C	Q9UNZ2	4
HLA-F	P30511	5	ORC1	Q13415	4
HLA-C	Q9TNN7	5	PAK2	Q13177	4
HLA-C	Q29963	5	PAK6	Q9NQU5	4
HLA-C	P04222	5	PITPNM1	O00562	4
HLA-B	Q95365	5	PKMYT1	Q99640	4
HLA-B	Q31612	5	PLK1	P53350	4
HLA-B	Q31610	5	PPIL5	PPIL5	4
HLA-B	Q29940	5	PPP1R12B	O60237	4
HLA-B	Q29836	5	PRKG1	Q13976	4
HLA-B	Q29718	5	PSMA1	P25786	4

HLA-B	Q04826	5	PSMA2	P25787	4
HLA-B	P30685	5	PSMA3	P25788	4
HLA-B	P30498	5	PSMA4	P25789	4
HLA-B	P30495	5	PSMA5	P28066	4
HLA-B	P30493	5	PSMA6	P60900	4
HLA-B	P30492	5	PSMA7	O14818	4
HLA-B	P30491	5	PSMA8	Q8TAA3	4
HLA-B	P30490	5	PSMB10	P40306	4
HLA-B	P30488	5	PSMB1	P20618	4
HLA-B	P30487	5	PSMB2	P49721	4
HLA-B	P30485	5	PSMB3	P49720	4
HLA-B	P30484	5	PSMB4	P28070	4
HLA-B	P30483	5	PSMB5	P28074	4
HLA-B	P30481	5	PSMB6	P28072	4
HLA-B	P30479	5	PSMB7	Q99436	4
HLA-B	P30475	5	PSMB8	P28062	4
HLA-B	P30466	5	PSMC1	P62191	4
HLA-B	P30464	5	PSMC2	P35998	4
HLA-B	P30462	5	PSMC3	P17980	4
HLA-B	P30461	5	PSMC4	P43686	4
HLA-B	P30460	5	PSMC5	P62195	4
HLA-B	P18465	5	PSMC6	P62333	4
HLA-B	P18464	5	PSMD10	O75832	4
HLA-B	P18463	5	PSMD11	O00231	4
HLA-B	P10319	5	PSMD12	O00232	4
HLA-B	P03989	5	PSMD13	Q9UNM6	4
HLA-A	P04439	5	PSMD14	O00487	4
HLA-A	P01892	5	PSMD1	Q99460	4
HIST1H2BK	O60814	5	PSMD2	Q13200	4
HIST1H2AG	P0C0S8	5	PSMD3	O43242	4
HDAC7	Q8WUI4	5	PSMD4	P55036	4
GRK4	P32298	5	PSMD5	Q16401	4
GKAP1	Q5VSY0	5	PSMD6	Q15008	4
GAPVD1	Q14C86	5	PSMD7	P51665	4
GANAB	Q14697	5	PSMD8	P48556	4
GAB1	Q13480	5	PSMD9	O00233	4
G6PD	P11413	5	PSME1	Q06323	4
FSHR	P23945	5	PSME2	Q9UL46	4
FRAT2	O75474	5	PSME3	P61289	4
FGFR4	P22455	5	PSME4	Q14997	4
FBXW11	Q9UKB1	5	PSMF1	Q92530	4
FBXO25	Q8TCJ0	5	PTCH1	Q13635	4
FANCM	Q8IYD8	5	PTPN2	P17706	4
EXOC7	Q9UPT5	5	PTTG1	O95997	4
ERBB3	P21860	5	RAB4A	P20338	4
EPN1	Q9Y6I3	5	RAB5B	P61020	4
EIF4G3	O43432	5	RABEP1	Q15276	4
EIF4EBP1	Q13541	5	RAGE	RAGE	4
EIF3E	P60228	5	RBX1	P62877	4
DIAPH3	Q9NSV4	5	RFWD2	Q8NHY2	4
DGCR8	Q8WYQ5	5	RPS27A	P62979	4
DERL2	Q9GZP9	5	RPS6KB1	P23443	4
DERL1	Q9BUN8	5	SEC61B	P60468	4
DDX24	Q9GZR7	5	SKP1	P63208	4
DCP1B	Q8IZD4	5	SKP2	Q13309	4
CSTA	P01040	5	SLX1A	Q9BQ83	4
CSMD2	Q7Z408	5	SLX1B	SLX1B	4
CIITA	P33076	5	SSBP1	Q04837	4
CETN3	O15182	5	STMN2	Q93045	4

CDKN3	Q16667	5	STX5	Q13190	4
CDC73	Q6P1J9	5	TAP1	Q03518	4
CDC25C	P30307	5	TAP2	Q03519	4
CD9	P21926	5	TGFBR2	P37173	4
CD2AP	Q9Y5K6	5	TPP2	P29144	4
CBLL1	Q75N03	5	TSC1	Q92574	4
CAPZA1	P52907	5	TSC2	P49815	4
CALM3	P62158	5	TSPYL2	Q9H2G4	4
BNIP2	Q12982	5	UBA52	P62987	4
BING2	BING2	5	UBB	P0CG47	4
BAG3	O95817	5	UBE2C	O00762	4
BAD	Q92934	5	UBE2D1	P51668	4
BABAM1	Q9NWV8	5	UBE2E1	P51965	4
ATP2B1	P20020	5	USP14	P54578	4
ATG9A	Q7Z3C6	5	USP16	Q9Y5T5	4
ATF4	P18848	5	USP9X	Q93008	4
ARHGDIB	P52566	5	VEZT	Q9HBM0	4
APLP1	P51693	5	VPS37A	Q8NEZ2	4
AP2S1	P53680	5	WEE1	P30291	4
AP1B1	Q10567	5	AASS	Q9UDR5	5
ANXA2	P07355	5	AATF	Q9NY61	5
ADM	P35318	5	ACAT2	Q9BWD1	5
ADD1	P35611	5	ACLY	P53396	5
ADCYAP1R1	P41586	5	ADNP2	Q6IQ32	5
ADAM9	Q13443	5	AGA	P20933	5
ADAM10	O14672	5	AIFM2	Q9BRQ8	5
ACE2	Q9BYF1	5	AKAP8L	Q9ULX6	5
ZNF318	Q5VUA4	4	ALB	P02768	5
ZHX2	Q9Y6X8	4	ANXA4	P09525	5
WAPAL	Q7Z5K2	4	ARID4A	P29374	5
VPRBP	Q9Y4B6	4	ATAD2	Q6PL18	5
VIPR2	P41587	4	ATF1	P18846	5
VIP	P01282	4	ATG5	Q9H1Y0	5
VAMP2	P63027	4	ATM	Q13315	5
USP9X	Q93008	4	ATR	Q13535	5
USP50	Q70EL3	4	BAG5	Q9UL15	5
USP44	Q9H0E7	4	BARD1	Q99728	5
USP21	Q9UK80	4	BLM	P54132	5
USP16	Q9Y5T5	4	BRCA1	P38398	5
USP14	P54578	4	BRCA2	P51587	5
UBE2Z	Q9H832	4	BRCC3	P46736	5
UBE2V1	Q13404	4	BRE	Q9NXR7	5
UBE2A	P49459	4	C21orf59	P57076	5
TTLL5	Q6EMB2	4	CAPZA1	P52907	5
TTI1	O43156	4	CBX1	P83916	5
TSSC1	Q53HC9	4	CBX3	Q13185	5
TSPYL2	Q9H2G4	4	CCNA1	P78396	5
TRPC6	Q9Y210	4	CCNA2	P20248	5
TRIOBP	Q9H2D6	4	CCNE1	P24864	5
TRAF4	Q9BUZ4	4	CDC45	O75419	5
TRA2B	P62995	4	CDC6	Q99741	5
TPM2	P07951	4	CDC7	O00311	5
TPM1	P09493	4	CDK2	P24941	5
TPI1	P60174	4	CDK6	Q00534	5
TOR1AIP1	Q5JTV8	4	CDK7	P50613	5
TGFB1	P01137	4	CDK9	P50750	5
TCOF1	Q13428	4	CDKN1A	P38936	5
TBL2	Q9Y4P3	4	CDKN1B	P46527	5
SYT1	P21579	4	CDKN2A	P42771	5

STX7	O15400	4	CDKN2C	P42773	5
STX5	Q13190	4	CDT1	Q9H211	5
STX4	Q12846	4	CDV3	Q9UKY7	5
STX1A	Q16623	4	CHAF1B	Q13112	5
STMN1	P16949	4	CHD1L	Q86WJ1	5
STK3	Q13188	4	CHEK1	O14757	5
STK11IP	Q8N1F8	4	CLSPN	Q9HAW4	5
SSSCA1	O60232	4	COBRA1	Q8WX92	5
SSBP1	Q04837	4	COIL	P38432	5
SRF	P11831	4	COPS2	P61201	5
SPAG5	Q96R06	4	COPS6	Q7L5N1	5
SOX10	P56693	4	CREB5	Q02930	5
SORT1	Q99523	4	CTR9	Q6PD62	5
SOCS5	O75159	4	CUL4A	Q13619	5
SNCAIP	Q9Y6H5	4	CUL4B	Q13620	5
SMURF1	Q9HCE7	4	DBF4	Q9UBU7	5
SMS	P52788	4	DDB1	Q16531	5
SLC2A1	P11166	4	DDB2	Q92466	5
SIRT3	Q9NTG7	4	DDX3X	O00571	5
SGTA	O43765	4	DNAJA3	Q96EY1	5
SERTAD1	Q9UHV2	4	DNAJB11	Q9UBS4	5
SERPINA5	P05154	4	DRG1	Q9Y295	5
SEPT7	Q16181	4	DTL	Q9NZJ0	5
SELS	SELS	4	E2F1	Q01094	5
SELE	P16581	4	E2F4	Q16254	5
SCT	P09683	4	EIF1B	O60739	5
SCAP	Q12770	4	ELF3	P78545	5
SATB2	Q9UPW6	4	EMD	P50402	5
SALL2	Q9Y467	4	ERCC1	P07992	5
RXFP2	Q8WXD0	4	ERCC2	P18074	5
RXFP1	Q9HBX9	4	ERCC8	Q13216	5
RPS6KB1	P23443	4	ETFA	P13804	5
RPS6KA2	Q15349	4	FANCA	O15360	5
RNH1	P13489	4	FANCC	Q00597	5
RLN2	P04090	4	FANCG	O15287	5
RICTOR	Q6R327	4	FANCI	Q9NVI1	5
RGS6	P49758	4	FANCM	Q8IYD8	5
RAMP3	O60896	4	FBXO21	O94952	5
RAMP1	O60894	4	FEN1	P39748	5
QRICH2	Q9H0J4	4	FIGNL1	FIGNL1	5
QRICH1	Q2TAL8	4	FOXM1	Q08050	5
Q14790	Q14790	4	FSCN1	Q16658	5
PTPRK	Q15262	4	GANAB	Q14697	5
PTPRG	P23470	4	GINS1	Q14691	5
PTGIR	P43119	4	GOLGA2	Q08379	5
PTGER4	P35408	4	GOT2	P00505	5
PTGER2	P43116	4	GSTM3	P21266	5
PSENEN	Q9NZ42	4	GTF2H3	Q13889	5
PSAT1	Q9Y617	4	H2AFX	P16104	5
PRPS1L1	P21108	4	HEATR1	Q9H583	5
PRNP	P04156	4	HIST1H1A	Q02539	5
PRKCH	P24723	4	HIVEP1	P15822	5
PRKAA2	P54646	4	HLA-B	P30480	5
PPP3CA	Q08209	4	HOMEZ	Q8IX15	5
PPIL5	PPIL5	4	HPRT1	P00492	5
PPIA	P62937	4	HUWE1	Q7Z6Z7	5
PPFIBP2	Q8ND30	4	HYRC	HYRC	5
POU5F1	Q01860	4	ILK	Q13418	5
POLB	P06746	4	KAT7	O95251	5

PLD1	Q13393	4	KCTD13	Q8WZ19	5
PKMYT1	Q99640	4	KLHL8	Q9P2G9	5
PIWIL1	Q96J94	4	LEO1	Q8WVC0	5
PGM1	P36871	4	LMNA	P02545	5
PARD6A	Q9NPB6	4	LMNB1	P20700	5
P4HA1	P13674	4	MAGED1	Q9Y5V3	5
OXSR1	O95747	4	MAPRE2	Q15555	5
OTUB1	Q96FW1	4	MCC	P23508	5
ODF1	Q14990	4	MCL1	Q07820	5
NR4A2	P43354	4	MCM10	Q7L590	5
NOTCH3	Q9UM47	4	MCM2	P49736	5
NID1	P14543	4	MCM3	P25205	5
NHP2	Q9NX24	4	MCM4	P33991	5
NFYC	Q13952	4	MCM5	P33992	5
NFE2L2	Q16236	4	MCM6	Q14566	5
NEDD9	Q14511	4	MCM7	P33993	5
NCAPH	Q15003	4	MCM8	Q9UJA3	5
NAPA	P54920	4	METTL13	Q8N6R0	5
MYO6	Q9UM54	4	MLH1	P40692	5
MORC4	Q8TE76	4	MPG	P29372	5
MLF2	Q15773	4	MSH2	P43246	5
MC5R	P33032	4	MSH6	P52701	5
MC1R	Q01726	4	MYBL2	P10244	5
MAPKBP1	O60336	4	MYC	P01106	5
MAPKAPK3	Q16644	4	MYH9	P35579	5
MAPK8IP1	Q9UQF2	4	MYOC	Q99972	5
MAP3K11	Q16584	4	MYST2	MYST2	5
MAG	P20916	4	NBN	O60934	5
MACF1	Q9UPN3	4	NDUFAF3	Q9BU61	5
LIN7A	O14910	4	NEK6	Q9HC98	5
LHB	P01229	4	NGLY1	Q96IV0	5
LDLR	P01130	4	NUMA1	Q14980	5
KRT17	Q04695	4	ORC2	Q13416	5
KRIT1	O00522	4	ORC3	Q9UBD5	5
KLK2	P20151	4	ORC4	O43929	5
KLK13	Q9UKR3	4	ORC5	O43913	5
KITLG	P21583	4	ORC6	Q9Y5N6	5
KIF14	Q15058	4	P4HA1	P13674	5
JPH1	Q9HDC5	4	PARP10	Q53GL7	5
ITGB2	P05107	4	PAXIP1	Q6ZW49	5
ITGA2	P17301	4	PCID2	Q5JVF3	5
ISYNA1	Q9NPH2	4	PCNA	P12004	5
IRS2	Q9Y4H2	4	PICK1	Q9NRD5	5
INSIG2	Q9Y5U4	4	PLEKHG4	Q58EX7	5
INO80B	Q9C086	4	POGZ	Q7Z3K3	5
IL2RG	P31785	4	POLA1	P09884	5
IL2RB	P14784	4	POLA2	Q14181	5
IAPP	P10997	4	POLB	P06746	5
HTRA2	O43464	4	POLE2	P56282	5
HTR7	P34969	4	POLE	Q07864	5
HSPA1B	HSPA1B	4	POT1	Q9NUX5	5
HSD17B3	P37058	4	PRIM1	P49642	5
HNRPLL	Q8WVV9	4	PRIM2	P49643	5
HNF4A	P41235	4	PRKAB1	Q9Y478	5
HMOX2	P30519	4	PRKDC	P78527	5
HMGN3	Q15651	4	PRPS1L1	P21108	5
HLA-C	P30508	4	RAD18	Q9NS91	5
GRIN2B	Q13224	4	RAD23B	P54727	5
GPBAR1	Q8TDU6	4	RAD51	Q06609	5

GMIP	Q9P107	4	RAG1	P15918	5
GLO1	Q04760	4	RAG2	P55895	5
GFI1	Q99684	4	RB1	P06400	5
GAB2	Q9UQC2	4	RBBP4	Q09028	5
FTL	P02792	4	RBL1	P28749	5
FSHB	P01225	4	RBL2	Q08999	5
FLOT1	O75955	4	RBM48	Q5RL73	5
FHL5	Q5TD97	4	RECQL4	O94761	5
FGFR3	P22607	4	RECQL5	O94762	5
FGF2	P09038	4	RFC1	P35251	5
FGF18	O76093	4	RFC4	P35249	5
FGB	P02675	4	RNF185	Q96GF1	5
FER	P16591	4	RPA1	P27694	5
FCHSD2	O94868	4	RPA2	P15927	5
FBXO21	O94952	4	RPA3	P35244	5
FBXO11	Q86XK2	4	RRM2	P31350	5
FAM134A	Q8NC44	4	RSBN1L	RSBN1L	5
ETFB	P38117	4	S100A7	P31151	5
ERG	P11308	4	SAP130	Q9H0E3	5
ERBB2IP	Q96RT1	4	SEZ6L2	Q6UXD5	5
ELF1	P32519	4	SMC3	Q9UQE7	5
EIF4H	Q15056	4	SMC6	Q96SB8	5
EGR2	P11161	4	SPAG1	Q07617	5
EEA1	Q15075	4	SSRP1	Q08945	5
EDF1	O60869	4	SUMO2	P61956	5
DR1	Q01658	4	SUPT5H	O00267	5
DNM1	Q05193	4	TARDBP	Q13148	5
DNAJA2	O60884	4	TDRD7	Q8NHU6	5
DMWD	Q09019	4	TERF1	P54274	5
DLG1	Q12959	4	TERF2IP	Q9NYB0	5
DHX16	O60231	4	TERF2	Q15554	5
DGKZ	Q13574	4	TFDP1	Q14186	5
DEDD	O75618	4	TFDP2	Q14188	5
DDX4	Q9NQI0	4	TKT	P29401	5
DDIT3	P35638	4	TLE1	Q04724	5
DBN1	Q16643	4	TMPO	P42166	5
DAZ1	Q9NQZ3	4	TOP2A	P11388	5
CYP17A1	P05093	4	TOPBP1	Q92547	5
CTTN	Q14247	4	TOR1AIP1	Q5JTV8	5
CSF2RB	P32927	4	TP53BP1	Q12888	5
CSF2RA	P15509	4	TRAF3IP1	Q8TDR0	5
CSF2	P04141	4	TSPYL5	Q86VY4	5
CRHR2	Q13324	4	UBA3	Q8TBC4	5
CRB1	P82279	4	UBE2A	P49459	5
COMT	P21964	4	UBE2T	Q9NPD8	5
CHMP5	Q9NZZ3	4	UBE3A	Q05086	5
CHIC2	Q9UKJ5	4	UBN2	Q6ZU65	5
CGA	P01215	4	UBTF	P17480	5
CEP350	Q5VT06	4	UCHL5	Q9Y5K5	5
CDKL5	O76039	4	UHRF2	Q96PU4	5
CD36	P16671	4	UIMC1	Q96RL1	5
CD19	P15391	4	UNG	P13051	5
CCT6B	Q92526	4	USP1	O94782	5
CASP2	P42575	4	USP25	Q9UHP3	5
CAPNS1	P04632	4	USP7	Q93009	5
CAMK2B	Q13554	4	VPRBP	Q9Y4B6	5
CALD1	Q05682	4	WAPAL	Q7Z5K2	5
CALCRL	Q16602	4	WBSCR22	O43709	5
CALCB	P10092	4	WDR5	P61964	5

CACNA1C	Q13936	4	WDR61	Q9GZS3	5
C9orf78	Q9NZ63	4	WDTC1	Q8N5D0	5
C19orf62	C19orf62	4	XRCC5	P13010	5
C14orf166	Q9Y224	4	ZBTB32	Q9Y2Y4	5
C12orf10	Q9HB07	4	ZMYM1	ZMYM1	5
BRD1	O95696	4	ZNF232	Q9UNY5	5
BRAF	P15056	4	ZNF541	Q9H0D2	5
BMPR1B	O00238	4	ABCA1	O95477	6
BIRC7	Q96CA5	4	AKAP2	Q9Y2D5	6
BHLHE40	O14503	4	**APLP2**	**Q06481**	**6**
BCL10	O95999	4	ARFGAP1	Q8N6T3	6
BCAT1	P54687	4	ASCC1	Q8N9N2	6
BAT3	BAT3	4	ASCC3	Q8N3C0	6
B4GALT1	P15291	4	ATP5A1	P25705	6
ATF3	P18847	4	ATRN	O75882	6
ASRGL1	Q7L266	4	BASP1	P80723	6
ANLN	Q9NQW6	4	BCL10	O95999	6
ANKLE2	Q86XL3	4	CCNDBP1	O95273	6
AMPH	P49418	4	CHL1	O00533	6
ALS2CR11	Q53TS8	4	CLCN7	P51798	6
ALDH18A1	P54886	4	COPS4	Q9BT78	6
ALCAM	Q13740	4	COPS5	Q92905	6
AKT2	P31751	4	COPS7B	Q9H9Q2	6
AIFM1	O95831	4	CRB1	P82279	6
ADNP2	Q6IQ32	4	CSF2	P04141	6
ADCYAP1	P18509	4	DACH1	Q9UI36	6
ACTC1	P68032	4	DAPK1	P53355	6
ABCA1	O95477	4	DCAF11	Q8TEB1	6
AARS	P49588	4	DCAF4	Q8WV16	6
ZZEF1	O43149	3	DOCK7	Q96N67	6
ZBTB5	O15062	3	EDF1	O60869	6
WIPF2	Q8TF74	3	EEF2K	O00418	6
WIBG	Q9BRP8	3	ELOF1	P60002	6
WFS1	O76024	3	EPB41L2	O43491	6
WDR6	Q9NNW5	3	ETS2	P15036	6
WDR16	Q8N1V2	3	FAM188A	Q9H8M7	6
WBP7	Q9UMN6	3	FBXO11	Q86XK2	6
VEZT	Q9HBM0	3	FBXO17	Q96EF6	6
VAMP7	P51809	3	FBXO22	Q8NEZ5	6
USPL1	Q5W0Q7	3	FBXO46	Q6PJ61	6
USP25	Q9UHP3	3	FBXW7	Q969H0	6
USO1	O60763	3	FOSL1	P15407	6
UBXN6	Q9BZV1	3	GCLM	P48507	6
UBA3	Q8TBC4	3	GFER	P55789	6
TSSK4	Q6SA08	3	GNB2L1	P63244	6
TSPAN4	O14817	3	GPS1	Q13098	6
TSKS	Q9UJT2	3	HDAC9	Q9UKV0	6
TSC22D3	Q99576	3	HERC1	Q15751	6
TRPC7	Q9HCX4	3	HHEX	Q03014	6
TRIP4	Q15650	3	HNF4G	Q14541	6
TRAM1	Q15629	3	IGFBP7	Q16270	6
TPM4	P67936	3	INADL	Q8NI35	6
TNP1	TNP1	3	JUN	P05412	6
THBS1	P07996	3	KLHDC5	Q9P2K6	6
TGFBR3	Q03167	3	KLHL15	Q96M94	6
TGFB2	P61812	3	KLHL18	O94889	6
TGFB1I1	O43294	3	KLHL24	Q6TFL4	6
TAPBP	O15533	3	KLHL9	Q9P2J3	6
SUGT1	Q9Y2Z0	3	LIN7C	Q9NUP9	6

STX8	Q9UNK0	3	LRRC14	Q15048	6
STMN2	Q93045	3	MAFB	Q9Y5Q3	6
STK4	Q13043	3	MAFG	O15525	6
STK39	Q9UEW8	3	MAFK	O60675	6
STIM1	Q13586	3	MAP2K2	P36507	6
STAT4	Q14765	3	MAPK14	Q16539	6
SRP72	O76094	3	MAPKAPK5	Q8IW41	6
SRP54	P61011	3	MAPRE3	Q9UPY8	6
SPATA24	Q86W54	3	MIF	P14174	6
SOX8	P57073	3	MLL3	Q8NEZ4	6
SOX30	O94993	3	MPDZ	O75970	6
SNX17	Q15036	3	MPP5	Q8N3R9	6
SNRK	Q9NRH2	3	NACA	Q13765	6
SNAPC5	O75971	3	NAT14	Q8WUY8	6
SNAP23	O00161	3	NEFM	P07197	6
SLC4A8	Q2Y0W8	3	NFE2L1	Q14494	6
SLAIN2	Q9P270	3	NR4A3	Q92570	6
SERPINF2	P08697	3	NR5A2	O00482	6
SELP	P16109	3	NTRK3	Q16288	6
SCAPER	Q9BY12	3	PACS1	Q6VY07	6
SARS	P49591	3	PLA2G4A	P47712	6
SARS2	Q9NP81	3	PPOX	P50336	6
S100A13	Q99584	3	PPP3CB	P16298	6
RTN4R	Q9BZR6	3	RNF187	Q5TA31	6
RSBN1L	RSBN1L	3	RNF20	Q5VTR2	6
RORA	P35398	3	RPS6KA2	Q15349	6
RNFT1	Q5M7Z0	3	S100A7A	S100A7A	6
RNF5	Q99942	3	SHISA5	Q8N114	6
RNF146	Q9NTX7	3	SLC4A8	Q2Y0W8	6
RGS2	P41220	3	SOX30	O94993	6
RECQL	P46063	3	SS18	Q15532	6
RBM48	Q5RL73	3	STOML2	Q9UJZ1	6
RAB3GAP2	Q9H2M9	3	TCF20	Q9UGU0	6
RAB3A	P20336	3	TCL1A	P56279	6
PRR5	P85299	3	TGIF1	Q15583	6
PROS1	P07225	3	TMOD1	P28289	6
PRKG1	Q13976	3	TOR1AIP2	Q8NFQ8	6
PRKCSH	P14314	3	TPM1	P09493	6
PRKACB	P22694	3	UCHL3	P15374	6
PPP3CB	P16298	3	ZNF282	Q9UDV7	6
PPP1R15A	O75807	3	ACTR1A	P61163	7
PPP1R13B	Q96KQ4	3	AKAP9	Q99996	7
PPP1R12B	O60237	3	ALMS1	Q8TCU4	7
PPP1R12A	O14974	3	AMOTL1	Q8IY63	7
PPA1	Q15181	3	ATP2B1	P20020	7
POU2F2	P09086	3	AZI1	Q9UPN4	7
PLOD1	Q02809	3	B4GALT1	P15291	7
PLK3	Q9H4B4	3	CALB2	P22676	7
PLAC8	Q9NZF1	3	CCP110	O43303	7
PLA2G2A	P14555	3	CD276	Q5ZPR3	7
PIK3CD	O00329	3	CDK11A	Q9UQ88	7
PGM2	Q96G03	3	CDK5RAP2	Q96SN8	7
PGK1	P00558	3	CENPJ	Q9HC77	7
PGAM5	Q96HS1	3	CEP135	Q66GS9	7
PDIA4	P13667	3	CEP152	O94986	7
PDHX	O00330	3	CEP164	Q9UPV0	7
PAX8	Q06710	3	CEP192	Q8TEP8	7
PASK	Q96RG2	3	CEP250	Q9BV73	7
PACSIN3	Q9UKS6	3	CEP290	O15078	7

P01213	P01213	3	CEP350	Q5VT06	7
OTUD5	Q96G74	3	CEP55	Q53EZ4	7
OGG1	O15527	3	CEP57	Q86XR8	7
ODF2L	Q9ULJ1	3	CEP63	Q96MT8	7
NUMB	P49757	3	CEP70	Q8NHQ1	7
NUMBL	Q9Y6R0	3	CEP72	Q9P209	7
NTRK2	Q16620	3	CEP76	Q8TAP6	7
NRG4	Q8WWG1	3	CEP78	Q5JTW2	7
NRG1	Q02297	3	CETN2	P41208	7
NQO2	P16083	3	CKAP5	Q14008	7
NOTCH2	Q04721	3	CLASP1	Q7Z460	7
NFASC	O94856	3	CNTN2	Q02246	7
NECAP1	Q8NC96	3	CNTRL	Q7Z7A1	7
NDN	Q99608	3	COPS7A	Q9UBW8	7
NCKIPSD	Q9NZQ3	3	COPS8	Q99627	7
MYSM1	Q5VVJ2	3	CSNK1D	P48730	7
MYOC	Q99972	3	CSNK1E	P49674	7
MSTO1	Q9BUK6	3	DAB1	O75553	7
MST4	Q9P289	3	DCTN1	Q14203	7
MPP5	Q8N3R9	3	DCTN2	Q13561	7
MORG1	MORG1	3	DCTN3	O75935	7
MMEL1	Q495T6	3	DCTN5	Q9BTE1	7
MLL5	Q8IZD2	3	DYNC1H1	Q14204	7
MLL4	MLL4	3	DYNC1I2	Q13409	7
MLF1	P58340	3	DYNLL1	P63167	7
MIB1	Q86YT6	3	ERBB2IP	Q96RT1	7
MEX3B	Q6ZN04	3	FGFR1OP	O95684	7
MCL1	Q07820	3	HAUS2	Q9NVX0	7
MAP7	Q14244	3	HSP90AA1	P07900	7
MAP4	P27816	3	KRT18	P05783	7
MALT1	Q9UDY8	3	MAPRE1	Q15691	7
LRRK2	Q5S007	3	MGEA5	O60502	7
LRP2	P98164	3	MMP14	P50281	7
LRMP	Q12912	3	NDE1	Q9NXR1	7
LINGO1	Q96FE5	3	NEDD1	Q8NHV4	7
LDLRAP1	Q5SW96	3	NEK2	P51955	7
LAMP2	P13473	3	NINL	Q9Y2I6	7
LAMB1	P07942	3	ODF1	Q14990	7
LAMA1	P25391	3	ODF2	Q5BJF6	7
KRI1	Q8N9T8	3	OFD1	O75665	7
KPNA5	O15131	3	PAFAH1B1	P43034	7
KL	Q9UEF7	3	PCM1	Q15154	7
KLK6	Q92876	3	PCNT	O95613	7
KLK10	O43240	3	PLK4	O00444	7
KLHL22	Q53GT1	3	PPP1R2	P41236	7
KLHL13	Q9P2N7	3	PPP2R1A	P30153	7
KLHDC3	Q9BQ90	3	PRKACA	P17612	7
KLB	Q86Z14	3	PRKAR2B	P31323	7
KCNIP3	Q9Y2W7	3	RYK	P34925	7
ITGB8	P26012	3	SDCCAG8	Q86SQ7	7
ITGA4	P13612	3	SFI1	A8K8P3	7
IQCG	Q9H095	3	SLAIN2	Q9P270	7
INSR	P06213	3	SPAG5	Q96R06	7
IL6ST	P40189	3	SSNA1	O43805	7
IL5RA	Q01344	3	TSGA14	Q9BYV8	7
IL16	Q14005	3	TUBA1A	Q71U36	7
IFITM1	P13164	3	TUBA4A	P68366	7
HYOU1	Q9Y4L1	3	TUBB2C	P68371	7
HSPG2	P98160	3	TUBB4	P04350	7

HSPA14	Q0VDF9	3	TUBB	P07437	7
HSDL2	Q6YN16	3	TUBG1	P23258	7
HSD17B10	Q99714	3	TUBG2	Q9NRH3	7
HOOK2	Q96ED9	3	TUBGCP2	Q9BSJ2	7
HES1	Q14469	3	TUBGCP3	Q96CW5	7
HDAC9	Q9UKV0	3	TUBGCP4	Q9UGJ1	7
GSS	P48637	3	TUBGCP5	Q96RT8	7
GSR	P00390	3	TUBGCP6	Q96RT7	7
GSK3A	P49840	3	WWTR1	Q9GZV5	7
GRM4	Q14833	3	YWHAE	P62258	7
GRIP1	Q9Y3R0	3	YWHAH	Q04917	7
GRIA1	P42261	3	ADD1	P35611	8
GPR37	O15354	3	AKAP1	Q92667	8
GOLGA3	Q08378	3	AKAP8	O43823	8
GOLGA2	Q08379	3	AKT2	P31751	8
GART	P22102	3	AKT3	Q9Y243	8
GALK1	P51570	3	ALS2CR12	Q96Q35	8
GADD45B	O75293	3	APPL1	Q9UKG1	8
GAD1	Q99259	3	ATG4D	Q86TL0	8
FRYL	O94915	3	ATP6V1E2	Q96A05	8
FRS2	Q8WU20	3	BCDIN3D	Q7Z5W3	8
FNTA	P49354	3	BIRC6	Q9NR09	8
FLOT2	Q14254	3	BIRC7	Q96CA5	8
FGFR2	P21802	3	BLNK	Q8WV28	8
FGF9	P31371	3	BTF3	P20290	8
FGF6	P10767	3	C17orf56	Q96N21	8
FGF4	P08620	3	CAMK4	Q16566	8
FGF22	Q9HCT0	3	CASP3	P42574	8
FGF1	P05230	3	CAST	P20810	8
FGF19	O95750	3	CAV1	Q03135	8
FDXR	P22570	3	CBL	P22681	8
FAM98A	Q8NCA5	3	CD2AP	Q9Y5K6	8
F8	P00451	3	CD55	P08174	8
F10	P00742	3	CD93	Q9NPY3	8
EXOC6	Q8TAG9	3	CDK11B	P21127	8
ERO1L	Q96HE7	3	CFDP1	Q9UEE9	8
ERN1	O75460	3	CGN	Q9P2M7	8
ERLIN2	O94905	3	CNN2	Q99439	8
EREG	O14944	3	CRKL	P46109	8
ERAP2	Q6P179	3	CSF1	P09603	8
ERAP1	Q9NZ08	3	CSF2RA	P15509	8
EIF2AK3	Q9NZJ5	3	CSF2RB	P32927	8
EEF2K	O00418	3	DBNL	Q9UJU6	8
EDC4	Q6P2E9	3	DCC	P43146	8
DVL2	O14641	3	DCLK1	O15075	8
DVL1	O14640	3	DIABLO	Q9NR28	8
DPH1	Q9BZG8	3	DIP2B	Q9P265	8
DOK1	Q99704	3	DLAT	P10515	8
DLGAP4	Q9Y2H0	3	DLGAP4	Q9Y2H0	8
DKKL1	Q9UK85	3	DOK1	Q99704	8
DHPS	P49366	3	DPH2	Q9BQC3	8
DFFB	O76075	3	DPYSL5	Q9BPU6	8
DFFA	O00273	3	DSG2	Q14126	8
DCXR	Q7Z4W1	3	DSG3	P32926	8
DCAF4	Q8WV16	3	EPHA8	P29322	8
DAZL	Q92904	3	EZR	P15311	8
DAZ4	Q86SG3	3	FES	P07332	8
DAPK1	P53355	3	FGF18	O76093	8
DALRD3	Q5D0E6	3	FGF19	O95750	8

DAB1	O75553	3	FGF1	P05230	8
CYP51A1	Q16850	3	FGF22	Q9HCT0	8
CTSH	P09668	3	FGF2	P09038	8
CTSB	P07858	3	FGF4	P08620	8
CST3	P01034	3	FGF6	P10767	8
CSNK1G1	Q9HCP0	3	FGF9	P31371	8
CRYZ	Q08257	3	FGFR1	P11362	8
CRYAB	P02511	3	FGFR2	P21802	8
CREB5	Q02930	3	FGFR3	P22607	8
CREB3	O43889	3	FGFR4	P22455	8
COX7A2	P14406	3	FHOD1	Q9Y613	8
COPS8	Q99627	3	FLOT1	O75955	8
COPS7A	Q9UBW8	3	FNBP1	Q96RU3	8
COPS3	Q9UNS2	3	FNTA	P49354	8
COPS2	P61201	3	FRS2	Q8WU20	8
COL25A1	Q9BXS0	3	FRS3	O43559	8
CNTN1	Q12860	3	FYN	P06241	8
CLYBL	Q8N0X4	3	GAB1	Q13480	8
CLU	P10909	3	GAB2	Q9UQC2	8
CEP55	Q53EZ4	3	GAD1	Q99259	8
CDK5R1	Q15078	3	GAS2	O43903	8
CD93	Q9NPY3	3	GCLC	P48506	8
CD8A	P01732	3	GLRX	P35754	8
CD55	P08174	3	GRIPAP1	Q4V328	8
CD14	P08571	3	HAX1	O00165	8
CBFB	Q13951	3	HCLS1	P14317	8
CAST	P20810	3	HEY2	Q9UBP5	8
CARS	P49589	3	HNF4A	P41235	8
CARD11	Q9BXL7	3	HRAS	P01112	8
CAPN11	Q9UMQ6	3	IL2RB	P14784	8
CAMP	P49913	3	IL2RG	P31785	8
CAMK4	Q16566	3	IL3RA	P26951	8
CAMK1	Q14012	3	IL4R	P24394	8
CAD	P27708	3	IL5RA	Q01344	8
C1D	Q13901	3	IL7R	P16871	8
C15orf39	Q6ZRI6	3	INPP5D	Q92835	8
BLNK	Q8WV28	3	INSR	P06213	8
BLMH	Q13867	3	IRS1	P35568	8
BIRC6	Q9NR09	3	IRS2	Q9Y4H2	8
BCL2L11	O43521	3	JAK1	P23458	8
BACE1	P56817	3	JAK2	O60674	8
ATF6	P18850	3	KIRREL	Q96J84	8
ATF1	P18846	3	KITLG	P21583	8
ASCC2	Q9H1I8	3	KIT	P10721	8
ASB9	Q96DX5	3	KLB	Q86Z14	8
APOE	P02649	3	KL	Q9UEF7	8
APOA1	P02647	3	KRAS	P01116	8
APOA1BP	Q8NCW5	3	LCK	P06239	8
APH1A	Q96BI3	3	LRBA	P50851	8
APBA1	Q02410	3	LYN	P07948	8
AP1M2	Q9Y6Q5	3	MAP4K5	Q9Y4K4	8
ANKIB1	Q9P2G1	3	MAPK10	P53779	8
ALDOA	P04075	3	MAPK1	P28482	8
ADAP1	O75689	3	MAPK8IP3	Q9UPT6	8
ADAM2	Q99965	3	MAPK8	P45983	8
ACIN1	Q9UKV3	3	MAPK9	P45984	8
ACHE	P22303	3	MAPKBP1	O60336	8
ABHD2	P08910	3	MAPT	P10636	8
AASS	Q9UDR5	3	MAZ	P56270	8

ZNHIT2	Q9UHR6	2	MET	P08581	8
ZNF541	Q9H0D2	2	MSN	P26038	8
ZNF282	Q9UDV7	2	MST1R	Q04912	8
ZNF274	Q96GC6	2	MST4	Q9P289	8
ZNF200	P98182	2	NCKIPSD	Q9NZQ3	8
ZMYM1	ZMYM1	2	NDUFS1	P28331	8
ZFC3H1	O60293	2	NEDD4	P46934	8
WNK3	Q9BYP7	2	NFATC3	Q12968	8
WDR35	Q9P2L0	2	NFE2L2	Q16236	8
VTI1B	Q9UEU0	2	NGFRAP1	Q00994	8
VTI1A	Q96AJ9	2	NMT1	P30419	8
VAPA	Q9P0L0	2	NMT2	O60551	8
UTRN	P46939	2	NPHS1	O60500	8
USP6	P35125	2	NR5A1	Q13285	8
USP3	Q9Y6I4	2	NRAS	P01111	8
USP32	Q8NFA0	2	NTRK1	P04629	8
USP30	Q70CQ3	2	NTRK2	Q16620	8
UNC5B	Q8IZJ1	2	OCLN	Q16625	8
UCHL3	P15374	2	OSTF1	Q92882	8
UBN2	Q6ZU65	2	PARG99	PARG99	8
UBE4B	O95155	2	PARG	Q86W56	8
UBE2M	P61081	2	PARK2	O60260	8
UBE2J2	Q8N2K1	2	PAWR	Q96IZ0	8
UBE2J1	Q9Y385	2	PDAP1	Q13442	8
UBAP2	Q5T6F2	2	PDE10A	Q9Y233	8
TUBA3D	TUBA3D	2	PDE5A	O76074	8
TTC1	Q99614	2	PDE6A	P16499	8
TSPYL5	Q86VY4	2	PDGFRA	P16234	8
TSPY3	TSPY3	2	PDGFRB	P09619	8
TSPY1	Q01534	2	PICALM	Q13492	8
TSPAN6	O43657	2	PIK3CB	P42338	8
TRPM7	Q96QT4	2	PIK3CD	O00329	8
TRIP12	Q14669	2	PIK3R1	P27986	8
TRIM38	O00635	2	PIP5K1A	Q99755	8
TRIB1	Q96RU8	2	PKN2	Q16513	8
TPD52L3	Q96J77	2	PLA2G6	O60733	8
TPD52L2	O43399	2	PLAC8	Q9NZF1	8
TMUB1	Q9BVT8	2	PLCG1	P19174	8
TMOD1	P28289	2	PLK3	Q9H4B4	8
TMEM62	Q0P6H9	2	POP1	Q99575	8
TMEFF2	Q9UIK5	2	PPP1R11	O60927	8
THOP1	P52888	2	PPP1R12A	O14974	8
TG	P01266	2	PRDX2	P32119	8
TGIF1	Q15583	2	PRKCD	Q05655	8
TES	Q9UGI8	2	PRKCQ	Q04759	8
TEK	Q02763	2	PRKCZ	Q05513	8
TDRD7	Q8NHU6	2	PRKRIP1	Q9H875	8
TDGF1	P13385	2	PTEN	P60484	8
TCL1A	P56279	2	PTK2	Q05397	8
TCF19	Q9Y242	2	PTPN11	Q06124	8
SV2A	Q7L0J3	2	PTPRE	P23469	8
STX6	O43752	2	PTPRG	P23470	8
STOML2	Q9UJZ1	2	PTPRK	Q15262	8
STAR	P49675	2	PXN	P49023	8
SS18	Q15532	2	RASA1	P20936	8
SPP1	P10451	2	RET	P07949	8
SPARCL1	Q14515	2	RIMS1	Q86UR5	8
SPAG1	Q07617	2	RNF219	Q5W0B1	8
SPACA3	Q8IXA5	2	RNF34	Q969K3	8

SPA17	Q15506	2	RNF7	Q9UBF6	8
SORL1	Q92673	2	S100A13	Q99584	8
SNAP29	O95721	2	SARS2	Q9NP81	8
SNAP25	P60880	2	SARS	P49591	8
SMUG1	Q53HV7	2	SCG3	Q8WXD2	8
SMPD2	O60906	2	SELP	P16109	8
SMC6	Q96SB8	2	SHC1	P29353	8
SLK	Q9H2G2	2	SLC1A2	P43004	8
SLC5A1	P13866	2	SLC4A1AP	Q9BWU0	8
SLC25A31	Q9H0C2	2	SLFN5	Q08AF3	8
SLC17A7	Q9P2U7	2	SLK	Q9H2G2	8
SIVA1	O15304	2	SNCG	O76070	8
SHE	Q5VZ18	2	SNX17	Q15036	8
SERPINA3	P01011	2	SOCS5	O75159	8
SEL1L	Q9UBV2	2	SOX8	P57073	8
SDCCAG3	Q96C92	2	SPTAN1	Q13813	8
SCFD1	Q8WVM8	2	SPTBN1	Q01082	8
SCAF4	O95104	2	SRC	P12931	8
RSPH3	Q86UC2	2	SRF	P11831	8
RNF219	Q5W0B1	2	STAT6	P42226	8
RIOK1	Q9BRS2	2	SYT1	P21579	8
RIMS1	Q86UR5	2	TCOF1	Q13428	8
RGS20	O76081	2	TEK	Q02763	8
RET	P07949	2	TES	Q9UGI8	8
REEP6	Q96HR9	2	THOP1	P52888	8
RASGRP2	Q7LDG7	2	TRIP13	Q15645	8
RASGRP1	O95267	2	UBE4B	O95155	8
RASA2	Q15283	2	UBR7	Q8N806	8
RAGE	RAGE	2	UNC5B	Q8IZJ1	8
RAB4A	P20338	2	USP32	Q8NFA0	8
PTPN13	Q12923	2	USP44	Q9H0E7	8
PTGFRN	Q9P2B2	2	USP6	P35125	8
PTCH1	Q13635	2	VAV1	P15498	8
PRPSAP1	Q14558	2	WNK3	Q9BYP7	8
PRPS2	P11908	2	XRCC4	Q13426	8
PRDM4	Q9UKN5	2	YES1	P07947	8
PPP1R2	P41236	2	ZFC3H1	O60293	8
PPM1E	Q8WY54	2	ZNF746	Q6NUN9	8
PPID	Q08752	2	A2M	P01023	9
PPFIA1	Q13136	2	ABL1	P00519	9
POP1	Q99575	2	ADAM2	Q99965	9
PLXNA1	Q9UIW2	2	AKAP3	O75969	9
PLEKHG4	Q58EX7	2	ALCAM	Q13740	9
PLCH1	Q4KWH8	2	AMOT	Q4VCS5	9
PLBD2	Q8NHP8	2	ANKS1A	Q92625	9
PKN2	Q16513	2	AP1M2	Q9Y6Q5	9
PKD2	Q13563	2	AP2A1	O95782	9
PITRM1	Q5JRX3	2	AP2S1	P53680	9
PIH1D2	PIH1D2	2	AP4S1	Q9Y587	9
PICK1	Q9NRD5	2	APBA3	O96018	9
PICALM	Q13492	2	APBB1	O00213	9
PHF7	PHF7	2	APBB2	Q92870	9
PDXDC1	Q6P996	2	APBB3	O95704	9
PDIA6	Q15084	2	APOE	P02649	9
PDAP1	Q13442	2	ARHGAP19	Q14CB8	9
PCSK7	Q16549	2	ARHGAP28	Q9P2N2	9
PC	P11498	2	ARHGDIA	P52565	9
PAWR	Q96IZ0	2	ARHGDIB	P52566	9
PARP10	Q53GL7	2	ARHGEF11	O15085	9

PARG	Q86W56	2	ARHGEF12	Q9NZN5	9
PAK6	Q9NQU5	2	ARPC5	O15511	9
PACSIN2	Q9UNF0	2	ASRGL1	Q7L266	9
PACS1	Q6VY07	2	ATN1	P54259	9
OSTF1	Q92882	2	BCAR1	P56945	9
OAT	P04181	2	BCAT1	P54687	9
NUDT11	Q96G61	2	BCL2L11	O43521	9
NUCB2	P80303	2	BCR	P11274	9
NTRK3	Q16288	2	BIN1	O00499	9
NR5A2	O00482	2	BNIP2	Q12982	9
NR4A3	Q92570	2	C21orf91	C21orf91	9
NPHS1	O60500	2	CACNA1A	O00555	9
NPAT	Q14207	2	CACNA1C	Q13936	9
NOS1	P29475	2	CALM3	P62158	9
NMT2	O60551	2	CAMLG	P49069	9
NMT1	P30419	2	CCDC97	Q96F63	9
NLRP3	Q96P20	2	CD19	P15391	9
NGLY1	Q96IV0	2	CD44	P16070	9
NEFM	P07197	2	CD46	P15529	9
NDUFAF3	Q9BU61	2	CD63	P08962	9
NDNL2	Q96MG7	2	CD81	P60033	9
NAPG	Q99747	2	CD82	P27701	9
NANOG	Q9H9S0	2	CD99	P14209	9
NACA	Q13765	2	CD9	P21926	9
NAA11	Q9BSU3	2	CDC42	P60953	9
MYOCD	Q8IZQ8	2	CDH2	P19022	9
MYCBPAP	Q8TBZ2	2	CDKL5	O76039	9
MUC1	P15941	2	CLGN	O14967	9
MTFR1	Q15390	2	CLTA	P09496	9
MST1R	Q04912	2	CLYBL	Q8N0X4	9
MPP1	Q00013	2	COL1A1	P02452	9
MMP11	P24347	2	CR2	P20023	9
MLL3	Q8NEZ4	2	CRK	P46108	9
MKRN2	Q9H000	2	CSK	P41240	9
MIF	P14174	2	CSMD2	Q7Z408	9
MGEA5	O60502	2	CYP2E1	P05181	9
MFI2	P08582	2	DEPDC7	Q96QD5	9
MFAP1	P55081	2	DHRS2	Q13268	9
METTL13	Q8N6R0	2	DKKL1	Q9UK85	9
METAP2	P50579	2	DLC1	Q96QB1	9
MBOAT7	Q96N66	2	DLG5	Q8TDM6	9
MAZ	P56270	2	DNM1	Q05193	9
MAT1A	Q00266	2	DTNBP1	Q96EV8	9
MAPRE3	Q9UPY8	2	DUSP15	Q9H1R2	9
MAPK8IP3	Q9UPT6	2	DYNLL2	Q96FJ2	9
MAP4K5	Q9Y4K4	2	ECT2	Q9H8V3	9
MAP4K3	Q8IVH8	2	EGF	P01133	9
MAFK	O60675	2	EGFR	P00533	9
MAFG	O15525	2	EPHB4	P54760	9
MAFB	Q9Y5Q3	2	EPN1	Q9Y6I3	9
LZIC	Q8WZA0	2	ERBB2	P04626	9
LSS	P48449	2	ERBB3	P21860	9
LRP8	Q14114	2	ERBB4	Q15303	9
LRP1B	Q9NZR2	2	F10	P00742	9
LRBA	P50851	2	FGB	P02675	9
LPIN2	Q92539	2	FN1	P02751	9
LIN7C	Q9NUP9	2	GCDH	Q92947	9
LEO1	Q8WVC0	2	GKAP1	Q5VSY0	9
KLHL23	Q8NBE8	2	GMIP	Q9P107	9

KLHDC5	Q9P2K6	2	GPANK1	O95872	9
KIRREL	Q96J84	2	GPX1	P07203	9
KIF20B	Q96Q89	2	GRB2	P62993	9
KIDINS220	Q9ULH0	2	GYPC	P04921	9
KBTBD3	Q8NAB2	2	HBEGF	Q99075	9
INTS7	Q9NVH2	2	HGS	O14964	9
INADL	Q8NI35	2	HSPG2	P98160	9
IL1B	P01584	2	IDE	P14735	9
IGSF8	Q969P0	2	IFIT5	Q13325	9
IDE	P14735	2	IGSF8	Q969P0	9
HSPB8	Q9UJY1	2	ITGA2	P17301	9
HSBP1	O75506	2	ITGA3	P26006	9
HOMEZ	Q8IX15	2	ITGA4	P13612	9
HOMER2	Q9NSB8	2	ITGA6	P23229	9
HNF4G	Q14541	2	ITGB1	P05556	9
HMOX1	P09601	2	ITGB2	P05107	9
HIVEP1	P15822	2	ITGB4	P16144	9
HEY2	Q9UBP5	2	ITGB8	P26012	9
HERC1	Q15751	2	JAK3	P52333	9
HCLS1	P14317	2	KALRN	O60229	9
HBXIP	O43504	2	KIAA1683	Q9H0B3	9
HAX1	O00165	2	KLK13	Q9UKR3	9
HADH	Q16836	2	KLK2	P20151	9
HADHB	P55084	2	KRIT1	O00522	9
GYPC	P04921	2	KRT17	Q04695	9
GRM1	Q13255	2	KRT7	P08729	9
GRIPAP1	Q4V328	2	L1CAM	P32004	9
GRIK3	Q13003	2	LAMA1	P25391	9
GRIA3	P42263	2	LAMA4	Q16363	9
GPX1	P07203	2	LAMB2	P55268	9
GOT2	P00505	2	LAMC1	P11047	9
GORASP1	Q9BQQ3	2	LAMC3	Q9Y6N6	9
GNAO1	P09471	2	LGALS1	P09382	9
GLRX	P35754	2	LINGO1	Q96FE5	9
GLE1	Q53GS7	2	LTBP1	Q14766	9
GCLM	P48507	2	MACF1	Q9UPN3	9
GCLC	P48506	2	MAGI1	Q96QZ7	9
GAS2	O43903	2	MAP2K6	P52564	9
G2E3	Q7L622	2	MAP3K5	Q99683	9
FUNDC2	Q9BWH2	2	MAP3K7	O43318	9
FOSL1	P15407	2	MAP4K3	Q8IVH8	9
FNBP1	Q96RU3	2	MAPK11	Q15759	9
FKBP1A	P62942	2	MAPK8IP2	Q13387	9
FIGNL1	FIGNL1	2	MAPKAPK3	Q16644	9
FHOD1	Q9Y613	2	MAST1	Q9Y2H9	9
FBXW7	Q969H0	2	MCF2	P10911	9
FAM92A1	A1XBS5	2	MEF2A	Q02078	9
FAM83F	Q8NEG4	2	MMP11	P24347	9
F7	P08709	2	MMP2	P08253	9
ETS2	P15036	2	MMP9	P14780	9
ERP44	Q9BS26	2	MPP1	Q00013	9
ERP29	P30040	2	MUC1	P15941	9
ERLIN1	O75477	2	NCK1	P16333	9
EPHB4	P54760	2	NID1	P14543	9
EPB41L3	Q9Y2J2	2	NPHP1	O15259	9
ELN	P15502	2	NUMBL	Q9Y6R0	9
ELAVL2	Q12926	2	NUMB	P49757	9
ECM1	Q16610	2	PARD6A	Q9NPB6	9
ECE1	P42892	2	PKN1	Q16512	9

DYRK3	O43781	2	PLCH1	Q4KWH8	9
DYRK2	Q92630	2	PLD1	Q13393	9
DUT	P33316	2	PLD2	O14939	9
DSG3	P32926	2	PPFIA1	Q13136	9
DSG2	Q14126	2	PPFIBP2	Q8ND30	9
DPYSL5	Q9BPU6	2	PPIA	P62937	9
DNM1L	O00429	2	PPP5C	P53041	9
DNHD1	DNHD1	2	PTGFRN	Q9P2B2	9
DNAJC3	Q13217	2	PTPN12	Q05209	9
DNAJC13	O75165	2	PTPN1	P18031	9
DNAJC10	Q8IXB1	2	PTPRB	P23467	9
DLGAP5	Q15398	2	PTPRC	P08575	9
DLAT	P10515	2	PTPRJ	Q12913	9
DHRS2	Q13268	2	PTPRO	Q16827	9
DGKQ	P52824	2	RAB3A	P20336	9
DGKK	Q5KSL6	2	RAC1	P63000	9
DGKI	O75912	2	RACGAP1	Q9H0H5	9
DGKH	Q86XP1	2	RAF1	P04049	9
DGKD	Q16760	2	RAP1A	P62834	9
DGKB	Q9Y6T7	2	RDX	P35241	9
DGKA	P23743	2	RHOA	P61586	9
DCTN5	Q9BTE1	2	RHOG	P84095	9
DCAF11	Q8TEB1	2	RNF32	Q9H0A6	9
DBNL	Q9UJU6	2	ROCK1	Q13464	9
DAGLB	Q8NCG7	2	RTN4	Q9NQC3	9
DACH1	Q9UI36	2	RTN4R	Q9BZR6	9
DAB2IP	Q5VWQ8	2	SCFD1	Q8WVM8	9
CYP2E1	P05181	2	SDCCAG3	Q96C92	9
CTSL2	O60911	2	SELE	P16581	9
CTSD	P07339	2	SERPINA3	P01011	9
CTR9	Q6PD62	2	SERPINA5	P05154	9
CSF1	P09603	2	SERPINF2	P08697	9
CRTAP	O75718	2	SFN	P31947	9
CPT1A	P50416	2	SH3GL3	Q99963	9
CPE	P16870	2	SH3KBP1	Q96B97	9
CPEB1	Q9BZB8	2	SHE	Q5VZ18	9
COPS4	Q9BT78	2	SLC17A7	Q9P2U7	9
COL6A3	P12111	2	SLC5A1	P13866	9
COL4A5	P29400	2	SMS	P52788	9
COL4A2	P08572	2	SNAP25	P60880	9
CLSTN1	O94985	2	SPA17	Q15506	9
CLGN	O14967	2	SPACA3	Q8IXA5	9
CLCN7	P51798	2	SPAG9	O60271	9
CKAP2	Q8WWK9	2	SPARCL1	Q14515	9
CIRH1A	Q969X6	2	SPARC	P09486	9
CHRNA7	P36544	2	SPP1	P10451	9
CGRRF1	Q99675	2	SRGAP3	O43295	9
CFDP1	Q9UEE9	2	STAM2	O75886	9
CDV3	Q9UKY7	2	STAT4	Q14765	9
CDK11B	P21127	2	STK39	Q9UEW8	9
CDK11A	Q9UQ88	2	TAB1	Q15750	9
CD63	P08962	2	TGFB1	P01137	9
CD276	Q5ZPR3	2	TGFB2	P61812	9
CCNF	P41002	2	TGFBR3	Q03167	9
CCHCR1	Q8TD31	2	TG	P01266	9
CCDC97	Q96F63	2	THBS1	P07996	9
CCDC58	Q4VC31	2	TIAM1	Q13009	9
CCDC14	Q49A88	2	TNC	P24821	9
CAT	P04040	2	TPD52L1	Q16890	9

CAPZA3	Q96KX2	2	TPD52L2	O43399	9
CAPZA2	P47755	2	TPD52L3	Q96J77	9
CAMLG	P49069	2	TRIB1	Q96RU8	9
C21orf59	P57076	2	TRIP6	Q15654	9
BTN3A3	O00478	2	TSKS	Q9UJT2	9
BPGM	P07738	2	TSPAN4	O14817	9
BLVRB	P30043	2	UBQLN1	Q9UMX0	9
BCAP29	Q9UHQ4	2	UBQLN4	Q9NRR5	9
BBS7	Q8IWZ6	2	VAPA	Q9P0L0	9
BAG5	Q9UL15	2	VAV2	P52735	9
BAG4	O95429	2	VPS13A	Q96RL7	9
ATP6V1E2	Q96A05	2	VTN	P04004	9
ATG4B	Q9Y4P1	2	VWF	P04275	9
ARPC5	O15511	2	WIPF2	Q8TF74	9
ARMC10	Q8N2F6	2	ZHX2	Q9Y6X8	9
ARL6	Q9H0F7	2	ACTL6A	O96019	10
ARL6IP5	O75915	2	APH1A	Q96BI3	10
ARL15	Q9NXU5	2	AURKB	Q96GD4	10
APPBP2	Q92624	2	BIRC5	O15392	10
APOB	P04114	2	BRD1	O95696	10
APOA2	P02652	2	BUB1	O43683	10
APEH	P13798	2	BUB3	O43684	10
APCS	P02743	2	CASC5	Q8NG31	10
APBB3	O95704	2	CDC20	Q12834	10
APBB2	Q92870	2	CDCA8	Q53HL2	10
APBA3	O96018	2	CENPA	P49450	10
APBA2	Q99767	2	CENPN	Q96H22	10
AP4S1	Q9Y587	2	CSNK2B	P67870	10
AMZ2	Q86W34	2	EIF6	P56537	10
AMOTL1	Q8IY63	2	FUNDC2	Q9BWH2	10
ALS2CR12	Q96Q35	2	HJURP	Q8NCD3	10
ALG2	Q9H553	2	INCENP	Q9NQS7	10
ALDH1A2	O94788	2	INO80B	Q9C086	10
AKAP8	O43823	2	ITGB3BP	Q13352	10
AKAP3	O75969	2	KAT5	Q92993	10
AIMP2	Q13155	2	KIAA1377	Q9P2H0	10
AIFM2	Q9BRQ8	2	KIF11	P52732	10
AGRN	O00468	2	KIF15	Q9NS87	10
AGPAT5	Q9NUQ2	2	KIF18A	Q8NI77	10
ADNP	Q9H2P0	2	KIF2B	Q8N4N8	10
ACOT7	O00154	2	KIF2C	Q99661	10
ACE	P12821	2	KIF9	Q9HAQ2	10
AATF	Q9NY61	2	KLC1	Q07866	10
ZPBP	Q9BS86	1	LPIN2	Q92539	10
ZP3	P21754	1	MKI67	P46013	10
ZNF711	Q9Y462	1	NDEL1	Q9GZM8	10
ZNF232	Q9UNY5	1	NFRKB	Q6P4R8	10
ZFHX4	ZFHX4	1	NPAT	Q14207	10
ZDHHC20	Q5W0Z9	1	NPM1	P06748	10
ZBTB39	ZBTB39	1	NUDC	Q9Y266	10
YBX2	Q9Y2T7	1	ODF2L	Q9ULJ1	10
WDR54	Q9H977	1	OIP5	O43482	10
VSIG4	Q9Y279	1	PPP1CA	P62136	10
VARS2	Q5ST30	1	PPP1CC	P36873	10
VAMP4	O75379	1	RORA	P35398	10
VAMP3	Q15836	1	RUVBL1	Q9Y265	10
VAMP1	P23763	1	RUVBL2	Q9Y230	10
USP47	Q96K76	1	SARNP	P82979	10
USP38	Q8NB14	1	SGOL2	Q562F6	10

UNC13B	O14795	1	TELO2	Q9Y4R8	10
UBR7	Q8N806	1	TOX4	O94842	10
UBE2Q1	Q7Z7E8	1	TRRAP	Q9Y4A5	10
TXNDC2	Q86VQ3	1	TTI1	O43156	10
TUBD1	Q9UJT1	1	TTI2	Q6NXR4	10
TTLL4	Q14679	1	XPO1	O14980	10
TTC7B	Q86TV6	1	YEATS4	O95619	10
TTC33	Q6PID6	1	ZNHIT2	Q9UHR6	10
TTC26	A0AVF1	1	ZWILCH	Q9H900	10
TSEN34	Q9BSV6	1	ACE2	Q9BYF1	11
TRIAP1	O43715	1	ADA	P00813	11
TRAF5	O00463	1	ADAP1	O75689	11
TPTE	P56180	1	ADCY1	Q08828	11
TPPP	O94811	1	ADCY2	Q08462	11
TOR1AIP2	Q8NFQ8	1	ADCY3	O60266	11
TOMM70A	O94826	1	ADCY4	Q8NFM4	11
TOMM5	Q8N4H5	1	ADCY5	O95622	11
TNFRSF21	O75509	1	ADCY6	O43306	11
TMPRSS12	TMPRSS12	1	ADCY7	P51828	11
TMEM67	Q5HYA8	1	ADCY8	P40145	11
TMEM57	Q8N5G2	1	ADCY9	O60503	11
TMEM30B	Q3MIR4	1	ADCYAP1	P18509	11
TMEM30A	Q9NV96	1	ADCYAP1R1	P41586	11
TMEM192	Q8IY95	1	ADM	P35318	11
TMEM191C	TMEM191C	1	ADORA1	P30542	11
TMEM191B	TMEM191B	1	ADORA2A	P29274	11
TMEM132A	Q24JP5	1	ADORA3	P33765	11
TMCC2	O75069	1	ADRA1A	P35348	11
TM2D1	Q9BX74	1	ADRA2A	P08913	11
THYN1	Q9P016	1	ADRB1	P08588	11
THUMPD3	Q9BV44	1	ADRBK1	P25098	11
THEM4	Q5T1C6	1	AGT	P01019	11
TDRKH	Q9Y2W6	1	AGTR1	P30556	11
TCF20	Q9UGU0	1	AGTR2	P50052	11
TBC1D8B	TBC1D8B	1	AMBP	P02760	11
TBC1D7	Q9P0N9	1	AMZ2	Q86W34	11
SYTL4	Q96C24	1	ANGPT1	Q15389	11
SYNRG	Q9UMZ2	1	ANXA1	P04083	11
SVIP	Q8NHG7	1	APH1B	Q8WW43	11
STX3	Q13277	1	APLN	Q9ULZ1	11
STX2	P32856	1	APLNR	P35414	11
STX1B	P61266	1	ARHGEF1	Q92888	11
STX1B1	STX1B1	1	ARHGEF25	Q86VW2	11
STX12	Q86Y82	1	ATG4B	Q9Y4P1	11
STX10	O60499	1	ATP2B4	P23634	11
SSPN	Q14714	1	ATPIF1	Q9UII2	11
SPON1	Q9HCB6	1	AVPR1A	P37288	11
SPEF2	Q9C093	1	BCL6	P41182	11
SORCS3	Q9UPU3	1	BDKRB1	P46663	11
SOAT1	P35610	1	BDKRB2	P30411	11
SNAP47	Q5SQN1	1	BTK	Q06187	11
SLFN5	Q08AF3	1	C1orf123	Q9NWV4	11
SLC4A1AP	Q9BWU0	1	C3AR1	Q16581	11
SLC45A4	SLC45A4	1	C3	P01024	11
SLC41A3	Q96GZ6	1	C5	P01031	11
SLC40A1	Q9NP59	1	CALCB	P10092	11
SLC32A1	Q9H598	1	CALCRL	Q16602	11
SLC2A5	P22732	1	CALM1	P62158	11
SLC2A3	P11169	1	CAMK2B	Q13554	11

SLC2A14	Q8TDB8	1	CCBP2	O00590	11
SLC1A2	P43004	1	CCDC14	Q49A88	11
SLC18A3	Q16572	1	CCKBR	P32239	11
SLC18A2	Q05940	1	CCL20	P78556	11
SIL1	Q9H173	1	CCL21	O00585	11
SHISA5	Q8N114	1	CCL27	Q9Y4X3	11
SEZ6L2	Q6UXD5	1	CCL28	Q9NRJ3	11
SETD4	Q9NVD3	1	CCL5	P13501	11
SESN3	P58005	1	CCR10	P46092	11
SERF1A	O75920	1	CCR2	P41597	11
SEC22C	Q9BRL7	1	CCR7	P32248	11
SEC22B	O75396	1	CGA	P01215	11
SEC14L2	O76054	1	CHRM2	P08172	11
SCO1	O75880	1	CHRM3	P20309	11
SCG3	Q8WXD2	1	CHRM5	P08912	11
SCCPDH	Q8NBX0	1	CNR1	P21554	11
SAMD9	Q5K651	1	CRHR2	Q13324	11
S100PBP	Q96BU1	1	CSAD	Q9Y600	11
S100A7A	S100A7A	1	CXCL10	P02778	11
RNF7	Q9UBF6	1	CXCL12	P48061	11
RNF34	Q969K3	1	CXCL16	Q9H2A7	11
RNF20	Q5VTR2	1	CXCL9	Q07325	11
RNF19B	Q6ZMZ0	1	CXCR3	P49682	11
RNF187	Q5TA31	1	CXCR4	P61073	11
RGS19	P49795	1	CXCR7	P25106	11
RGS12	O14924	1	CYP17A1	P05093	11
RELN	P78509	1	DAGLB	Q8NCG7	11
RASGRP3	Q8IV61	1	DGKA	P23743	11
RAPGEF2	Q9Y4G8	1	DGKB	Q9Y6T7	11
RAB5B	P61020	1	DGKD	Q16760	11
RAB2A	P61019	1	DGKE	P52429	11
RAB28	RAB28	1	DGKH	Q86XP1	11
RAB24	Q969Q5	1	DGKI	O75912	11
PXDNL	PXDNL	1	DGKK	Q5KSL6	11
PTPN9	P43378	1	DGKQ	P52824	11
PTPLB	Q6Y1H2	1	DGKZ	Q13574	11
PTDSS2	Q9BVG9	1	DLG1	Q12959	11
PSMG1	O95456	1	DLG4	P78352	11
PRSSL1	PRSSL1	1	DNAJC13	O75165	11
PRSS3	P35030	1	DRD3	P35462	11
PRSS2	P07478	1	DYSF	O75923	11
PRSS21	Q9Y6M0	1	EDN1	P05305	11
PRSS1	P07477	1	EDNRA	P25101	11
PRMT7	Q9NVM4	1	EDNRB	P24530	11
PRKRIP1	Q9H875	1	ELF1	P32519	11
PRKG2	Q13237	1	EREG	O14944	11
PREP	P48147	1	F2RL1	P55085	11
PPP3R2	Q96LZ3	1	F2RL2	O00254	11
PPP1R11	O60927	1	F2RL3	Q96RI0	11
PPOX	P50336	1	F2R	P25116	11
POP7	O75817	1	FOSL2	P15408	11
PLP2	Q04941	1	FOXE1	O00358	11
PLA2G6	O60733	1	FPR2	P25090	11
PITPNM1	O00562	1	FPR3	P25089	11
PIP5K1A	Q99755	1	FSHB	P01225	11
PIGV	Q9NUD9	1	FSHR	P23945	11
PI4K2A	Q9BTU6	1	GABBR1	Q9UBS5	11
PGM3	O95394	1	GABBR2	O75899	11
PGK2	P07205	1	GALR1	P47211	11

PGAM1	P18669	1	GHRL	Q9UBU3	11
PDZK1P1	PDZK1P1	1	GHSR	Q92847	11
PDE6A	P16499	1	GNA11	P29992	11
PDE5A	O76074	1	GNA12	Q03113	11
PDE10A	Q9Y233	1	GNA13	Q14344	11
PCYT2	Q99447	1	GNA14	O95837	11
PARG99	PARG99	1	GNA15	P30679	11
PAPOLB	Q9NRJ5	1	GNAI1	P63096	11
PAK3	O75914	1	GNAI2	P04899	11
P2RX7	Q99572	1	GNAI3	P08754	11
OSTC	Q9NRP0	1	GNAQ	P50148	11
OSMR	Q99650	1	GNAS	P63092	11
ORMDL2	Q53FV1	1	GNAT2	P19087	11
OR4N4	Q8N0Y3	1	GNAZ	P19086	11
OCLN	Q16625	1	GNB1	P62873	11
NXT2	Q9NPJ8	1	GNG2	P59768	11
NUDT2	P50583	1	GNRH1	P01148	11
NUDT12	Q9BQG2	1	GNRHR2	Q96P88	11
NTF4	P34130	1	GNRHR	P30968	11
NSUN3	Q9H649	1	GPBAR1	Q8TDU6	11
NSDHL	Q15738	1	GPER	Q99527	11
NPTXR	O95502	1	GPR18	Q14330	11
NPTX2	P47972	1	GPR44	GPR44	11
NPTX1	Q15818	1	GPR4	P46093	11
NOV	P48745	1	GPR55	Q9Y2T6	11
NME5	P56597	1	GPR65	Q8IYL9	11
NHLRC1	Q6VVB1	1	GPR68	Q15743	11
NFE2L1	Q14494	1	GPRC6A	Q5T6X5	11
NF1	P21359	1	GPSM2	P81274	11
NES	P48681	1	GRIK3	Q13003	11
NEK10	Q6ZWH5	1	GRIN2B	Q13224	11
NEFH	P12036	1	GRK4	P32298	11
NECAB3	Q96P71	1	GRK5	P34947	11
NDUFS1	P28331	1	GRM1	Q13255	11
NDRG3	Q9UGV2	1	GRM2	Q14416	11
NAT14	Q8WUY8	1	GRM4	Q14833	11
MYT1	Q01538	1	GRP	P07492	11
MUM1L1	MUM1L1	1	GRPR	P30550	11
MTX2	O75431	1	HCAR2	Q8TDS4	11
MSR1	P21757	1	HCRT	O43612	11
MRAS	O14807	1	HDAC7	Q8WUI4	11
MPDZ	O75970	1	HEBP1	Q9NRV9	11
MORC1	Q86VD1	1	HRH1	P35367	11
MNDA	P41218	1	HRH3	Q9Y5N1	11
MMP17	Q9ULZ9	1	HRH4	Q9H3N8	11
MFF	Q9GZY8	1	HSD17B3	P37058	11
MFAP5	Q13361	1	HTR2A	P28223	11
MFAP3L	O75121	1	HTR5A	P47898	11
MEX3C	Q5U5Q3	1	HTR7	P34969	11
METAP1	P53582	1	IAPP	P10997	11
MEFV	O15553	1	IL1RAP	Q9NPH3	11
MAP3K2	Q9Y2U5	1	INSL3	P51460	11
MAP2K2	P36507	1	ITGAX	P20702	11
MAGEB1	MAGEB1	1	ITPR1	Q14643	11
LZTFL1	Q9NQ48	1	ITPR2	Q14571	11
LYPD3	O95274	1	ITPR3	Q14573	11
LTBP4	Q8N2S1	1	JAG2	Q9Y219	11
LRWD1	Q9UFC0	1	JUNB	P17275	11
LRRC37B	Q96QE4	1	JUND	P17535	11

LRRC14	Q15048	1	KIDINS220	Q9ULH0	11
LRP6	O75581	1	KISS1	Q15726	11
LRP1	Q07954	1	KLHL23	Q8NBE8	11
LOXL2	Q9Y4K0	1	LHB	P01229	11
LMBRD2	LMBRD2	1	LHCGR	P22888	11
LCORL	Q8N3X6	1	LIN7A	O14910	11
LATS1	O95835	1	LPAR1	Q92633	11
LAMC2	Q13753	1	LPAR2	Q9HBW0	11
KLHL9	Q9P2J3	1	LPAR3	Q9UBY5	11
KLHL7	Q8IXQ5	1	LPAR4	Q99677	11
KLHL24	Q6TFL4	1	LPAR6	P43657	11
KLHL18	O94889	1	LTB4R2	Q9NPC1	11
KLHL15	Q96M94	1	MC1R	Q01726	11
KLHL11	Q9NVR0	1	MC5R	P33032	11
KIAA1704	Q8IXQ4	1	MCHR1	Q99705	11
KIAA0319L	Q8IZA0	1	MCHR2	Q969V1	11
KCTD7	Q96MP8	1	METAP2	P50579	11
KCNE3	Q9Y6H6	1	MLL4	MLL4	11
KBTBD4	Q9NVX7	1	MMEL1	Q495T6	11
KATNAL1	Q9BW62	1	MRAS	O14807	11
ITM2B	Q9Y287	1	MTNR1A	P48039	11
ITM2A	O43736	1	NCSTN	Q92542	11
ITGAM	P11215	1	NMB	P08949	11
IMPACT	Q9P2X3	1	NMBR	P28336	11
IL32	P24001	1	NMU	P48645	11
IL1RAP	Q9NPH3	1	NMUR1	Q9HB89	11
IL13RA2	Q14627	1	NMUR2	Q9GZQ4	11
IGSF21	Q96ID5	1	NOS1	P29475	11
IGFBP7	Q16270	1	NOTCH1	P46531	11
IFT81	Q8WYA0	1	NOTCH2	Q04721	11
IFT74	Q96LB3	1	NOTCH3	Q9UM47	11
IFT52	Q9Y366	1	NPB	Q8NG41	11
IFT122	Q9HBG6	1	NPBWR1	P48145	11
HTRA1	Q92743	1	NPFF	O15130	11
HTR3A	P46098	1	NPFFR1	Q9GZQ6	11
HSPB6	O14558	1	NPFFR2	Q9Y5X5	11
HN1	Q9UK76	1	NPSR1	Q6W5P4	11
HMGA2	P52926	1	NPW	Q8N729	11
HLA-A	P18462	1	NPY1R	P25929	11
HINT3	Q9NQE9	1	NPY5R	Q15761	11
HHEX	Q03014	1	NRG1	Q02297	11
HFE	Q30201	1	NRG4	Q8WWG1	11
HENMT1	Q5T8I9	1	NTSR2	O95665	11
HAS3	O00219	1	NUDT12	Q9BQG2	11
HACL1	Q9UJ83	1	OPN4	Q9UHM6	11
GUCY1B3	Q02153	1	OPRK1	P41145	11
GUCY1A3	Q02108	1	OR4N4	Q8N0Y3	11
GRM2	Q14416	1	OXER1	Q8TDS5	11
GRIP2	Q9C0E4	1	OXT	P01178	11
GRIA4	P48058	1	P01213	P01213	11
GRIA2	P42262	1	P2RY11	Q96G91	11
GPSM2	P81274	1	P2RY12	Q9H244	11
GPS1	Q13098	1	P2RY13	Q9BPV8	11
GPNMB	Q14956	1	P2RY14	Q15391	11
GPC1	P35052	1	P2RY1	P47900	11
GPAT2	GPAT2	1	PCSK7	Q16549	11
GPANK1	O95872	1	PDPK1	O15530	11
GOSR1	O95249	1	PDYN	PDYN	11
GNAT2	P19087	1	PENK	PENK	11

GFER	P55789	1	PIK3CA	P42336	11
GAS7	O60861	1	PIK3CG	P48736	11
FOXE1	O00358	1	PIK3R2	O00459	11
FIS1	Q9Y3D6	1	PIK3R3	Q92569	11
FCHSD1	Q86WN1	1	PLCB1	Q9NQ66	11
FBXO46	Q6PJ61	1	PLCB2	Q00722	11
FBXO2	Q9UK22	1	PLCB3	Q01970	11
FBXO27	Q8NI29	1	PLCB4	Q15147	11
FBXO22	Q8NEZ5	1	PMCH	P20382	11
FBXO17	Q96EF6	1	POMC	P01189	11
FBRS	Q9HAH7	1	PPP3CA	Q08209	11
FAM54A	FAM54A	1	PRKCE	Q02156	11
FAM188A	Q9H8M7	1	PRKCH	P24723	11
FAM184A	Q8NB25	1	PRKD1	Q15139	11
FAM135A	Q9P2D6	1	PRKG2	Q13237	11
FABP7	O15540	1	PRMT7	Q9NVM4	11
F13A1	P00488	1	PROK1	P58294	11
F12	P00748	1	PROK2	Q9HC23	11
ERO1LB	Q86YB8	1	PROKR1	Q8TCW9	11
EPM2A	O95278	1	PROKR2	Q8NFJ6	11
EPHA10	EPHA10	1	PSENEN	Q9NZ42	11
EPB41L2	O43491	1	PTAFR	P25105	11
ELOF1	P60002	1	PTGER2	P43116	11
EGFLAM	Q63HQ2	1	PTGER3	P43115	11
EFEMP1	Q12805	1	PTGER4	P35408	11
EFCAB11	Q9BUY7	1	PTGFR	P43088	11
DYSF	O75923	1	PTGIR	P43119	11
DVL3	Q92997	1	PTH1R	Q03431	11
DPH2	Q9BQC3	1	PTH2R	P49190	11
DPEP1	P16444	1	PTHLH	P12272	11
DOPEY1	Q5JWR5	1	PYY	P10082	11
DOLK	Q9UPQ8	1	QRFP	P83859	11
DOCK7	Q96N67	1	RAMP1	O60894	11
DOC2B	Q14184	1	RAMP3	O60896	11
DOC2A	Q14183	1	RASA2	Q15283	11
DNAJC5	Q9H3Z4	1	RASGRP1	O95267	11
DNAJC28	Q9NX36	1	RASGRP2	Q7LDG7	11
DNAJC1	Q96KC8	1	RASGRP3	Q8IV61	11
DNAJC17	Q9NVM6	1	RGS12	O14924	11
DNAH1	Q9P2D7	1	RGS19	P49795	11
DNAAF1	Q8NEP3	1	RGS2	P41220	11
DLG5	Q8TDM6	1	RGS6	P49758	11
DIP2B	Q9P265	1	RLN2	P04090	11
DIP2A	Q14689	1	RLN3	Q8WXF3	11
DHX35	DHX35	1	RPS6KB2	Q9UBS0	11
DHDDS	Q86SQ9	1	RXFP1	Q9HBX9	11
DENND4C	DENND4C	1	RXFP2	Q8WXD0	11
DCLK1	O15075	1	RXFP3	Q9NSD7	11
DCBLD1	Q8N8Z6	1	RXFP4	Q8TDU9	11
DAZ3	Q9NR90	1	S1PR1	P21453	11
DAZ2	Q13117	1	S1PR2	O95136	11
DAPP1	Q9UN19	1	S1PR3	Q99500	11
CTGF	P29279	1	S1PR5	Q9H228	11
CTAG2	O75638	1	SCT	P09683	11
CSNK1G3	Q9Y6M4	1	SEC14L2	O76054	11
CSAD	Q9Y600	1	SERF1A	O75920	11
CRYZL1	O95825	1	SET	Q01105	11
CRYGC	P07315	1	SIVA1	O15304	11
CRYBB2	P43320	1	SLC2A5	P22732	11

CR2	P20023	1	SLC9A3R1	O14745	11
CPLX1	O14810	1	SLC9A3R2	Q15599	11
COPS7B	Q9H9Q2	1	SMPD2	O60906	11
COL4A6	Q14031	1	SSTR2	P30874	11
COL4A3	Q01955	1	SSTR3	P32745	11
COL4A1	P02462	1	SYK	P43405	11
COL18A1	P39060	1	TAC1	TAC1	11
CNTN4	Q8IWV2	1	THEM4	Q5T1C6	11
CNTN3	Q9P232	1	TPM2	P07951	11
CNN2	Q99439	1	TPM4	P67936	11
CLUAP1	Q96AJ1	1	TRH	P20396	11
CLSTN3	Q9BQT9	1	TRIO	O75962	11
CLPTM1L	Q96KA5	1	TRPC1	P48995	11
CIAPIN1	Q6FI81	1	TRPC6	Q9Y210	11
CHRNB1	P11230	1	TRPC7	Q9HCX4	11
CHL1	O00533	1	TRPM7	Q96QT4	11
CGN	Q9P2M7	1	TTC1	Q99614	11
CDCA2	Q69YH5	1	USP3	Q9Y6I4	11
CCNDBP1	O95273	1	USP47	Q96K76	11
CCDC86	Q9H6F5	1	USPL1	Q5W0Q7	11
CCDC42	Q96M95	1	UTRN	P46939	11
CCBP2	O00590	1	UTS2D	Q765I0	11
CATSPERG	Q6ZRH7	1	UTS2	O95399	11
CASP8	Q14790	1	VARS2	Q5ST30	11
CALU	O43852	1	VCAN	P13611	11
CACNG4	Q9UBN1	1	VIP	P01282	11
CACNG2	Q9Y698	1	VIPR1	P32241	11
CACNB4	O00305	1	VIPR2	P41587	11
CACNB3	P54284	1	VSIG4	Q9Y279	11
CACNA2D2	Q9NY47	1	WBP7	Q9UMN6	11
CACNA1B	Q00975	1	WDR35	Q9P2L0	11
CABYR	O75952	1	AMFR	Q9UKV5	12
C5orf35	C5orf35	1	ASCC2	Q9H1I8	12
C2CD2	Q9Y426	1	ATF4	P18848	12
C21orf91	C21orf91	1	ATF6	P18850	12
C20orf111	Q9NX31	1	ATP6AP1	Q15904	12
C1orf173	Q5RHP9	1	BIRC2	Q13490	12
C1orf123	Q9NWV4	1	CAPN11	Q9UMQ6	12
C1orf112	Q9NSG2	1	CAPNS1	P04632	12
C19orf51	C19orf51	1	CKAP4	Q07065	12
C19orf42	C19orf42	1	CUL2	Q13617	12
C18orf21	Q32NC0	1	DERL1	Q9BUN8	12
C17orf56	Q96N21	1	DERL2	Q9GZP9	12
C17orf53	Q8N3J3	1	ERLIN1	O75477	12
C16orf78	C16orf78	1	ERN1	O75460	12
C15orf23	C15orf23	1	FAF1	Q9UNN5	12
C13orf27	C13orf27	1	FBXW11	Q9UKB1	12
C13orf15	C13orf15	1	G6PD	P11413	12
BTF3	P20290	1	GET4	Q7L5D6	12
BRSK1	Q8TDC3	1	GSR	P00390	12
BRPF1	P55201	1	HERPUD1	Q15011	12
BGN	P21810	1	HFE	Q30201	12
BEX1	Q9HBH7	1	KLHDC3	Q9BQ90	12
BCDIN3D	Q7Z5W3	1	KLHL22	Q53GT1	12
BASP1	P80723	1	LRR1	Q96L50	12
BACE2	Q9Y5Z0	1	OTUB1	Q96FW1	12
ATRN	O75882	1	PC	P11498	12
ATPIF1	Q9UII2	1	PLA2G2A	P14555	12

ATP8B3	O60423	1	PLAA	Q9Y263	12
ATP6AP1	Q15904	1	PPM1E	Q8WY54	12
ATG4D	Q86TL0	1	PRKAR1A	P10644	12
ATCAY	Q86WG3	1	PRKCSH	P14314	12
ASCC3	Q8N3C0	1	SEL1L	Q9UBV2	12
ASCC1	Q8N9N2	1	SELS	SELS	12
ARFGAP1	Q8N6T3	1	SGTA	O43765	12
APMAP	Q9HDC9	1	SNCAIP	Q9Y6H5	12
ANGPT1	Q15389	1	SVIP	Q8NHG7	12
ANGEL1	Q9UNK9	1	SYVN1	Q86TM6	12
AMBP	P02760	1	TMUB1	Q9BVT8	12
ALG8	Q9BVK2	1	TNIK	Q9UKE5	12
ALDOC	P09972	1	UBE2J1	Q9Y385	12
AKT3	Q9Y243	1	UBE2J2	Q8N2K1	12
AKAP2	Q9Y2D5	1	UBE2S	Q16763	12
AKAP1	Q92667	1	UBE2Z	Q9H832	12
AGPS	O00116	1	UBR2	Q8IWV8	12
AGER	Q15109	1	UBXN6	Q9BZV1	12
AGA	P20933	1	UBXN7	O94888	12
ADO	Q96SZ5	1	UCHL1	P09936	12
ADAMTSL2	Q86TH1	1	UFD1L	Q92890	12
ADAM8	P78325	1	USP21	Q9UK80	12
ADAM33	Q9BZ11	1	USP50	Q70EL3	12
ACSL5	Q9ULC5	1	VCP	P55072	12
ACBD3	Q9H3P7	1	WFS1	O76024	12
ABCB1	P08183	1	XBP1	P17861	12

(c)

APP interactors in YTH	degree	clustering coefficient	betweenness centrality	*k*-core	module
ACBD3	1	0	0	1	2
SYNRG	1	0	0	1	2
ATF6	3	1	0	3	12
BCAP29	3	0.333333	0.000036	2	2
BNIP2	5	0.3	0.000058	5	9
APMAP	1	0	0	1	2
CAMLG	2	0	0.000001	2	9
CCHCR1	2	0	0.000459	2	2
CD81	27	0.02849	0.005357	8	9
CD99	13	0.064103	0.001072	6	9
CHRNB1	1	0	0	1	2
CLPTM1L	1	0	0	1	2
COPS5	152	0.014291	0.045504	17	6
CREB3	3	0.333333	0.000011	3	2
OSTC	1	0	0	1	2
DPEP1	1	0	0	1	2
FAM134A	4	0	0.000096	4	3
FANCM	5	0.6	0.000079	5	5
FTL	4	0.166667	0.000029	4	2
GPNMB	1	0	0	1	2
ITGB5	10	0.088889	0.000296	8	2
KIAA0319L	1	0	0	1	2
KIAA1704	1	0	0	1	2
LYPD3	1	0	0	1	2
RANBP9	8	0.071429	0.000124	6	3
RPN2	6	0.266667	0.000071	6	1
SSPN	1	0	0	1	2
SEC22C	1	0	0	1	2
STX5	4	0.166667	0.000043	4	4
TAF15	5	0.2	0.000076	5	1
TBC1D7	1	0	0	1	2
TMPRSS12	1	0	0	1	2
TOMM5	1	0	0	1	2
TSPAN6	2	0	0.000008	2	2
TMEM191C	1	0	0	1	2
TMEM191B	1	0	0	1	2
PDZK1P1	1	0	0	1	2
