# Supplementary figures and images for: Amyloid precursor protein interaction network in human testis: sentinel proteins for male reproduction
Source: BMC Bioinformatics. 2015 Jan 16;16(1):12. doi: 10.1186/s12859-014-0432-9 (PMC4384327; doi:10.1186/s12859-014-0432-9)

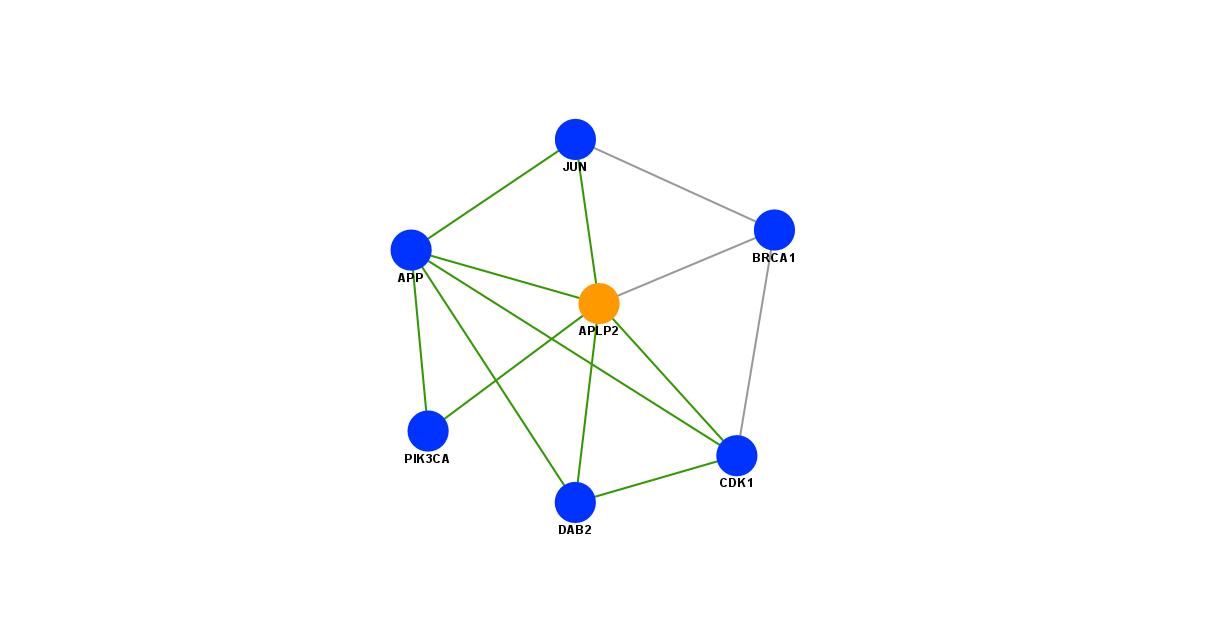

Supplement: Additional file 7: Figure S1 — Modular structures of poteins sharing APP-APLP2 interaction. Four common proteins interacting with APP and APLP2 form triangle or square modules (indicated by green links) which shows that they can be highly possible functional modules. Blue nodes are testis-specific and orange node (APLP2) is not. [file 12859_2014_432_MOESM7_ESM.jpeg]
